# Humanized *Candida* and NanoBiT Assays Expedite Discovery of Bdf1 Bromodomain Inhibitors With Antifungal Potential

**DOI:** 10.1002/advs.202404260

**Published:** 2025-01-16

**Authors:** Kaiyao Wei, Marie Arlotto, Justin M. Overhulse, Tuan‐Anh Dinh, Yingsheng Zhou, Nathan J. Dupper, Jiayi Yang, Boris A. Kashemirov, Hasan Dawi, Cécile Garnaud, Gaëlle Bourgine, Flore Mietton, Morgane Champleboux, Amédé Larabi, Yordan Hayat, Rose‐Laure Indorato, Marjolaine Noirclerc‐Savoye, Dimitrios Skoufias, Muriel Cornet, Gwenaël Rabut, Charles E. McKenna, Carlo Petosa, Jérôme Govin

**Affiliations:** ^1^ Univ. Grenoble Alpes CEA CNRS Institut de Biologie Structurale (IBS) Grenoble 38000 France; ^2^ Univ. Grenoble Alpes Inserm CNRS Institute for Advanced Biosciences (IAB) Grenoble 38000 France; ^3^ Department of Chemistry Dana and David Dornsife College of Letters Arts, and Sciences University of Southern California University Park Campus Los Angeles CA 90089 USA; ^4^ Univ. Grenoble Alpes CNRS Grenoble INP CHU Grenoble Alpes, Laboratoire TIMC Grenoble 38000 France; ^5^ Univ. Rennes CNRS INSERM Institut de Génétique et Développement de Rennes (IGDR) UMR 6290, U1305 Rennes 35000 France

**Keywords:** antifungal, Bdf1, BET inhibition, bromodomain, *Candida*

## Abstract

The fungal Bromodomain and Extra‐Terminal (BET) protein Bdf1 is a potential antifungal target against invasive fungal infections. However, the need to selectively inhibit both Bdf1 bromodomains (BDs) over human orthologs and the lack of molecular tools to assess on‐target antifungal efficacy hamper efforts to develop Bdf1 BD inhibitors as antifungal therapeutics. This study reports a phenyltriazine compound that inhibits both Bdf1 BDs from the human fungal pathogen *Candida glabrata* with selectivity over the orthologous BDs from the human BET protein Brd4. On‐target antifungal activity is established by devising two yeast‐based inhibition assays: a growth assay using humanized *Candida* strains in which the Bdf1 BDs are replaced by their Brd4 counterparts, and a NanoBiT assay that evaluates the BD‐mediated association of Bdf1 with chromatin. These assays additionally enable the discovery that BET inhibitor I‐BET726 targets both Bdf1 BDs, inhibits the growth of a broad spectrum of *Candida* species, including antifungal‐resistant clinical isolates, and displays efficacy in an invertebrate animal model of infection. These collective findings highlight the promising potential of Bdf1 BD inhibitors as an innovative class of antifungal therapeutics and the pivotal role of yeast‐based assay development toward achieving this end.

## Graphical abstract

The fungal BET protein Bdf1 is a potential antifungal target in pathogenic *Candida* species requiring the inhibition of both its bromodomains. Humanized *Candida* strains and NanoBiT complementation assays developed to evaluate on‐target antifungal efficacy expedite the discovery of Bdf1 bromodomain inhibitors with antifungal potential, supporting BET inhibition as a promising strategy to combat invasive *Candida* infection.

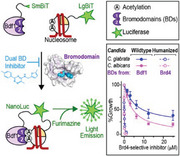

## 1 Introduction

Invasive fungal infections (IFIs) are a significant cause of morbidity and mortality,^[^ ^1^ ^]^ posing a major global public health challenge due to the limited repertoire of current antifungal drugs.^[^ ^2^ ^]^ *Candida* species are responsible for ≈700 000 IFIs per year, yielding 40% mortality and a heavy economic burden,^[^ ^1^, ^3^ ^]^ with an alarming increase in candidemia cases in recent years.^[^ ^3^, ^4^ ^]^ In Europe and North America, the first and second most frequently isolated *Candida* species are *C. albicans* and *C. glabrata*, respectively, accounting for ≈70% of all systemic candidiasis.^[^ ^4^, ^5^ ^]^ The rapidly emerging multidrug‐resistant fungal strain *Candida auris* epitomizes the threat posed by IFIs.^[^ ^6^ ^]^ *C. auris*, *C. albicans* and *C. glabrata* rank among the top five pathogens on the World Health Organization's Fungal Priority Pathogens (FPP) List.^[^ ^7^ ^]^

Small‐molecule inhibitors targeting chromatin signaling pathways (“epi‐drugs”) are intensely studied as potential therapeutics against cancer and other diseases.^[^ ^8^ ^]^ Considerable efforts have focused on the Bromodomain and Extra‐Terminal (BET) family of proteins, which regulate gene transcription and chromatin organization.^[^ ^9^ ^]^ In mammals this family comprises four proteins (Brd2, Brd3, Brd4 and Brdt), of which the best studied is Brd4. BET proteins bind chromatin through their two bromodomains (BDs), BD1 and BD2, which specifically recognize histones acetylated on lysines.^[^ ^10^ ^]^ Numerous small‐molecule BET inhibitors (BETi) have been developed, including several currently in phase II or phase III clinical trials for cancer, diabetes and cardiovascular disease.^[^ ^8^, ^11^ ^]^ While many BET inhibitors target both the BD1 and BD2 domains of human BET proteins, efforts are increasingly focused on domain‐selective compounds to avoid the dose‐limiting toxicities observed with pan‐BET inhibitors (reviewed in ref.[12]).

Except for certain histone deactylases (HDACs), epigenetic targets have largely remained unexplored in the fungal infection field.^[^ ^13^ ^]^ One attractive target is the fungal BET protein Bdf1, a global transcriptional regulator.^[^ ^14^ ^]^ In *S. cerevisiae*,^[^ ^15^ ^]^ Bdf1 interacts with the transcription factor TFIID,^[^ ^16^ ^]^ is a subunit of the SWR1 chromatin remodeling complex,^[^ ^17^ ^]^ and plays a key role in the salt stress response^[^ ^18^ ^]^ and in regulating transcription and chromatin compaction during yeast sporulation.^[^ ^19^ ^]^ We previously showed that Bdf1 is essential in *C. albicans* and that mutations simultaneously inactivating both BDs are lethal.^[^ ^20^ ^]^ We identified small molecules that inhibit individual Bdf1 BDs with selectivity over human BDs. Compounds selective for Bdf1 BD1 showed antifungal activity when tested on *C. albicans* strains harboring inactivating mutations in BD2, and vice versa, indicating Bdf1 as a potential antifungal drug target.

However, a major obstacle to the development of antifungal BET inhibitors is the requirement for a fungal‐selective dual BD inhibitor (a compound that inhibits both Bdf1 BDs with selectivity over human BET BDs). This challenge arises because the ligand binding pockets of Bdf1 BD1 and BD2 are more divergent than those of their orthologous human BET BDs. This problem is exacerbated by the difficulty in establishing on‐target antifungal efficacy. While the on‐target activity of a small molecule that inhibits only a single Bdf1 BD (i.e., either BD1 or BD2) can be shown using a fungal strain expressing Bdf1 mutated in the non‐targeted BD (i.e., BD2 or BD1, respectively),^[^ ^20^ ^]^ such a strategy is ineffective for a dual BD inhibitor (since mutating both BDs is lethal). Additionally, it is unclear whether Bdf1 BD inhibition would be effective against antifungal‐resistant strains as well as non‐*albicans Candida* species.

The present study addresses these issues. We investigate Bdf1 inhibition in *C. glabrata*, which is phylogenetically, genetically and phenotypically very different from *C. albicans*, more closely resembling *S. cerevisiae*.^[^ ^21^ ^]^ *C. glabrata* presents clinical challenges because of its inherent low susceptibility to azole drugs and rapid development of multidrug resistance under antifungal pressure. We show that Bdf1 is essential in *C. glabrata*, and that inactivation of both Bdf1 BDs induces lethality. We identified a phenyltriazine compound that inhibits both Bdf1 BDs selectively over human BET BDs and determined the structural basis of its selectivity. We engineered a *C. glabrata* strain expressing a humanized Bdf1 variant to demonstrate on‐target antifungal activity, which we additionally confirmed by developing a yeast‐based NanoBiT complementation assay. The humanized *Candida* and NanoBiT assays further enabled the discovery that BET inhibitor I‐BET726 possesses antifungal activity against a wide range of *Candida* species, including drug‐resistant clinical isolates, and exhibits efficacy in an invertebrate model of systemic *Candida* infection. Taken together our findings support the feasibility of developing Bdf1 BD inhibitors as antifungal therapeutics while highlighting the role of yeast‐based assays in facilitating this goal.

## 2 Results and Discussion

### 2.1 Lethality of Dual Bdf1 BD Inactivation and Sensitivity to BET Inhibitors

We used a peptide array comprising histone sequences with diverse post‐translational modifications to probe the ligand selectivity of *C. glabrata* Bdf1 (*Cg*Bdf1) BD1 and BD2 (*Cg*BD1 and *Cg*BD2; **Figure** 4). Like other BET BDs,^[^ ^10^, ^19^, ^20^ ^]^ the two *Cg*BDs displayed weak binding to mono‐acetylated peptides, enhanced binding to di‐ and tri‐acetylated peptides, and strongest binding to a tetra‐acetylated H4 peptide (H4ac4; acetylated on K5, K8, K12 and K16) (Figure S1A–C, Supporting Information). Substituting a conserved tyrosine implicated in acetyllysine recognition^[^ ^15^, ^19^, ^20^ ^]^ by a phenylalanine (“Y/F” mutations Y166F and Y343F) abolished the interaction with acetylated peptides on the array (Figure S1B,C, Supporting Information) and in pull‐down assays (Figure S1D, Supporting Information), demonstrating binding specificity. Crystal structures of *Cg*BD1 and *Cg*BD2 confirmed the expected BD fold (comprising helices Z, A, B and C), allowed comparison with human BET and *C. albicans* Bdf1 (*Ca*Bdf1) BD structures (Figure S2A,B and Tables S1 and S2, Supporting Information), and revealed details of the ligand binding pockets defined by the ZA and BC loops, including six structurally conserved water molecules (Figure S2C,D, Supporting Information).

**Figure 1:**
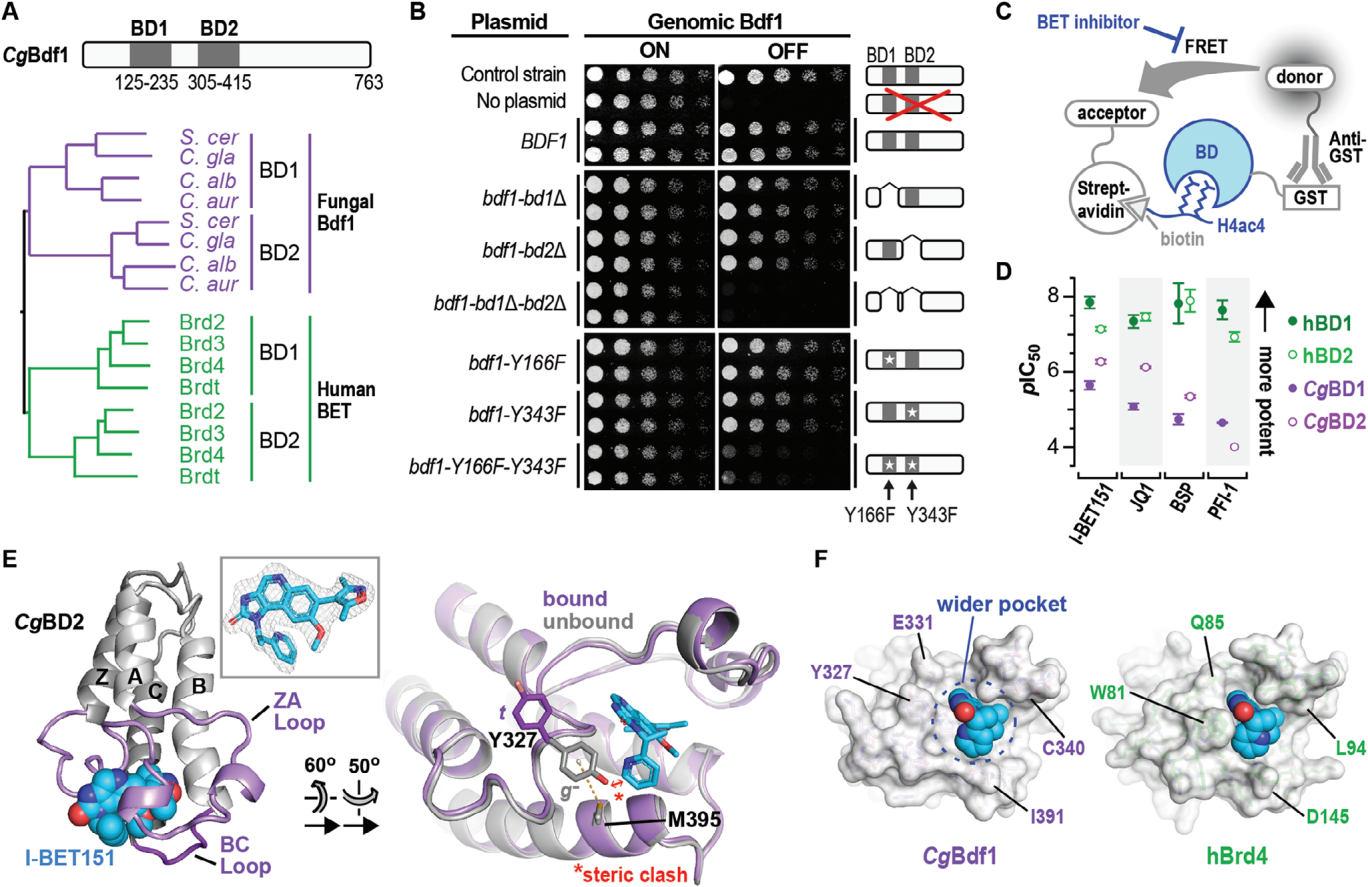
Lethality of dual Bdf1 BD inactivation and sensitivity to BET inhibitors. A) Domain boundaries of *Cg*Bdf1 BDs and phylogeny of BET BDs. Fungal species listed are *S. cerevisiae*, *C. glabrata*, *C. albicans* and *C. auris*. B) Colony formation assays showing the effect of Bdf1 deletion or Bdf1 BD inactivation on *C. glabrata* growth. C) HTRF assay. The binding of a GST‐tagged BET BD (bound to an anti‐GST antibody coupled to the donor fluorophore) to a biotinylated H4ac4 peptide (bound to streptavidin coupled to the acceptor fluorophore) yields a FRET signal, which is reduced in the presence of a BET inhibitor. D) Summary of *p*IC_50_ values determined for BET inhibitors against Brd4 and *Cg*Bdf1 BDs. BSP, bromosporine. E) *Left*. Crystal structure of *Cg*BD2 bound to I‐BET151. *Inset*. Simulated annealing omit *F*_o_‐*F*_c_ density for I‐BET151 contoured at 3.5 σ. *Right*. Structural alignment of *Cg*BD2 in the unbound state (grey) and bound to I‐BET151 (violet) (rmsd_108Cα_ = 0.418 Å) showing the Tyr327 *gauche−* (*g−*) and *trans* (*t*) conformations. Dashed line: sulfur‐π interaction. F) Surface representations showing the wider binding pocket of *Cg*BD2 (left) compared to Brd4 BD1 (right).

To verify whether Bdf1 BDs are essential in *C. glabrata*, we replaced the endogenous *BDF1* promoter by the *MET3* gene promoter (*pMET*), which is inhibited by adding excess methionine and cysteine to the growth medium^[^ ^22^ ^]^ (Figure S3A, Supporting Information). Immunoblotting confirmed effective repression of *pMET‐BDF1* by methionine and cysteine, with most Bdf1 protein disappearing within 8 h (Figure S3B,C, Supporting Information). Colony formation and liquid growth assays showed that the absence of Bdf1 protein led to lethality, which was rescued by ectopic expression from an autonomous plasmid, confirming that Bdf1 is essential for *C. glabrata* growth in vitro (Figure 1B; Figure S3D, Supporting Information). We subsequently generated *Cg*Bdf1 mutants lacking one or both BDs or containing one or both Y/F point mutations and verified that their introduction did not strongly affect Bdf1 expression levels (Figure S3E, Supporting Information). Notably, whereas disrupting only a single BD did not markedly affect growth, mutating both was lethal (Figure 1B; Figure S3D, Supporting Information). Hence, dual BD inactivation is required to compromise viability.

We next assessed the ability of small molecules to differentially inhibit human BET and *Cg*Bdf1 BDs by evaluating the ability of BETi compounds JQ1, I‐BET151, bromosporine (BSP) and PFI‐1 to inhibit binding to an H4ac4 peptide in a homogeneous time‐resolved fluorescence (HTRF) assay^[^ ^20^ ^]^ (Figure 1C). While these compounds potently inhibited human Brd4 BD1 and BD2 (IC_50_ values of ≈10–100 nM), consistent with previous reports,^[^ ^23^ ^]^ they inhibited *Cg*Bdf1 BDs more weakly (IC_50_ values between 0.5 and >100 µM; i.e., a 10‐ to 1000‐fold reduction in sensitivity) (Figure 1D; Figure S4A, Supporting Information) and failed to inhibit *C. glabrata* growth in vitro (Figure S4B, Supporting Information). These results demonstrate that small molecules can strongly discriminate between human BET and *Cg*Bdf1 BDs.

To rationalize these data, we determined the crystal structure of the highest affinity *Cg*BD/BETi complex, *Cg*BD2 bound to I‐BET151 (Table S1, Supporting Information). *Cg*BD2 undergoes a minor conformational change to accommodate IBET‐151: in unbound *Cg*BD2, the side chain of “YPF‐shelf” residue Tyr327 adopts a *gauche* minus (*g−*) conformation, stabilized by a sulfur‐π interaction with residue Met395, whereas, in the complex, Tyr327 adopts the *trans* conformation to avoid a steric clash with I‐BET151, thereby widening the ligand binding pocket (Figure 1E). I‐BET151 adopts the same pose as previously observed with the BD1 domains of human Brd2 and Brd4^[^ ^23^, ^24^ ^]^ (Figure S5A,B, Supporting Information) but interacts less intimately with the *Cg*BD2 binding pocket because of the lack of contact with Tyr327, whereas the corresponding human WPF‐shelf Trp residue (Trp51 in Brd4) packs against the pyridine and imidazoquinoline rings (Figure S5C, Supporting Information). The shorter side chains (Cys340, Val392) and altered conformation (Glu331) of three *Cg*BD2 residues further widen the binding pocket and reduce the number of hydrophobic contacts with I‐BET151 (Figure 1F; Figure S5C,D, Supporting Information). Consistent with this observation, the Brd4 BD1 pocket buries more of the ligand's solvent‐accessible surface area (SASA) compared to *Cg*BD2 (55.4% vs. 47.2%; Figure S6A, Supporting Information).

Structural modeling predicts that *Cg*BD1 would also engage I‐BET151 less intimately than Brd4 BD1, since *Cg*BD1 residues Arg150 and Gln154 point away from the ligand, unlike the corresponding inward‐pointing Trp81 and Gln85 residues of Brd4 (Figure S6A, Supporting Information). Analogous analyses predict that JQ1, bromosporine and PFI‐1 similarly would exhibit a loose fit within the binding pockets of *Cg*BD1 and *Cg*BD2, on average burying ≈200 Å^2^ less SASA and making fewer contacts than in the corresponding human structures (Figure S6B–D, Supporting Information). The looser fit of these BETi compounds within the more spacious *Cg*BD1 and *Cg*BD2 binding pockets explains their reduced potency relative to human BET BDs. These findings suggest the feasibility of discovering inhibitors with the inverse selectivity (more potent on *Cg*BDs than on human BDs).

### 2.2 Chemical Screening Identifies *Cg*BD1‐Selective Compounds and Non‐Selective Dual *Cg*BD Inhibitors

**Figure 2:**
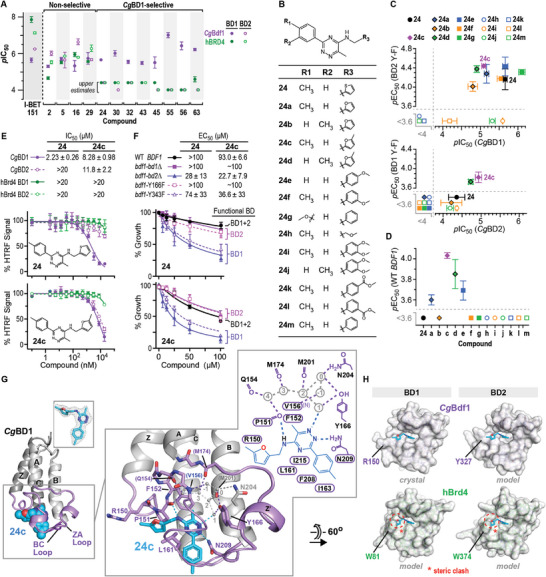
Identification of *Cg*Bdf1‐selective dual BD inhibitor **24c**. A) Summary of HTRF results for the 12 most potent hits. B) Analogs of **24**. C) Inhibitory activity of analogs of **24**. Inhibition (*p*IC_50_) observed in HTRF assays with *Cg*BD1 or *Cg*BD2 is plotted against growth inhibition (*p*EC_50_) observed with *C. glabrata* strains expressing Bdf1 mutated in the other BD. D) Growth inhibition observed against WT *C. glabrata*. E) HTRF inhibition assays performed on **24** and **24c** against the BDs from *Cg*Bdf1 and human Brd4. F) Growth inhibition assays performed on **24** and **24c** with *C. glabrata* strains expressing WT and mutant Bdf1. Functionally active BDs are indicated for each strain. Data represent the mean and s.d. values from three independent experiments (panels A, C and E) or biological replicates (panels D and F). G) Crystal structure of *Cg*BD1 bound to **24c**. *Small inset*. Simulated annealing omit *F*_o_‐*F*_c_ density for **24c** contoured at 3 σ. *Large inset*. Details of ligand binding. Hydrogen bonds are shown as dashed lines. Water molecules are in grey and numbered as in ref. [25]. Labels within a cartouche indicate residues in van der Waals contact with **24c**. H) Surface representations of the *Cg*BD1/**24c** crystal structure and structural alignment models of *Cg*BD2 and of Brd4 BD1 and BD2 showing observed or predicted interactions with the binding pocket.

We adapted our HTRF assay to perform a high‐throughput (HT) screen of small molecule inhibitors selective for *Cg*Bdf1 BDs over human BET BDs. Screening a library of ≈100 000 chemically diverse compounds against *Cg*BD1 yielded ≈250 confirmed hits, of which 63 had IC_50_ values in the low micromolar range and were commercially available (Figure S7, Supporting Information). We repurchased and rescreened these compounds (Table S3, Supporting Information) and subsequently tested the 12 most potent hits against *Cg*BD1 and *Cg*BD2 and the corresponding human Brd4 BDs (Table S4, Supporting Information). This revealed 4 non‐selective inhibitors active against all four BDs and 8 compounds selective for *Cg*BD1 (**Figure** **2**A).

To better understand the different selectivities of these compounds, we determined BD co‐crystal structures for the non‐selective tetrahydropyridoindole **29** and the *Cg*BD1‐selective methylpyrazole **63** (Figure S8A,B and Table S1, Supporting Information). The structure of **29** bound to *Cg*BD2 shows that the compound's acetyl group forms direct and water‐mediated H bonds with Asn386 and Tyr343 in the BC and ZA loops, respectively, while the morpholinylphenyl moiety packs against Tyr327 in its *gauche^−^* conformation (Figure S8C,D, Supporting Information). Structural alignments reveal that the binding pockets of *Cg*BD1 and of Brd4 BD1 and BD2 can all accommodate **29** (Figure S8E, Supporting Information), explaining its poor selectivity. The structure of *Cg*BD1 bound to **63** shows that the latter's pyrazole 2‐nitrogen forms direct and water‐mediated H bonds with Asn209 and Tyr166, respectively, while its carboxyamide nitrogen forms a H bond with the Pro151 carbonyl (Figure S8F,G, Supporting Information). The oxadiazole and two thiophen moieties pack against residue Met218 within a shallow groove defined by residues Lys147 and RPF‐shelf residue Arg150 on one side and Val214 and Ile215 on the other. Structural alignments predict a severe clash between these moieties and the (W/Y)PF‐shelf residue corresponding to Arg150 in the two Brd4 BDs and in *Cg*BD2 (Figure S8H, Supporting Information). These observations highlight the initial residue of the (W/Y/R)PF‐shelf as an important ligand selectivity determinant.

### 2.3 Hit Optimization Identifies a *Cg*Bdf1‐Selective Dual BD Inhibitor

We next tested the 12 above‐described inhibitors for their ability to inhibit *C. glabrata* growth in liquid media. Compounds were tested against strains expressing wildtype (WT) Bdf1 or a mutant form lacking either BD1 or BD2. Because the inactivation of both BDs is required to reduce viability (Figure 1B), dual *Cg*BD inhibitors that successfully target Bdf1 within the fungal cell should inhibit the growth of all three strains, whereas *Cg*BD1‐selective compounds should only inhibit the strain expressing Bdf1‐bd2Δ. Eleven compounds displayed no significant activity, suggesting a lack of on‐target efficacy within the fungal cell (Figure S9A, Supporting Information). In contrast, the phenyltriazine **24**, a *Cg*BD1‐selective compound, inhibited the strain expressing Bdf1‐Δbd2 but not that expressing Bdf1‐Δbd1 or the WT protein, consistent with an on‐target effect.

We obtained or synthesized analogs of **24** and tested these in HTRF and fungal growth inhibition assays (Figure 2B–D). Shifting the methyl group from the *para* to the *meta* phenyl ring position caused an ≈2‐fold drop in potency against *Cg*BD1 and a loss of growth inhibition of the Bdf1‐Δbd1 expressing strain (Figure 2C, upper panel, compounds **24a**, **c**, **i** versus **24b**, **d**, **j**, respectively). Although replacing the thiophenyl by a furyl (**24a**) or methylfuryl (**24c**) group caused a 3‐ or 4‐fold drop in *Cg*BD1 inhibition, respectively, it resulted in slightly enhanced growth inhibition of the *bdf1‐Δbd1* mutant, possibly because of improved solubility. In contrast, replacement by a phenyl (**24** **m**) or methylcarboxyphenyl (**24k** and **l**) group severely compromised all inhibitory activity, whereas replacement by a methoxy‐ or dimethoxyphenyl (**24e, f**, **i**, **j**) did not affect the IC_50_ for *Cg*BD1, although the latter replacement abolished growth inhibition. None of the analogs significantly enhanced the potency against human Brd4 BD1 or BD2. Surprisingly, unlike **24** and the other analogs, methylfuryl‐containing compounds **24c** and **d** significantly inhibited *Cg*BD2 in the HTRF assay and inhibited the growth of *C. glabrata* strains expressing WT Bdf1 and the Bdf1‐Δbd1 mutant (Figure 2C,D). Of the two compounds, **24c** exhibited the more potent BD inhibition (IC_50_ values of 8 and 12 µm against *Cg*BD1 and *Cg*BD2, respectively) and antifungal activity against the WT strain (EC_50_ value of 90 µM) (Figure 2E,F), while displaying only modest cytotoxicity toward HeLa and IMR90 (primary fibroblast) cells at the highest concentration tested (300 µM) in an MTT colorimetric assay (Figure S9B, Supporting Information).

The crystal structure of *Cg*BD1 bound to **24c** (Table S1, Supporting Information) revealed that the methyltriazine moiety forms direct and water‐mediated H bonds with BC loop residue Asn209 and ZA loop residue Tyr166, respectively (Figure 2G). The *p‐*methylphenyl moiety is recognized by hydrophobic residues Leu161, Ile163, Phe208 and Ile215, including a close contact between the *p‐*methyl group of **24c** and the Ile163 methyl group. Shifting the compound's methyl group to a *meta* position would abolish this contact and (in one of the two *meta* positions) yield a steric clash with Phe208, explaining the loss of potency for compounds with this configuration. The methlyfuryl ring packs against Pro151 in the RPF shelf, while the methyl group makes a van der Waals contact with Arg150. Considerable space surrounds this moiety in the binding pocket, explaining why its replacement by certain bulkier substituents is well tolerated.

An alignment with the IBET151‐bound structure of *Cg*BD2 suggests that the *Cg*BD2 binding pocket can accommodate **24c** and mediate nearly identical interactions as those mediated by *Cg*BD1 (Figure 2H). Superimposing the structures of **24** and **24a** onto that of **24c** reveals that the thiophenyl, furyl and methylfuryl groups of these compounds would all clash with the Tyr327 *gauche‐* conformation but that only the methylfuryl group would form a favorable contact with the *trans* conformation, explaining why compounds **24c** and **24d**, but not the other analogs, inhibit *Cg*BD2. The selectivity of **24c** for *Cg*BDs over human Brd4 BDs is attributable to differences in the (W/Y/R)PF shelf as described above for **63**. Namely, whereas *Cg*BD residues Arg150 and Tyr327 point away from the binding pocket, the corresponding Brd4 Trp81 and Typ374 side chains point into the pocket and would clash with the compound's methylfuryl group (Figure 2H).

In summary, the identification of **24c** demonstrates the feasibility of developing inhibitors that target both *Cg*BDs with selectivity over the human BET BDs. The small size (294 Da) of **24c** compared to typical BET inhibitors (usually 350–500 Da) suggest the potential for significant enhancement of potency through chemical optimization. Notably, **24c** weakly inhibited the Bdf1 BDs from *C. albicans* and *C. auris* (Figure S9C, Supporting Information), raising the prospect of developing phenyltriazine‐based inhibitors targeting multiple *Candida* species.

### 2.4 Humanized *Candida* Strains Reveal On‐Target Activity of 24c and Potent Inhibition by I‐BET726

**Figure 3:**
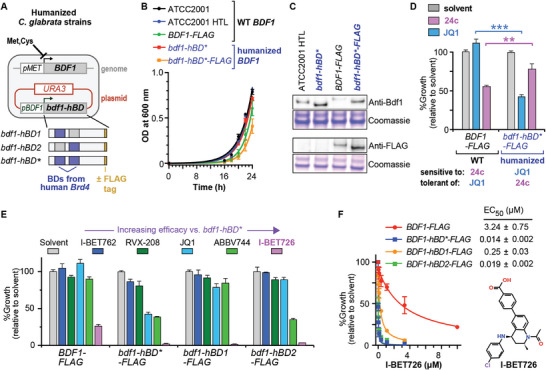
Humanized *Candida* strains reveal on‐target activity of **24c** and potent inhibition by I‐BET726. A) *Cg*Bdf1 containing BD1 and BD2 from human Brd4 was expressed from a plasmid while endogenous Bdf1 expression was repressed by including methionine and cysteine in the growth medium. Strains were also constructed in which humanized Bdf1 is expressed from a modified version of the endogenous *BDF1* gene. B) Growth curves of *C. glabrata* expressing WT or humanized Bdf1. Data in panels B, D and E represent the mean and s.d. values from three biological replicates. C) Western blots showing the expression of WT and humanized Bdf1. Coomassie staining shows equivalent protein loading. D) Growth assays showing differential sensitivity of *C. glabrata* expressing WT or humanized Bdf1 to **24c** and JQ1 (both at 50 µM). ** *p* ≤ 0.01; *** *p* ≤ 0.001 (Holm‐Sidak method). E) Effect of BET inhibitors (50 µM) on the growth of *C. glabrata* expressing WT or humanized Bdf1. F) I‐BET726 inhibits the growth of WT and humanized *C. glabrata*, with different degrees of BD humanization (BD1 only, BD2 only or both BDs [hBD*]). Data represent the mean and s.d. values from three independent experiments. Data are shown for strains expressing humanized Bdf1 from a plasmid (panels A and D) or from a modified version of the genomic *BDF1* ORF (panels B, C, E and F).

We next sought to verify that **24c** inhibits *C. glabrata* growth by engaging Bdf1 BDs in the cell rather than through an off‐target effect. To this end we generated *C. glabrata* strains expressing “humanized” variants of Bdf1 (either untagged or C‐terminally FLAG‐tagged to facilitate immunodetection), in which one or both Bdf1 BDs were replaced by the corresponding human Brd4 BDs (**Figure** **3**A). Growth assays showed that the double BD replacement did not compromise viability and that the humanized strains grew with similar kinetics to the corresponding WT strain (Figure 3B), indicating that the human BDs could functionally replace their fungal counterparts. An immunoblot of cell extracts revealed that humanized Bdf1 was more highly expressed than the WT protein, presumably to compensate for a partial loss in functional efficacy (Figure 3C).

We then examined the effect of the BET inhibitor JQ1 and **24c** on the growth of the WT and humanized strains. Notably, JQ1 more potently inhibited the humanized strain than the WT strain, whereas the converse was true for **24c**, mirroring the efficacy of these inhibitors toward human Brd4 and *Cg*Bdf1 BDs in vitro (Figure 3D). The results show that JQ1 and **24c** can interact with their intracellular BD targets long enough to compromise viability before being extruded or metabolically inactivated by the fungal cell. These findings strongly confirm that the antifungal activity of **24c** is an on‐target Bdf1 BD inhibitory effect.

Because JQ1 and other BETi compounds inhibit both Brd4 BDs in vitro with IC_50_ values in the low‐ to mid‐nanomolar range (Figure S4A, Supporting Information), their efficacy in inhibiting the growth of the humanized Bdf1 strain provides insights into the potential antifungal potency achievable with a dual Bdf1 BD inhibitor optimized to have similar IC_50_ values. We therefore tested the ability of several BETi compounds, including pan‐BET inhibitors JQ1, I‐BET726 and I‐BET762 and BD2‐selective molecules RVX‐208 and ABBV‐744, to inhibit the growth of WT and humanized strains. These compounds displayed varying efficacy against the humanized strains, likely reflecting differences in fungal cell permeability, efflux rate, or metabolism (Figure 3E).

To assess the effect of efflux rate, we tested compounds against an efflux mutant strain of *C. glabrata* harboring a deletion of the *PDR1* gene.^[^ ^26^ ^]^ *PDR1* encodes a transcription factor that regulates the expression of efflux pump proteins, including the ABC transporter protein Cdr1, a central contributor to azole resistance.^[^ ^27^ ^]^ As expected, sensitivity to fluconazole is enhanced upon deletion of *PDR1* and restored to the WT level when Pdr1 expression is rescued (Figure S10A, Supporting Information). Testing the different BET inhibitors as well as **24c** against the *pdr1Δ* and rescue strains revealed a Pdr1‐dependent sensitivity specific to JQ1 (Figure S10B, Supporting Information). This suggests that JQ1 undergoes significant efflux, likely accounting for the relatively weak activity of JQ1 against the humanized *C. glabrata* strains (Figure 3D,E) despite its potent inhibition of human BET bromodomains (Figure S4A, Supporting Information). In contrast, the other tested compounds did not exhibit Pdr1‐dependent activity, indicating that they are not strongly subject to efflux.

Notably, among the latter group, the tetrahydroquinoline‐based compound I‐BET726 showed exceptional activity, markedly diminishing the growth of the WT strain and completely abolishing growth when either Bdf1 BD1 or BD2 was replaced by the corresponding Brd4 BD (Figure 3E). Dose‐response assays revealed EC_50_ values for I‐BET726 of 3 µm and 14 nm against strains expressing WT or humanized Bdf1, respectively (Figure 3F). Thus, I‐BET726 appears especially efficient at penetrating the yeast cell and associating with its BD target. These findings strongly support the feasibility of developing a dual Bdf1 BD inhibitor with high antifungal potency.

### 2.5 NanoBiT Assays Confirm the On‐Target Activity of 24c and I‐BET726

**Figure 4:**
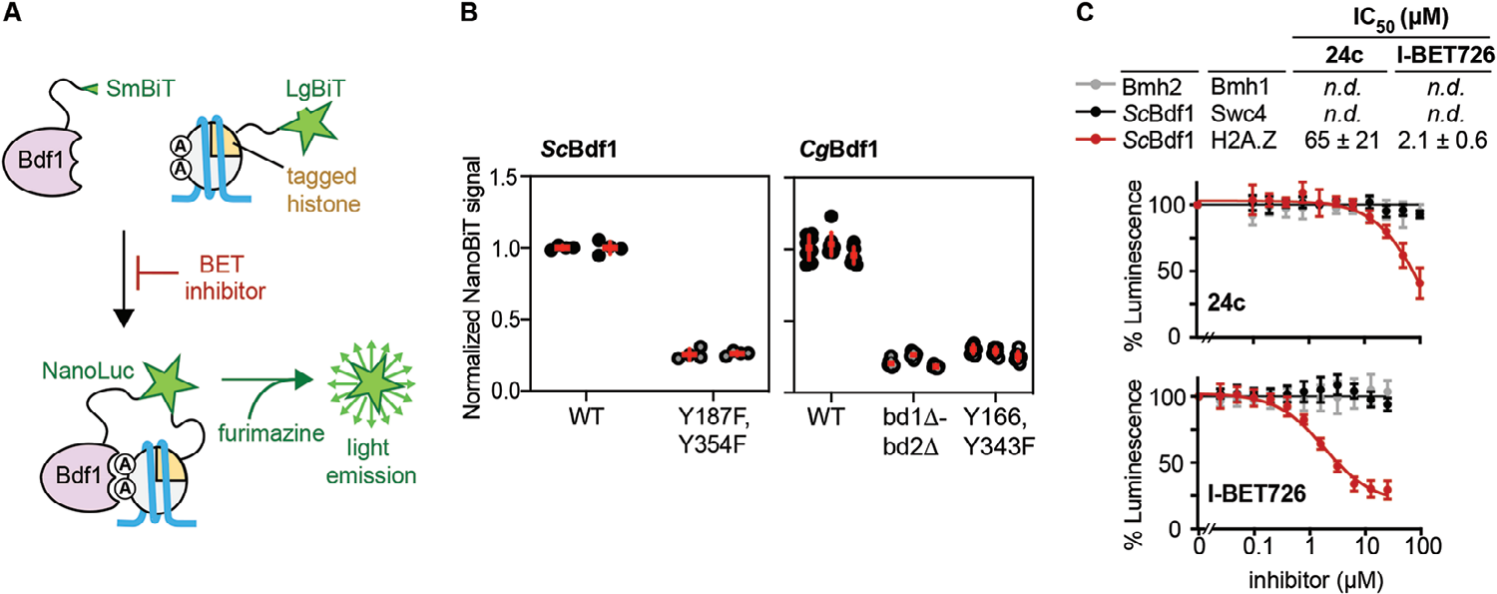
NanoBiT assays confirm the on‐target activity of **24c** and I‐BET726. A) NanoBiT assay. Two fragments of NanoLuc luciferase, SmBiT and LgBiT, are genetically fused to Bdf1 and a histone, respectively. Bdf1 recruitment to chromatin reassembles NanoLuc, which emits light in the presence of furimazine. Acetylated lysines are indicated by an encircled “A”. B) The NanoBiT signal requires functional Bdf1 BDs. Bdf1‐SmBiT was overexpressed from a plasmid, while H2A.Z was endogenously tagged with LgBiT. When expressing *S. cerevisiae* Bdf1 (*Sc*Bdf1, left) or *Cg*Bdf1 (right) in *S. cerevisiae*, the NanoBiT signal is greatly diminished upon mutation of Bdf1 BDs. Results are those of 2–3 biological replicates (independent yeast isolates), each comprising 4–8 technical replicates. C) *In cellulo* validation of on‐target effect. Compounds **24c** and iBET726 inhibit the NanoBiT signal in a dose‐dependent manner, which is specific for the Bdf1‐SmBiT/H2A.Z‐LgBiT pair. Bdf1/Swc4 and Bmh1/Bmh2 are known interactants whose NanoBiT signal is independent of Bdf1 BDs. Data represent the mean and s.d. values from 4 independent experiments.

We next pursued additional verification of on‐target inhibition by **24c** and I‐BET726 by developing an orthogonal assay to probe the histone binding activity of Bdf1 in yeast cells. We chose the NanoBiT bioluminescence assay, an improved protein‐fragment complementation assay that can efficiently be used to probe protein‐protein interactions in live yeast.^[^ ^28^ ^]^ The development of the assay for Bdf1 necessitated the use of strain collections available for *S. cerevisiae*, which we exploited as a surrogate for *C. glabrata* given its phylogenetic proximity relative to the more distant *C. albicans*.^[^ ^29^ ^]^ The NanoBiT assay uses a luciferase (NanoLuc) that is split into a large (LgBiT, 18 kDa) and a small (SmBiT, 11‐residue peptide) fragment that are separately fused to the two interacting proteins of interest (**Figure** **4**A). Upon interaction, the fused moieties can reconstitute an active luciferase that emits light upon addition of the substrate fumirazine. Because this assay is sensitive to the interaction geometry of the fused proteins, we screened different histones for the optimal pairing with Bdf1. We took advantage of a genome‐wide collection of *S. cerevisiae* strains fused to LgBiT and SmBiT^[^ ^28^, ^30^ ^]^ to screen Bdf1 in combination with multiple yeast histone genes. Since the addition of the C‐terminal SmBiT tag had a milder effect on Bdf1 expression compared to LgBiT (Figure S11A, Supporting Information), we screened Bdf1‐SmBiT against LgBiT‐tagged histones. The strongest NanoBiT signal was observed with H2A variant H2A.Z (Figure S11B, Supporting Information), consistent with the latter's genomic colocalization with Bdf1 in vivo.^[^ ^31^ ^]^

To validate the specificity of the NanoBiT signal, WT and mutated Bdf1‐SmBiT were then subcloned in an autonomous plasmid under the control of its endogenous promoter and ectopically expressed in the H2A.Z‐LgBiT strain. Using the *BDF1* sequence from either *S. cerevisiae* or *C. glabrata*, we observed a dramatic decrease in NanoBiT signal when both Bdf1 BDs were inactivated by point or deletion mutations, confirming specificity of the signal (Figure 4B). We then tested the effect of **24c** and I‐BET726. Both compounds strongly decreased the NanoBiT signal for the Bdf1/H2A.Z interaction, yielding IC_50_ values of 65 and 2.1 µM, respectively (Figure 4C), comparable to the EC_50_ values observed in *C. glabrata* growth inhibition assays (93 and 3.2 µM; Figures 2F and 3F). Inhibition was specific to this protein pair and not observed with other Bdf1 interactants (Swc4) or an unrelated NanoBiT pair (Bmh1/Bmh2). These results strongly support Bdf1 BDs as the *in cellulo* target of both **24c** and I‐BET726.

### 2.6 I‐BET726 is Active Against diverse *Candida* Species and Antifungal‐Resistant Strains

**Figure 5:**
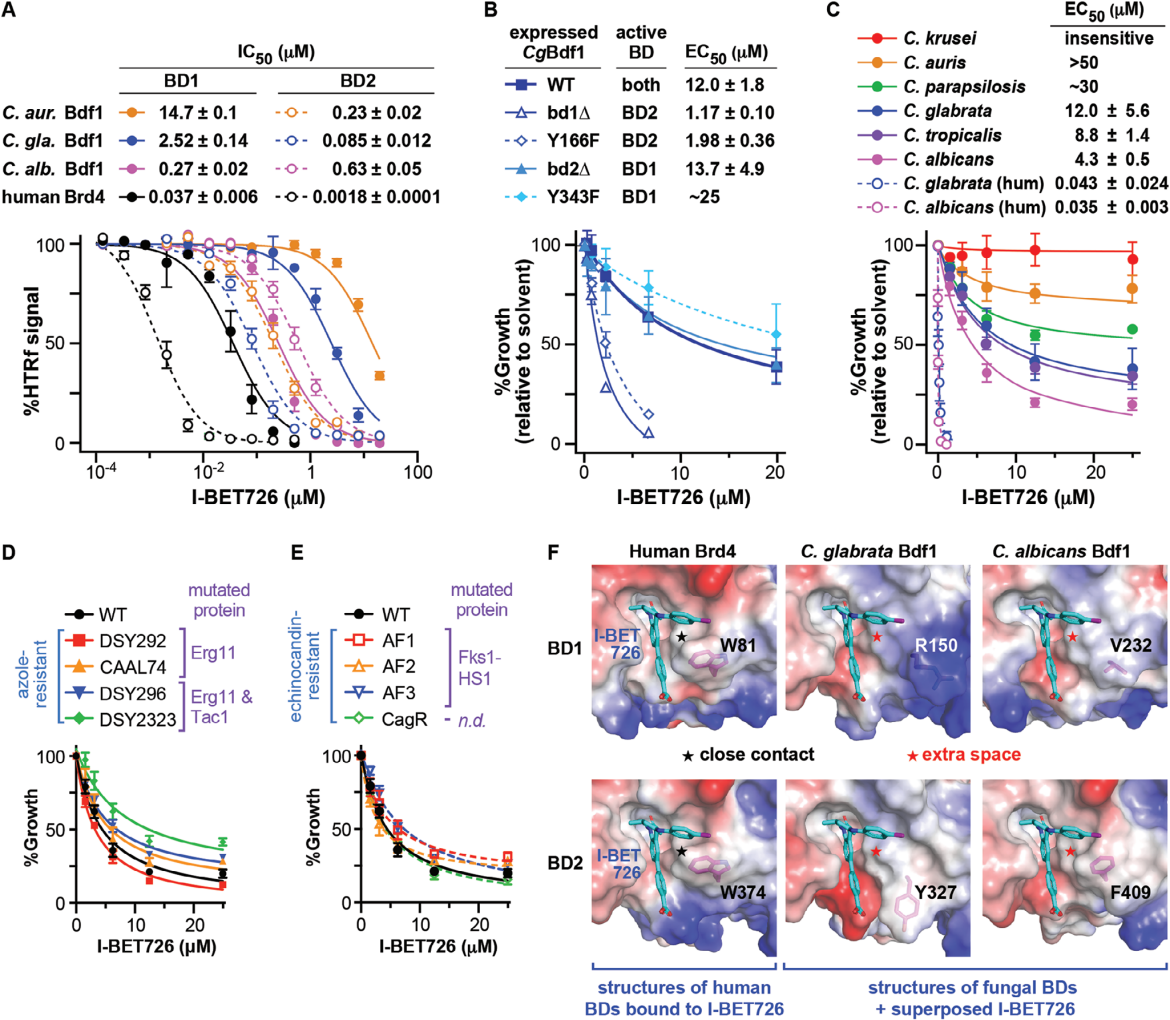
I‐BET726 is active against diverse *Candida* species. A) I‐BET726 inhibits Bdf1 BDs from phylogenetically diverse (*C. glabrata*, *C. albicans* and *C. auris*) *Candida* species. B) *C. glabrata* growth inhibition assays. I‐BET726 shows higher efficacy against BD1‐ than against BD2‐inactivated strains, reflecting its higher potency against BD2 in vitro. C) I‐BET726 inhibits the growth of several *Candida* species. Enhanced inhibition is observed for humanized (hum) *C. glabrata* and *C. albicans* strains (both BDs were humanized). D,E) I‐BET726 inhibits the growth of clinical *C. albicans* strains resistant to D) azoles and E) echinocandins. F) A modified analog of I‐BET726 that clashes with the conserved human Trp in the WPF shelf could potentially confer selectivity for *Candida* Bdf1 BDs. Data in panels A–E represent the mean and s.d values from A) four replicates (two independent experiments each with technical duplicates), B,C) three independent experiments and D,E) three biological replicates.

Because I‐BET726 efficiently inhibited *C. glabrata* growth (Figure 3E,F), we further characterized this compound to evaluate its activity against other *Candida* species. In HTRF assays I‐BET726 displayed submicromolar activity on four of the six Bdf1 BDs from *C. glabrata, C. albicans* and *C. auris* (all three BD2 domains plus *Ca*Bdf1 BD1) and low micromolar activity on the other two (**Figure** **5**A). Growth inhibition assays with *C. glabrata* strains expressing Bdf1 mutants showed that inhibition mirrored the drug sensitivity of the functionally active BD: strains expressing Bdf1 mutants in BD1 but with a functionally active BD2 were more susceptible than strains expressing the converse mutants (compare Figure 5B with blue curves in Figure 5A). In growth inhibition assays I‐BET726 showed efficacy against diverse *Candida* species, especially *C. albicans*, *C. glabrata* and *C. tropicalis* (EC_50_ values in the low micromolar range), whereas the effect against *C. auris and C. parapsilosis* was more modest and *C. krusei* appeared insensitive (Figure 5C). For the three species for which HTRF data were available, growth inhibition mirrored the potency of I‐BET726 against the less sensitive of the two BDs, with EC_50_ and IC_50_ values related by a factor of ≈5–7 (compare orange, blue and pink curves in Figure 5A,C). We developed humanized *C. albicans* strains in which both *BDF1* alleles were modified to replace BD1 and BD2 by the corresponding human Brd4 BDs. As in *C. glabrata*, humanizing CaBdf1 BDs dramatically enhanced the growth inhibitory effect of I‐BET726 (Figure 5C).

We next tested I‐BET726 against antifungal‐resistant *C. albicans* clinical isolates, including four azole‐ and four echinocandin‐resistant strains (Figure 5D,E and Table S5, Supporting Information). Two of the former group bear mutations in *ERG11*, which encodes the enzyme targeted by azoles, while the other two are additionally mutated in *TAC1*, which encodes a transcription factor regulating efflux pump expression.^[^ ^32^ ^]^ Three of the echinocandin‐resistant strains bear mutations in *FKS1*, which encodes the echinocandin target, while the fourth is not genetically characterized. Notably, I‐BET726 inhibited the growth of all eight strains, with EC_50_ values (5–10 µM) comparable to that for the reference WT strain. Similarly, two azole‐resistant *C. glabrata* clinical isolates^[^ ^32^, ^33^ ^]^ also exhibited susceptibility to I‐BET726 comparable to that of the WT strain (Figure S12 and Table S5, Supporting Information). These observations suggest that BET inhibition could help address resistance issues with current antifungal drugs.

As expected, I‐BET726 inhibits human BET BDs more potently than *Candida* Bdf1 BDs (Figure 5A). Structural analysis suggests how to invert this selectivity. I‐BET726 packs snugly against the WPF‐shelf Trp residue conserved across human BET proteins (Figure 5F). As shown for **24c** (Figure 2H), the corresponding *Cg*Bdf1 residues Arg150 and Tyr327 permit additional space in the binding pocket that may be occupied by *a Cg*BD‐selective inhibitor. A similar space is available in the binding pocket of *Ca*Bdf1 BDs, where residues Val232 and Phe409 replace the WPF‐shelf tryptophan (Figure 5F). These observations suggest the potential development of Bdf1‐selective I‐BET726 analogs by introducing modifications that clash with the human Trp residue while favorably occupying the extra space available in the *Candida* BD binding pockets.

### 2.7 Bdf1 BD Inhibition Ameliorates Disease in an Invertebrate Model of *Candida* Infection

**Figure 6:**
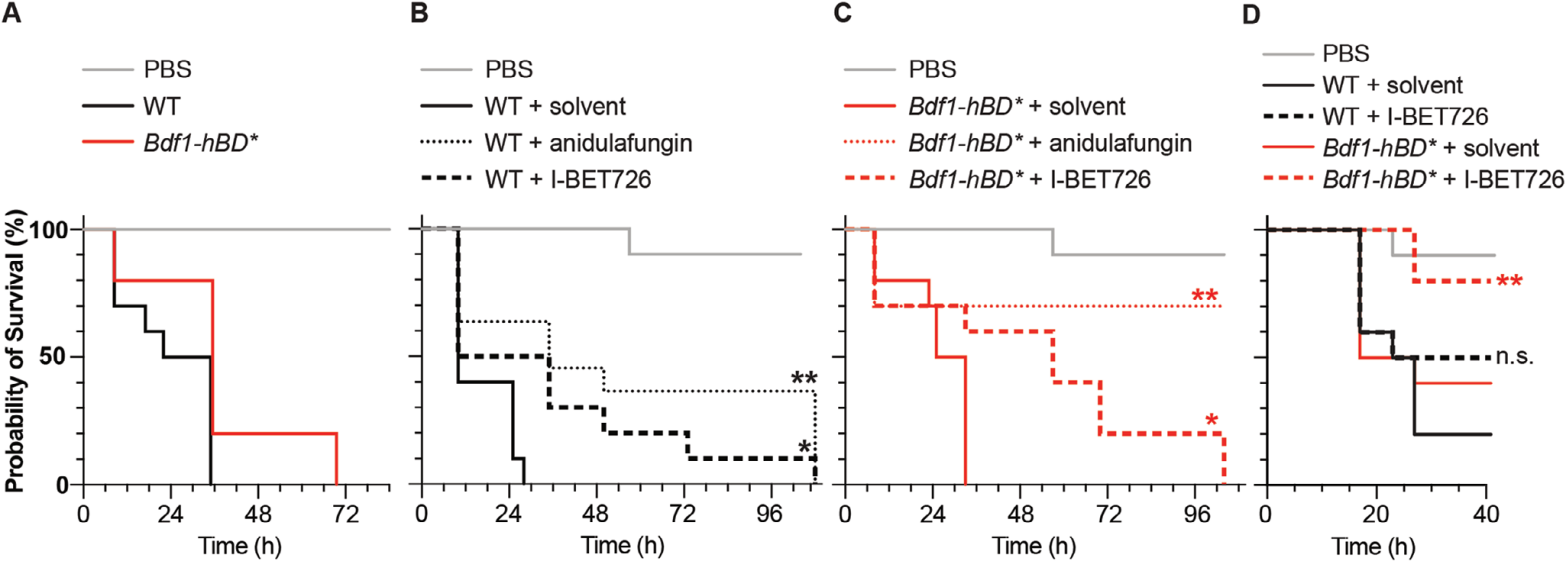
I‐BET726 has antifungal activity in an invertebrate infection model. A) *C. albicans* strains expressing WT or humanized Bdf1 (both BDs) rapidly induce the death of infected *Galleria mellonella* larvae. B,C) I‐BET726 (66 mg kg^−1^) promotes *Galleria* survival when infected with either the B) WT or C) humanized strain. Anidulafungin was used as a control.*p < 0.05, ** *p* < 0.01; Log‐rank (Mantel‐Cox) test. D) A lower dose of I‐BET726 (44 mg kg^−1^) protects against infection with the humanized (*p* = 0.0115; Mantel‐Cox test) but not the WT strain (n.s., not significant). Experiments in panels A‐D were performed at least 3 times independently.

We next explored the antifungal therapeutic potential of Bdf1 BD inhibition in the greater wax moth *Galleria mellonella*, an established model of *Candida* infection.^[^ ^34^ ^]^ *G. mellonella* larvae are convenient for evaluating the activity of antifungal agents because the virulence of fungal pathogens in this model is strongly correlated with that in mice,^[^ ^35^ ^]^ the larval immune system closely resembles the mammalian innate immune response,^[^ ^36^ ^]^ and antifungal efficacy in the larvae correlates well with that in humans.^[^ ^37^ ^]^ Infection of larvae with *C. albicans* strains expressing WT or humanized Bdf1 caused death within ≈36 h (**Figure** **6**A). I‐BET726 treatment of larvae infected with *C. albicans* expressing WT or humanized Bdf1 significantly prolonged survival, up to ≈100 h (Figure 6B,C). For both strains, I‐BET726 was nearly as effective in protecting against disease as anidulafungin, a clinically used echinocandin‐class drug. Use of a lower I‐BET726 dose revealed a differential response between the two strains, with significant protection against infection observed for the humanized but not the WT strain (Figure 6D), reflecting the different sensitivities of the corresponding BDs (Figure 5A). These results highlight the translational potential of BET inhibitors as antifungal therapeutics.

## 3 Conclusion

We previously reported the fungal BET protein Bdf1 as a potential antifungal target in *C. albicans* that required the inhibition of both BDs.^[^ ^20^ ^]^ However, the feasibility of targeting both Bdf1 BDs selectively over human BDs, as well as the antifungal potential of BET inhibition against drug‐resistant strains and other *Candida* species remained unaddressed. Moreover, the lack of molecular tools to verify on‐target antifungal activity hindered progress.

The present study has addressed these critical issues. We validated Bdf1 as a potential antifungal target in *C. glabrata* by demonstrating its essentiality and the lethal effects of dual BD inactivation. We identified the phenyltriazine compound **24c**, that displays low micromolar potency against both *Cg*BDs with selectivity against orthologous human BDs, and validated on‐target antifungal activity through the development of humanized *Candida* and NanoBiT assays. **24c** is the first example of a BET inhibitor that selectively targets fungal BET BDs and exhibits activity against a WT *Candida* strain, establishing a key proof of concept.

Furthermore, our investigations revealed that BET inhibitor I‐BET726 has remarkable potency against humanized *C. glabrata* and *C. albicans* strains, is active against a broad range of unmodified *Candida* species and strains, including azole‐ and echinocandin‐resistant clinical isolates, and displays efficacy in an invertebrate model of systemic *C. albicans* infection. These findings support the feasibility of developing Bdf1 BD inhibitors as antifungal agents with the potential for broad spectrum anti‐*Candida* activity and for combatting antifungal resistance. Notably, JQ1 was recently reported to show efficacy against *Aspergillus fumigatus* in a *Galleria* infection model,^[^ ^38^ ^]^ suggesting BET inhibition as a potential antifungal strategy against highly diverse fungal pathogens, encompassing not only yeasts but also filamentous fungi.

Although **24c** exhibits selectivity for fungal over human BET bromodomains, its inhibitory activity is relatively modest, precluding validation in the *Galleria* infection model. In contrast, while I‐BET726 shows potent antifungal efficacy, it preferentially targets human rather than fungal BET bromodomains, displaying the opposite selectivity required for an effective antifungal agent. Thus, the next challenge will be to identify, through further chemical screening and optimization, a lead compound that combines both the high potency of I‐BET726 and the selective targeting of fungal BET bromodomains demonstrated by **24c**. The results and experimental tools described in the present study provide an important basis to meet this challenge.

In conclusion, our findings underscore the promising potential of Bdf1 BD inhibitors as an innovative class of antifungal therapeutics while highlighting the utility of developing yeast‐based assays to expedite this drug discovery program.

## 4 Experimental Section

### Chemicals

BET inhibitors I‐BET151, bromosporine, PFI‐1 and I‐BET762 were purchased from Sigma. I‐BET726, RVX208 and ABBV744 were purchased from Euromedex while JQ1 was from Clinisciences (Nanterre, France). Screening validation and derived compounds were purchased from ChemDiv as powder and dissolved in dimethylsulfoxide (DMSO) without further purification. Compounds **24k**, **24l** and **24m** were synthesized and compound **24c** was resynthesized in‐house. Reagents for synthesis were purchased from Sigma Aldrich. Reaction mixtures were monitored on a Finnigan LCQ Deca XP Max mass spectrometer equipped with an ESI source, both in negative and positive ion mode. MS *m*/*z* values were calculated using ChemDraw 15.0.0.106. ^1^H, ^13^C, and COSY NMR spectra were taken on a Varian Mercury 400, Varian 500, or Varian 600. Proton chemical shifts (δ) are reported in parts per million (ppm) relative to residual CD_2_HOD in CD_3_OD (δ 3.34, ^1^H NMR), CHCl_3_ in CDCl_3_ (δ 7.26, ^1^H NMR) or HDO in D_2_O (δ 4.80, ^1^H NMR). Carbon chemical shifts (δ) are reported in parts per million (ppm) relative to CD_3_OD (δ 49.00, ^13^C NMR), CDCl_3_ (δ 77.16, ^13^C NMR). NMR spectra processing was performed with MestReNova 14.2.0. High‐resolution mass spectrometry (HRMS) data were acquired on a Thermo LTQ‐Orbitrap XL in positive ion mode over a *m*/*z* range of 100–1000 at a resolution of 60 000 and a target ion population of 5E+5. Synthesized compounds were purified by HPLC or automated flash chromatography (Teledyne CombiFlash Rf+ Lumen Flash Chromatography system).

### Synthesis of Compounds **24c**, **24k**, **24l** and **24m**

**Scheme 1:**
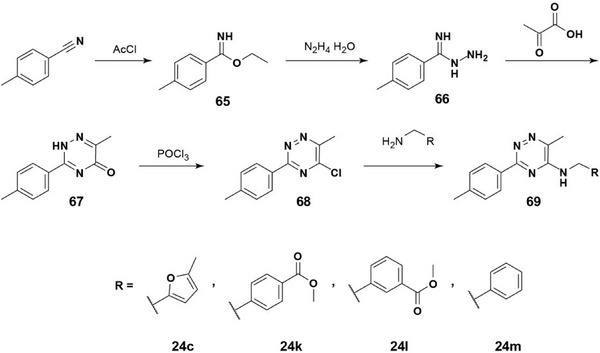
Synthesis of **24c** and **k**‐**m**.

The general synthetic route is outlined in **Scheme** **1**.

*Ethyl 4‐methylbenzimidate (**65**)*: AcCl (8 eq, 387.9 mmol, 30.45 g) was added dropwise to a solution of 4‐methyl‐benzonitrile (1 eq, 48.49 mmol, 5 g) in 34 mL of anhydrous ethanol at 0 °C and the reaction mixture was stirred overnight at rt. When reaction was complete (silica gel TLC, 50:50 EtOAc:hexanes), saturated sodium bicarbonate was added until the solution was slightly basic, when it was extracted with ethyl acetate, and the organic layer washed with brine. The solvent was removed under reduced pressure, leaving **65** as an oil (82%) which was used in the next step without further purification.

*4‐Methylbenzimidohydrazide (**66**)*: Compound **65** was dissolved in ethanol and hydrazine monohydrate (1 eq, 6.703 mmol, 0.36 g) was added to the solution at 0 °C. The reaction mixture was stirred on ice for 2 h. Removal of the solvent under reduced pressure provided **66** as a white crystalline solid.

### 6‐Methyl‐3‐(*p*‐tolyl)‐1,2,4‐triazin‐5(2*H*)‐one (67)

*6‐Methyl‐3‐(p‐tolyl)‐1,2,4‐triazin‐5(2H)‐one (67)*: A solution of **66** (1 eq, 0.643 mmol, 86.9 mg) in isopropanol (1 mL) was slowly added with continuous stirring to a solution of pyruvic acid (1 eq, 0.643 mmol, 56.6 mg) in isopropanol (0.5 mL) at rt and stirring was continued overnight. The desired product **67** was isolated by gradient flash chromatography (DCM:MeOH 0%–20%) (62%).

### 5‐Chloro‐6‐methyl‐3‐(*p*‐tolyl)‐1,2,4‐triazine (68)

Compound **67** (40 mg) was dissolved in phosphoryl chloride (2 mL), and stirred for 1 h at 80 °C. After removal of the solvent under reduced pressure, a residual sticky brown oil was co‐evaporated twice with toluene, giving crude **68** as a dark solid which was used in the next step without further purification.

### 24c, k, l and m

The corresponding amine (1.1 eq) was added to a solution of **68** (1 eq, 70 mg) in dry acetonitrile (4 mL), followed by DIEA (1.3 eq). The mixture was stirred for several hours at rt, until reaction was complete (silica gel TLC, 100% EtOAc). After removal of solvents under reduced pressure, the crude product was purified by flash chromatography (hexanes:acetone).

### 6‐methyl‐*N*‐((5‐methylfuran‐2‐yl)methyl)‐3‐(*p*‐tolyl)‐1,2,4‐triazin‐5‐amine (24c)

^1^H NMR (400 MHz, CD_3_OD) δ 8.40 (d, 2H), 7.31 (d, 2H), 6.25 (s, 1H), 5.91 (s, 1H), 4.79 (s, 2H), 2.52 (d, 3H), 2.42 (d, 3H), 2.26 (d, 3H). ^13^C NMR (101 MHz, CD_3_OD) δ 149.55, 141.10, 128.78, 127.46, 108.17, 105.83, 36.68, 29.31, 20.01, 16.13, 11.96. *m*/*z* calcd for C_17_H_18_N_4_O [M+H]^+^ 295.2, found: 295.1.

### Methyl 4‐(((6‐methyl‐3‐(p‐tolyl)‐1,2,4‐triazin‐5‐yl)amino)methyl)benzoate (24k)

^1^H NMR (400 MHz, CD_3_OD) δ 8.03 – 7.93 (m, 2H), 7.87 – 7.74 (m, 2H), 7.48 – 7.40 (m, 2H), 7.34 – 7.23 (m, 2H), 4.57 (s, 2H), 3.89 (s, 3H), 2.40 (s, 3H), 2.25 (s, 3H). *m*/*z* calcd for C_20_H_20_N_4_O_2_ [M+H_3_O]^+^ 367.2, found: 367.2.

### Methyl 3‐(((6‐methyl‐3‐(p‐tolyl)‐1,2,4‐triazin‐5‐yl)amino)methyl)benzoate (24l)

^1^H NMR (400 MHz, CD_3_OD) δ 8.06 – 7.98 (m, 1H), 7.94 (d, *J* = 7.6 Hz, 1H), 7.79 (d, *J* = 8.2 Hz, 2H), 7.61 (d, *J* = 7.7 Hz, 1H), 7.55 – 7.39 (m, 3H), 4.59 (s, 2H), 3.91 (d, *J* = 0.8 Hz, 3H), 2.48 (s, 3H), 2.34 (s, 3H). *m*/*z* calcd for C_20_H_20_N_4_O_2_ [M+H_3_O]^+^ 367.2, found: 367.2.

### *N*‐benzyl‐6‐methyl‐3‐(p‐tolyl)‐1,2,4‐triazin‐5‐amine (24 m)

^1^H NMR (400 MHz, CD_3_OD) δ 7.80 (dd, *J* = 8.8, 2.5 Hz, 2H), 7.41 – 7.15 (m, 6H), 4.51 (s, 2H), 2.40 (s, 3H), 2.25 (d, *J* = 4.7 Hz, 3H). *m*/*z* calculated for C_18_H_18_N_4_ [M+H_3_O]^+^ calculated: 309.2, found: 309.2.

### Protein Expression and Purification

*Proteins used for crystallization*: DNA fragments encoding BD1 (residues 128–237) or BD2 (residues 304–418) from *Cg*Bdf1 (*Candida* genome database entry Cagl0c02541gp) were PCR amplified from genomic DNA and cloned into a pETM11 vector as fusion constructs bearing an N‐terminal His tag followed by a tobacco etch virus (TEV) protease cleavage site. Transformed *E. coli* strains BL21(DE3) (New England Biolabs, ref. C2527I) were grown in LB medium containing kanamycin (50 µg mL^−1^) at 37 °C until an OD600 of 0.5, induced with 1 mM IPTG and further incubated at 18 °C for 12–20 h before collecting. Cells were resuspended in buffer A (50 mM Tris‐HCl pH 7.5, 300 mM NaCl, 10% glycerol, 25 mM imidazole, 5 mM β‐mercaptoethanol and protease inhibitors) and lysed by sonication. The cleared lysate was incubated with Ni‐NTA resin (Qiagen) and washed with buffer A containing 0.5 M NaCl. Proteins were eluted with 250 mM imidazole, dialyzed overnight in the presence of His‐tagged TEV protease against buffer A (containing no imidazole). After dialysis, Ni‐NTA resin was used to remove His‐tagged species. Proteins were further purified on a Superdex 75 10/300 (GE Healthcare) column in 50 mM Hepes pH 7.5, 150 mM NaCl, 0.5 mM dithiothreitol (DTT). Proteins were concentrated to >20 mg mL^−1^ on a Centricon device (Millipore).

*Proteins used for HTRF assays*: GST‐tagged BD2 (residues 349–460) from human Brd4 (UniProt ID O60885) was purchased from Reaction Biology Corp. Human Brd4 BD1 (residues 22–204), BD1 (residues 193–327) and BD2 (residues 361–501) from *Ca*Bdf1 (Uniprot ID Q5A4W8), BD1 (residues 120–248) and BD2 (residues 289–411) from *Cg*Bdf1 (*Candida* genome database entry Cagl0c02541gp), and BD1 (residues 127–257) and BD2 (residues 287–416) from *C. auris* Bdf1 (UniProt ID A0A0L0NRM7) were cloned into a pGEX4t1 vector as GST‐tagged fusion proteins. Expression in *E. coli* strain BL21(DE3) cells was performed as for His‐tagged constructs. Collected cells resuspended in 50 mM Tris‐HCl pH 7.5, 150 mM NaCl and protease inhibitors were lysed by sonication. The clarified lysate was incubated with glutathione sepharose (GE Healthcare) and then washed with 50 mM Tris‐HCl pH 7.5, 500 mM NaCl and 1% NP‐40. Proteins were eluted with 10 mM glutathione and further purified on a Superdex 200 10/300 (GE Healthcare) column in 50 mM Hepes pH 7.5, 150 mM NaCl, 0.5 mM DTT. Glycerol was added additionally to a final concentration of 30%.

### Pull‐Down Assay

Pull‐down assays were performed as described.^[^ ^1^ ^]^ Biotinylated peptides corresponding to non‐acetylated and tetra‐acetylated (H4K5acK8acK12acK16ac) histone H4 tails were synthesized by Covalab (Villeurbanne, France) and immobilized on Streptavidin‐coated magnetic beads (Dynabeads MyOne Streptavidin C1; Thermo Fisher) according to the manufacturer's instructions. Beads were incubated with 1.25 µg of GST‐tagged *Cg*Bdf1 BD1 or BD2 in binding buffer (50 mM Tris pH 7.4, 150 mM NaCl, 0.1% NP‐40, 10% glycerol, 1 mM DTT) in a volume of 250 µL for 2 h at 4 °C and subsequently washed in binding buffer containing 500 mM NaCl. Bound proteins were eluted by boiling in SDS–PAGE sample loading buffer and analyzed by Western blot using an anti‐GST antibody (GE Healthcare).

### Histone Peptide Array Assay

MODified Histone Peptide Array Kit was purchased from Active Motif (Ref. 13 005). The array was prepared as described in the manufacturer's instructions. 1 µM of proteins in binding buffer (50 mM Tris pH 7.4, 150 mM NaCl, 0.1%, NP‐40, 10% glycerol, 1 mM DTT) were incubated with the array at 4 °C for 2 h. The array was then washed three times with binding buffer. Anti‐GST antibody (GE Healthcare) was used for the detection of GST‐tagged bromodomains. Images were acquired on a ChemiDoc imaging device (Biorad) and treated as recommended by Active Motif.

### HTRF Assay

HTRF reagents and buffers were purchased from Cisbio Bioassays. The assay used a terbium (III) cryptate donor reagent conjugated to an anti‐GST antibody (MAb anti‐GST‐Tb; ref. 61GSTTLA), a streptavidin‐conjugated acceptor reagent (streptavidin‐d2; ref. 610SADLA) and Cisbio proprietary buffers (EPIgeneous Binding Domain Diluent and Detection buffer; refs. 62DLBDDF and 62DB2FDG, respectively). Incubation with GST‐tagged BDs and biotinylated H4ac4 brings the donor and acceptor into close proximity and allows FRET. The non‐biotinylated H4ac4 peptide competes for binding and was used as a positive control of inhibition. GST‐tagged proteins in 25 mM Hepes pH 7.5, 150 mM NaCl, 0.5 mM DTT were assayed at a final concentration of 5 nM. Biotinylated H4ac4 peptides were used at a final concentration of 50, 600, 300, 400, 30, 250, 176 or 1450 nM in assays involving Brd4 BD1 and BD2, *Ca*Bdf1 BD1 and BD2, *Cg*Bdf1 BD1 and BD2, and *C. auris* Bdf1 BD1 and BD2, respectively. The antibody‐conjugated donor was used at 0.5 nM and the streptavidin‐conjugated acceptor was used at 1/8 of the H4ac4 peptide concentration. Inhibitors were tested by performing an 11‐point 2.5‐fold dilution series with a maximal final concentration of 20 mM. These concentrations allowed a fixed DMSO concentration at 0.2%, critical for a Z’ factor ≥ 0.8. Components were incubated at 4 °C for 4 h (all BD1) or for 24 h (all BD2). Experiments were performed in 384‐well white plates (Greiner ref. 781 080) in a volume of 16 µL and analyzed in a ClarioStar plate reader (BMG LABTECH, excitation at 330 nm and emission at 620 and 665 nm, corresponding to the donor and acceptor emission peaks, respectively; the 665/620 ratio is used to calculate the specific HTRF signal) with an integration delay of 60 µs and an integration time of 400 µs. The vehicle used for all inhibitors was DMSO.

### High‐Throughput Chemical Screening

The HTRF assay described above was miniaturized in a 5 µL volume in 1536‐well black plates. Because of better HT assay parameters (signal‐to‐background ratio and Z’‐factor) for *Cg*BD1 than *Cg*BD2, we performed the primary screen against *Cg*BD1. Approximately 100 000 compounds comprising the Soluble Diversity (ChemDiv), Targeted Diversity (ChemDiv) and 30K Diversity (LifeChem) collections were screened. These collections were selected based on their broad chemical diversity and their potential to include molecules with drug‐like properties. The Soluble Diversity library was chosen specifically for its inclusion of small, soluble compounds suitable for in vitro screening, while the Targeted Diversity library was selected for its focus on drug‐like scaffolds known to interact with key protein families, including bromodomains. The LifeChem Diversity Library was chosen due to its extensive coverage of chemical space, providing a large pool of compounds with diverse physicochemical properties. The compounds in these libraries meet criteria that include compliance with Lipinski's Rule of Five, Veber criteria, and the exclusion of reactive molecules through PAINS filters, ensuring a high‐quality, drug‐like screening set. Compounds dissolved in DMSO were dispensed into wells by an Echo acoustic liquid dispenser. A master mix comprising MAb anti‐GST‐Tb donor, streptavidin‐d2 acceptor, GST‐tagged *Cg*BD1 protein, biotinylated H4ac4 peptide was then added, and the plates incubated for 4 h prior to reading. The primary screen was performed with compounds at a final concentration of 20 µM, corresponding to a final DMSO concentration of 1%. Hits were initially confirmed by repeating the assay at a single concentration in triplicate, and subsequently by dose–response curves constructed using 8‐point dilutions between 0 and 20 µM.

### Crystallization and Crystal Structure Determination

Initial crystallization conditions were identified by the sitting drop vapor diffusion method at 4 °C using a Cartesian PixSys 4200 crystallization robot at the High Throughput Crystallization Laboratory of the EMBL Grenoble (https://htxlab.embl.org). Crystals used for data collection were obtained by the hanging drop method at 4 °C by mixing 1 µL of the protein or protein/inhibitor sample with 1 µL of the reservoir solution, as follows. Crystals were harvested directly from the crystallization drop and flash‐cooled in liquid nitrogen unless otherwise specified. Unbound *Cg*Bdf1 BD1 (25 mg mL^−1^) was mixed with 25% (w/v) PEG 1500 and 0.1 MIB (malonic acid:imidazole:boric acid in 2:3:3 molar ratio) buffer (pH 9). Unbound *Cg*Bdf1 BD2 (40 mg mL^−1^) was mixed with 0.1 M CH_3_COONa (pH 4.6) and 33% (v/v) MPD. *Cg*Bdf1 BD1 bound to compound **63** was crystallized by mixing 40 mg/mL protein and 6 mM inhibitor with 26% PEG 10 000, 0.2 M ammonium sulfate and 0.1 M sodium cacodylate (pH 6.5); crystals were harvested in the same crystallization buffer but that additionally contained 25% PEG 400. *Cg*Bdf1 BD1 bound to compound **24c** was crystallized by mixing a solution of 35 mg mL^−1^ protein and 5.4 mM inhibitor with 0.072 M NaH_2_PO_4_ and 1.73 M K_2_HPO_4_ (pH 8.2). *Cg*Bdf1 BD2 bound to I‐BET151 was crystallized by mixing a solution of 15 mg mL^−1^ protein and 2.2 mM inhibitor with 0.9 M (NH_4_)_2_SO_4_ and 0.1 M sodium cacodylate (pH 6.5); crystals were harvested in a cryoprotectant containing 1.1 M (NH_4_)_2_SO_4_, 0.1 M sodium cacodylate (pH 6.5) and 30% (v/v) glycerol. *Cg*Bdf1 BD2 bound to compound **29** was crystallized by mixing 20 mg mL^−1^ protein and 1.5 mM inhibitor with 0.8 M NaH_2_PO_4_, 0.8 M KH_2_PO_4_ and 0.1 M HEPES (pH 7.5).

X‐ray diffraction data collected at beamlines of the ESRF and Soleil were processed using XDS^[^ ^2^ ^]^ and programs of the CCP4 suite.^[^ ^3^ ^]^ Structures were solved by molecular replacement using Phaser,^[^ ^4^ ^]^ improved by manual building using Coot,^[^ ^5^ ^]^ and refined with Phenix.^[^ ^6^ ^]^ Data collection and refinement statistics are summarized in Table S1 (Supporting Information). Structural figures were made using PyMOL.^[^ ^7^ ^]^

### Generation of *C. glabrata* Mutant Strains

Plasmids used in this study are listed in Table S6 (Supporting Information). All DNA fragments were fused in pCR2.1‐TOPO (genome integration cassette) or pGRB2.0 (rescue plasmids) vectors using a Gibson assembly kit (New England Biolabs) and validated by sequencing. The *BDF1* point mutant plasmids were obtained using the QuikChange II Site‐directed mutagenesis kit (Agilent) with the *BDF1* plasmid pJG267. *C. glabrata* was transformed by a classic lithium acetate‐based procedure, as previously described^[^ ^8^ ^]^ with minor changes (incubation at 30 °C for 30 min instead of overnight). Plasmids were cloned in plasmid pCU‐MET3^[^ ^9^ ^]^ (Addgene #45 336). *C. glabrata, C. albicans* and *S. cerevisiae* strains used in this study were listed in Tables S7 and S8 (Supporting Information).

### Growth Assays

Growth assays were performed as described^[^ ^1^ ^]^ with minor changes. *Growth on solid media*. *C. glabrata* strains were grown in SC or SC‐U (SC medium without uracil) media to an OD_600_ of 0.5–0.8, pelleted and resuspended in sterile water at a final OD_600_ of 0.13. Cells were spotted on solid media in a threefold dilution series starting at an OD_600_ of 0.13. Plates were incubated at 30 °C for 1 day before imaging.

*Growth on Liquid Media with C. glabrata Lab Strains*: Cells were grown at 30 °C in SC+M+C‐U medium (SC medium with 5 mM methionine, 0.25 mM cysteine, without uracil) to an OD_600_ of 0.5–0.8. For the evaluation of growth defects related to Bdf1 mutations, log‐phase growing cells were counted using a Neubauer chamber and diluted in liquid media to a final concentration of 13 500 cells mL^−1^ (67 500 cells per mL for *BDF1*‐hBD‐FLAG and *BDF1*‐hBD1‐FLAG pMET strains) per well in a sterile 96‐well plate. Plates were incubated at 30 °C and OD_600_ was measured using a Multiskan FC Microplate Photometer (Thermo Fisher). For the evaluation of chemical compounds, *C. glabrata* pMET strains were grown in SC+M+C‐U medium 24 h before counting and then seeded in 96‐well plates at the same concentration as mentioned above, with or without chemical compounds.

### Growth Assays to Assess Compound Efflux

Cells from a single colony were diluted in 0.85% NaCl before being counted using a Neubauer chamber. They were diluted to a final concentration of 250 000 cells mL^−1^ per well in a sterile 96‐well plate for *C. glabrata* strains (WT, *pdr1∆* or *PDR1* rescue). Compound **24c** and BETi compounds I‐BET762, RVX‐208, JQ1, and ABBV744 were tested at a final concentration of 50 µM, while I‐BET726 was tested at 1 µM concentration to ensure detection of an efflux pump effect. Plates were incubated at 30 °C for 20 h and OD_600_ was measured using a Multiskan FC Microplate Photometer (Thermo Fisher). The effect of fluconazole was tested by titration on these strains under similar conditions.

### Growth of Clinical Strains in Liquid Media

Strains were presented in Table S8 (Supporting Information). Cells from a single colony were diluted in 0.85% NaCl before being counted using a Neubauer chamber. They were diluted to a final concentration of 6500 cells mL^−1^ per well for all clinical strains of *C. albicans* and *C. glabrata*; 65 000 cells mL^−1^ per well for *C. auris*; 13 500 cells mL^−1^ per well for *C. parapsilosis*; 3375 cells mL^−1^ per well for *C. krusei* and 1687.5 cells mL^−1^ per well for *C. tropicalis*, in a sterile 96‐well plate. Cells were then grown in SC medium with or without chemical compounds at 30 °C with regular shaking. The significance of growth defects was assessed using Holm‐Sidak method by GraphPad Prism 10.

### Analysis of Whole‐Cell Extracts and Antibodies

*C. glabrata* strains were grown at 30 °C in liquid media to an OD600 of 0.5–0.8. Cells were lysed in Fastprep (MPBiologicals) twice at 6 ms^−1^ for 30 s with intermediate incubation on ice. FLAG antibody was purchased from Sigma–Aldrich (ref. F3165) and endogenous *Cg*Bdf1 detected with an antibody developed internally against Bdf1 from *S. cerevisiae*.^[^ ^10^ ^]^

### Cytotoxicity Assays on Human Cells

Cytotoxicity assays were performed as described^[^ ^1^ ^]^ with minor changes. Proliferation of human cells was assessed using an MTT colorimetric assay (Cell Proliferation Kit I, Roche). HeLa (epithelial cells, ATCC number CCL‐2) and IMR90 (primary fibroblasts cells, ATCC number CCL‐186) cells were cultured in humidified atmosphere (37 °C and 5% CO_2_) in DMEM medium containing 10% heat inactivated fetal calf serum and 2 mM glutamine. Cells were seeded at a concentration of 5000 (HeLa) or 5600 (IMR90 cells) per well in 100 µL culture medium containing the test compound (**24c**, amphotericin B or fluconazole) into 96 wells microplates (Falcon ref. 353 072). Plates were incubated at 37 °C and 5% CO_2_ for 24 h before adding 10 mL of MTT labelling reagent (final concentration 0.5 mg mL^−1^) to each well. After incubating for a further 4 h, 100 µL of the solubilization solution were added in each well. Plates were allowed to stand overnight in the incubator before measuring the absorbance at 570 nm and at 690 nm in a ClarioStar plate reader. The values of A570–A690 nm were normalized relative to that obtained with vehicle (0.2% DMSO, 0.8% ethanol) and plotted against compound concentration.

### NanoBiT Assays

Yeast strains were distributed in 96‐well microplates containing SC(MSG)‐U medium (SC‐U medium with 0.1% monosodium glutamate instead of ammonium sulfate as a nitrogen source) and grown at 30 °C. 40 µL of exponentially growing cells were transferred in white 96‐well half‐area microplates (ProxiPlate Plus, Greiner Bio‐One) previously filled with 40 µL per well of SC(MSG) containing 100 µM furimazine. The microplates were then incubated for 10 min in the dark before the luminescence signals were recorded using a microplate reader (Ensight, PerkinElmer). Luminescence measurements were performed for 1 s per well at a distance of 0.1 mm between the plate and the detector. The luminescence values were normalized with the optical density (OD_600_) of the corresponding cultures. For NanoBiT assays with BET inhibitors, yeast cultures were grown overnight at 30 °C in SC(MSG) medium in the presence of serial dilutions of **24c** and I‐BET726. Overnight cultures were diluted 5 fold in SC(MSG) and incubated for 1 h before luminescence was recorded as previously indicated. Luminescence values for each condition were normalized using the average of the luminescence recorded for the Bdf1/Swc4 and Bmh1/Bmh2 controls.

### Evaluation of the Effect of Compounds in *Galleria* Fungal Infection Model

*Galleria mellonella* larvae were purchased from Decathlon (La Tronche, France). They were selected in the late larval stages weighing between 300 and 400 mg. Groups of 10 larvae were injected into the right or left last pro‐leg with 10 µl of either PBS, *C. albicans* suspensions or antifungal drug using an insulin syringe (BDUltra‐Fine 0.3 ml). *C. albicans* suspensions of the WT and humanized Bdf1 strains were adjusted to 10^6^ cells larva^−1^. Antifungal drugs (anidulafungin 5 mg kg^−1^ or I‐BET726 66 or 44 mg kg^−1^) were injected once 30 min before fungal infection. An infected control group received the *C. albicans* inoculum and the solvent of the drug. A non‐infected control group received only PBS. After inoculation, larvae were placed in Petri dishes and incubated in the dark at 37 °C. The larvae were monitored for 5 days, and survival outcome was determined. For experiments performed at the lower (44 mg kg^−1^) dose of IBET‐726, the difference in protection against infection with humanized versus WT *C. albicans* strains was more evident using the survival data at 40 h rather than at 100 h post‐infection. Larvae were considered dead when no response was observed following touch. All experiments were performed at least 3 times independently. The significance of survival was assessed by a Log‐rank (Mantel‐Cox) test in GraphPad Prism 10.

### Statistical Analysis

All experiments were repeated at least three times. Data represent biological replicates, each comprising one or more technical replicates, with the number and type of replicates indicated in each figure legend. Results are expressed as the mean ± SD. Statistical significance was assessed using the Holm‐Sidak, Mantel‐Cox or two‐tailed Student's *t*‐test method (as indicated in each Figure legend) with GraphPad Prism 10 software.

## Conflict of Interest

J.G. received speaking fees from Gilead.

## Author Contributions

C.E.M., C.P., and J.G. performed conceptualization and funding acquisition; K.W., M.A., H.D., G.B., D.S., M.C, G.R., C.P., and J.G. performed formal analysis; K.W., M.A., J.M.O., T.‐A.D., Y.Z., N.J.D., J.Y., H.D., C.G., G.B., F.M., M.C., A.L., Y.H., R.‐L.I., D.S., M.Co., G.R., C.P., and J.G. performed investigation; K.W., M.A., M.N.‐S., M.C., G.R., and J.G. performed methodology; M.C., G.R., C.E.M., C.P., and J.G. performed project administration; M.A., J.M.O., Y.Z., N.J.D., J.Y., G.B., F.M., M.C., A.L., Y.H., M.N.‐S., M.C., G.R., and J.G. provided resources; B.A.K., M.C., G.R., C.E.M., C.P., J.G. performed supervision; M.A., B.A.K., D.S., M.C., G.R., C.E.M., C.P., and J.G. performed validation; K.W., M.A., H.D., Y.H., D.S., M.C., G.R., C.P., and J.G. performed visualization; C.P. wrote the original draft; K.W., J.M.O., J.Y., M.N.‐S., D.S., M.C., G.R., C.E.M., C.P., and J.G. wrote, reviewed and edited.

## Supporting information



Supporting Information

Supporting Information

Supporting Information

Supporting Information

## Data Availability

Crystal structures and diffraction data have been deposited in the Protein Data Bank for *Cg*Bdf1 BD1 in the unbound form (PDB 8R6I) or in complex with **63** (PDB 8R6J) or with **24c** (8R6K) and for *Cg*Bdf1 BD2 in the unbound form (PDB 8R6L) or in complex with I‐BET151 (8R6M) or with **29** (8R6N). Other data that support the findings of this study are available in the supplementary material of this article.
