# Supplementary material for: Humanized Candida and NanoBiT Assays Expedite Discovery of Bdf1 Bromodomain Inhibitors With Antifungal Potential
Source: Adv Sci (Weinh). 2025 Jan 16;12(10):2404260. doi: 10.1002/advs.202404260 (PMC11904993; doi:10.1002/advs.202404260)
Supplement: Supplementary file 1 — Supporting Information [file ADVS-12-2404260-s002.pdf]

ADVANCED SCIENCE Open Access

Supporting Information

for *Adv. Sci.*, DOI 10.1002/advs.202404260

Humanized *Candida* and NanoBiT Assays Expedite Discovery of Bdf1 Bromodomain Inhibitors With Antifungal Potential

Kaiyao Wei, Marie Arlotto, Justin M. Overhulse, Tuan-Anh Dinh, Yingsheng Zhou, Nathan J. Dupper, Jiayi Yang, Boris A. Kashemirov, Hasan Dawi, Cécile Garnaud, Gaëlle Bourgine, Flore Mietton, Morgane Champleboux, Amédé Larabi, Yordan Hayat, Rose-Laure Indorato, Marjolaine Noirclerc-Savoye, Dimitrios Skoufias, Muriel Cornet, Gwenaël Rabut, Charles E. McKenna, Carlo Petosa* and Jérôme Govin**

Supporting Information

Humanized *Candida* and NanoBiT Assays Expedite Discovery of Bdf1 Bromodomain Inhibitors with Antifungal Potential

Kaiyao Wei, Marie Arlotto, Justin M. Overhulse, Tuan-Anh Dinh, Yingsheng Zhou, Nathan J. Dupper, Jiayi Yang, Boris A. Kashemirov, Hasan Dawi, Cécile Garnaud, Gaëlle Bourgine, Flore Mietton, Morgane Champleboux, Amédé Larabi, Yordan Hayat, Rose-Laure Indorato, Marjolaine Noirclerc-Savoye, Dimitrios Skoufias, Muriel Cornet, Gwenaël Rabut, Charles E. McKenna, Carlo Petosa *, Jérôme Govin**

Table of Contents

Figures S1-S11 1
Tables S1-S8 13
List of Supplementary Files 21
Supplementary References 21
LRMS data for compounds 24c, 24k, 24l and 24m 22
NMR spectra for compounds 24c, 24k, 24l and 24m 24
Data availability for commercially purchased compounds 27
Supplier-provided LC/MS data for commercially purchased compounds 28
Supplier-provided NMR spectra for commercially purchased compounds 58

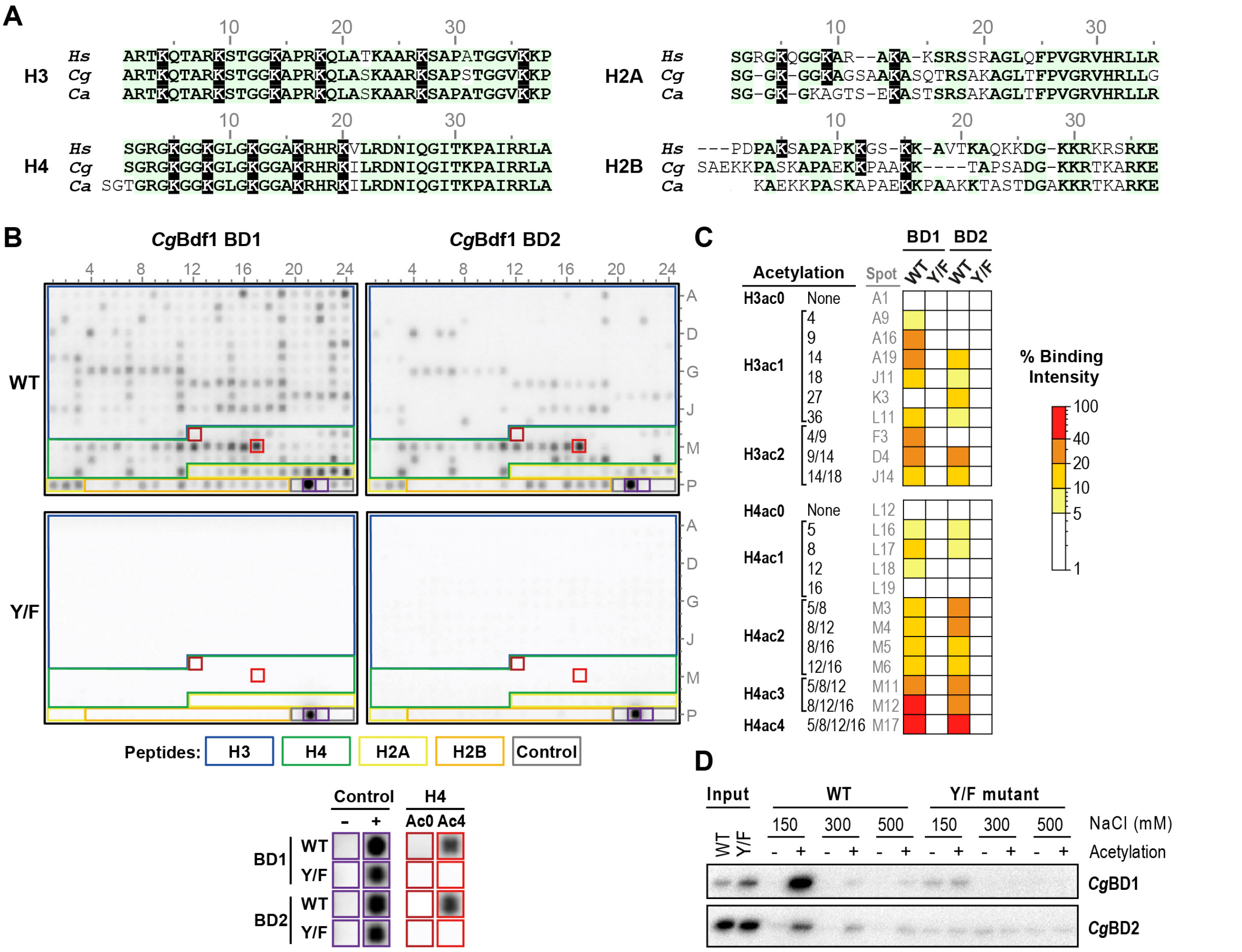

Figure S1. *Cg*Bdf1 BDs recognize multi-acetylated histone peptides.

(**A**) Alignment of N-terminal sequences from human (*Hs*), *C. glabrata* (*Cg*) and *C. albicans* (*Ca*) histones showing the high sequence conservation of the H3 and H4 tails. Lysine positions acetylated in peptides on the array in (B) are shown in inverse font.

(**B**) Binding of WT *Cg*Bdf1 BDs (top) and Y/F mutants (Y166F or Y343F; bottom) to an array of human histone peptides. The array includes control peptides (grey outline) and N-terminal peptides from histones H3, H4, H2A and H2B (blue, green, yellow and orange outlines, respectively), with H3 and H4, which are nearly identical between human and *C. glabrata*, comprising the majority (347 of 384 peptides). Signals for background and positive-binding controls (boxed in red) or for unacetylated (Ac0) and tetra-acetylated (Ac4) H4 peptides (boxed in purple) are shown magnified below the array.

(**C**) Summary of binding intensities of WT and mutant *Cg*Bdf1 BD1 and BD2 to the acetylated H3 and H4 peptides from the array in (B). Binding intensities are normalized to a positive control signal, obtained from the interaction of an anti-c-Myc antibody with a c-Myc control peptide included on the array. The two *Cg*BDs exhibit very similar binding profiles: of the 20 acetylated H3 and H4 peptides on the array, 15 showed either substantial (14 peptides) or poor (1 peptide) binding by both BDs, whereas only 5 were preferentially bound by either BD1 (4 peptides) or BD2 (1 peptide).

(**D**) Pull-down assay. Immobilized H4ac0 and H4ac4 peptides were incubated with GST-tagged *Cg*Bdf1 BDs or with the corresponding Y/F mutants. After washing with buffers containing either 150, 300 or 500 mM NaCl, bound proteins were eluted and visualized by Western blotting with an anti-GST antibody. Whereas the wildtype (WT) BDs showed specific H4ac4 peptide binding, those bearing the Y/F mutation bound weakly and failed to discriminate between the acetylated and non-acetylated H4 peptide.

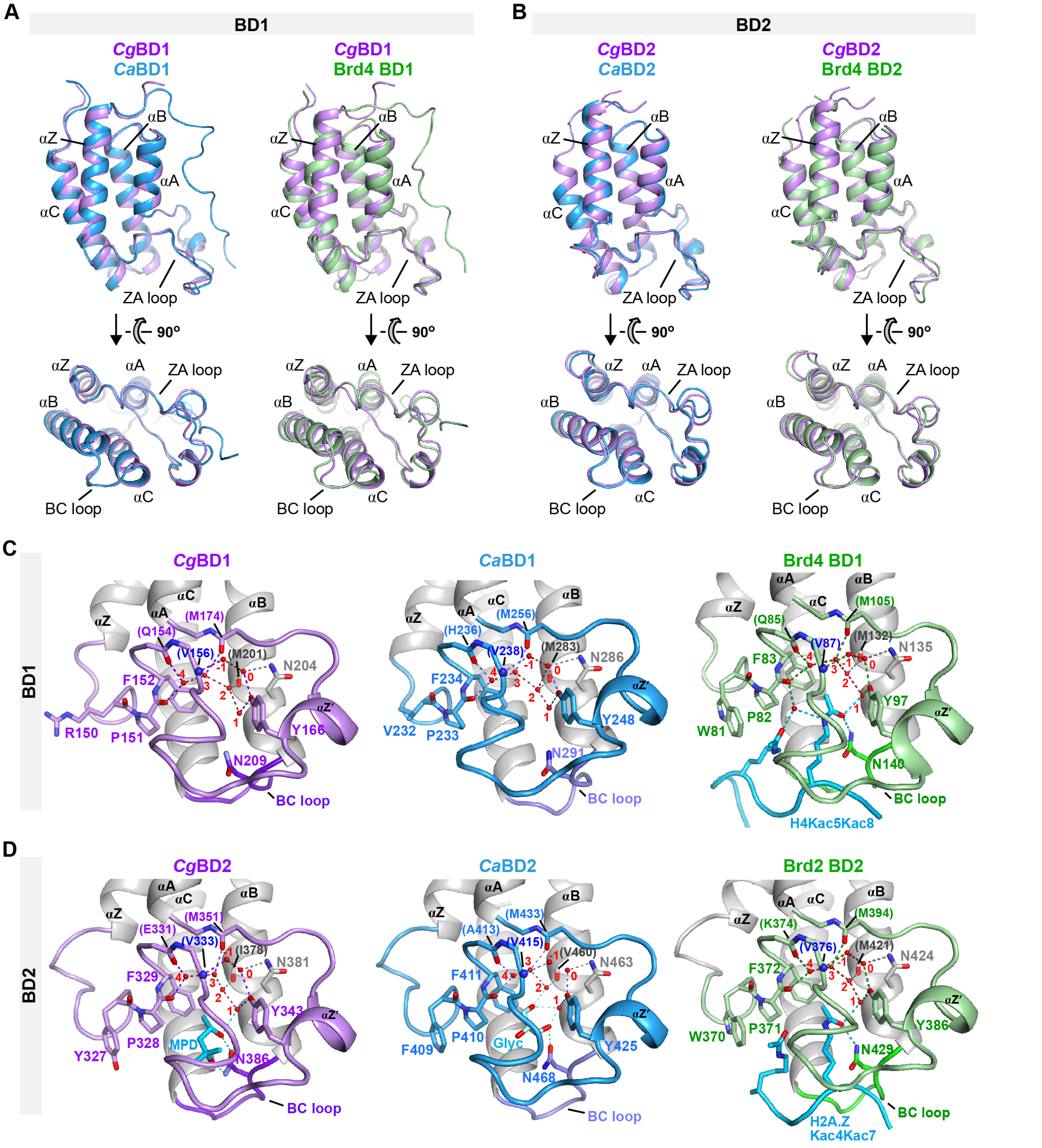

Figure S2. Structure of *Cg*Bdf1 BDs and comparison with *C. albicans* and human orthologs.

(**A**) Alignment of the unbound BD1 structure from *Cg*Bdf1 (violet) with that of *Ca*Bdf1 (blue; PDB 5n15) and human Brd4 (green; PDB 2oss).

(**B**) Alignment of the unbound BD2 structure from *Cg*Bdf1 (violet) with that of *Ca*Bdf1 (blue; PDB 5n18 chain B) and human Brd4 (green; PDB 2ouo).

(**C**) Close-up view of *Cg*BD1 (violet) and *Ca*BD1 (blue, PDB 5n15) compared with that of human Brd4 BD1 bound to a diacetylated H4 peptide (green, PDB 3uvw) showing conserved water structure and hydrogen bonding interactions (dashed lines) in the ligand binding site. Residues interacting through backbone atoms are labelled in parentheses. Water molecules are numbered as in ref. [11].

(**D**) Ligand binding sites of *Cg*BD2 (violet) and *Ca*BD2 (blue) compared with that of human Brd2 BD2 bound to a diacetylated H2A.Z peptide (green, PDB 7jx7). Small molecules present in the crystallization buffers used for *Cg*BD2 (MPD) and *Ca*BD2 (glycerol) occupy the Kac binding pocket.

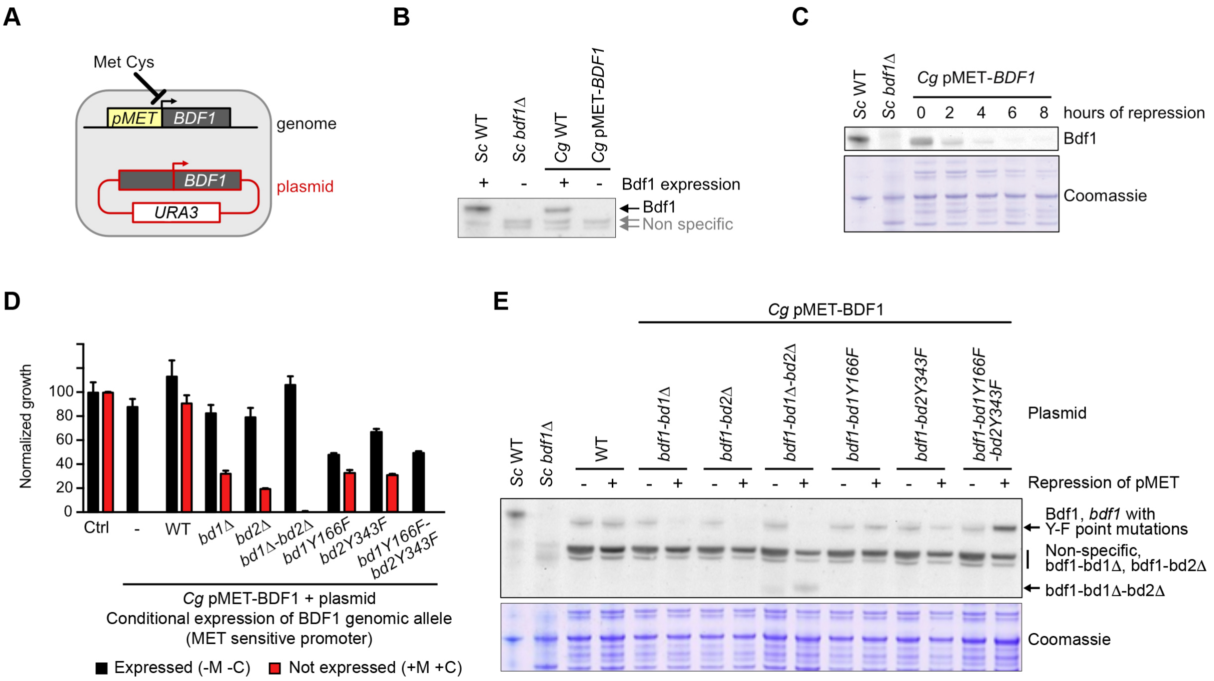

Figure S3. *Cg*Bdf1 bromodomains are essential for yeast viability.

(**A**) Strategy for Bdf1 expression used in this study. The endogenous *BDF1* promoter was replaced by a *MET3* promoter (pMET), which is repressed in the presence of an excess of cysteine and methionine. *BDF1* expression is rescued from an autonomous plasmid.

(**B**) Validation of the Bdf1 antibody [10], which cross-reacts between Bdf1 from *S. cerevisiae* (*Sc*) and *C. glabrata* (*Cg*).

(**C**) Repression of *BDF1* expression following the addition of methionine and cysteine.

(**D**) Growth assays in liquid media showing impact of repression of the genomic copy of *BDF1* on vegetative growth with ectopic expression of WT or mutant *BDF1* from a plasmid. Data represent the mean and s.d. values from three independent replicates.

(**E**) Expression levels of *BDF1* upon repression of its genomic copy. Its expression is rescued from a plasmid (*BDF1* WT or mutant).

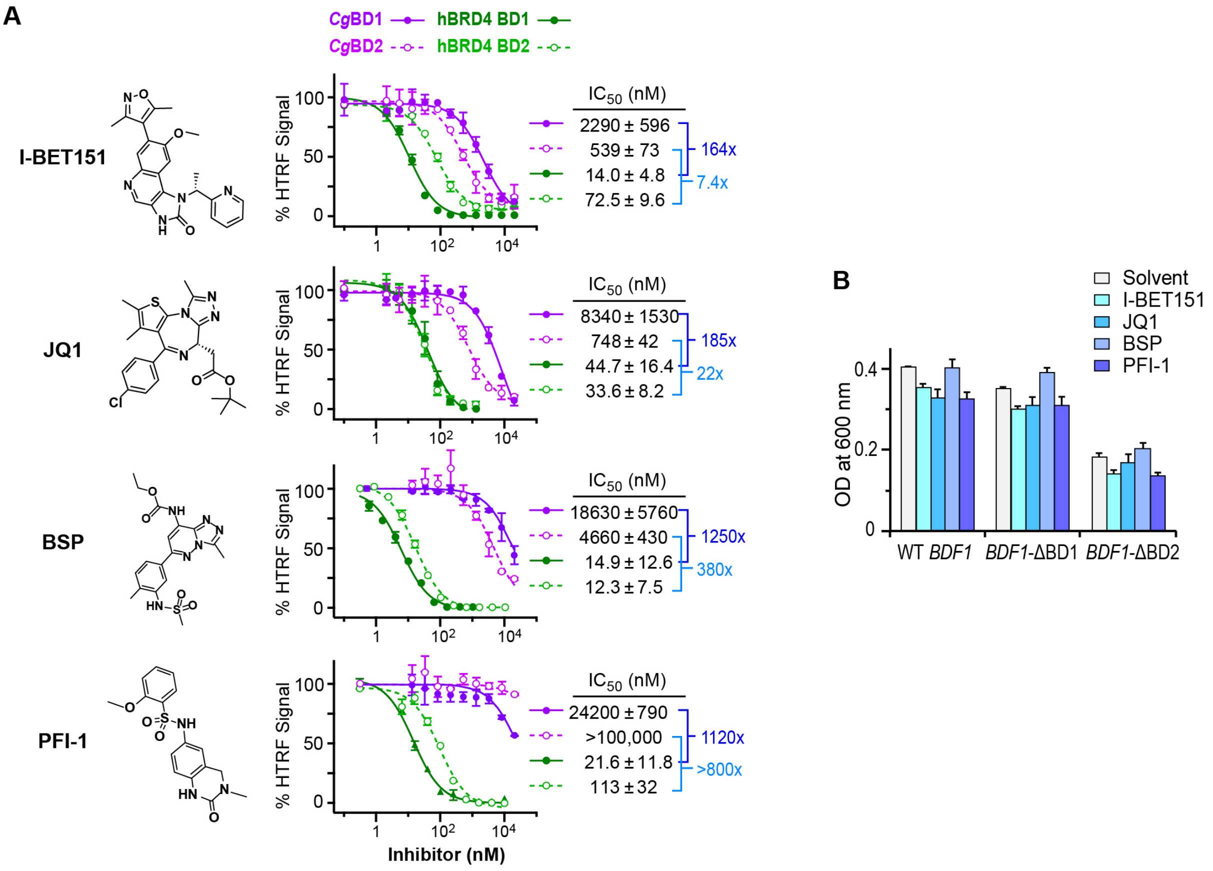

Figure S4. BET inhibitors discriminate human from *C. glabrata* BET BDs with high selectivity.

(**A**) HTRF assays performed on *Cg*Bdf1 BD1 and BD2 (closed purple and open magenta circles, respectively) and human Brd4 BD1 and BD2 (closed dark green and open light green circles, respectively) in the presence of the indicated BETi compound. Data represent the mean and s.d. values from three independent experiments. BSP, bromosporine.

(**B**) The four BET inhibitors do not affect *C. glabrata* growth, even when Bdf1 BD1 or BD2 is deleted. Expression of endogenous *BDF1* was suppressed by a 23 h incubation with methionine and cysteine and hence only the WT or mutant Bdf1 protein encoded by the plasmid was expressed. Strains were then incubated with BETi compounds at 10 μM for 24 h and viability was assessed. Data represent the mean and s.d. values from three biological replicates.

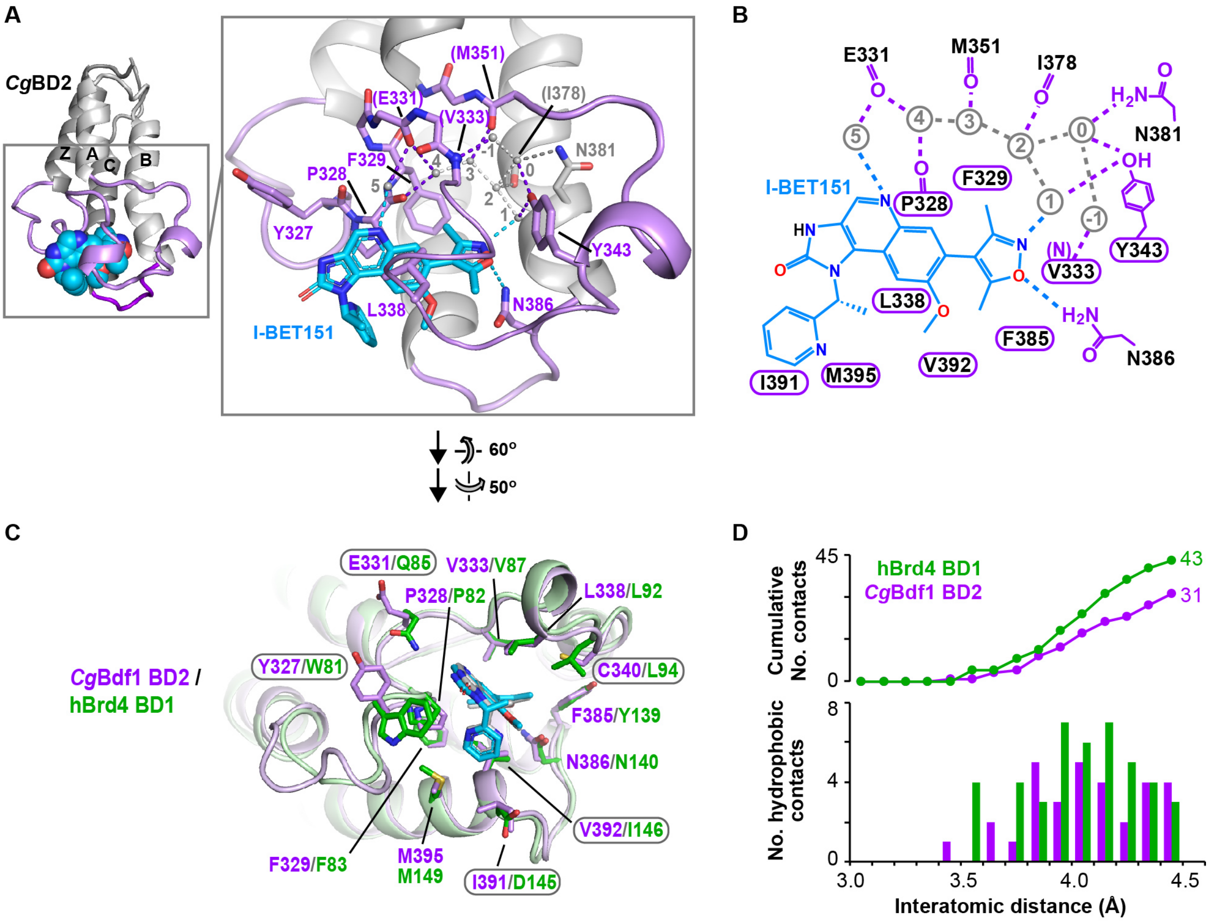

Figure S5. Structure of *Cg*BD2 bound to I-BET151.

(**A**) Hydrogen bonding interactions and water structure in the binding site. Residues interacting with I-BET151 through direct and water-mediated hydrogen bonds (dashed lines) are shown in stick representation. Residues interacting through backbone atoms are labelled in parentheses. Water molecules are indicated in grey and numbered as in ref. [11].

(**B**) Summary of ligand interactions. Hydrogen bonds are shown as dashed lines. Residues mediating van der Waals contacts with I-BET151 are indicated by labels within a cartouche.

(**C**) Alignment of the I-BET151-bound structures of *Cg*BD2 (violet) and human Brd4 BD1 (PDB 3ZYU; green). The ligands bound to *Cg*BD2 and to Brd4 BD1 are shown in cyan and grey, respectively. Labels within a cartouche indicate *Cg*BD2 residues that contact I-BET151 less intimately than the corresponding Brd4 BD1 residue.

(**D**) Number of hydrophobic contacts between BD atoms and I-BET151 plotted as a function of interatomic distance, shown as a histogram (bottom) and cumulative distribution (top). I-BET151 makes fewer hydrophobic contacts with *Cg*BD2 than with Brd4 BD1, especially at interatomic distances below 4 Å. Hydrophobic contacts were identified using Arpeggio [12].

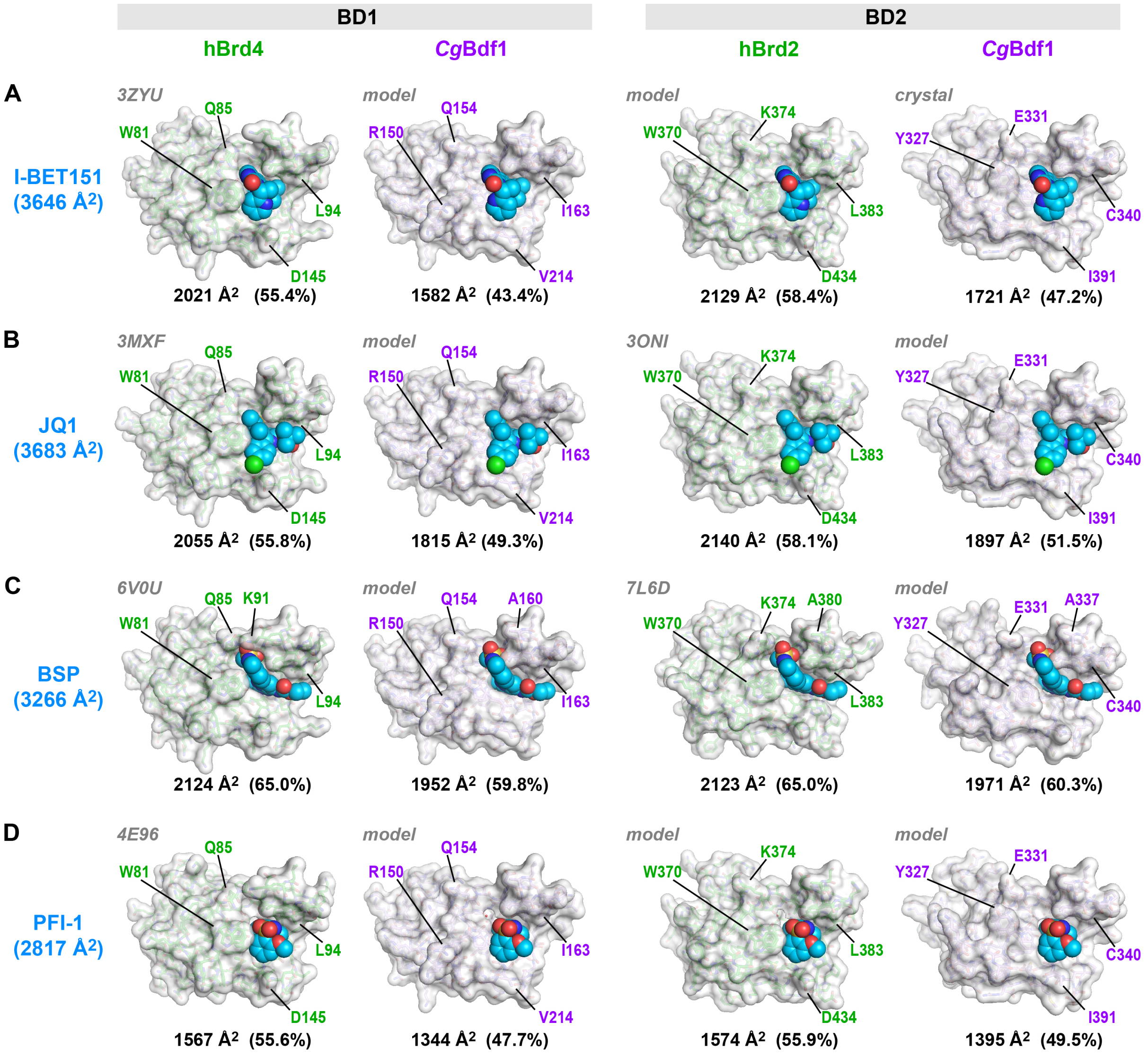

Figure S6. A wider binding pocket explains the lower potency of BET inhibitors toward *Cg*Bdf1 BDs.
(**A**, **B**, **C**, **D**) Crystal structures and hypothetical models of human BET and *Cg*Bdf1 BDs bound to (A) I-BET151, (B) JQ1, (C) bromosporine (BSP) and (D) PFI-1. The total solvent-accessible surface area (SASA) of the isolated BETi compound is shown in cyan. The compound's SASA that is buried by each BD is indicated below the structure. Crystal structures are shown for human Brd4 BD1 bound to I-BET151, JQ1, BSP and PFI-1, human Brd2 BD2 bound to JQ1 and BSP (PDB entries 3ZYU, 3MXF, 6V0U, 4E96, 3ONI and 7L6D, respectively) and *Cg*BD2 bound to I-BET151 (this work). Brd2 BD2 bound to I-BET151 and PFI-1 were modelled by aligning the corresponding ligand-bound structure of Brd4 BD1 onto that of JQ1-bound Brd2 BD2 and replacing the atomic coordinates of JQ1 with those of I-BET151 or PFI-1. BETi-bound *Cg*BD1 structures were modelled by aligning the structure of *Cg*BD2 bound to I-BET151 or that of Brd4 BD1 bound to JQ1, BSP or PFI-1 onto the unbound *Cg*BD1 crystal structure, altering the *Cg*BD1 residue Ile215 rotamer to match the corresponding Brd4 Ile146 rotamer (to avoid a steric clash with the ligand), and transferring the ligand coordinates. Structure alignments predict that JQ1 and PFI-1 are sterically incompatible with the *gauche*$^-$ conformation of *Cg*BD2 Tyr327 but that BSP is compatible with both the *trans* and *gauche*$^-$ conformations. Accordingly, JQ1- and PFI-1-bound *Cg*BD2 were modelled by aligning the corresponding Brd4 BD1 structure onto that of I-BET151-bound *Cg*BD2 and replacing the I-BET151 coordinates with those of JQ1 or PFI-1, whereas BSP-bound *Cg*BD2 was modelled by aligning the BSP-bound structure of Brd4 BD1 onto that of unbound *Cg*BD2 and transferring the BSP coordinates. All ions, non-BETi ligands and water molecules (except for the 6 structurally conserved waters, numbered -1 to 4 in fig. S2, C and D) were removed from each structure prior to calculating SASA values in PyMOL [7]. For JQ1 and PFI-1, the poorer fit to the CgBD binding pockets arises from the same structural differences between the human and fungal binding pockets as described for I-BET151 (see main text). An additional difference contributes to the poor fit of bromosporine: in the human BET BDs a lysine amino group (Lys91 in Brd4 BD1 and Lys374 in Brd2 BD2) is within H-bonding distance of the compound’s sulfonamide group, whereas the corresponding *Cg*BD1 (Ala160) and *Cg*BD2 (Glu331) side chains are either too short or lack the required proton to mediate such an interaction.

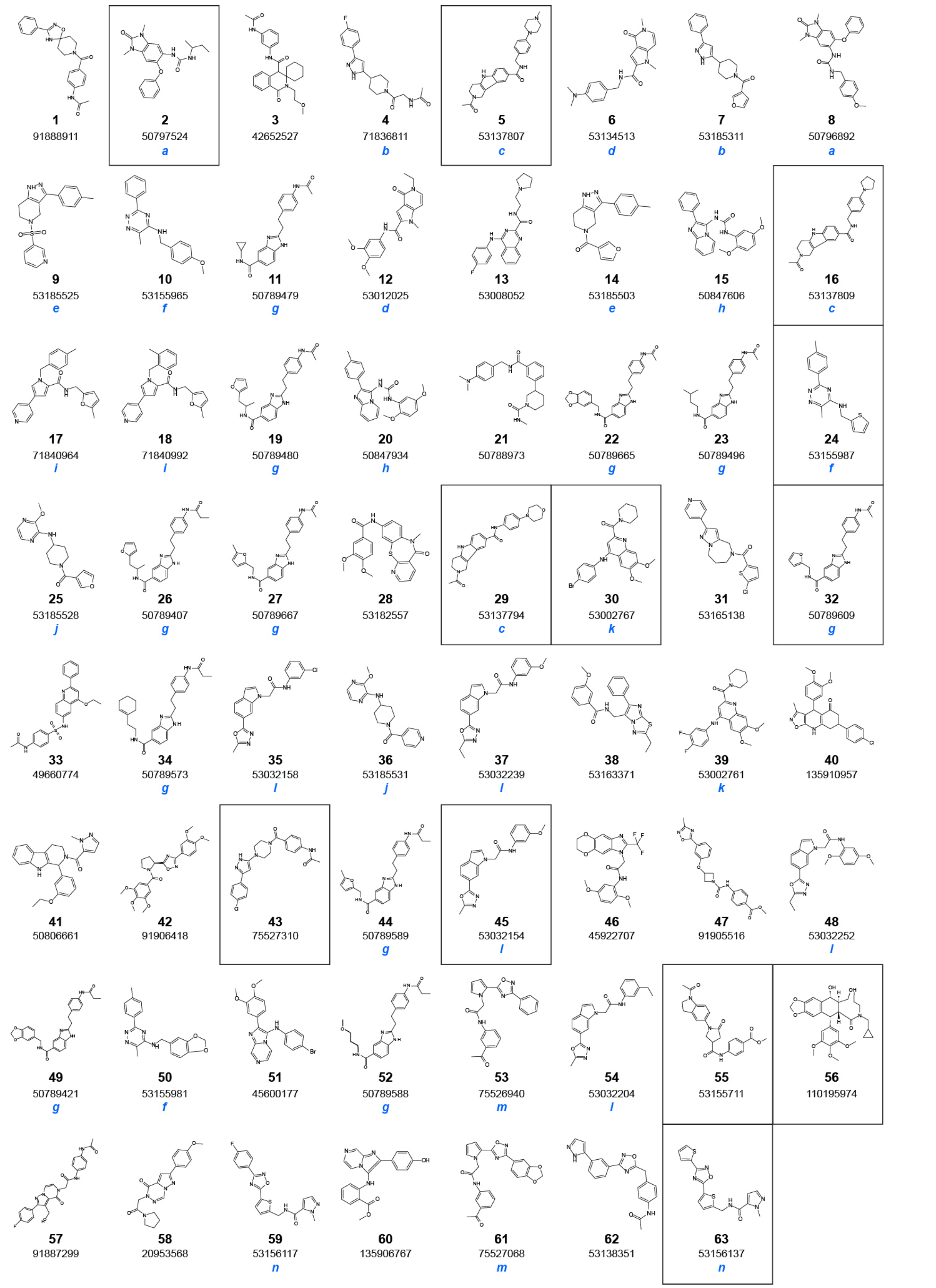

Figure S7. *Cg*Bdf1 BD1 inhibitors identified by high-throughput screening.

The PubChem Compound ID is indicated for each inhibitor. Compounds sharing the same scaffold are labeled with the same letter (*a*-*n*) in blue. The 12 compounds pursued for detailed characterization are boxed.

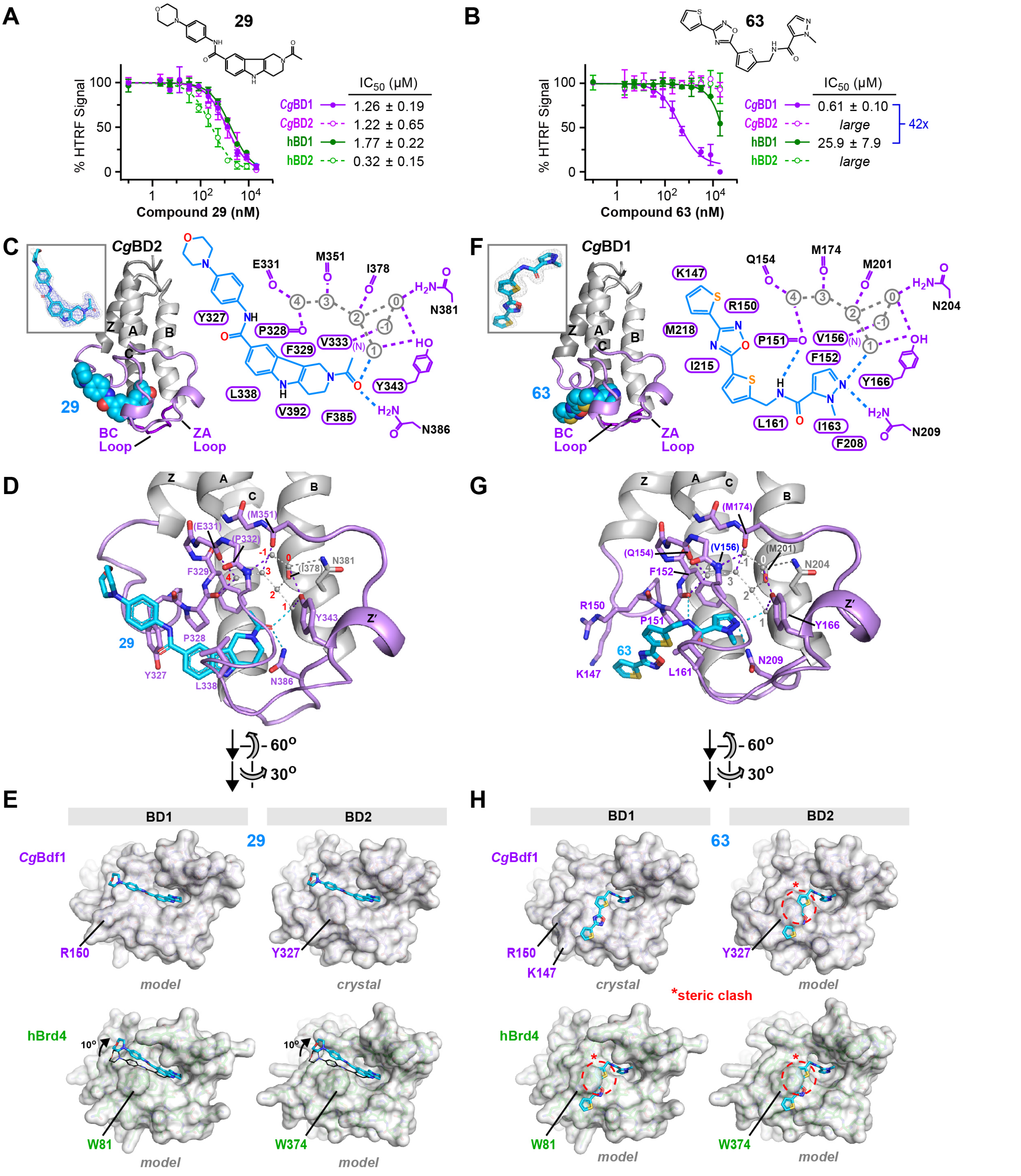

Figure S8. Structural basis of non-selective and selective inhibition of *Cg*Bdf1 BDs by 29 and 63.

(**A**, **B**) HTRF assays performed on *Cg*Bdf1 BD1 and BD2 (closed purple and open magenta circles, respectively) and human Brd4 BD1 and BD2 (closed dark green and open light green circles, respectively) in the presence of compound (A) **29** and (B) **63**. Data represent the mean and s.d. values from three independent experiments.

(**C**) Crystal structure of *Cg*BD2 bound to **29** and schematic summary of ligand interactions. Hydrogen bonds are shown as dashed lines. Residues mediating van der Waals contacts with **29** are indicated by labels within a cartouche. Water molecules are indicated in grey and are numbered as in ref. [11]. *Inset.* Simulated annealing omit F_o-F_c density for **29** contoured at 3 σ.

(**D**) Details of the ligand binding site. Residues interacting with **29** through direct and water-mediated hydrogen bonds (dashed lines) are shown in stick representation. Residues interacting through backbone atoms are labelled in parentheses.

(**E**) Surface representations of the *Cg*BD2/**29** crystal structure and of structural alignment models of *Cg*BD1, Brd4 BD1 and BD2 showing how the four BD binding pockets could accommodate **29**. The replacement of Tyr327 by the bulkier Trp81 and Trp370 residues in the human BDs would cause a steric clash with the **29** phenyl group, but this could easily be relieved by a small rotation of the compound without disrupting the intimate contacts at the opposite end of the molecule.

(**F**) Crystal structure of *Cg*BD1 bound to **63** and schematic summary of ligand interactions. *Inset.* Simulated annealing omit F_o-F_c density for **63** contoured at 3 σ.

(**G**) Details of the ligand binding site. Residues interacting with **63** through direct and water-mediated hydrogen bonds (dashed lines) are shown in stick representation. Residues interacting through backbone atoms are labelled in parentheses.

(**H**) Surface representations of the *Cg*BD1/**63** crystal structure and of structural alignment models of *Cg*BD2, Brd4 BD1 and BD2 showing observed or predicted interactions with the binding pocket. Steric clashes are indicated by red circles and asterisks. The alignment with *Cg*BD2 shows that the oxadiazole and two thiene moieties of **63** would extensively overlap with Tyr327 in its preferred *gauche*$^-$ conformation and, unlike I-BET151, topologically prevent its flipping to the *trans* form to accommodate the ligand, explaining the inability of **63** to inhibit *Cg*BD2. Similarly, a severe steric clash predicted between these moieties and WPF-shelf residues Trp81 and Trp374 explains the weak activity of **63** against the two Brd4 BDs.

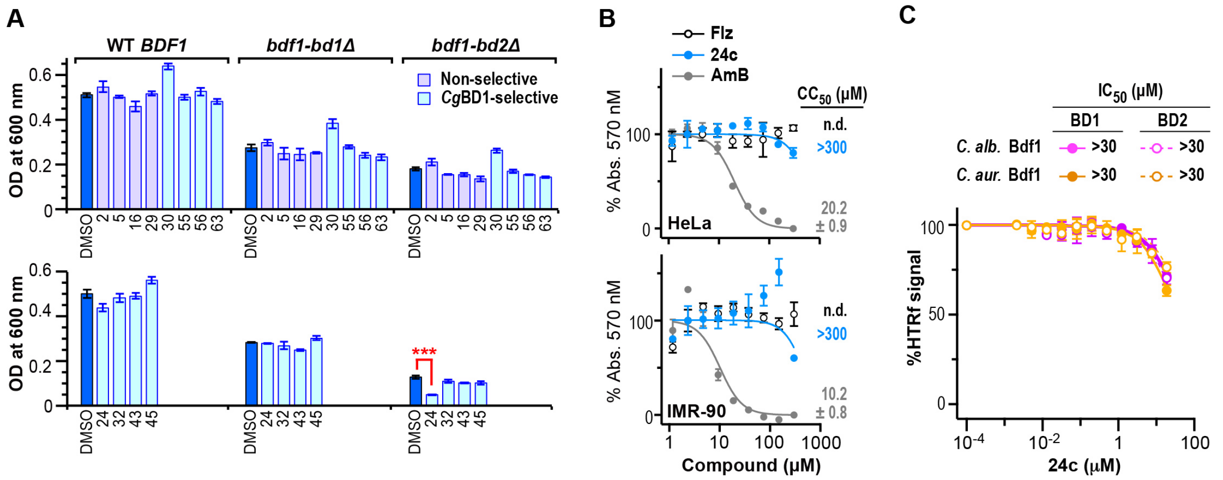

Figure S9. Characterization of 24c in fungal growth inhibition and human cytotoxicity assays.

(**A**) Summary of results for 12 hit compounds in growth inhibition assays against *C. glabrata* strains expressing WT and mutant Bdf1. Statistical significance was determined by the Holm-Sidak method in GraphPad Prism 10; *** $p \leq 0.001$. Data in panels (A)-(C) repesent the mean and s.d. values from three biological replicates.

(**B**) MTT cytotoxicity assays on HeLa and IMR90 cells. Antifungal compounds fluconazole (Flz) and Amphotericin B (AmB) were included as controls. Values of half cytotoxic concentration (CC_{50}) are indicated on the right.

(**C**) HTRF assays performed on Bdf1 BD1 and BD2 (closed and open circles, respectively) from *C. albicans* (magenta) and *C. auris* (orange) in the presence of **24c**. Data represent the mean and s.d. values from three independent experiments.

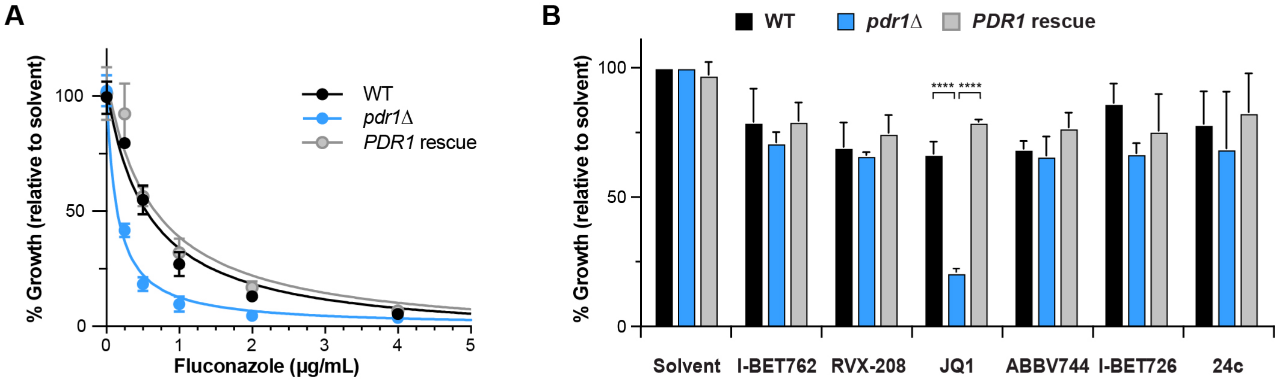

Figure S10. A *C. glabrata* efflux mutant strain exhibits increased susceptibility to JQ1.

(A) Deletion of *PDR1* in *C. glabrata* reduces efflux pump expression and sensitizes the strain to fluconazole, as previously described. [13]

(B) JQ1 shows increased efficacy against the *PDR1* deletion strain, while the activity of **24c** and of four other tested BET inhibitors was unaffected by the deletion. All compounds were tested at 50 µM, except I-BET726 (1 µM). Data in panels (A) and (B) represent the mean and s.d. values from three biological replicates, each comprising three technical replicates. Statistical significance was determined by a two-tailed *t*-test in GraphPad Prism 10; **** $p \leq 10^{-4}$.

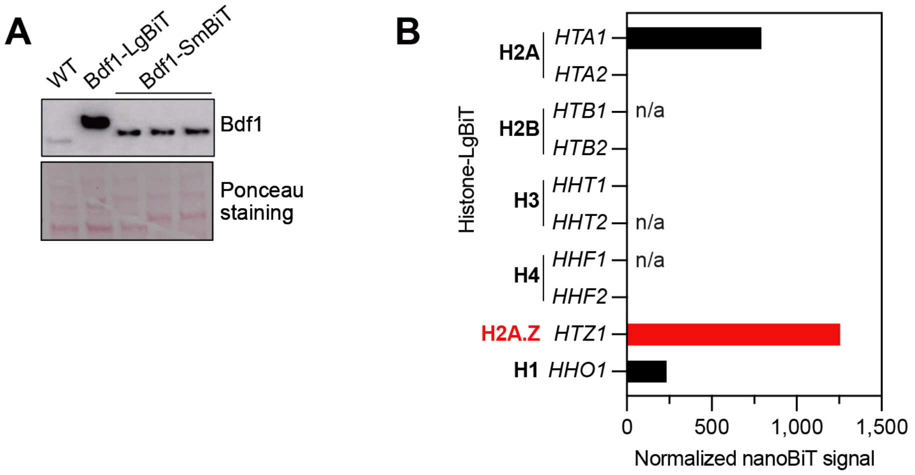

Figure S11. Development of the NanoBit assay.

(**A**) Expression levels in *S. cerevisiae* of Bdf1 when tagged with SmBiT or LgBiT. Bdf1-SmBiT is expressed at levels closer to those of the WT protein.

(**B**) Selection of the best Bdf1/histone gene pair. Several histone strains from the LgBiT C-SWAT collection were analyzed. H2A.Z (*HTZ1* gene) provided the best signal, probably because it is enriched at the same loci as Bdf1, on the +1 nucleosome (Bdf1 interacts with SWR1C, the complex that loads H2A.Z onto chromatin at the +1 nucleosome).

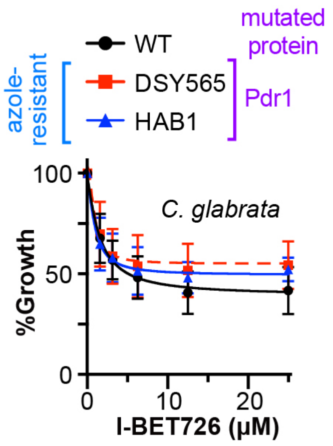

Figure S12. I-BET726 inhibits the growth of azole-resistant clinical *C. glabrata* strains harboring mutations in *PDR1*. Data represent the mean and s.d. values from three biological replicates.

Table S1. Crystallographic data collection and refinement statistics.

Bromodomain/ligand	*Cg*BD1 (unbound)	*Cg*BD1/ 63	*Cg*BD1/ 24c	*Cg*BD2 (unbound)	*Cg*BD2/ I-BET151	*Cg*BD2/ 29
PDB ID	8R6I	8R6J	8R6K	8R6L	8R6M	8R6N
**Data Collection**[a)]						
Synchrotron beamline	ESRF ID30A-1	ESRF ID30A-1	Soleil PX-1	ESRF ID30A-1	ESRF ID30A-3	ESRF ID30A-1
Wavelength (Å)	0.9660	0.9660	1.0332	0.9660	0.9677	0.9660
Space group	P1	$C222_1$	I422	$P2_1$	$C222_1$	$I4_122$
Unit cell dimensions						
a (Å)	22.89	32.84	128.70	80.29	70.59	67.58
b (Å)	40.59	142.62	"	67.69	88.43	"
c (Å)	48.87	93.55	148.21	96.62	257.79	152.83
α (°)	98.14	90	90	90	90	90
β (°)	94.37	"	"	95.5	"	"
γ (°)	94.77	"	"	90	"	"
Molecules in asym. unit	2	2	4	7	4	1
Resolution range (Å)	48.2 – 1.08	39.1 – 1.82	64.4 – 2.19	44.4 – 1.96	55.2 – 1.95[b)]	47.8 – 2.11[c)]
(outer shell)	(1.11 – 1.08)	(1.86 – 1.82)	(2.26 – 2.19)	(2.12 – 1.96)	(2.18 – 1.95)	(2.41 – 2.11)
No. of measured reflections	110,608 (6882)	82,654 (4328)	222,933 (19,474)	223,412 (45,343)	207,522 (10,888)	70,838 (2492)
No. of unique reflections	66,239 (4162)	20,025 (1160)	32,208 (2779)	73,550 (15349)	28,171 (1410)	5781 (289)
Multiplicity	1.7 (1.7)	4.1 (3.7)	6.9 (7.0)	3.0 (3.0)	7.4 (7.7)	12.3 (8.6)
Completeness (%) - spherical	88.5 (75.0)	99.1 (99.6)	100.0 (100.0)	99.0 (99.2)	47.4 (8.5)	54.7 (8.9)
- ellipsoidal					91.7 (76.9)	93.5 (81.5)
Mean I/sigma(I)	9.1 (1.1)	10.4 (1.7)	7.3 (3.4)	13.2 (1.8)	6.7 (1.7)	8.3 (1.9)
R_{meas}	0.052 (0.851)	0.116 (0.990)	0.220 (0.747)	0.050 (0.795)	0.292 (2.056)	0.277 (1.266)
$CC_{1/2}$	0.999 (0.540)	0.996 (0.532)	0.993 (0.415)	0.999 (0.823)	0.990 (0.601)	0.997 (0.696)
**Refinement**						
Resolution used for refinement	48.2 – 1.08	39.1 – 1.82	57.5-2.19	44.4 – 1.96	43.6 – 1.95	47.8 – 2.11
Reflections used (total/R_{free})	66,240 / 2099	19,989 / 1001	32,199/ 1661	73,405 / 3610	28,164 / 1407	5781 / 295
R_{work}/R_{free}	0.1736 / 0.1945	0.1988 / 0.2342	0.2090 / 0.2475	0.2001 / 0.2273	0.1892 / 0.2417	0.2574 / 0.2870
No. of atoms/<B-factor> (Å^2)						
Protein	1810 / 12.0	1780 / 31.4	3583 / 27.8	6618 / 76.9	3753 / 36.7	950 / 42.4
Water	390 / 21.3	161 / 33.1	432 / 30.1	179 / 52.9	226 / 30.4	23/ 24.6
Inhibitor	0 / --	76 / 29.3	120 / 45.7	0 / --	124 / 21.9	57/ 49.3
Other (buffer, ions)	0 / --	10 / 43.2	21 / 63.1	176 / 74.9	154 / 54.6	0 / --
RMS deviations:						
Bond distances (Å)	0.009	0.003	0.007	0.010	0.008	0.003
Bond angles (°)	1.051	0.606	0.812	0.931	0.920	0.677
Ramachandran analysis (%)						
Favored/ outliers	100 / 0.0	99.5/0.0	99.3 / 0.0	98.7 / 0.0	98.7 / 0.0	98.3 / 0.0
Molprobity analysis						
Clash Score / Overall score	1.64 / 0.91	4.82 / 1.25	6.44 / 1.36	1.87 / 0.95	5.70 / 1.31	3.97 / 1.18

[a)] Numbers in parentheses refer to the outer resolution shell.

[b)] Diffraction was anisotropic. The diffraction limits are 2.80 Å in the *a** and *b** directions and 2.11 Å in the *c** direction.

[c)] Diffraction was anisotropic. The diffraction limits are 2.73 Å, 2.89 Å and 1.95 Å in the *a**, *b** and *c** directions, respectively.

Table S2. RMSD values for alignments of *Cg*Bdf1 BDs with *Ca*Bdf1 and human BET BDs

Bromo-domain[a)]	PDB ID	No. independent copies[b)]	Residues aligned	Mean RMSD ± SD[c)]	
				***Cg*BD1**	***Cg*BD2**
*Cg*BD1	This work	2	130-144, 148-231	0.566[d)]	1.214 ± 0.046
*Ca*BD1	5n15	4	212-226, 230-313	0.834 ± 0.116	1.085 ± 0.046
Brd2 BD1	1x0j	3	77-91, 95-178	1.771 ± 0.086	1.434 ± 0.062
Brd3 BD1	2nxb	2	37-51, 55-138	1.813 ± 0.067	1.467 ± 0.021
Brd4 BD1	2oss	1	61-75, 79-162	1.791 ± 0.009	1.421 ± 0.016
Brdt BD1	7mra	1	61-75, 79-162	1.673 ± 0.062	1.367 ± 0.017
*Cg*BD2	This work	7	304-318, 325-408	1.214 ± 0.046	0.289 ± 0.053[e)]
*Ca*BD2	5n13	1	386-400, 407-490	1.677 ± 0.039	1.261 ± 0.064
Brd2 BD2	7jx7	1	347-361, 368-451	1.302 ± 0.024	0.858 ± 0.039
Brd3 BD2	2oo1	4	309-323, 330-413	1.323 ± 0.083	0.912 ± 0.080
Brd4 BD2	2ouo	1	351-365, 372-455	1.428 ± 0.011	1.099 ± 0.040
Brdt BD2	7lej	1	270-284, 291-374	1.527 ± 0.047	1.032 ± 0.041

[a)] All BD structures are in the unbound state except for Brd2 BD2 (bound to a peptide) and Brdt BD1 and BD2 (bound to small molecules).
[b)] Number of BD molecules in the asymmetric unit.
[c)] RMSD values were calculated by program lsqkab of the CCP4 suite [3] using the Cα positions of the aligned residues indicated. The mean RMSD and standard deviation are reported for all pairwise alignments of independent molecules of the compared BD structures.
[d)] RMSD between the two different molecules in the asymmetric unit.
[e)] Mean pairwise RMSD between non-identical molecules in the asymmetric unit.

Table S3. IC$_{50}$ estimates for HTS hit compounds in preliminary HTRF assay.

	IC$_{50}$ [μM] [a)]					IC$_{50}$ [μM]			
**Com-pound**	***Cg*Bdf1 BD1**	***Cg*Bdf1 BD2**	**Brd4 BD1**	**Brd4 BD2**	**Com-pound**	***Cg*Bdf1 BD1**	***Cg*Bdf1 BD2**	**Brd4 BD1**	**Brd4 BD2**
**1**	9.4	>20[b)]	>20	>20	**33**	6.1	*L*	*L*	*L*
**2*** [c)]	4.4	1.1	13	2.8	**34**	>20	>20	*L*	*L*
**3**	19	>20	>20	>20	**35**	>20	*L*	>20	*L*
**4**	>20	*L*[b)]	*L*	*L*	**36**	*L*	*L*	*L*	>20
**5***	1.8	1.0	2.5	1.4	**37**	>20	*L*	*L*	*L*
**6**	19	8.2	>20	2.7	**38**	>20	6.5	*L*	>20
**7**	*L*	*L*	>20	>20	**39**	6.7	*L*	*L*	*L*
**8**	6.4	6.3	18	12	**40**	>20	*L*	*L*	>20
**9**	*L*	*L*	*L*	*L*	**41**	>20	>20	*L*	>20
**10**	5.0	>20	*L*	*L*	**42**	5.9	8.8	19	14
**11**	>20	*L*	*L*	*L*	**43***	2.7	13	>20	>20
**12**	>20	*L*	>20	10	**44**	19	>20	>20	*L*
**13**	>20	*L*	*L*	*L*	**45***	4.2	*L*	>20	*L*
**14**	*L*	*L*	>20	>20	**46**	>20	*L*	*L*	*L*
**15**	*L*	*L*	*L*	>20	**47**	11	*L*	>20	*L*
**16***	1.8	0.30	1.2	1.5	**48**	>20	*L*	*L*	>20
**17**	7.0	21	9.5	>20	**49**	>20	*L*	*L*	*L*
**18**	7.8	13	16	>20	**50**	>20	*L*	*L*	*L*
**19**	>20	*L*	>20	*L*	**51**	12	>20	12	>20
**20**	23	>20	>20	>20	**52**	>20	*L*	*L*	*L*
**21**	>20	>20	>20	3.5	**53**	>20	*L*	*L*	*L*
**22**	21	>20	>20	>20	**54**	>20	*L*	*L*	*L*
**23**	>20	*L*	*L*	*L*	**55***	0.11	*L*	>20	*L*
**24***	2.5	>20	>20	>20	**56***	0.36	*L*	*L*	*L*
**25**	>20	>20	>20	>20	**57**	>20	*L*	*L*	*L*
**26**	8.4	*L*	>20	*L*	**58**	>20	>20	>20	>20
**27**	5.9	17	>20	*L*	**59**	14	*L*	>20	>20
**28**	>20	>20	>20	3.5	**60**	>20	>20	>20	>20
**29***	1.1	2.3	2.9	0.44	**61**	>20	>20	>20	*L*
**30***	0.64	*L*	>20	>20	**62**	>20	*L*	>20	*L*
**31**	6.3	*L*	>20	*L*	**63***	0.62	*L*	>20	*L*
**32***	2.9	>20	>20	>20					

[a)] All IC$_{50}$ values were determined from a single-replicate HTRF assay.
[b)] IC$_{50}$ values for compounds yielding weak (<40%) or no detectable inhibition at the maximal tested concentration are indicated as >20 and *L* (large), respectively.
[c)] Compounds retained for further characterization are indicated by an asterisk.

Table S4. **IC_{50} values of HTS hits with highest potency towards *Cg*BD1.**

Compound	IC_{50} [µM] [a)]			
	***Cg*BD1**	***Cg*BD2**	**hBrd4 BD1**	**hBrd4 BD2**
**2**	4.97 ± 0.77	0.97 ± 0.25	21.8 ± 3.6	2.93 ± 0.57
**5**	1.95 ± 0.35	2.22 ± 1.68	3.07 ± 0.52	1.33 ± 0.07
**16**	4.65 ± 2.74	0.23 ± 0.08	1.13 ± 0.29	1.57 ± 0.10
**24**	2.23 ± 0.26	>20 [b)]	>20	>20
**29**	1.81 ± 1.14	2.06 ± 0.23	2.73 ± 0.34	0.52 ± 0.20
**30**	0.96 ± 0.32	*L* [b)]	>20	>20
**32**	2.84 ± 0.59	>20	>20	>20
**43**	3.20 ± 0.59	*n.d.* [c)]	>20	>20
**45**	3.08 ± 1.86	*L*	>20	*L*
**55**	0.10 ± 0.03	*L*	*L*	*L*
**56**	0.38 ± 0.17	*L*	*L*	*L*
**63**	0.61 ± 0.10	*L*	25.9 ± 7.9	*L*

[a)] Data are presented as means ± SD for *n*=3 independent experiments.
[b)] IC_{50} values for compounds yielding weak (<40%) or no detectable inhibition at the maximal tested concentration (20 µM) are indicated as >20 and *L* (large), respectively.
[c)] A reliable value could not be determined because of unusually large scatter of the data.

Table S5. Growth inhibition of clinical strains of *C. albicans* by I-BET726.

Strains	Resistance	Mutations[a)]	EC_{50} [µM][b)]
C. albicans			
ATCC90028	–	WT	4.25 ± 0.47
DSY292	Azoles	Erg11 (Y132H, G464S, R467K)	3.07 ± 0.26
CAAL74	Fluconazole	Erg11 (Y132F, E266D, G448V, V488I)	5.87 ± 1.03
DSY296	Azoles	Erg11 (G129A, G464S, G450E),<br>Tac1 (N977D)	7.36 ± 0.76
DSY2323	Azoles	Erg11 (S405F),<br>Tac1 (G980E)	11.5 ± 1.7
AF1	Echinocandins	Fks1-HS1 (S645P)	7.13 ± 1.48
AF2	"	Fks1-HS1 (S645P)	4.59 ± 0.95
AF3	"	Fks1-HS1 (S645P)	6.83 ± 0.26
CagR	"	Not characterized	4.14 ± 0.48
C. glabata			
ATCC2001	–	WT	1.52 ± 0.44
DSY565	Azoles	Pdr1 (L280F)	1.14 ± 0.62
HAB1	Azoles	Pdr1 (T370I)	1.66 ± 0.56

[a)] All mutant strains were characterized in ref.[14] except for HAB1 which was characterized in ref.[15].
[b)] Data are presented as means ± SD from 3 independent experiments.

Table S6. **Plasmids used in this study.**

Name	Internal ref.	Description	Cassette to transform
HIS3-pMET-*BDF1*	pJG253	pCR2.1 TOPO containing *HIS3* marker, MET3 promoter and flanking regions for integration in *BDF1* promoter	Digestion with XhoI and SacI
URA3-pGRB2.0	pJG244	pGRB2.0 containing *URA3* marker	Autonomous plasmid
BDF1-URA3	pJG245/267	pGRB2.0 containing *BDF1* ORF fused to *URA3* marker	" "
BDF1-ΔBD1	pJG269	pGRB2.0 containing *BDF1*-BD1 del sequence fused to *URA3* marker	" "
BDF1-ΔBD2	pJG271	pGRB2.0 containing *BDF1*-BD2 del sequence fused to *URA3* marker	" "
BDF1-ΔBD1ΔBD2	pJG272	pGRB2.0 containing *BDF1*-BD1 del-BD2 del sequence fused to *URA3* marker	" "
BDF1-Y166F	pJG277	pGRB2.0 containing *BDF1*-BD1Y166F sequence fused to *URA3* marker	" "
BDF1-Y343F	pJG275	pGRB2.0 containing *BDF1*-BD2Y343F sequence fused to *URA3* marker	" "
BDF1-Y166F-Y343F	pJG278	pGRB2.0 containing *BDF1*-BD1Y166F-BD2Y343F sequence fused to *URA3* marker	" "
BDF1-FLAG	pJG413	pGRB2.0 containing *BDF1*-FLAG sequence fused to *URA3* marker	" "
BDF1-hBD1-hBD2-FLAG	pJG483	pGRB2.0 containing *BDF1*-hBD1-hBD2-FLAG sequence fused to *URA3* marker	" "
BDF1-hBD1-FLAG	pJG484	pGRB2.0 containing *BDF1*-hBD1-FLAG sequence fused to *URA3* marker	" "
BDF1-hBD2-FLAG	pJG485	pGRB2.0 containing *BDF1*-hBD2-FLAG sequence fused to *URA3* marker	" "
HIS3-BDF1-hBD1-hBD2-FLAG	pJG504b	pCR2.1 TOPO containing *BDF1*- hBD1-hBD2-FLAG sequence fused to *HIS3* marker	Digestion with XhoI and SacI
HIS3-BDF1-FLAG	pJG505	pCR2.1 TOPO containing *BDF1*-FLAG sequence fused to *HIS3* marker	Digestion with XhoI and SacI
CgBDF1-SmBIT	pJG520	pRS416 plasmid containing *CgBDF1*-SmBIT ORF	
CgBDF1(Y166F,Y343F)-SmBIT	pJG531	pRS416 plasmid containing *CgBDF1*(Y166F,Y343F)-SmBIT ORF	
CgBDF1(ΔBD1,ΔBD2-SmBIT	pJG532	pRS416 plasmid containing *CgBDF1*(ΔBD1,ΔBD2 -SmBIT ORF	
ScBDF1-SmBIT	pJG533	pRS416 plasmid containing Sc*BDF1*-SmBIT ORF	
ScBDF1(Y187F,Y354F)-SmBIT	pJG534	pRS416 plasmid containing *ScBDF1*(Y187F,Y354F)-SmBIT ORF	

Table S7. ***C. glabrata*, *C.* albicans and *S. cerevisiae* strains used in this study.**

C. glabrata

Name	Internal ref.	Parent	Genotype	Plasmid
ATCC2001	Clin02	-	Wildtype	-
ATCC2001 HTU (ATCC200989)	CgJG1	-	*his3Δ trp1Δ ura3Δ*	-
ATCC2001 HTL	CgJG72	-	*leu2::trp1::his3*	-
WT *BDF1* + Empty plasmid	CgJG26	CgJG1	*ura3::trp1::his3*	pJG244
pMET-*BDF1*	CgJG12	CgJG1	pMET-*BDF1::HIS3 his3Δ trp1Δ ura3Δ*	-
pMET-*BDF1* + WT *BDF1*	CgJG19/20	CgJG12	pMET-*BDF1::HIS3 his3Δ trp1Δ ura3Δ*	pJG245
pMET-*BDF1* + *BDF1*-ΔBD1	CgJG35/36	CgJG12	pMET-*BDF1::HIS3 his3Δ trp1Δ ura3Δ*	pJG269
pMET-*BDF1* + *BDF1*-ΔBD2	CgJG39/40	CgJG12	pMET-*BDF1::HIS3 his3Δ trp1Δ ura3Δ*	pJG271
pMET-*BDF1* + *BDF1*-ΔBD1ΔBD2	CgJG43/44	CgJG12	pMET-*BDF1::HIS3 his3Δ trp1Δ ura3Δ*	pJG272
pMET-*BDF1* + *BDF1*-BD1 Y-F	CgJG1/52	CgJG12	pMET-*BDF1::HIS3 his3Δ trp1Δ ura3Δ*	pJG277
pMET-*BDF1* + *BDF1*-BD2 Y-F	CgJG47/49	CgJG12	pMET-*BDF1::HIS3 his3Δ trp1Δ ura3Δ*	pJG275
pMET-*BDF1* + *BDF1*-BD1&BD2 Y-F	CgJG55/56	CgJG12	pMET-*BDF1::HIS3 his3Δ trp1Δ ura3Δ*	pJG278
pMET-*BDF1* + *BDF1*-FLAG	CgJG80	CgJG12	pMET-*BDF1::HIS3 his3Δ trp1Δ ura3Δ*	pJG413
pMET-*BDF1* + *BDF1*-hBD*-FLAG	CgJG88	CgJG12	pMET-*BDF1::HIS3 his3Δ trp1Δ ura3Δ*	pJG483
pMET-*BDF1* + *BDF1*-hBD1-FLAG	CgJG89	CgJG12	pMET-*BDF1::HIS3 his3Δ trp1Δ ura3Δ*	pJG484
pMET-*BDF1* + *BDF1*-hBD2-FLAG	CgJG90	CgJG12	pMET-*BDF1::HIS3 his3Δ trp1Δ ura3Δ*	pJG485
BDF1-hBD*	CgJG92	CgJG72	*BDF1-hBD1-hBD2::HIS3 his3Δ trp1Δ ura3Δ*	-
BDF1-FLAG	CgJG95b	CgJG72	*BDF1-FLAG::HIS3 his3Δ trp1Δ ura3Δ*	-
BDF1-hBD*-FLAG	CgJG93b	CgJG72	*BDF1-hBD1-hBD2-FLAG::HIS3 his3Δ trp1Δ ura3Δ*	-
DSY562 WT *PDR1* [a)]	CgJG103			-
SFY92 *pdr1Δ* [b)]	CgJG101	DSY562	*pdr1Δ::SAT1-FLIP*	-
SFY114 *PDR1* rescue [b)]	CgJG102	SFY92	*pdr1Δ::CgPDR1-SAT1*	-

C. albicans

Name	Internal ref.	Parent	Genotype
SN152			*ura3/::imm434::URA3/ura3::imm434 iro1::IRO1/iro1::imm434 his1::hisG/his1::hisG leu2/leu2 arg4/arg4*
BDF1	Ca174	SN152	*SN152 + bdf1::BDF1-ARG4 / "-LEU2*
BDF1-hBD*	Ca165	SN152	*SN152 + bdf1::BDF1-hBD1-hBD2-ARG4 / "-LEU2*

S. cerevisiae

Name	Internal ref.	Parent	Genotype	Plasmid
HTZ1-LgBiT	scGR1721		*ura3Δ0 leu2Δ0 his3Δ1 met15Δ0*<br>*can1::STE3pr-LEU2-GAL1pr-NLS-SceI*<br>*lyp1Δ*<br>*htz1::HTZ1-LgBiT-10HIS-HPH*	
CgBdf1-SmBiT<br>Htz1-LgBiT	scGB0003<br>scGB0004<br>scGB0005	scGR1721	*ura3Δ0 leu2Δ0 his3Δ1 met15Δ0*<br>*can1::STE3pr-LEU2-GAL1pr-NLS-SceI*<br>*lyp1Δ*<br>*htz1::HTZ1-LgBiT-10HIS-HPH*	pJG520
CgBdf1(Y166F,Y343F)-SmBiT<br>Htz1-LgBiT	scGB0009<br>scGB0010<br>scGB0011	scGR1721	*ura3Δ0 leu2Δ0 his3Δ1 met15Δ0*<br>*can1::STE3pr-LEU2-GAL1pr-NLS-SceI*<br>*lyp1Δ*<br>*htz1::HTZ1-LgBiT-10HIS-HPH*	pJG531
CgBdf1(ΔBD1, ΔBD2)-SmBiT	scGB0012	scGR1721	*ura3Δ0 leu2Δ0 his3Δ1 met15Δ0*	pJG532

Htz1-LgBiT	scGB0013<br>scGB0014		*can1::STE3pr-LEU2-GAL1pr-NLS-SceI*<br>*lyp1Δ*<br>*htz1::HTZ1-LgBiT-10HIS-HPH*	
ScBdf1-SmBiT<br>Htz1-LgBiT	scGB0015<br>scGB0016	scGR1721	*ura3Δ0 leu2Δ0 his3Δ1 met15Δ0*<br>*can1::STE3pr-LEU2-GAL1pr-NLS-SceI*<br>*lyp1Δ*<br>*htz1::HTZ1-LgBiT-10HIS-HPH*	pJG533
ScBdf1(Y187F,Y354F)-SmBiT<br>Htz1-LgBiT	scGB0017<br>scGB0018	scGR1721	*ura3Δ0 leu2Δ0 his3Δ1 met15Δ0*<br>*can1::STE3pr-LEU2-GAL1pr-NLS-SceI*<br>*lyp1Δ*<br>*htz1::HTZ1-LgBiT-10HIS-HPH*	pJG534
Bdf1-SmBiT<br>Htz1-LgBiT	scGR1730	scGR1721	*ura3Δ0 leu2Δ0 his3Δ1 met15Δ0*<br>*can1::STE3pr-LEU2-GAL1pr-NLS-SceI*<br>*lyp1Δ*<br>*bdf1::BDF1-SmBiT-NAT*<br>*htz1::HTZ1-LgBiT-10HIS-HPH*	
Bdf1-SmBiT<br>Swc4-LgBiT	scGR1732		*ura3Δ0 leu2Δ0 his3Δ1 met15Δ0*<br>*can1::STE3pr-LEU2-GAL1pr-NLS-SceI*<br>*lyp1Δ*<br>*bdf1::BDF1-SmBiT-NAT*<br>*swc4::SWC4-LgBiT-10HIS-HPH*	
Bmh1-SmBiT<br>Bmh2-LgBiT	scGR1726		*ura3Δ0 leu2Δ0 his3Δ1 met15Δ0*<br>*can1::STE3pr-LEU2-GAL1pr-NLS-SceI*<br>*lyp1Δ*<br>*bmh1::BMH1-LgBiT-10HIS-HPH*<br>*bmh2::BMH2-SmBiT-NAT*	

a) Strain described in ref. [16].

b) Strain described in ref. [13].

Table S8. Other *Candida* strains used in this study.

Species	Origin name	Internal ref.	Description
C. albicans	ATCC90028	Clin01	No known resistance
C. tropicalis	ATCC7349	Clin03	" " "
C. parapsilosis	ATCC22019	Clin04	" " "
C. krusei	ATCC6258	Clin05	" " "
C. auris	CBS10913	Clin06	" " "
C. albicans	CagS	Clin07	" " "
C. albicans	CagR	Clin08	Echinocandin resistant; serial isolate with Clin07
C. albicans	DSY292	Clin11	Azole resistant
C. albicans	DSY296	Clin12	" "
C. albicans	DSY2323	Clin15	" "
C. albicans	CAAL74	Clin16	" "
C. albicans	AF1	Clin17	Echinocandin resistant
C. albicans	AF2	Clin18	" "
C. albicans	AF3	Clin19	" "
C. glabrata	DSY565	Clin23	Azole resistant
C. glabrata	HAB1	Clin24	" "

All mutant strains were characterized in ref.[14] except for HAB1 which was characterized in ref.[15].

List of Supplementary Files

The following files are provided as additional supporting materials.

1) Original images of Western blots and gels.

PDF file containing original images of Western blots and gels shown in Figures 3C, S1B, S1D, S3B-C, S3E and S10A.

2) Summary of peptide array data.

Excel file listing histone peptide sequences and intensities of spots shown in Figure S1B.

3) Original data used to generate graphs and histograms.

Excel file containing raw data used to generate plots, graphs and histograms shown in Figures 1D, 2A, 2C-F, 3B, 3D-F, 4B-C, 5A-E, 6A-D, S3D, S4A-B, S5D, S8A-B, S9A-C, S10, S11B, S12 and Table S4.

4) PDB validation reports.

PDF file containing PDB validation reports for the six new crystal structures reported in this study.

Supplementary References

[1] F. Mietton, E. Ferri, M. Champleboux, N. Zala, D. Maubon, Y. Zhou, M. Harbut, D. Spittler, C. Garnaud, M. Courcon, M. Chauvel, C. d'Enfert, B. A. Kashemirov, M. Hull, M. Cornet, C. E. McKenna, J. Govin, C. Petosa, *Nat Commun* **2017**, *8*, 15482.

[2] W. Kabsch, *Acta crystallographica* **2010**, *66*, 125-132.

[3] M. D. Winn, C. C. Ballard, K. D. Cowtan, E. J. Dodson, P. Emsley, P. R. Evans, R. M. Keegan, E. B. Krissinel, A. G. Leslie, A. McCoy, S. J. McNicholas, G. N. Murshudov, N. S. Pannu, E. A. Potterton, H. R. Powell, R. J. Read, A. Vagin, K. S. Wilson, *Acta crystallographica* **2011**, *67*, 235-242.

[4] A. J. McCoy, R. W. Grosse-Kunstleve, P. D. Adams, M. D. Winn, L. C. Storoni, R. J. Read, *J Appl Crystallogr* **2007**, *40*, 658-674.

[5] P. Emsley, B. Lohkamp, W. G. Scott, K. Cowtan, *Acta crystallographica* **2010**, *66*, 486-501.

[6] P. D. Adams, P. V. Afonine, G. Bunkoczi, V. B. Chen, I. W. Davis, N. Echols, J. J. Headd, L. W. Hung, G. J. Kapral, R. W. Grosse-Kunstleve, A. J. McCoy, N. W. Moriarty, R. Oeffner, R. J. Read, D. C. Richardson, J. S. Richardson, T. C. Terwilliger, P. H. Zwart, *Acta crystallographica* **2010**, *66*, 213-221.

[7] L. L. C. Schrödinger, **2023**.

[8] A. Walther, J. Wendland, *Curr Genet* **2003**, *42*, 339-343.

[9] R. E. Zordan, Y. Ren, S. J. Pan, G. Rotondo, A. De Las Penas, J. Iluore, B. P. Cormack, *G3* **2013**, *3*, 1675-1686.

[10] E. Garcia-Oliver, C. Ramus, J. Perot, M. Arlotto, M. Champleboux, F. Mietton, C. Battail, A. Boland, J. F. Deleuze, M. Ferro, Y. Coute, J. Govin, *PLoS Genet* **2017**, *13*, e1006541.

[11] E. M. Flynn, O. W. Huang, F. Poy, M. Oppikofer, S. F. Bellon, Y. Tang, A. G. Cochran, *Structure* **2015**, *23*, 1801-1814.

[12] H. C. Jubb, A. P. Higueruelo, B. Ochoa-Montano, W. R. Pitt, D. B. Ascher, T. L. Blundell, *Journal of molecular biology* **2017**, *429*, 365-371.

[13] S. Ferrari, F. Ischer, D. Calabrese, B. Posteraro, M. Sanguinetti, G. Fadda, B. Rohde, C. Bauser, O. Bader, D. Sanglard, *PLoS Pathog* **2009**, *5*, e1000268.

[14] C. Garnaud, F. Botterel, N. Sertour, M. E. Bougnoux, E. Dannaoui, S. Larrat, C. Hennequin, J. Guinea, M. Cornet, D. Maubon, *J Antimicrob Chemother* **2015**, *70*, 2556-2565.

[15] L. Vale-Silva, E. Beaudoing, V. D. T. Tran, D. Sanglard, *G3* **2017**, *7*, 2413-2426.

[16] D. Sanglard, F. Ischer, D. Calabrese, P. A. Majcherczyk, J. Bille, *Antimicrob Agents Chemother* **1999**, *43*, 2753-2765.

LRMS data for compounds 24c, 24k, 24l and 24m.

Compound: **24c**

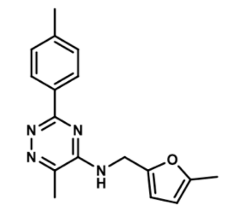

$C_{17}H_{18}N_4O$
M.W.=294.36

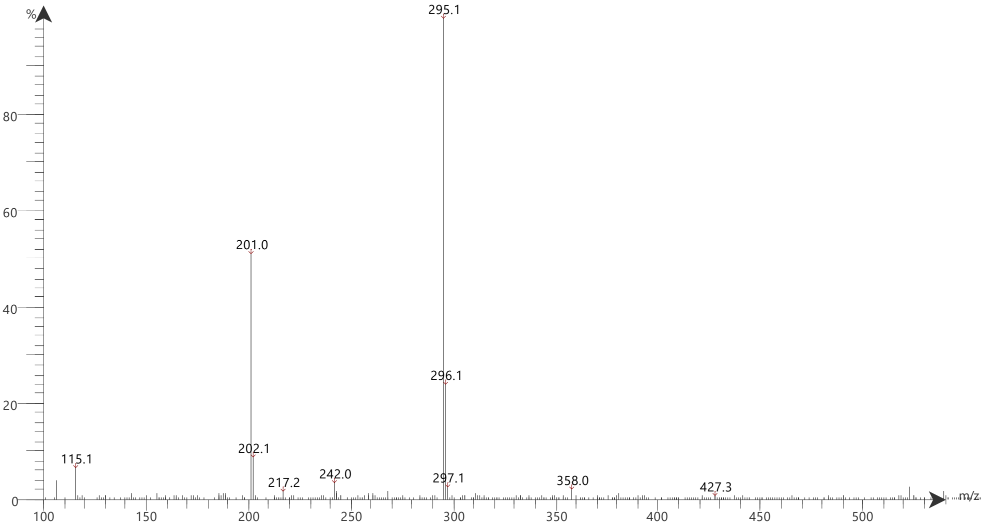

Calcd *m/z* for $[M+H]^+$: 295.16; found: 295.1 (100%).

Compound: **24k**

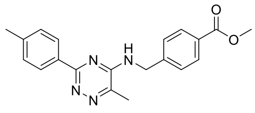

$C_{20}H_{20}N_4O_2$
M.W.=348.41

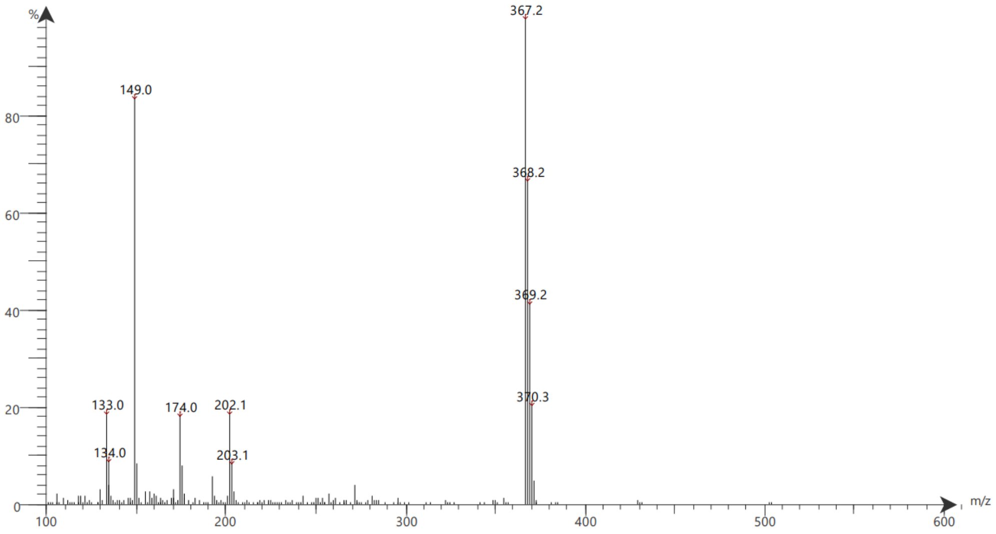

Calcd *m/z* $[M+H_3O]^+$: 367.18, found: 367.2 (100%)

Compound: **24l**

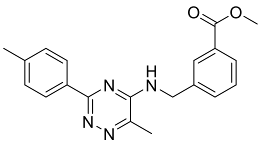

$C_{20}H_{20}N_4O_2$
M.W.=348.41

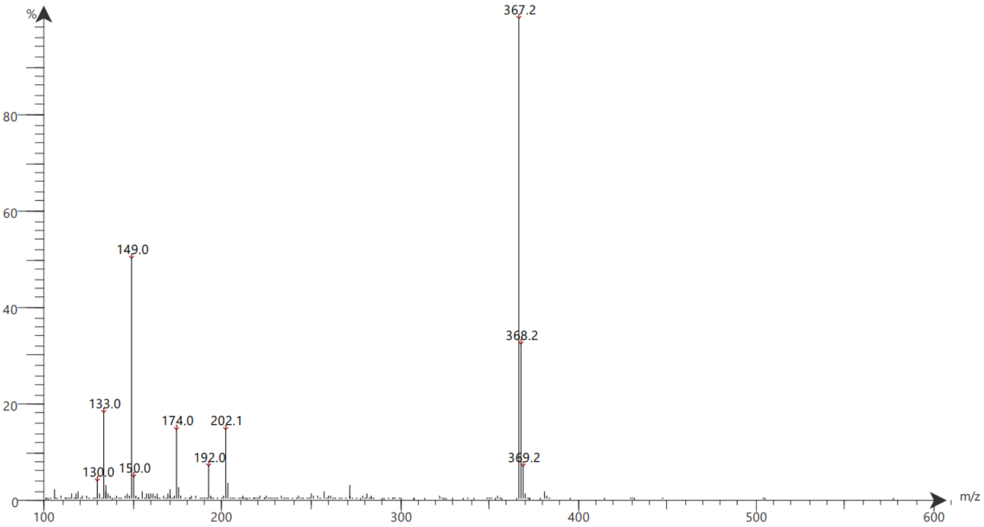

Calcd *m/z* $[M+H_3O]^+$: 367.18, found: 367.2 (100%)

Compound: **24m**

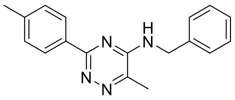

$C_{18}H_{18}N_4$
M.W.: 290.37

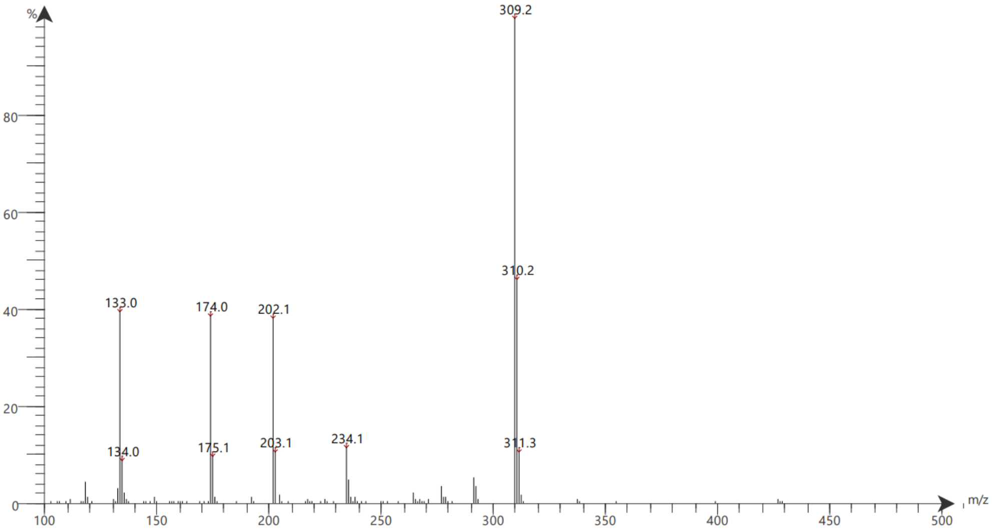

Calcd *m/z* $[M+H_3O]^+$: 309.17, found: 309.2 (100%).

NMR spectra for compounds 24c, 24k, 24l and 24m.

24c ^{1}H NMR (400 MHz, CD_3OD)

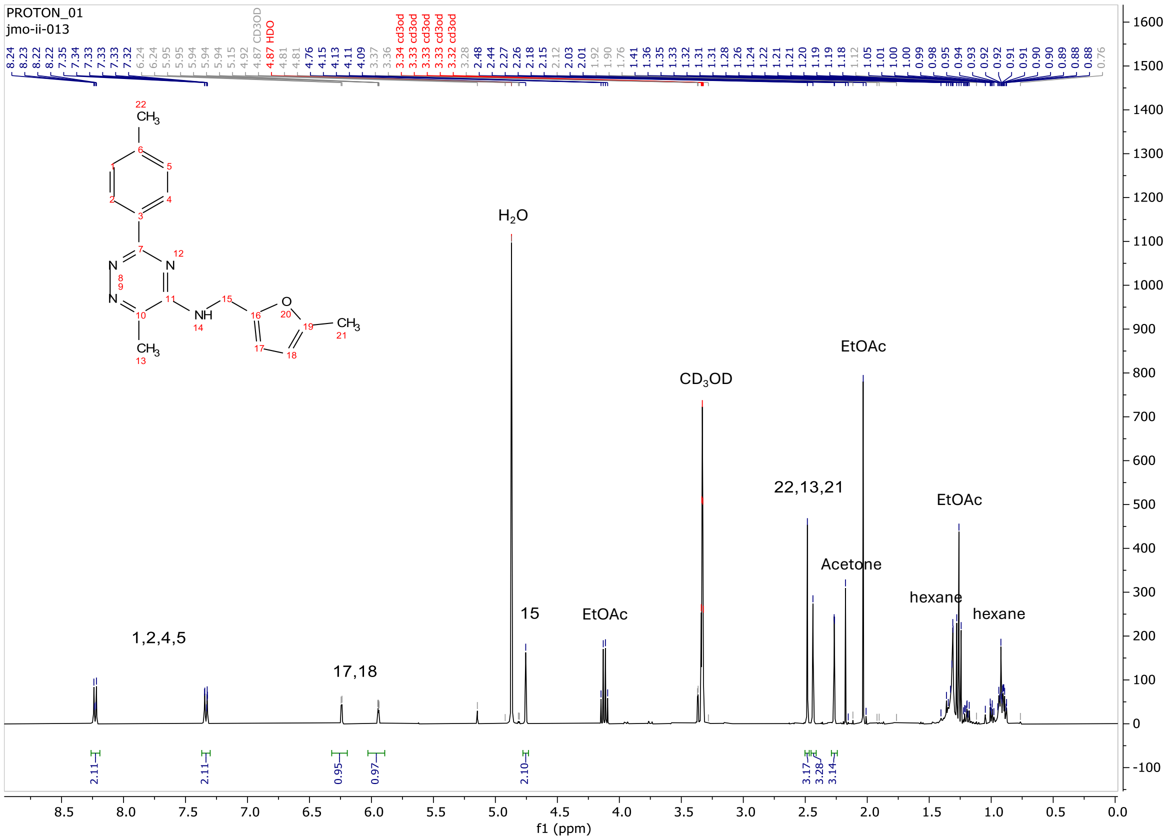

^{13}C NMR (101 MHz, CD_3OD)

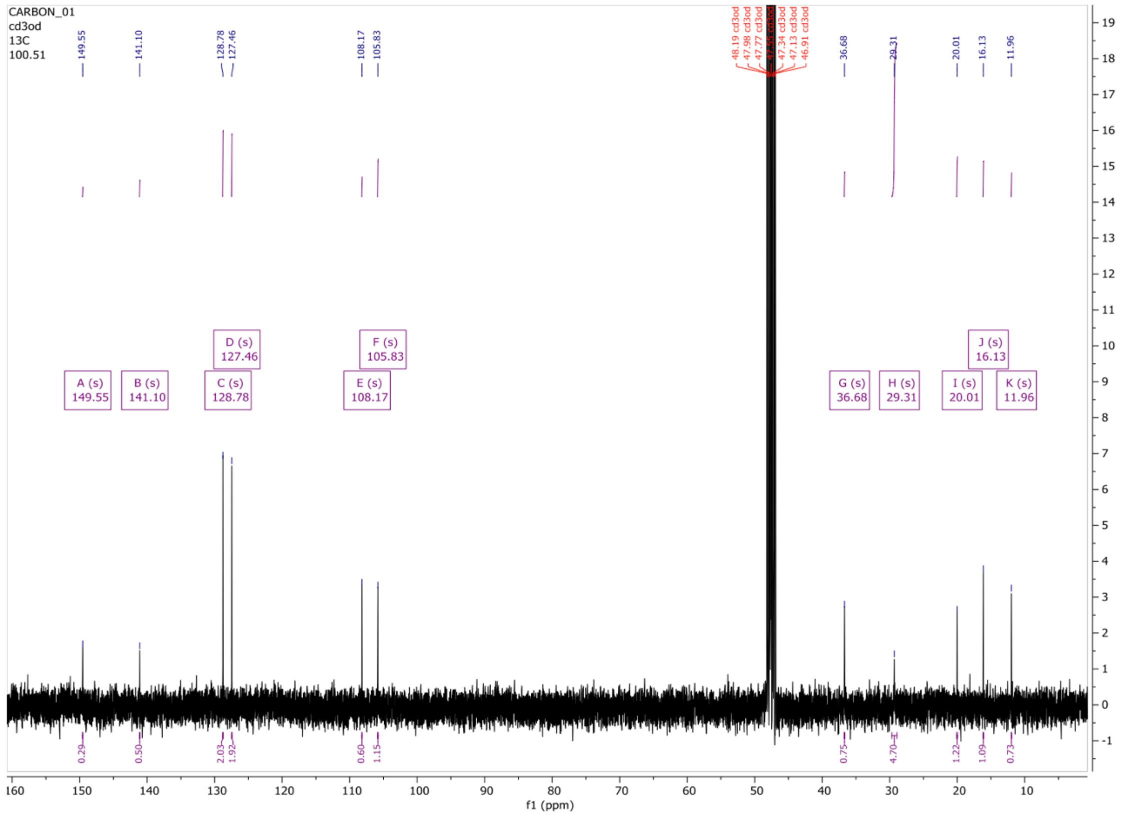

24k

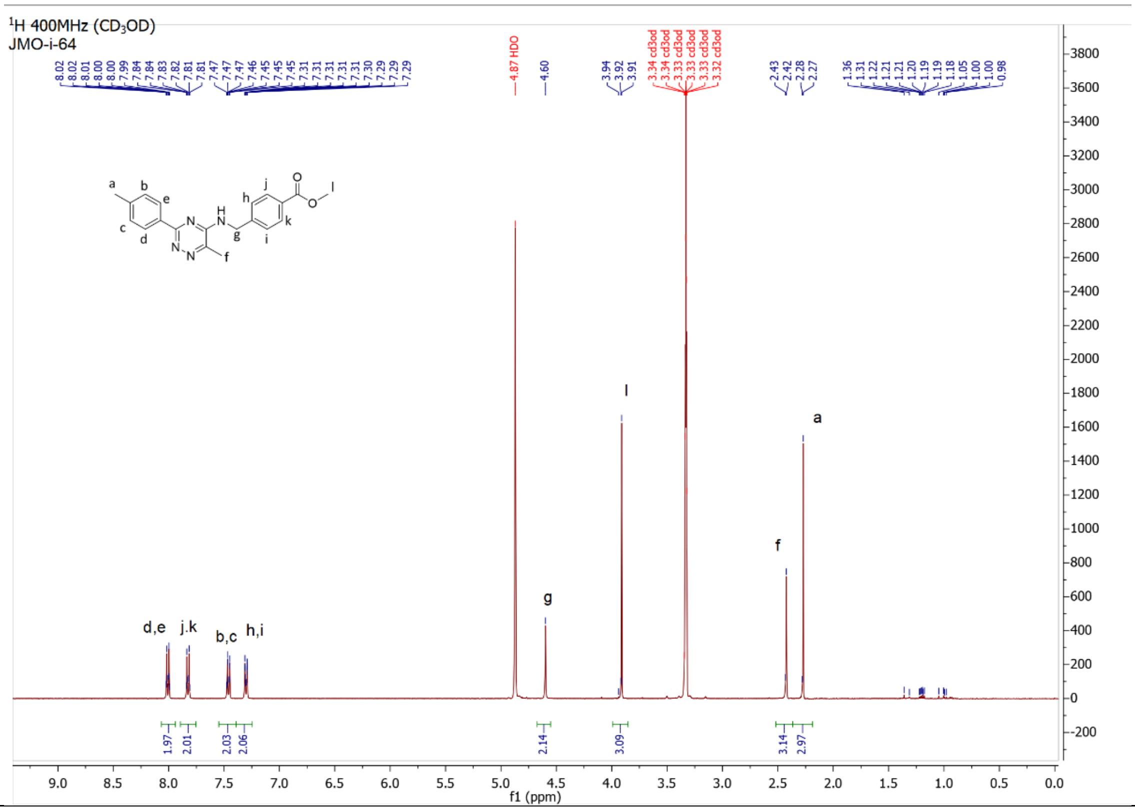

1H 400MHz (CD3OD)
JMO-i-64
d,e
j,k
b,c
h,i
g
l
f
a
f1 (ppm)

24l

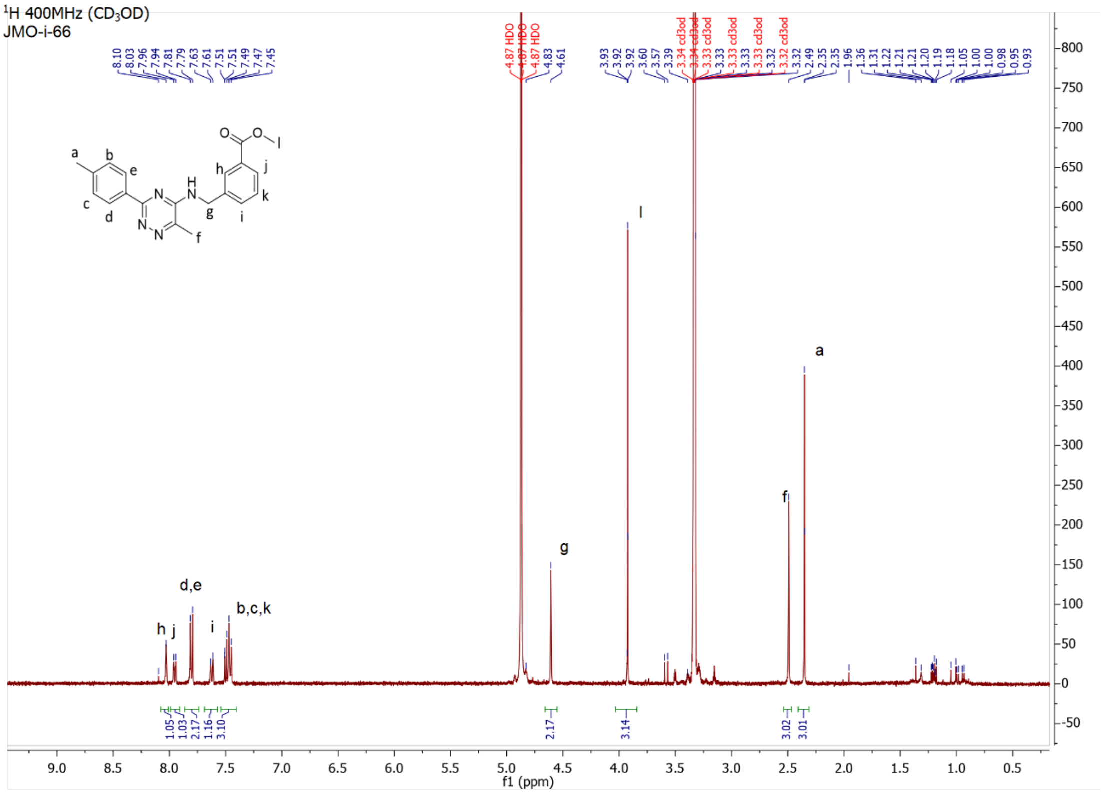

1H 400MHz (CD3OD)
JMO-i-66
h j
d,e
i
b,c,k
g
l
f
a
f1 (ppm)

24m

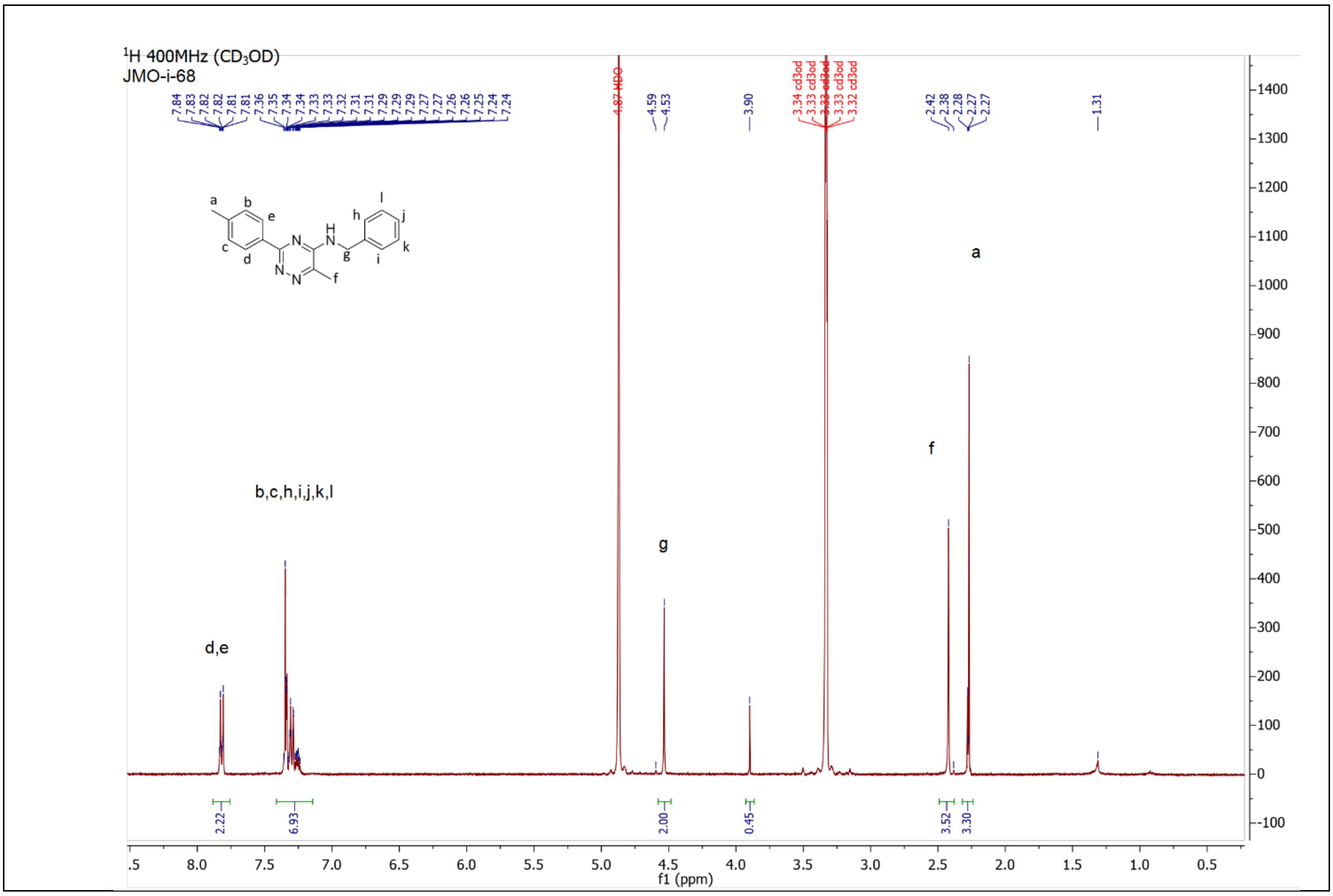

^{1}H 400MHz (CD_3OD)
JMO-i-68
d,e
b,c,h,i,j,k,l
g
f
a
f1 (ppm)

Data availability for commercially purchased compounds.

Compound	ChemDiv ID	Available Data		Compound	ChemDiv ID	Available Data	
		LC/MS	NMR			LC/MS	NMR
**1**	S340-0346	N	Y	**28**	P895-0088	Y	Y
**2**	G433-0451	N	Y	**29**	M290-1030	Y	N
**3**	J104-0018	Y	Y	**30**	L485-2714	Y	Y
**4**	S058-0164	Y	Y	**31**	P221-0105	Y	N
**5**	M290-1113	Y	N	**32**	F972-0214	N	Y
**6**	M058-0692F	Y	N	**33**	L970-0124	Y	Y
**7**	S058-0002	Y	Y	**34**	F972-0097	Y	Y
**8**	G433-0624	N	Y	**35**	M337-0030	Y	Y
**9**	S062-1442	Y	Y	**36**	S063-0016	Y	Y
**10**	P006-0040	Y	Y	**37**	M337-0343	Y	Y
**11**	F972-0247	N	Y	**38**	P169-2258	Y	Y
**12**	M058-0019	N	Y	**39**	L485-2700	Y	Y
**13**	G582-3119	N	Y	**40**	D588-0063	Y	Y
**14**	S062-1309	Y	Y	**41**	G811-1254	Y	Y
**15**	P077-0019	Y	N	**42**	S338-1010	Y	Y
**16**	M290-1115	Y	N	**43**	P804-0861	Y	Y
**17**	P076-0778c	Y	N	**44**	F972-0105	Y	Y
**18**	P076-0602d	Y	Y	**45**	M337-0025	Y	Y
**19**	F972-0325	N	Y	**46**	D622-0107	Y	Y
**20**	P077-0116	Y	N	**47**	S051-0072	Y	N
**21**	F926-1884	N	Y	**48**	M337-0418	Y	Y
**22**	F972-0224	Y	Y	**49**	F972-0045	Y	Y
**23**	F972-0249	Y	Y	**50**	P006-0346	Y	Y
**24**	P006-0394	Y	Y	**51**	D600-0244	Y	Y
**24a**	P006-0334	Y	Y	**52**	F972-0103	N	Y
**24b**	P006-0601	Y	Y	**53**	S333-0330	Y	Y
**24c**	P006-0409	Y	Y	**54**	M337-0141	Y	Y
**24d**	P006-0676	Y	Y	**55**	M976-0157	Y	Y
**24e**	P006-0040	Y	Y	**56**	N124-0015	Y	N
**24f**	P006-0381F	Y	N	**57**	P165-2563	Y	Y
**24g**	P006-1485	N	Y	**58**	G361-0372	N	Y
**24h**	P006-0376	Y	N	**59**	P018-0789	Y	Y
**24i**	P006-0533	Y	Y	**60**	D667-0063	Y	Y
**24j**	P006-0800	Y	Y	**61**	S333-0480	Y	Y
**25**	S063-0006	Y	Y	**62**	M321-0040	Y	Y
**26**	F972-0129	Y	Y	**63**	P018-1139	Y	Y
**27**	F972-0296	Y	Y				

Supplier-provided LC/MS data for commercially purchased compounds.

Compound: **3**	ChemDiv ID: J104-0018

C26 H31 N3 O4
M.W.=449.55

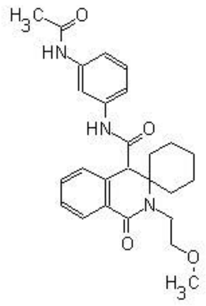

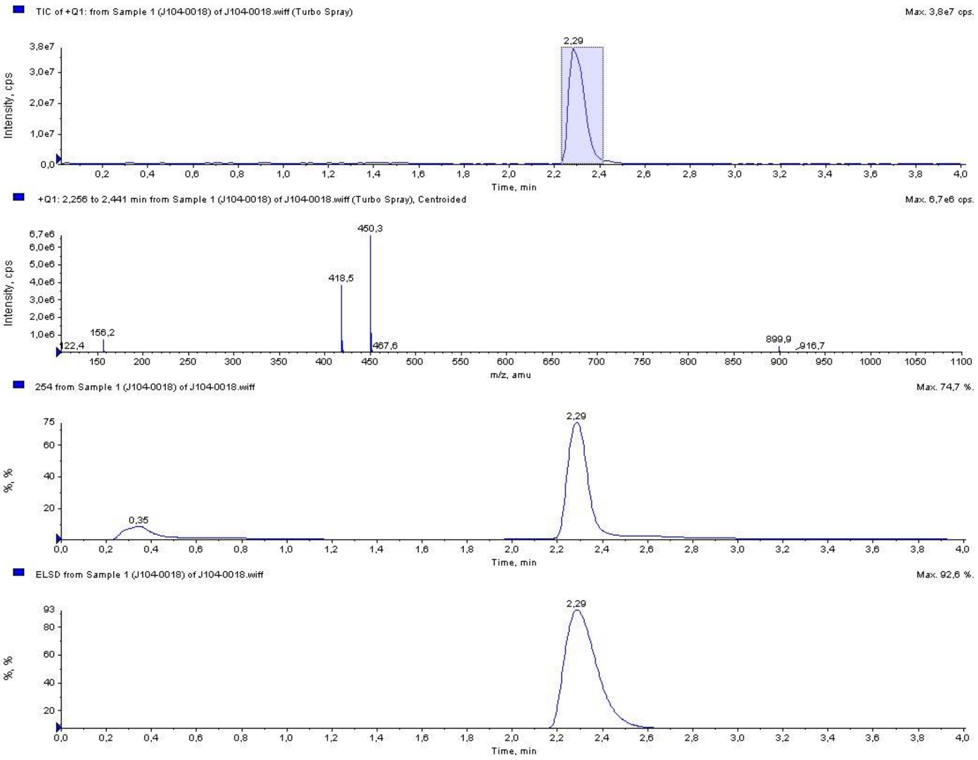

Compound: **4**	ChemDiv ID: S058-0164

C18 H21 F N4 O2
M.W.=344.39

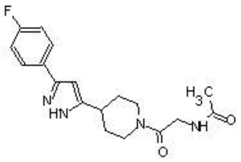

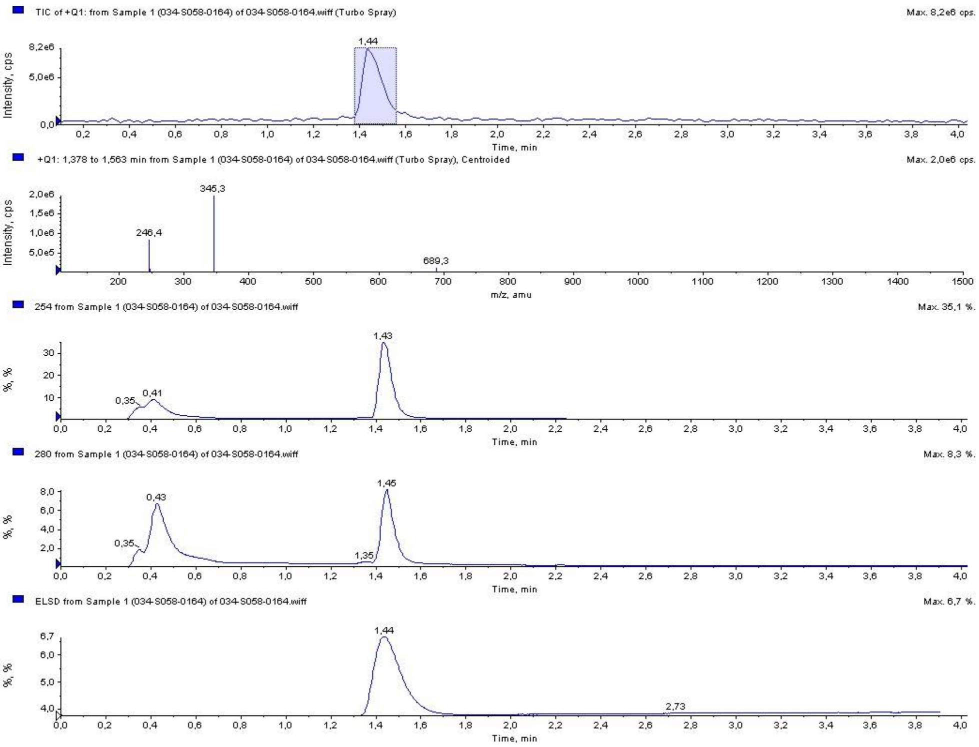

Compound: **5** ChemDiv ID: M290-1113

C26 H31 N5 O2
M.W.=445.56

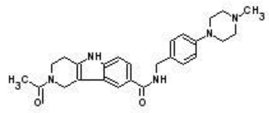

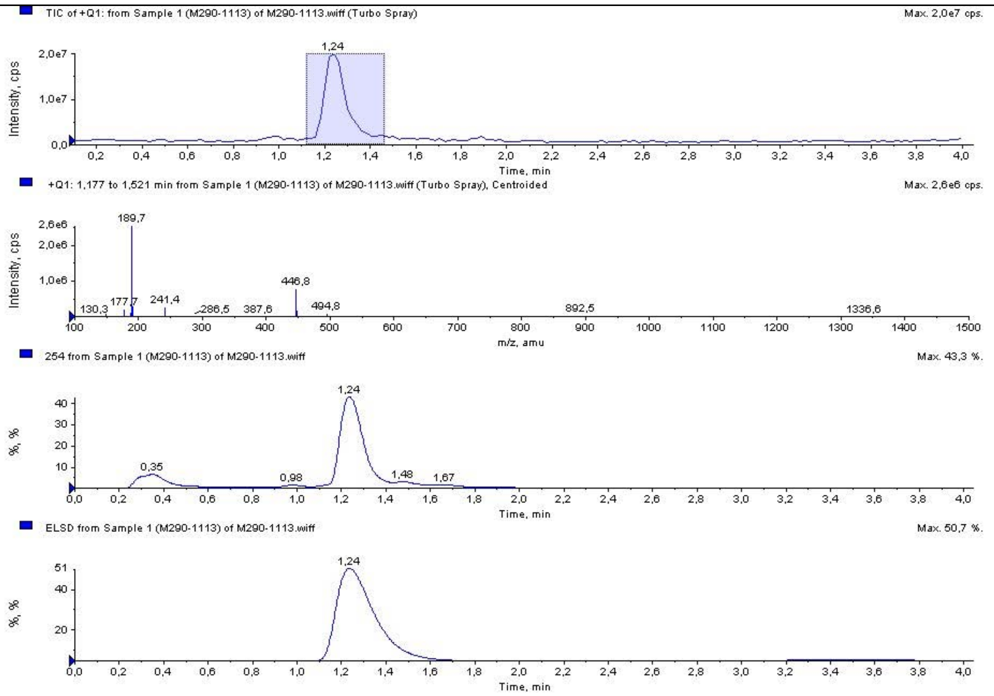

Compound: **6** ChemDiv ID: M058-0692F

C19 H22 N4 O2
M.W.=338.41

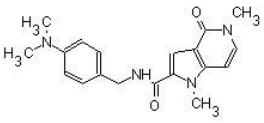

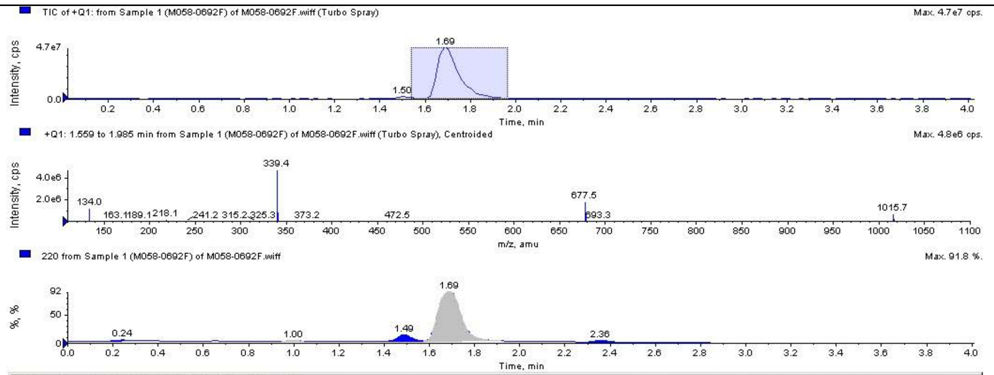

220 from Sample 1 (M058-0692F) of M058-0692F.wiff

Data List | Peak List

	Time (min)	Area (counts)	% Area	Height (%)	% Height	Width (min)	Baseline Type
4	1.6896	614.0542	90.3324	87.1858	84.0549	0.3433	Valley
3	1.4874	46.4710	6.8363	11.2242	10.8211	0.1700	Valley
5	2.3571	12.8737	1.8938	3.2093	3.0941	0.1533	Base to Base

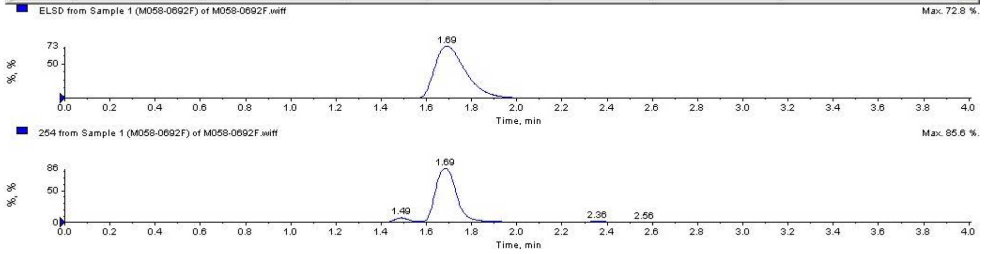

Compound: **7** ChemDiv ID: S058-0002

C19 H19 N3 O2
M.W.=321.38

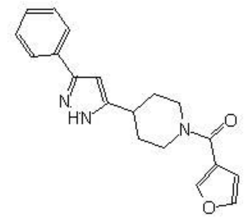

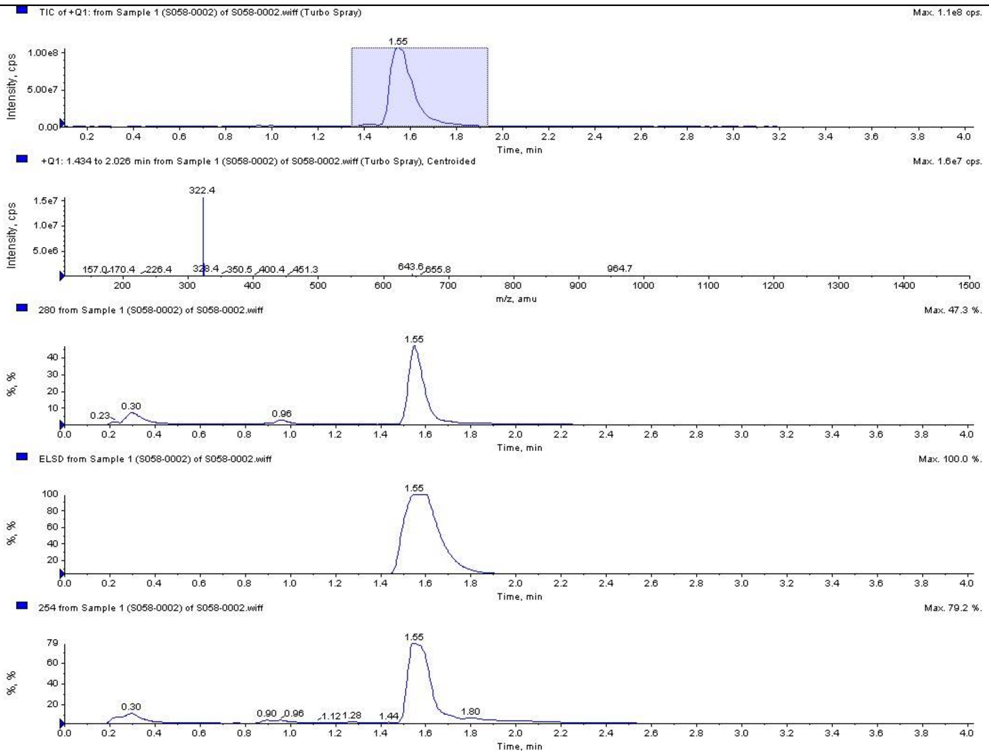

Compound: **9** ChemDiv ID: S062-1442

C18 H18 N4 O2 S
M.W.=354.43

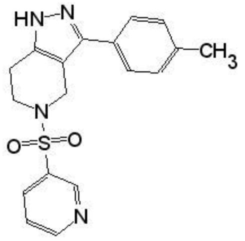

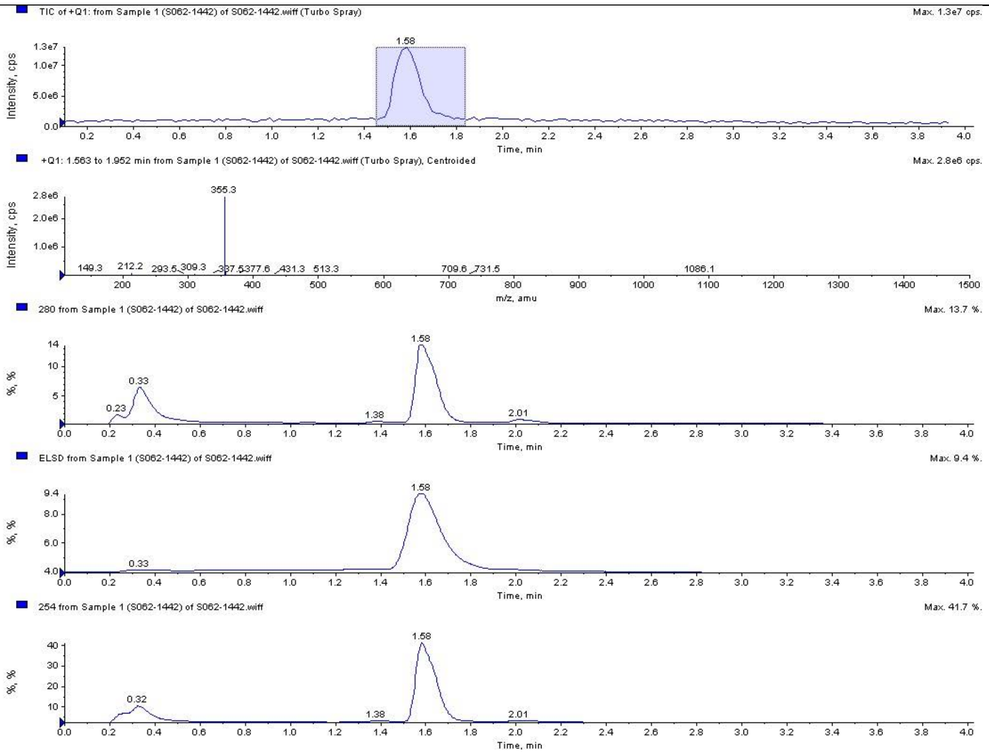

Compound: **10** (=**24e**)	ChemDiv ID: P006-0040

C18 H18 N4 O
M.W.=306.37

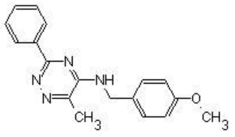

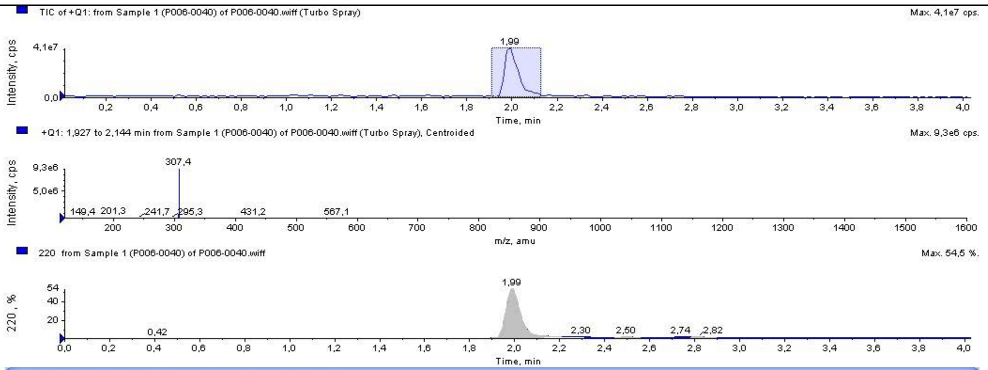

220 from Sample 1 (P006-0040) of P006-0040.wiff

Data List | Peak List

	Time (min)	Area (counts)	% Area	Height (%)	% Height	Width (min)	Baseline Type
2	1,9885	241,1585	95,1984	53,1817	94,2787	0,3133	Base to Base
5	2,7400	4,3945	1,7347	1,1622	2,0604	0,1033	Valley
6	2,8201	3,9122	1,5444	0,9614	1,7043	0,1133	Valley

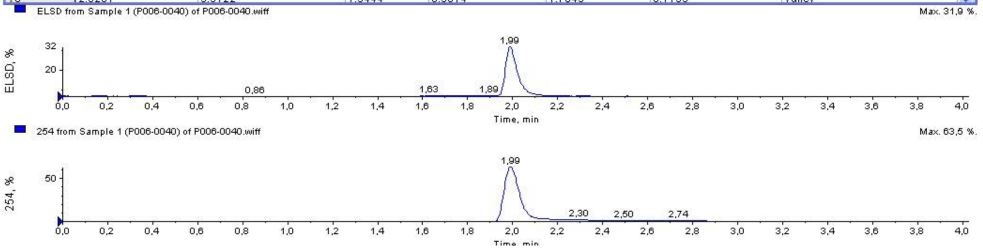

Compound: **14**	ChemDiv ID: S062-1309

C18 H17 N3 O2
M.W.=307.35

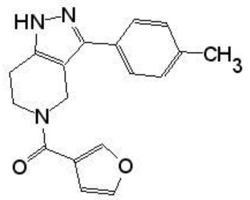

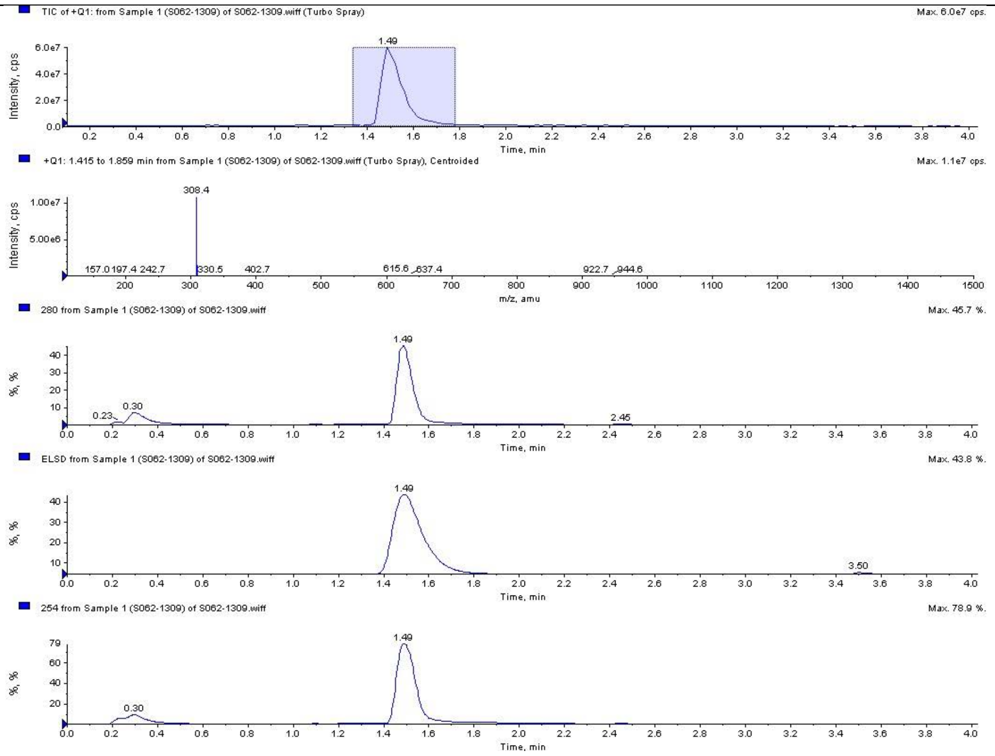

Compound: **15** ChemDiv ID: P077-0019

C22 H20 N4 O3
M.W.=388.43

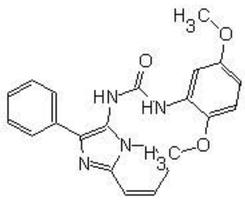

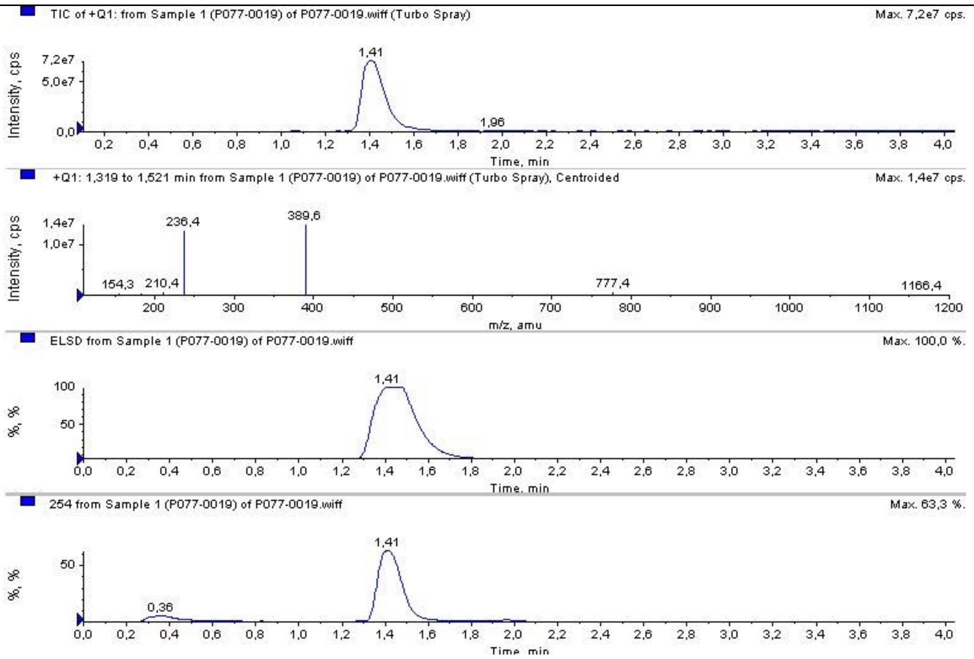

Compound: **16** ChemDiv ID: M290-1115

C25 H28 N4 O2
M.W.=416.52

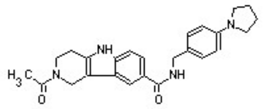

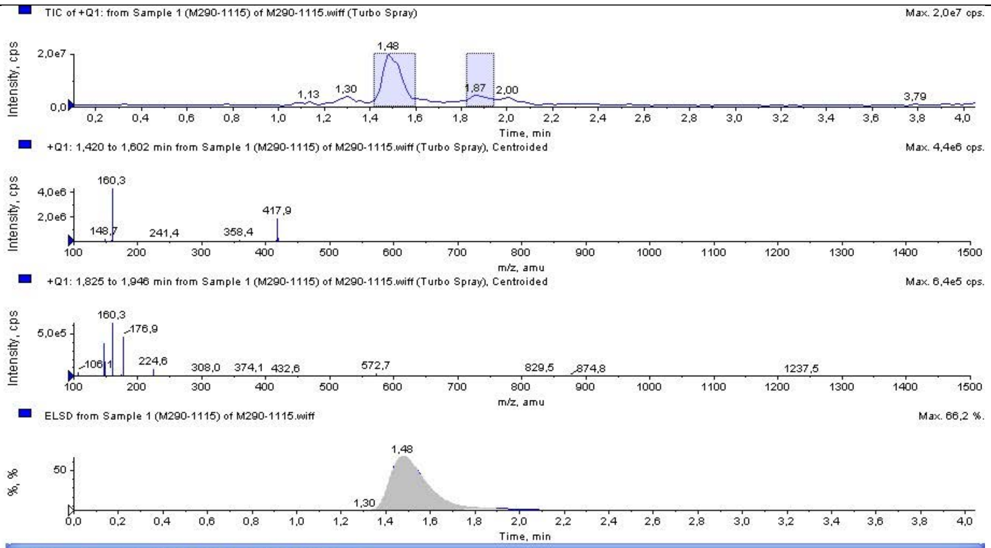

ELSD from Sample 1 (M290-1115) of M290-1115.wiff

Data List | Peak List

	Time (min)	Area (counts)	% Area	Height (%)	% Height	Width (min)	Baseline Type
1	1,3039	1,7477	0,2579	0,3258	0,5349	0,1267	Valley
2	1,4774	675,8227	99,7421	60,5867	99,4651	0,5733	Valley

Compound: **17**	ChemDiv ID: P076-0778c

C24 H23 N3 O2
M.W.=385.47

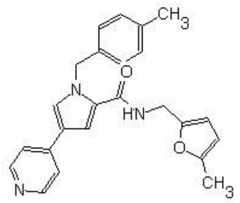

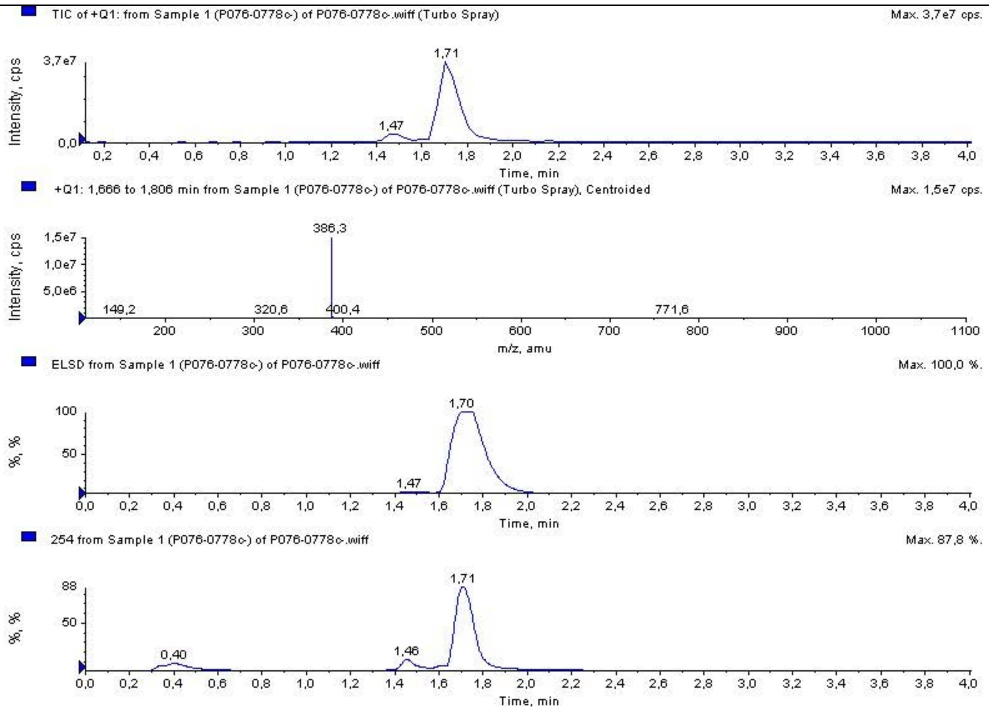

Compound: **18**	ChemDiv ID: P0776-0602d

C24 H23 N3 O2
M.W.=385.47

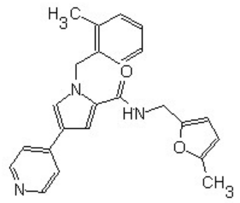

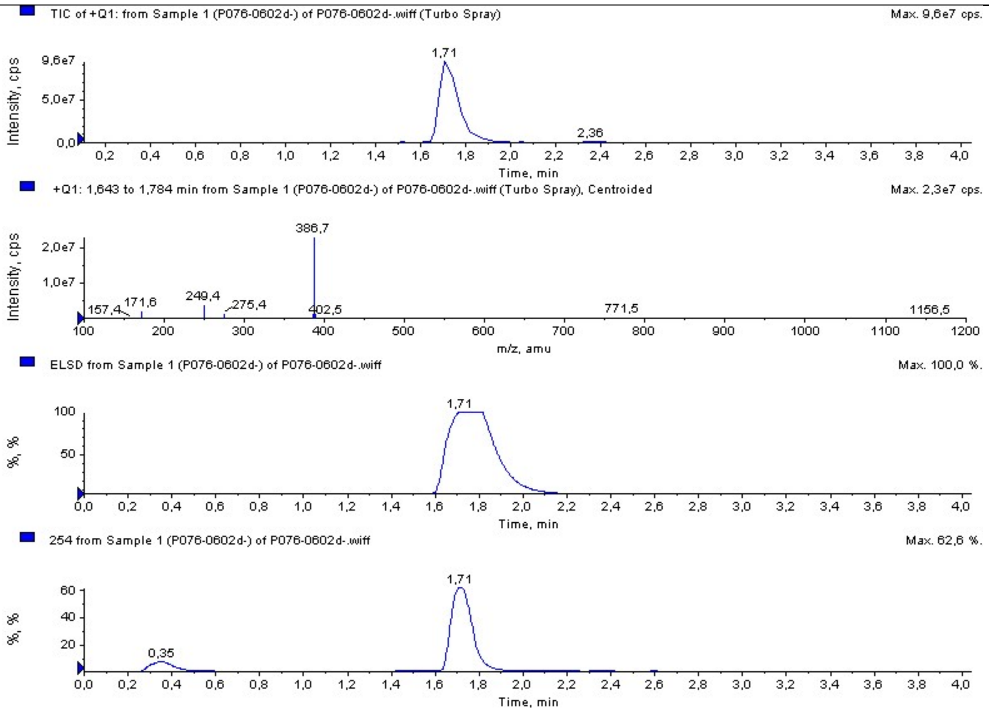

Compound: **20**	ChemDiv ID: P077-0116

C23 H22 N4 O3
M.W.=402.45

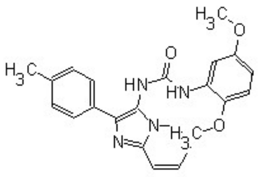

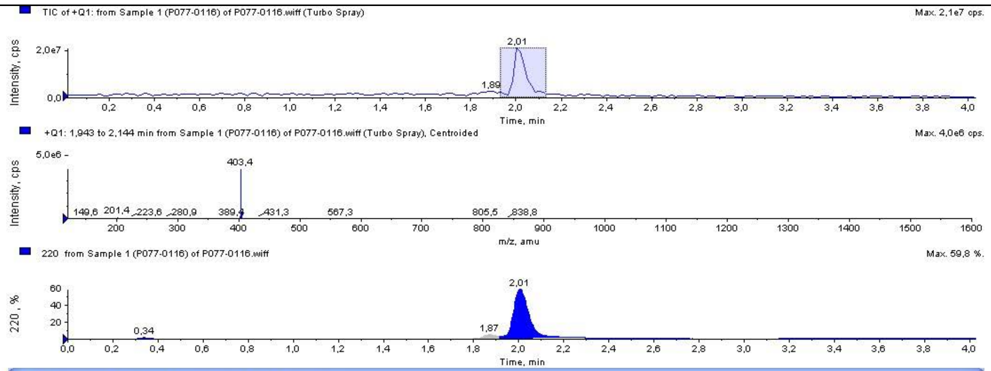

220 from Sample 1 (P077-0116) of P077-0116.wiff

Data List | Peak List

	Time (min)	Area (counts)	% Area	Height (%)	% Height	Width (min)	Baseline Type
3	2,0057	271,9611	91,8812	57,9897	90,1132	0,2967	Valley
2	1,8727	18,8954	6,3837	4,7402	7,3661	0,1433	Valley
1	0,3385	5,1356	1,7350	1,6221	2,5207	0,1167	Base to Base

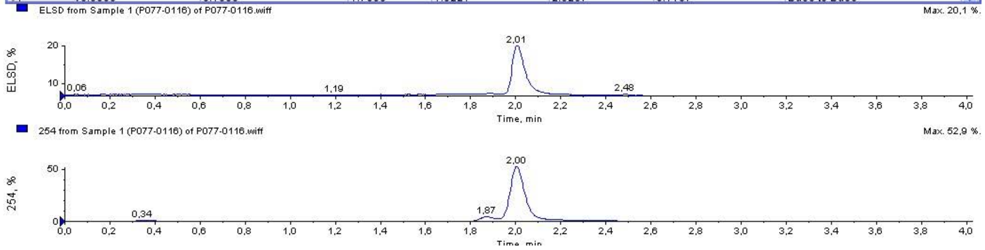

Compound: **22**	ChemDiv ID: F972-0224

C26 H24 N4 O4
M.W.=456.50

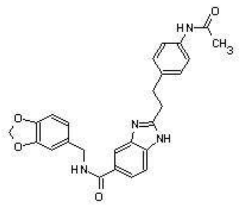

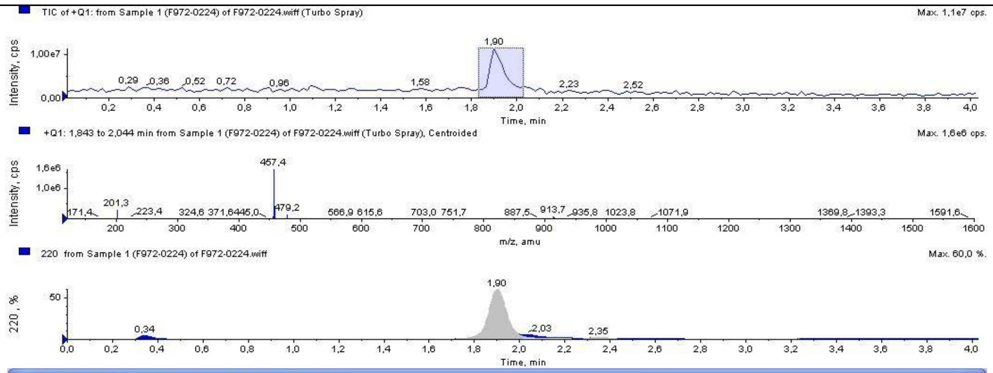

220 from Sample 1 (F972-0224) of F972-0224.wiff

Data List | Peak List

	Time (min)	Area (counts)	% Area	Height (%)	% Height	Width (min)	Baseline Type
2	1,9007	335,3140	90,9466	58,2635	87,7025	0,3333	Expo. Skim
1	0,3431	19,3463	5,2473	4,7374	7,1311	0,1933	Base to Base
3	2,0347	8,8408	2,3979	2,1422	3,2245	0,1367	Expo. Daughter

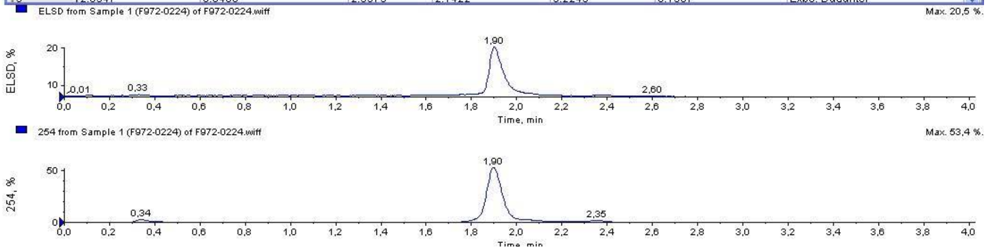

Compound: **23** ChemDiv ID: F972-0249

C23 H28 N4 O2
M.W.=392.50

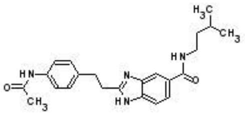

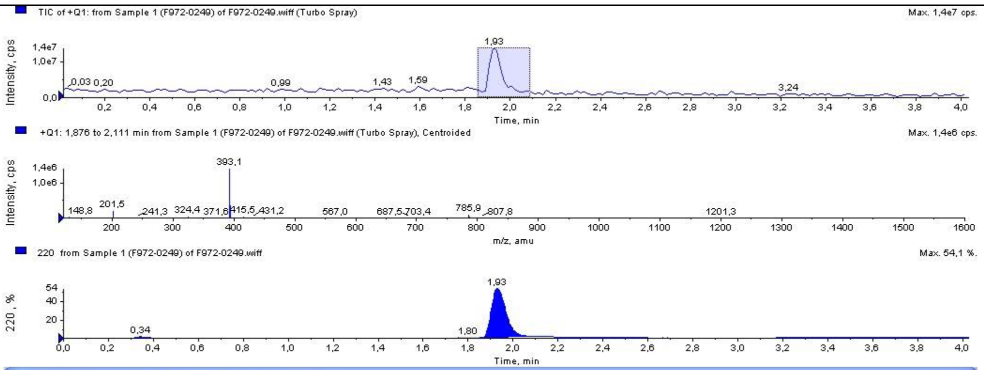

220 from Sample 1 (F972-0249) of F972-0249.wiff

Data List | Peak List

	Time (min)	Area (counts)	% Area	Height (%)	% Height	Width (min)	Baseline Type
3	1,9311	235,1120	97,0909	52,7042	96,2866	0,2400	Base to Base
1	0,3427	4,8790	2,0148	1,5344	2,8032	0,1167	Base to Base
2	1,7997	2,1657	0,8943	0,4982	0,9102	0,1467	Base to Base

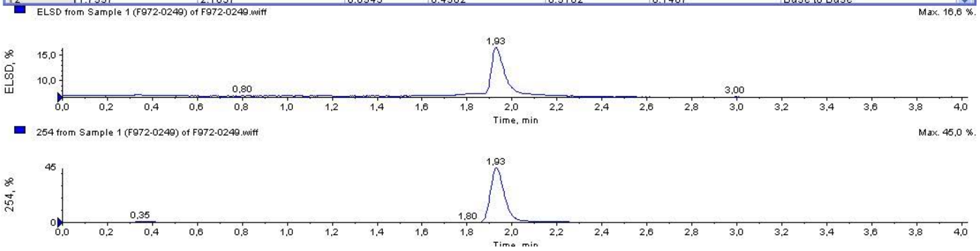

Compound: **24** ChemDiv ID: P006-0394

C16 H16 N4 S
M.W.=296.40

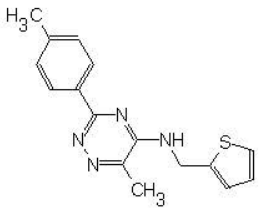

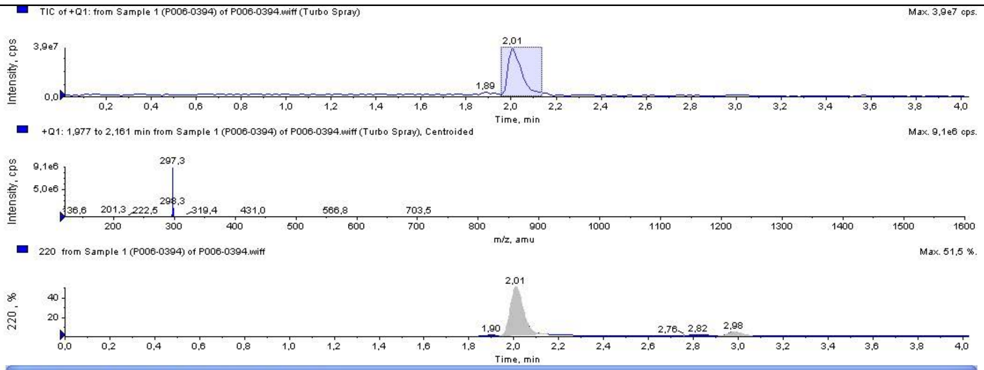

220 from Sample 1 (P006-0394) of P006-0394.wiff

Data List | Peak List

	Time (min)	Area (counts)	% Area	Height (%)	% Height	Width (min)	Baseline Type
2	2,0097	199,7446	90,7294	49,5142	89,8047	0,2100	Base to Base
4	2,9826	15,4902	7,0361	4,0681	7,3784	0,1500	Base to Base
3	2,8234	2,5553	1,1607	0,7886	1,4304	0,1033	Base to Base

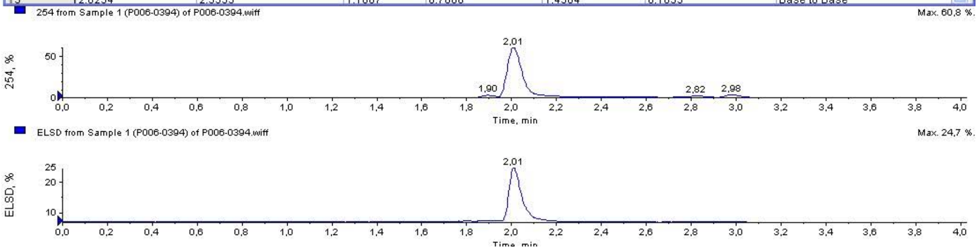

Compound: **24a**	ChemDiv ID: P006-0334

C16 H16 N4 O
M.W.=280.33

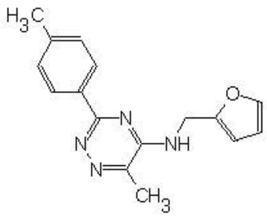

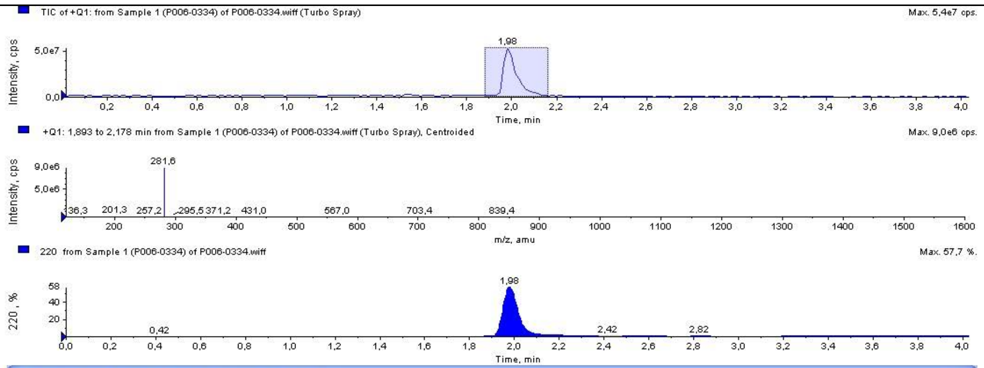

220 from Sample 1 (P006-0334) of P006-0334.wiff

Data List | Peak List

	Time (min)	Area (counts)	% Area	Height (%)	% Height	Width (min)	Baseline Type
3	1,9784	271,8049	98,9167	56,6673	98,3254	0,4867	Base to Base
5	2,8244	0,9558	0,3478	0,2715	0,4711	0,1067	Base to Base
4	2,4192	0,8433	0,3069	0,2640	0,4582	0,0967	Base to Base

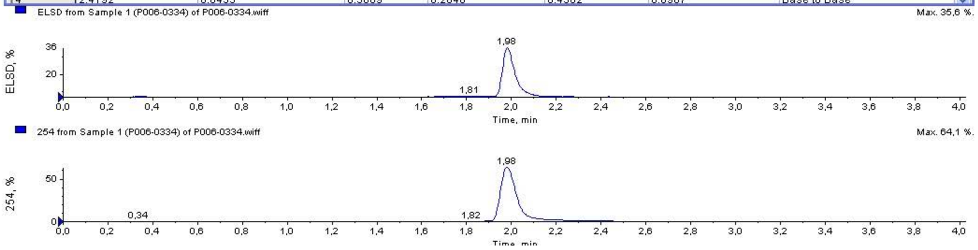

Compound: **24b**	ChemDiv ID: P006-0601

C16 H16 N4 O
M.W.=280.33

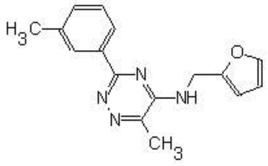

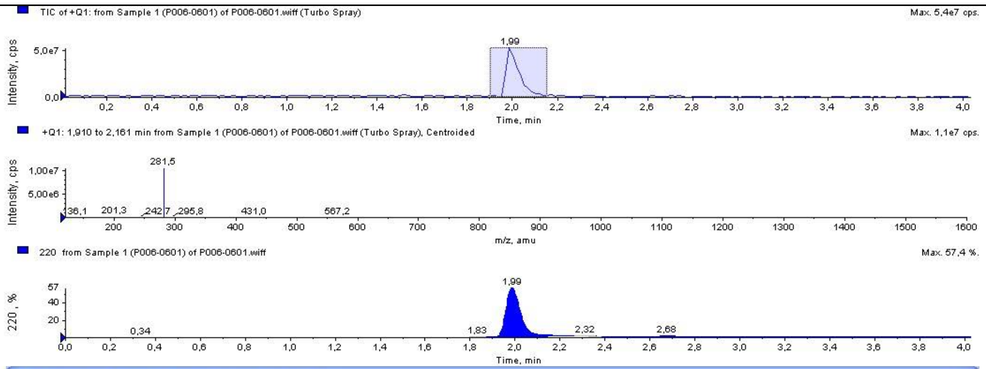

220 from Sample 1 (P006-0601) of P006-0601.wiff

Data List | Peak List

	Time (min)	Area (counts)	% Area	Height (%)	% Height	Width (min)	Baseline Type
3	1,9864	254,8005	96,3845	55,9853	95,5126	0,3667	Valley
5	2,6777	4,5063	1,7046	1,1467	1,9563	0,1433	Base to Base
2	1,8342	2,2846	0,8642	0,5489	0,9365	0,1233	Valley

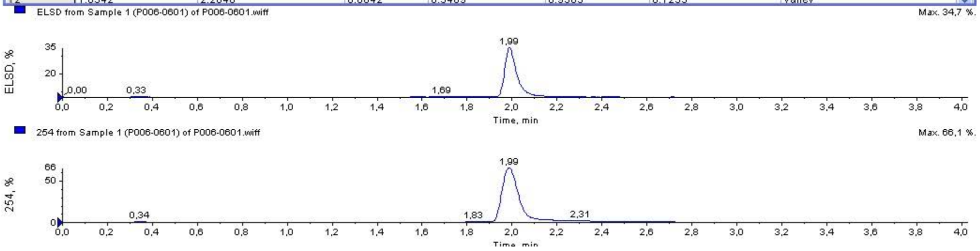

Compound: **24c** ChemDiv ID: P006-0409

C17 H18 N4 O
M.W.=294.36

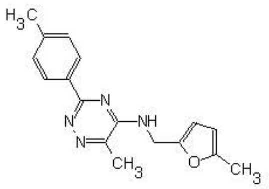

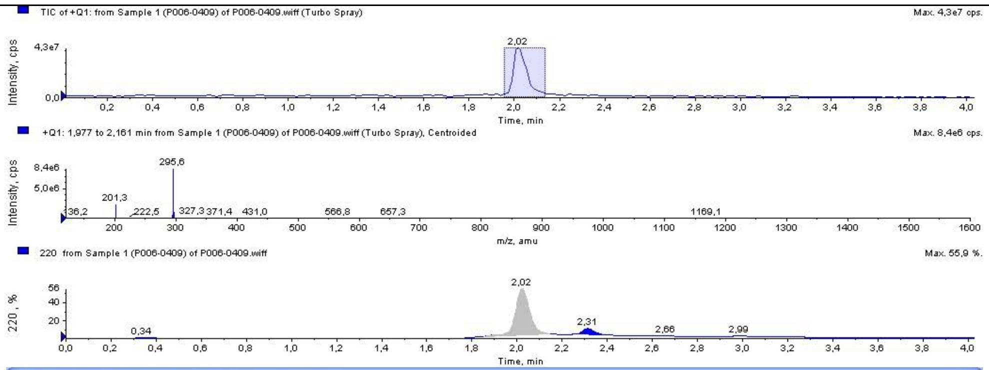

220 from Sample 1 (P006-0409) of P006-0409.wiff

Data List | Peak List

	Time (min)	Area (counts)	% Area	Height (%)	% Height	Width (min)	Baseline Type
2	2,0220	223,5451	90,3997	51,4279	87,8146	0,2900	Base to Base
3	2,3139	22,3669	9,0450	6,4961	11,0922	0,1333	Base to Base
1	0,3377	1,3733	0,5553	0,6402	1,0932	0,0600	Base to Base

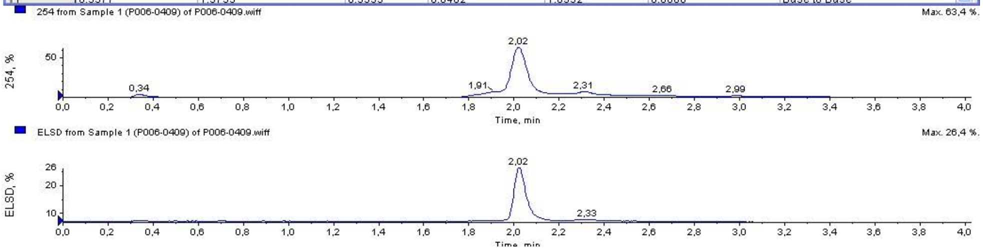

Compound: **24d** ChemDiv ID: P006-0676

C17 H18 N4 O
M.W.=294.36

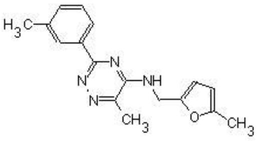

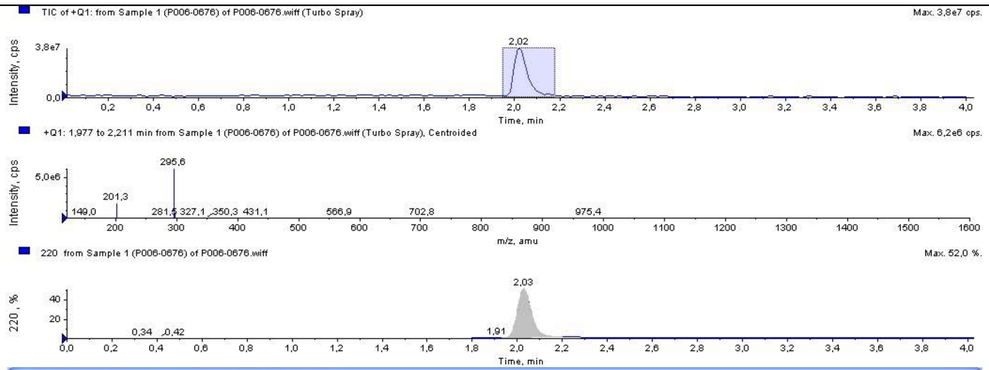

220 from Sample 1 (P006-0676) of P006-0676.wiff

Data List | Peak List

	Time (min)	Area (counts)	% Area	Height (%)	% Height	Width (min)	Baseline Type
4	2,0288	217,7850	97,5140	50,3684	97,7058	0,2667	Valley
3	1,9106	2,3426	1,0489	0,2305	0,4472	0,1733	Valley
1	0,3357	1,8817	0,8425	0,5852	1,1351	0,1067	Valley

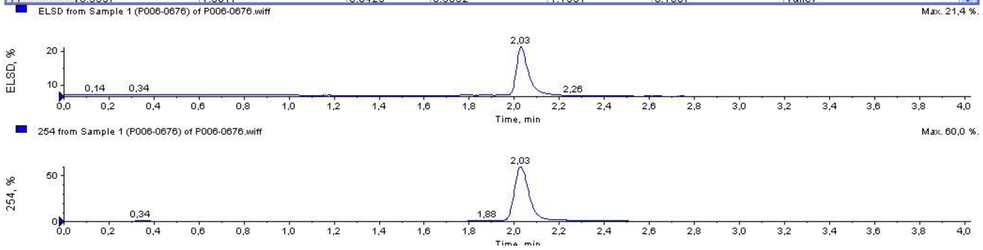

Compound: **24e**<br>(See Compound **10**)	ChemDiv ID: P006-0040

Compound: **24f**	ChemDiv ID: P006-0381F

C19 H20 N4 O
M.W.=320.39

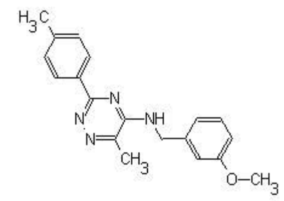

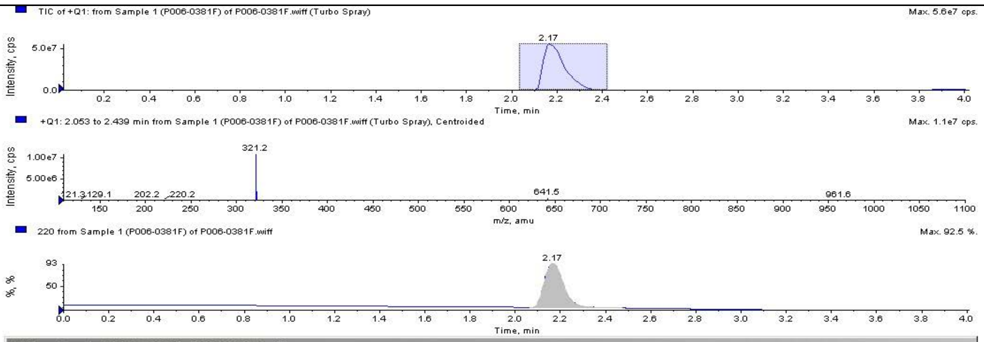

220 from Sample 1 (P006-0381F) of P006-0381F.wiff

Data List | Peak List

	Time (min)	Area (counts)	% Area	Height (%)	% Height	Width (min)	Baseline Type
2	2.1673	487.3473	99.8344	81.7714	99.6045	0.4067	Base to Base
1	0.2320	0.8083	0.1656	0.3247	0.3955	0.0833	Base to Base

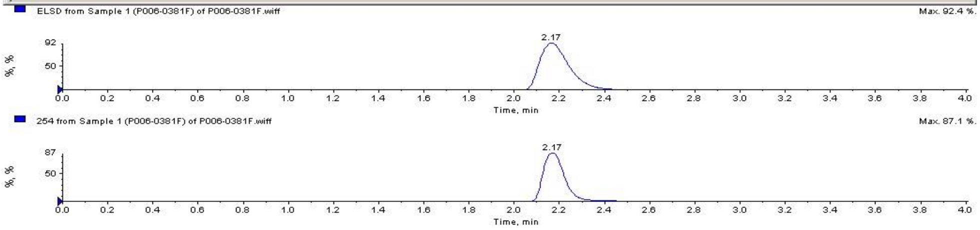

Compound: **24h**	ChemDiv ID: P006-0376

C14 H18 N4 O
M.W.=258.32

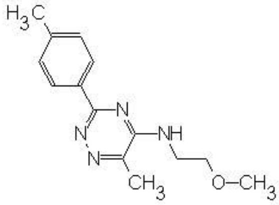

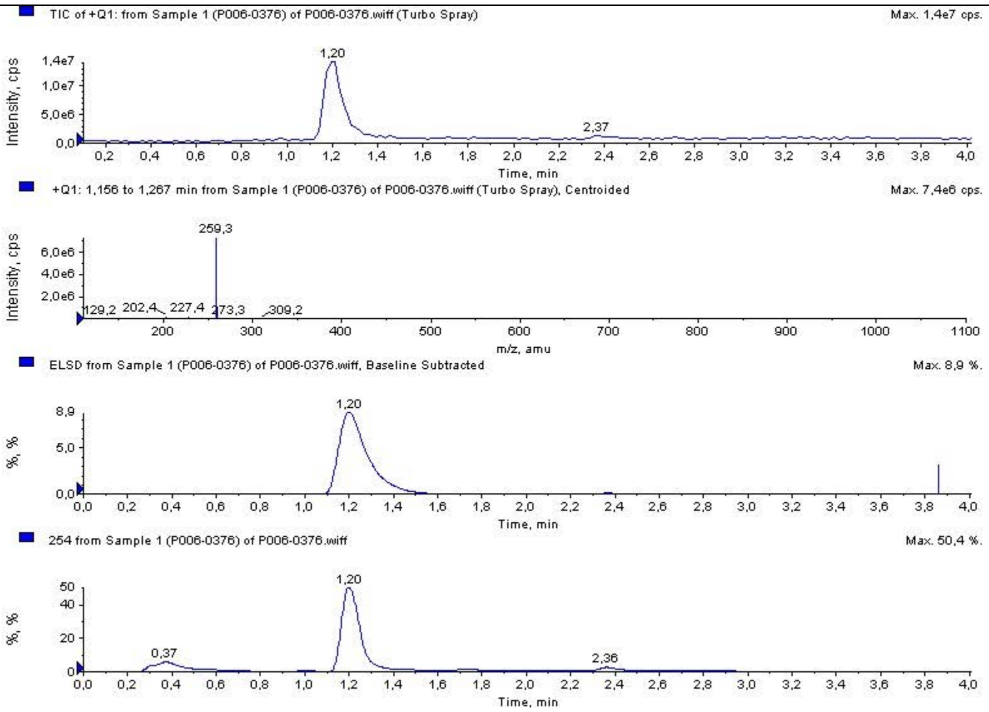

Compound: **24i** ChemDiv ID: P006-0533

C20 H22 N4 O2
M.W.=350.42

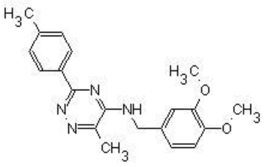

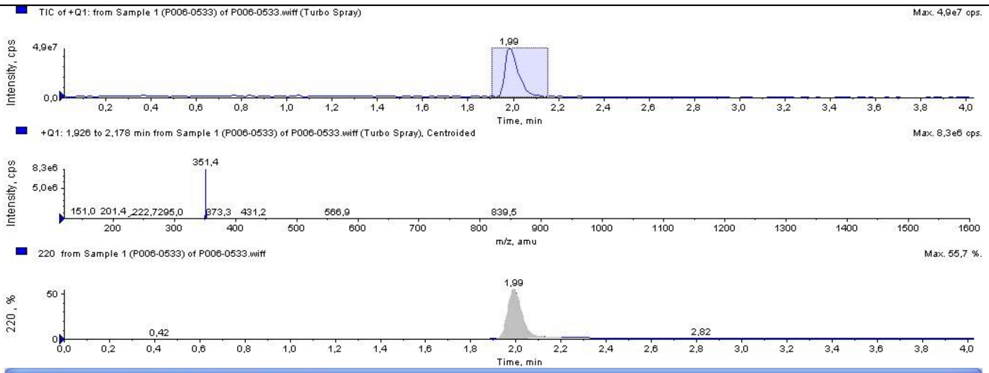

220 from Sample 1 (P006-0533) of P006-0533.wiff

Data List | Peak List

	Time (min)	Area (counts)	% Area	Height (%)	% Height	Width (min)	Baseline Type
2	1,9905	237,7187	98,9526	54,3908	98,7292	0,2833	Base to Base
1	0,4241	1,5923	0,6628	0,4166	0,7563	0,1100	Base to Base
3	2,8219	0,9240	0,3846	0,2835	0,5145	0,1067	Base to Base

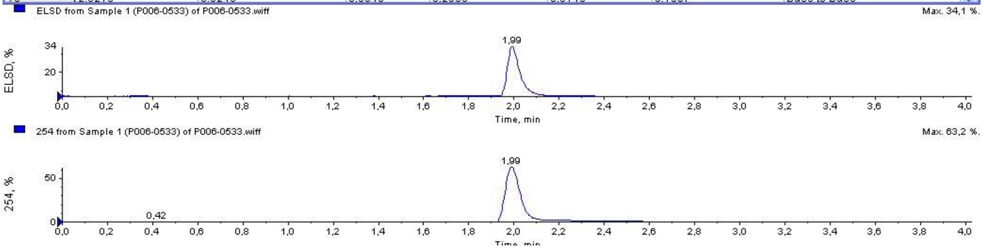

Compound: **24j** ChemDiv ID: P006-0800

C20 H22 N4 O2
M.W.=350.42

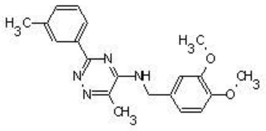

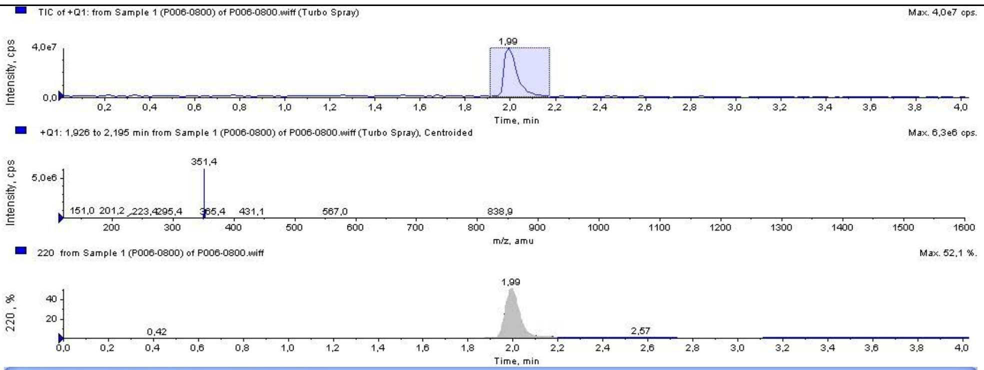

220 from Sample 1 (P006-0800) of P006-0800.wiff

Data List | Peak List

	Time (min)	Area (counts)	% Area	Height (%)	% Height	Width (min)	Baseline Type
2	1,9931	223,2473	99,3531	50,8965	99,0112	0,3633	Base to Base
1	0,4206	0,9241	0,4112	0,2973	0,5783	0,0900	Base to Base
3	2,5722	0,5296	0,2357	0,2111	0,4106	0,0800	Base to Base

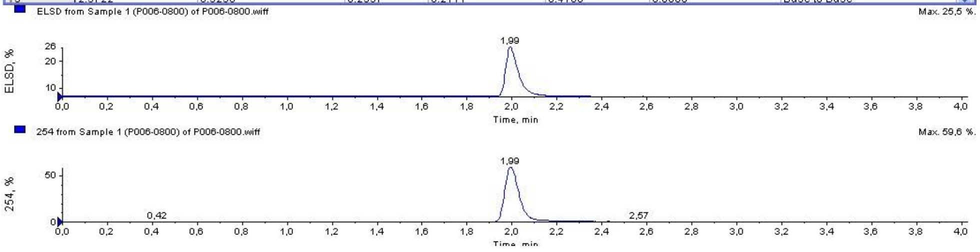

Compound: **25** ChemDiv ID: S063-0006

C15 H18 N4 O3
M.W.=302.33

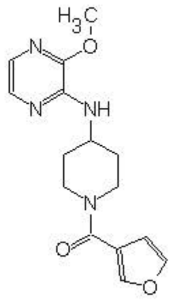

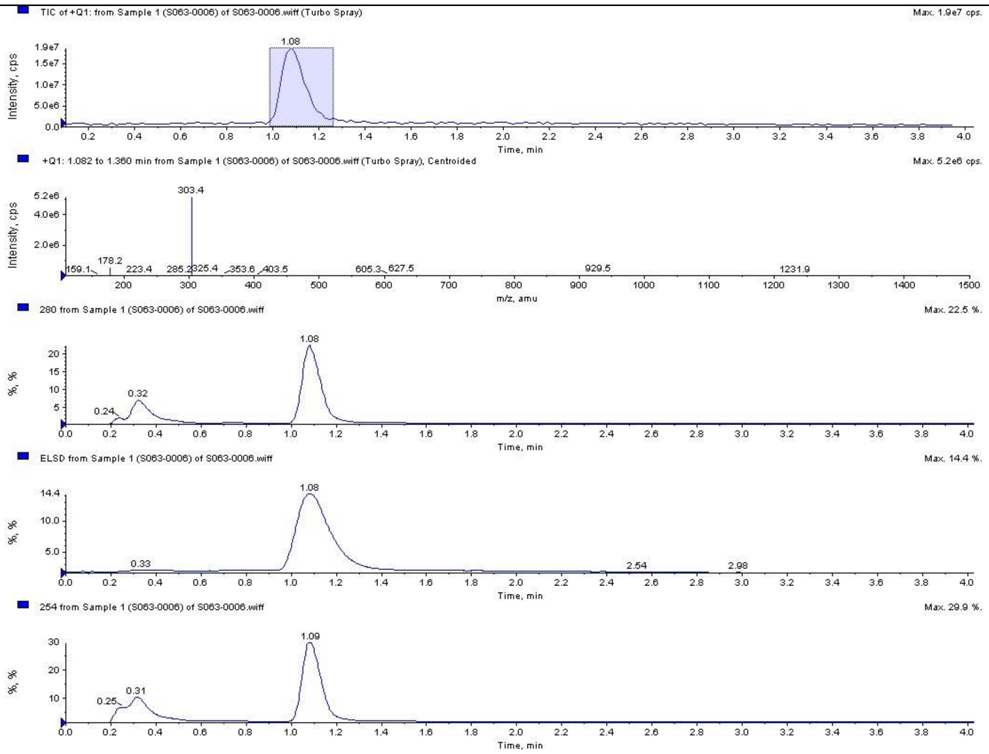

Compound: **26** ChemDiv ID: F972-0129

C26 H28 N4 O3
M.W.=444.53

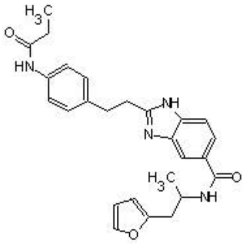

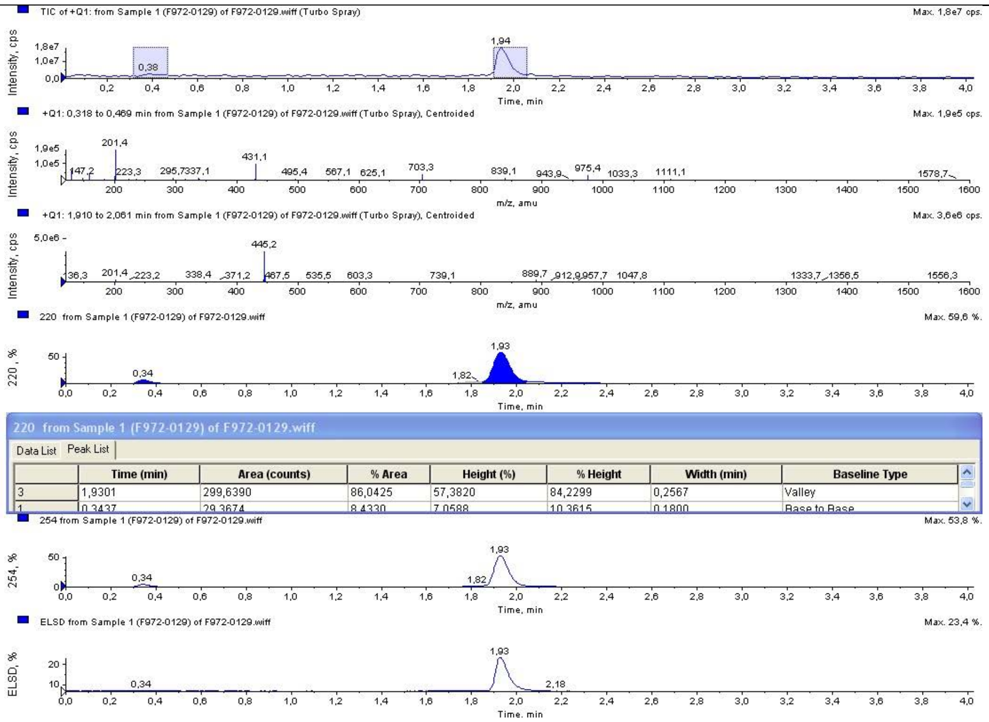

220 from Sample 1 (F972-0129) of F972-0129.wiff

Data List | Peak List

	Time (min)	Area (counts)	% Area	Height (%)	% Height	Width (min)	Baseline Type
3	1,9301	299,6390	86,0425	57,3820	84,2299	0,2567	Valley
1	0,3437	29,3674	8,4330	7,0588	10,3615	0,1800	Base to Base

Compound: **27**	ChemDiv ID: F972-0296

C24 H24 N4 O3

M.W.=416.48

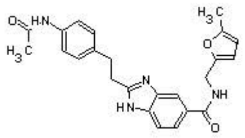

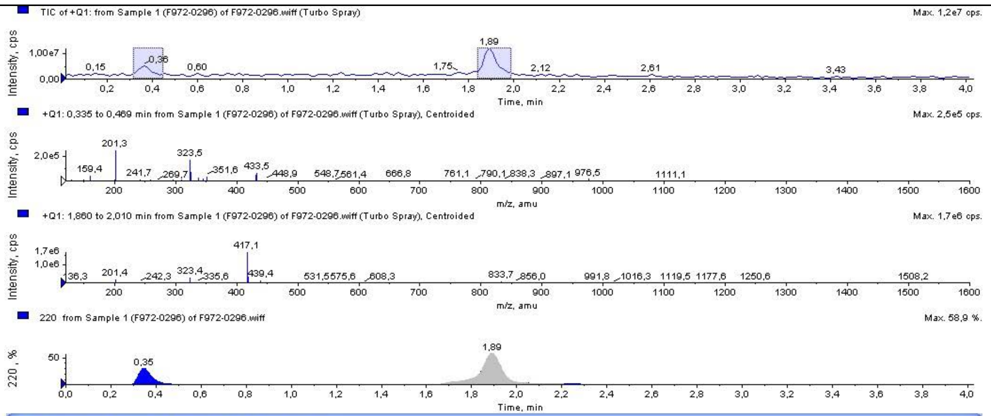

220 from Sample 1 (F972-0296) of F972-0296.wiff

Data List | Peak List

	Time (min)	Area (counts)	% Area	Height (%)	% Height	Width (min)	Baseline Type
2	1,8903	397,9948	75,5344	57,7447	65,4457	0,6000	Base to Base
1	0,3471	128,9108	24,4656	30,4883	34,5543	0,2667	Base to Base

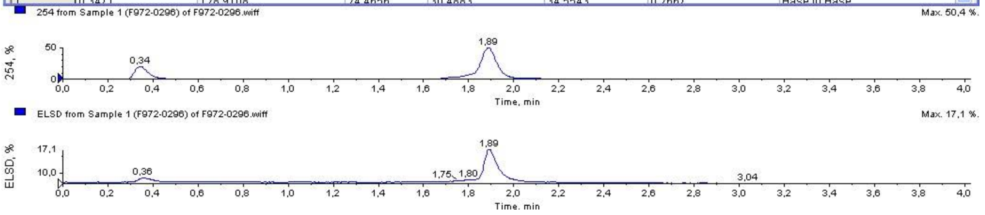

Compound: **28**	ChemDiv ID: P895-0088

C22 H19 N3 O4 S

M.W.=421.48

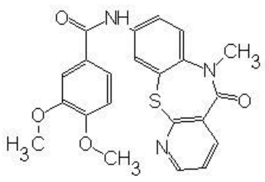

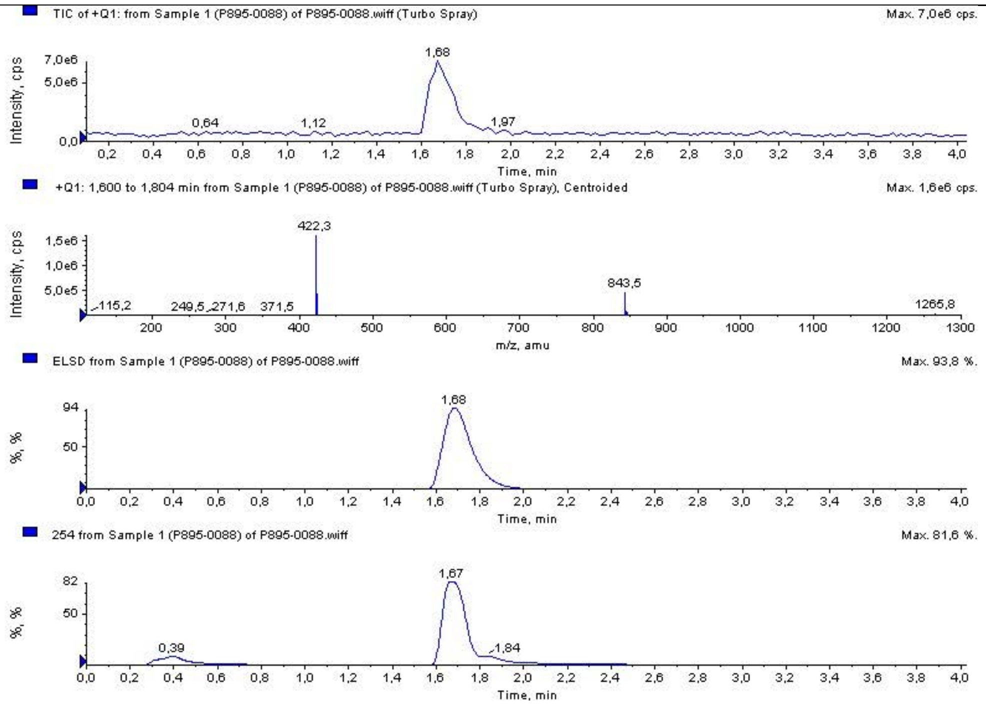

Compound: **29** ChemDiv ID: M290-1030

C24 H26 N4 O3
M.W.=418.50

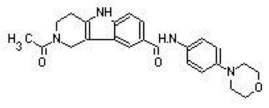

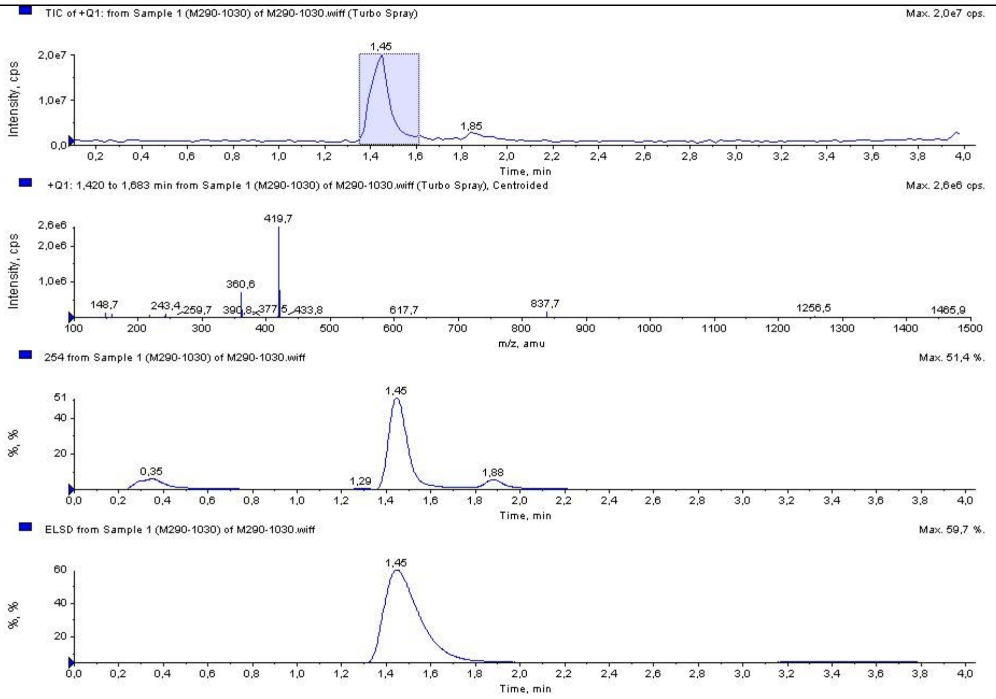

Compound: **30** ChemDiv ID: L485-2714

C23 H24 Br N3 O3
M.W.=470.37

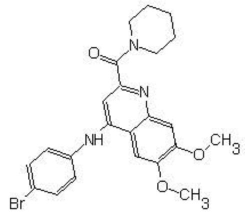

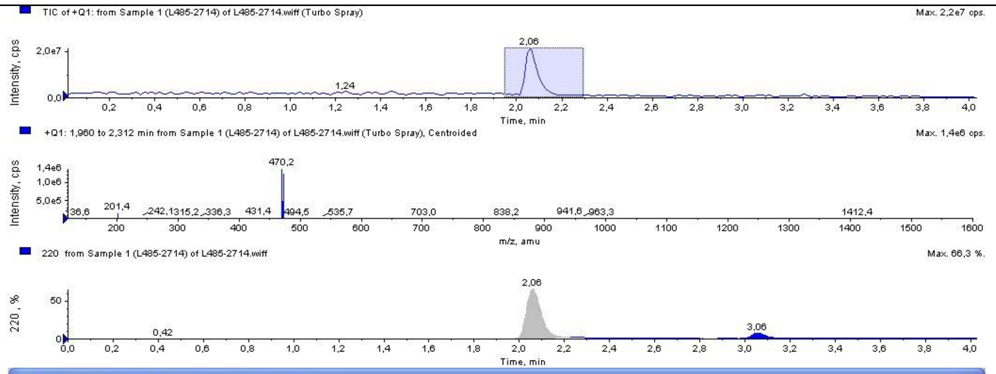

220 from Sample 1 (L485-2714) of L485-2714.wiff

Data List | Peak List

	Time (min)	Area (counts)	% Area	Height (%)	% Height	Width (min)	Baseline Type
2	2,0596	291,4956	91,0515	64,7948	90,2243	0,2600	Base to Base
3	3,0555	27,7947	8,6819	6,7794	9,4401	0,1800	Base to Base
1	0,4243	0,8533	0,2665	0,2410	0,3356	0,0867	Base to Base

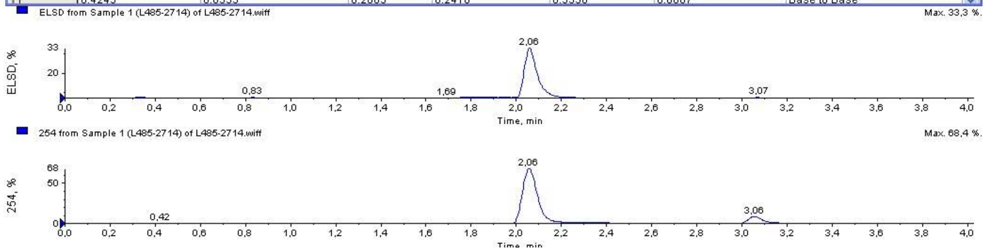

Compound: **31**	ChemDiv ID: P221-0105

C17 H15 Cl N4 O S
M.W.=358.85

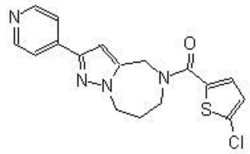

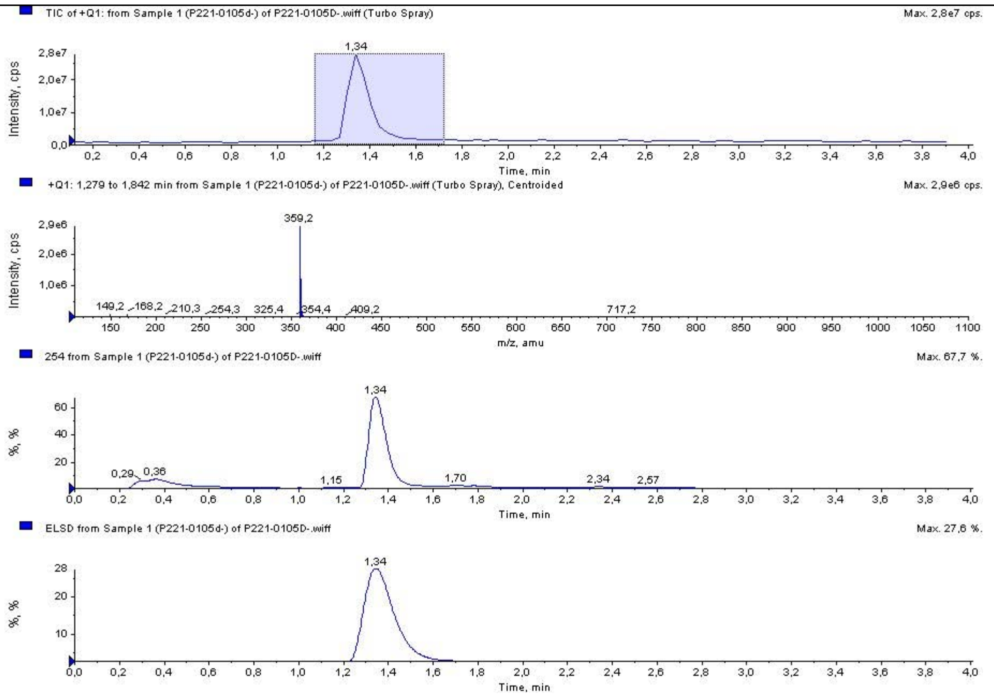

Compound: **33**	ChemDiv ID: L970-0124

C25 H23 N3 O4 S
M.W.=461.54

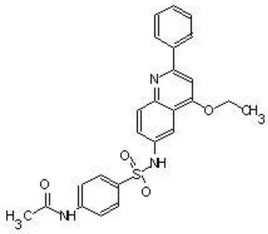

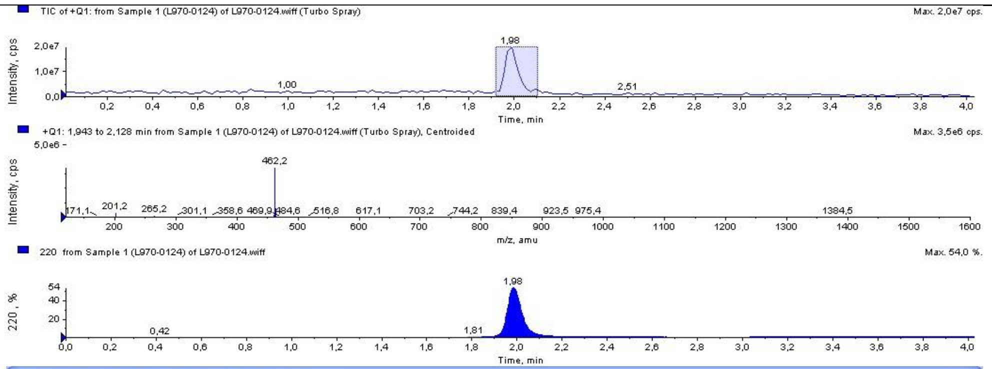

220 from Sample 1 (L970-0124) of L970-0124.wiff

Data List | Peak List

	Time (min)	Area (counts)	% Area	Height (%)	% Height	Width (min)	Baseline Type
3	1,9846	237,9825	98,9482	52,8071	98,6032	0,3333	Valley
2	1,8068	1,7910	0,7447	0,5047	0,9423	0,1100	Valley
1	0,4173	0,7387	0,3071	0,2434	0,4545	0,0833	Base to Base

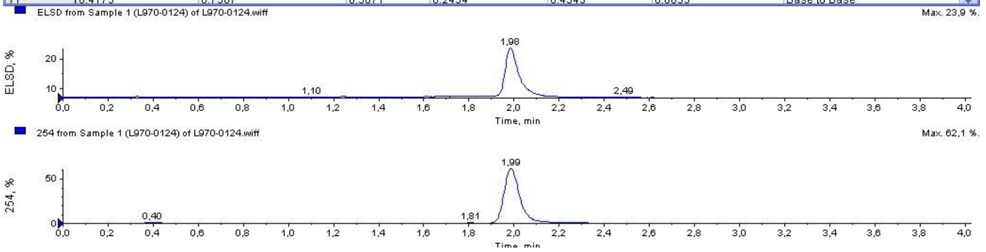

Compound: **34**	ChemDiv ID: F972-0097

C27 H32 N4 O2
M.W.=444.58

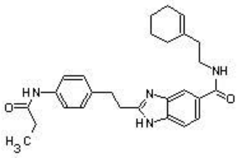

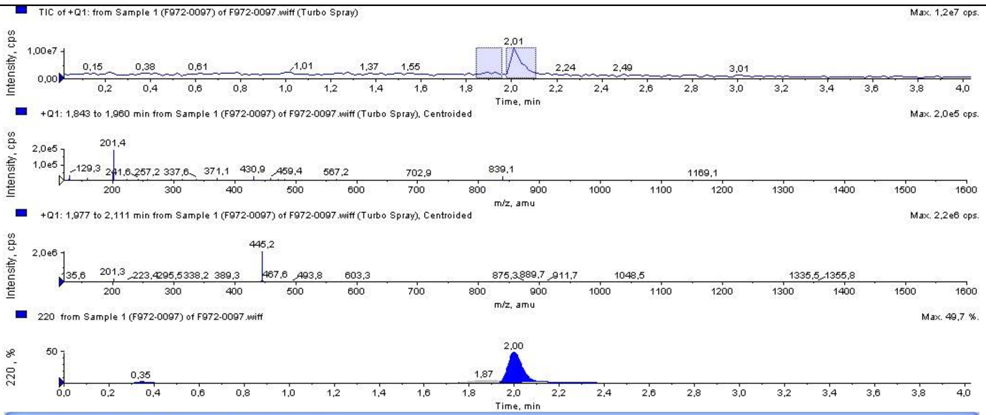

220 from Sample 1 (F972-0097) of F972-0097.wiff

Data List | Peak List

	Time (min)	Area (counts)	% Area	Height (%)	% Height	Width (min)	Baseline Type
3	1,9993	205,8544	83,7733	47,5658	87,7653	0,1933	Valley
2	1,8668	30,8241	12,5440	4,1518	7,6606	0,2233	Valley

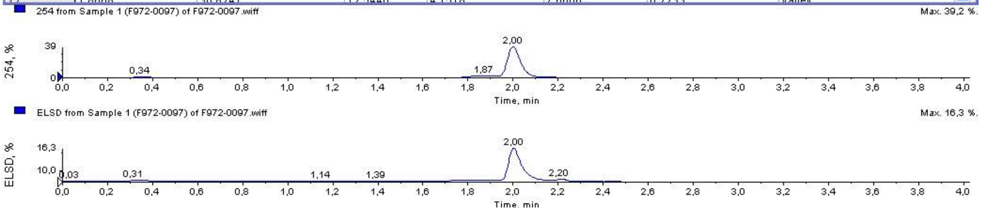

Compound: **35**	ChemDiv ID: M337-0030

C19 H15 Cl N4 O2
M.W.=366.81

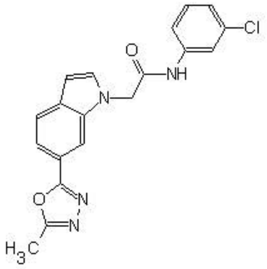

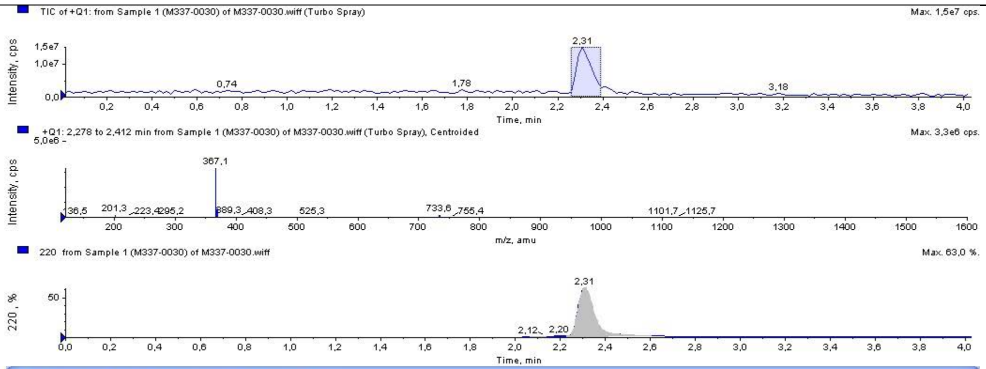

220 from Sample 1 (M337-0030) of M337-0030.wiff

Data List | Peak List

	Time (min)	Area (counts)	% Area	Height (%)	% Height	Width (min)	Baseline Type
4	2,3088	313,4997	98,0600	61,2552	96,9895	0,3767	Valley
3	2,1969	4,7053	1,4718	1,4168	2,2434	0,0867	Valley
2	2,1232	0,7787	0,2436	0,2711	0,4292	0,0767	Valley

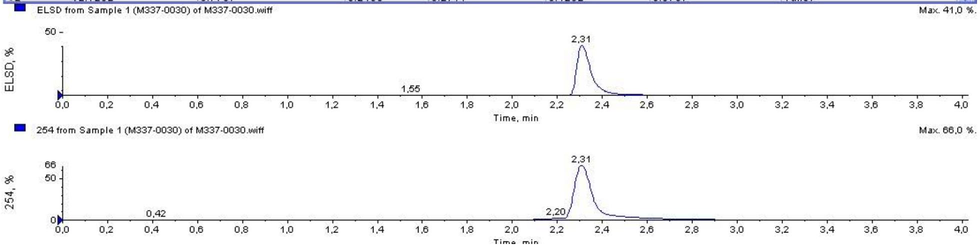

Compound: **36**	ChemDiv ID: S063-0016

C16 H19 N5 O2
M.W.=313.36

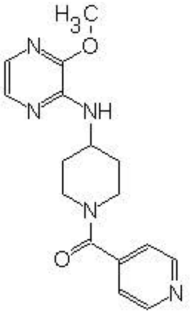

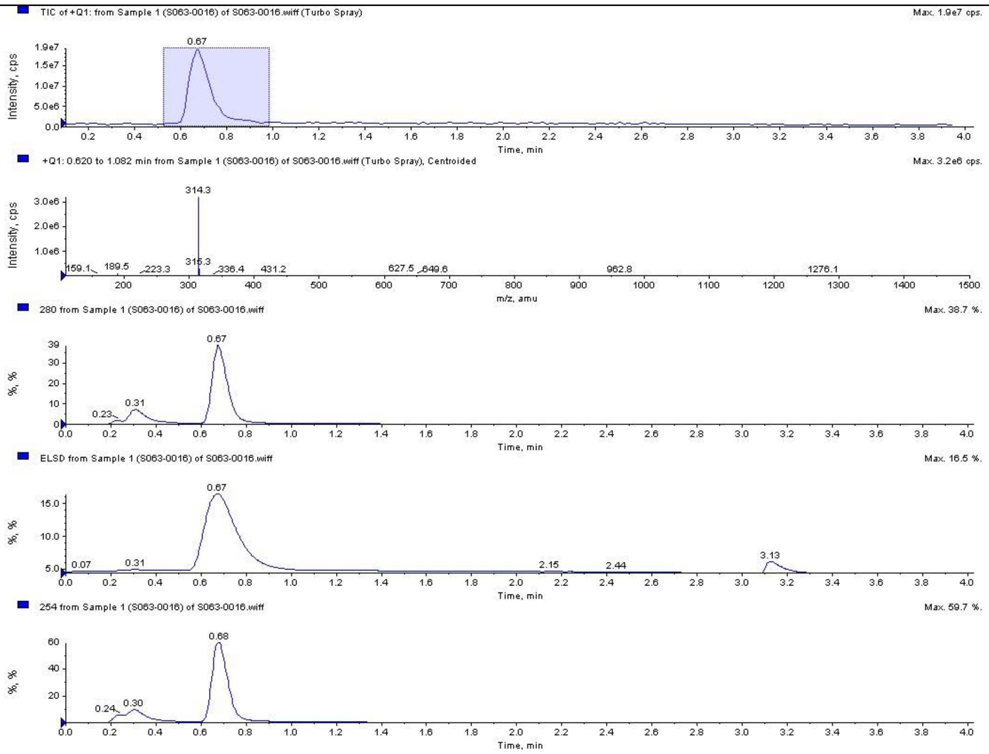

Compound: **37**	ChemDiv ID: M337-0343

C21 H20 N4 O3
M.W.=376.41

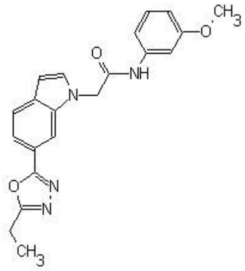

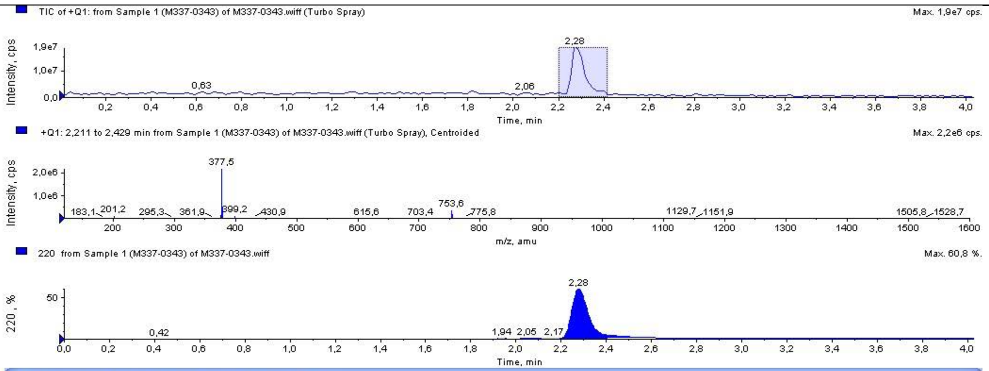

220 from Sample 1 (M337-0343) of M337-0343.wiff

Data List | Peak List

	Time (min)	Area (counts)	% Area	Height (%)	% Height	Width (min)	Baseline Type
5	2,2781	315,4225	99,1880	59,3930	98,4539	0,4233	Valley
4	2,1725	0,9333	0,2935	0,3373	0,5591	0,0800	Valley
1	0,4214	0,7268	0,2285	0,2462	0,4080	0,0800	Base to Base

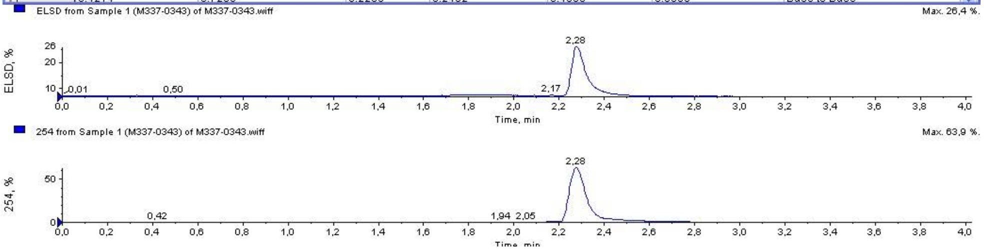

Compound: **38**	ChemDiv ID: P169-2258

C21 H20 N4 O2 S
M.W.=392.48

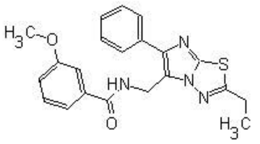

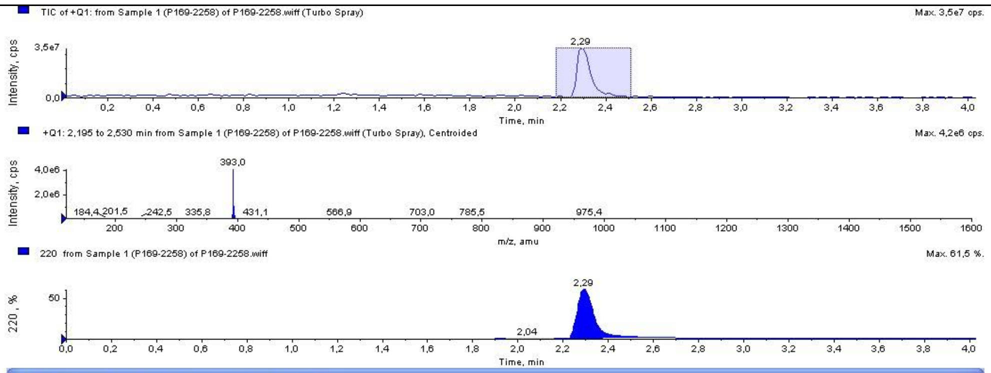

220 from Sample 1 (P169-2258) of P169-2258.wiff

Data List | Peak List

	Time (min)	Area (counts)	% Area	Height (%)	% Height	Width (min)	Baseline Type
3	2,2934	308,7088	98,6127	59,8428	98,6388	0,4267	Base to Base
2	2,0428	3,3392	1,0667	0,5217	0,8600	0,2067	Base to Base
1	0.4220	1.0038	0.3206	0.3041	0.5013	0.0933	Base to Base

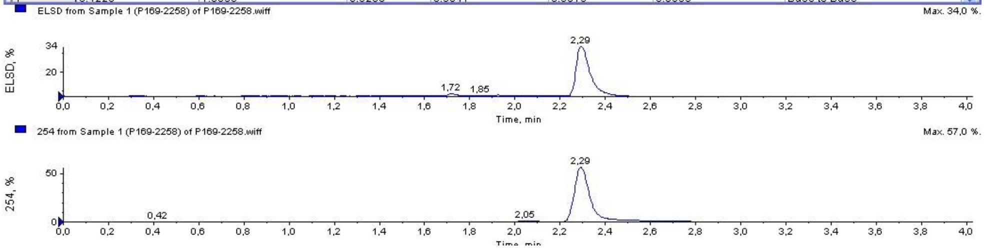

Compound: **39**	ChemDiv ID: L485-2700

C23 H23 F2 N3 O3
M.W.=427.45

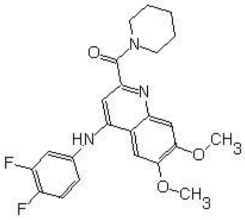

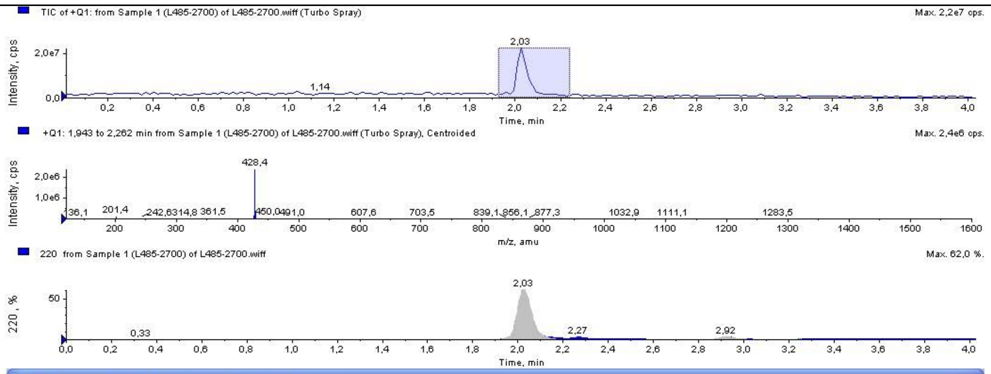

220 from Sample 1 (L485-2700) of L485-2700.wiff

Data List | Peak List

	Time (min)	Area (counts)	% Area	Height (%)	% Height	Width (min)	Baseline Type
2	2,0251	282,7000	91,6762	61,0767	91,4154	0,2333	Expo. Skim
4	2,9208	12,5917	4,0833	3,1702	4,7450	0,1633	Base to Base
3	2.2690	12.3708	4.0117	2.2491	3.3663	0.2233	Expo. Daughter

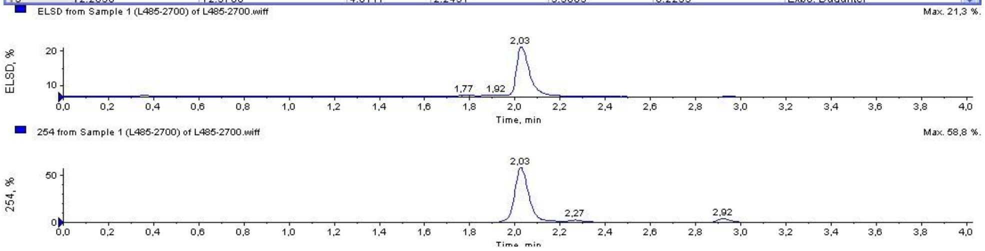

Compound: **40**	ChemDiv ID: D588-0063

C25 H23 Cl N2 O4
M.W.=450.92

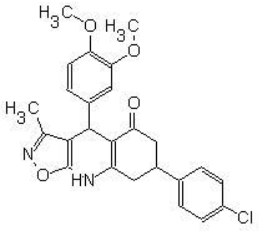

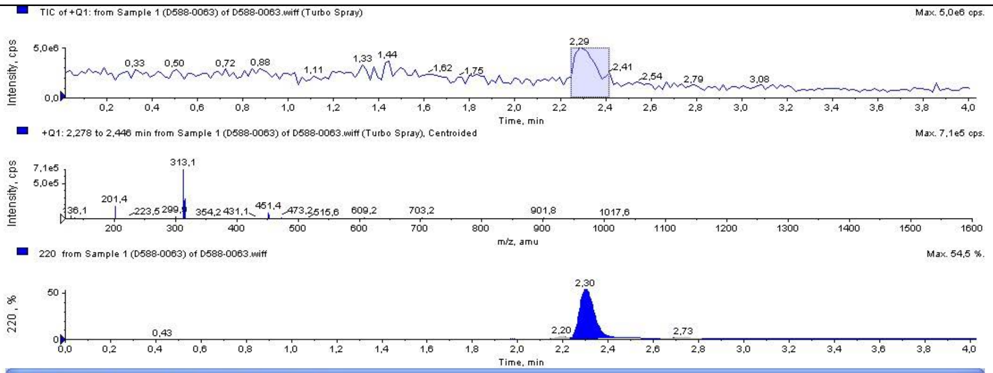

220 from Sample 1 (D588-0063) of D588-0063.wiff

	Time (min)	Area (counts)	% Area	Height (%)	% Height	Width (min)	Baseline Type
3	2,3016	241,0792	94,8558	53,1416	94,3004	0,2500	Valley
2	2,1952	7,5512	2,9711	2,1125	3,7487	0,0967	Valley
4	2,7333	4,5056	1,7728	0,8337	1,4794	0,1767	Base to Base

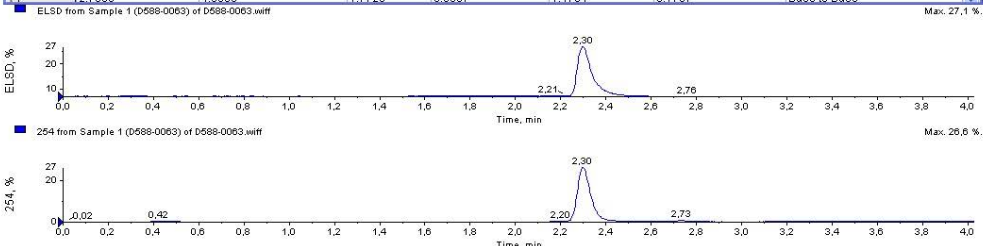

Compound: **41**	ChemDiv ID: G811-1254

C24 H24 N4 O2
M.W.=400.48

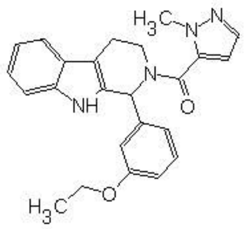

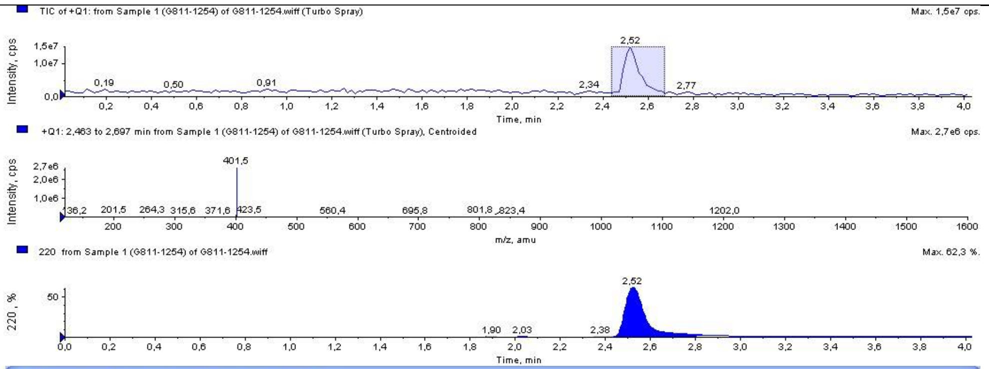

220 from Sample 1 (G811-1254) of G811-1254.wiff

	Time (min)	Area (counts)	% Area	Height (%)	% Height	Width (min)	Baseline Type
5	2,5237	380,9114	98,5787	60,9618	97,2349	0,4867	Base to Base
4	2,3821	2,2436	0,5806	0,6879	1,0973	0,1167	Base to Base
2	1,8954	1,5796	0,4088	0,4680	0,7464	0,1133	Base to Base

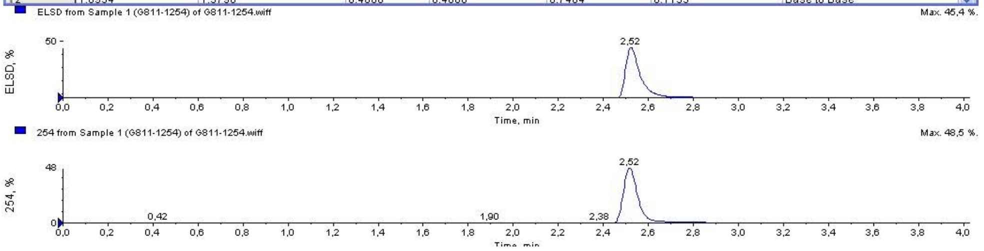

Compound: **42**	ChemDiv ID: S338-1010

C24 H27 N3 O7
M.W.=469.49

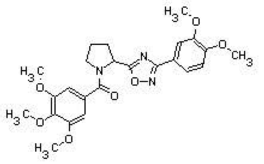

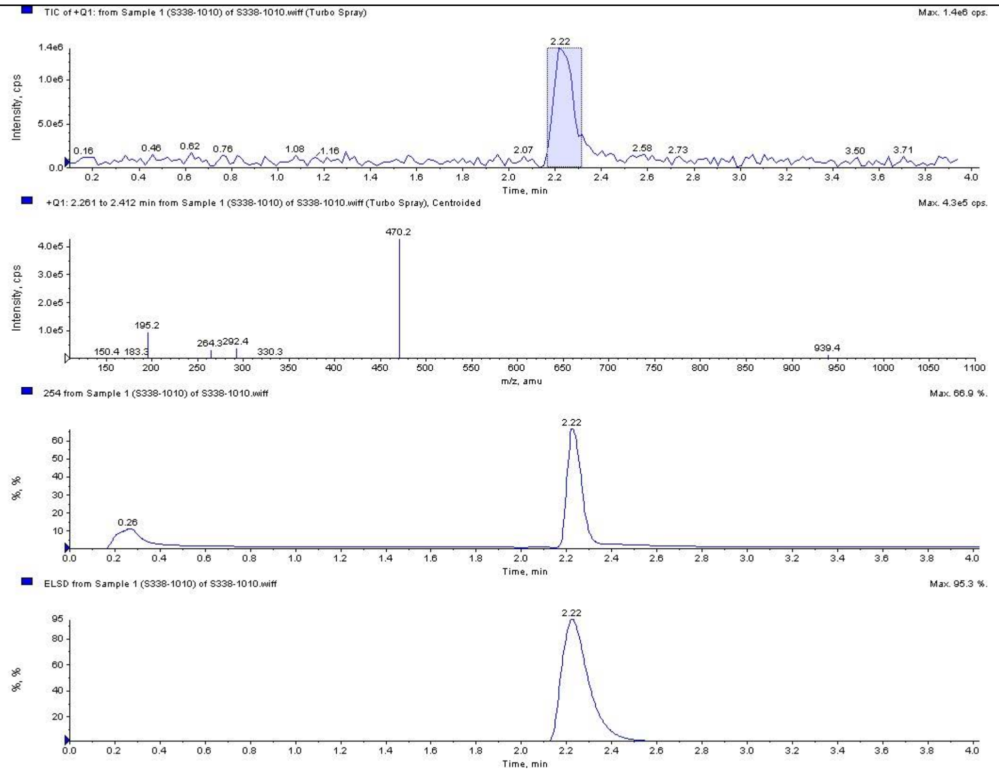

Compound: **43**	ChemDiv ID: P804-0861

C22 H22 Cl N5 O2
M.W.=423.90

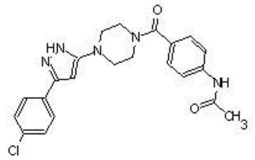

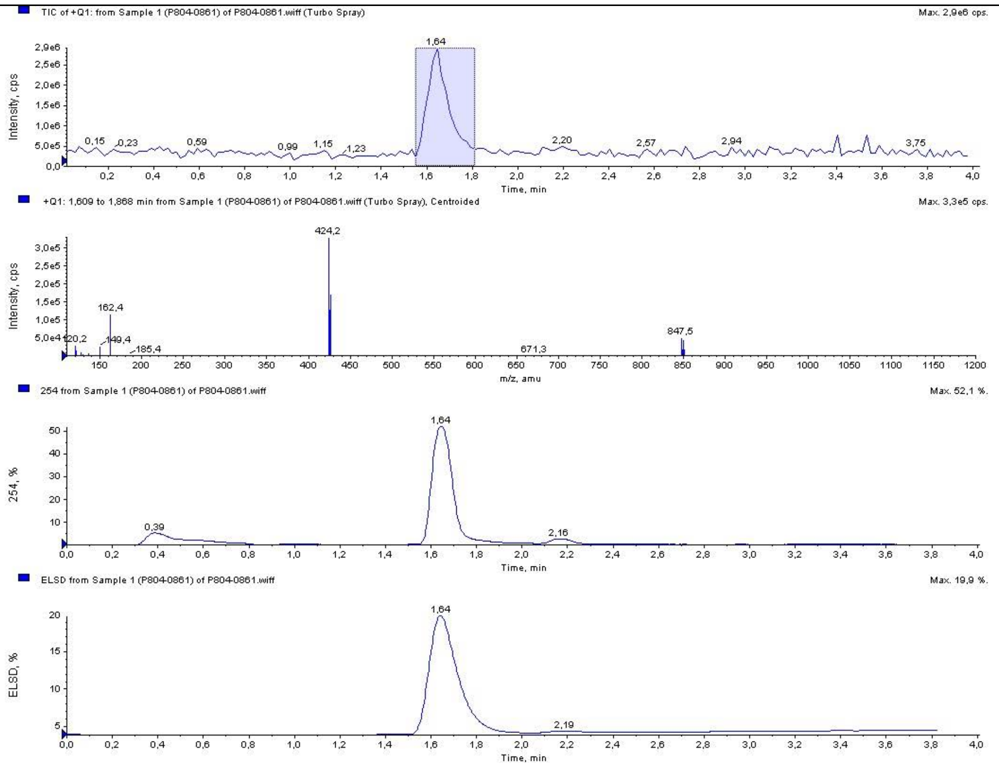

Compound: **44**	ChemDiv ID: F972-0105

C25 H26 N4 O3
M.W.=430.51

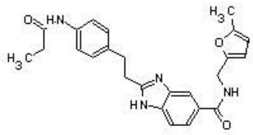

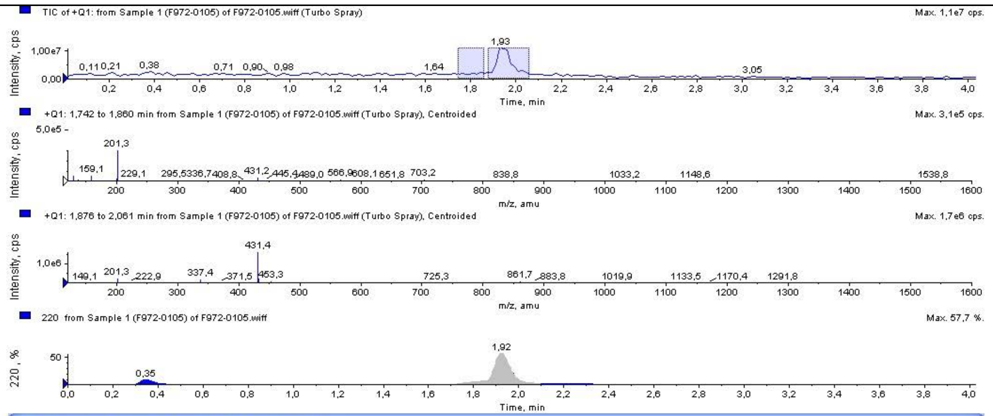

220 from Sample 1 (F972-0105) of F972-0105.wiff

Data List | Peak List

	Time (min)	Area (counts)	% Area	Height (%)	% Height	Width (min)	Baseline Type
2	1,9234	304,6520	87,8262	55,7723	85,5104	0,4133	Base to Base
1	0,3477	42,2285	12,1738	9,4506	14,4896	0,2367	Base to Base

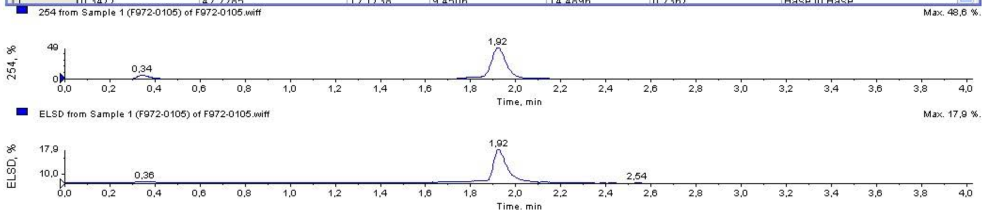

Compound: **45**	ChemDiv ID: M337-0025

C20 H18 N4 O3
M.W.=362.39

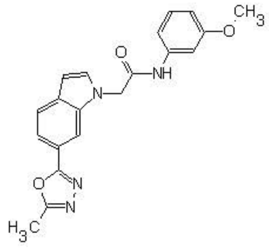

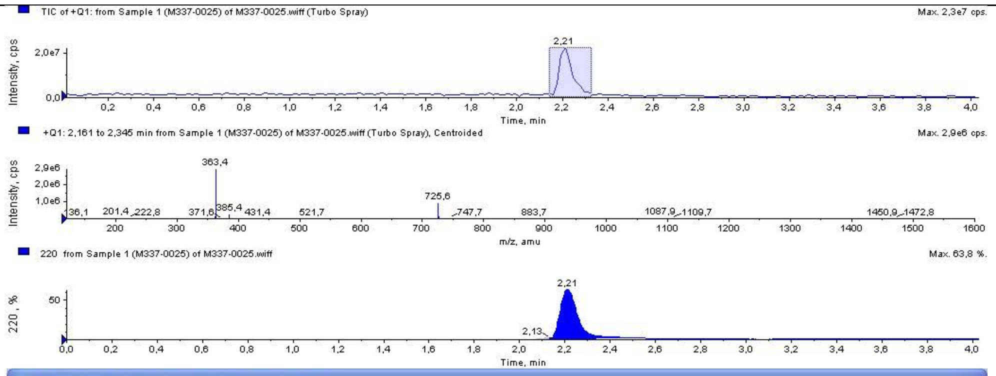

220 from Sample 1 (M337-0025) of M337-0025.wiff

Data List | Peak List

	Time (min)	Area (counts)	% Area	Height (%)	% Height	Width (min)	Baseline Type
3	2,2112	342,0301	98,8438	62,4595	97,2558	0,4133	Valley
2	2,1261	3,3305	0,9625	1,5577	2,4256	0,0800	Valley
1	0,4258	0,6701	0,1937	0,2046	0,3186	0,0767	Base to Base

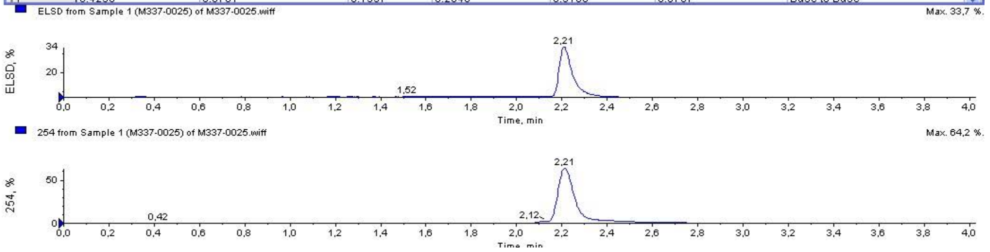

Compound: **46**	ChemDiv ID: D622-0107

C20 H18 F3 N3 O5
M.W.=437.38

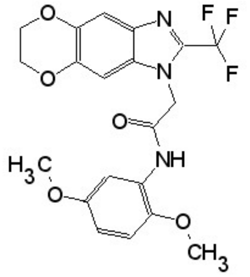

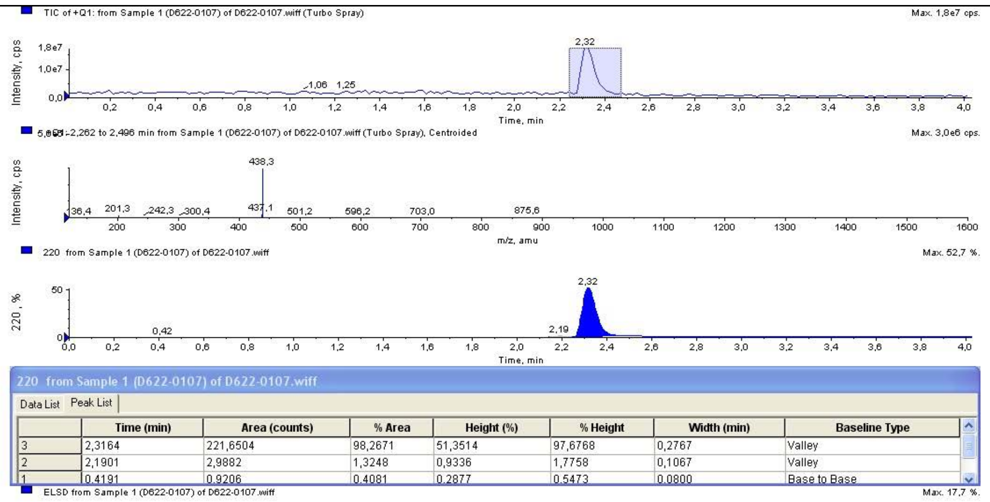

220 from Sample 1 (D622-0107) of D622-0107.wiff

Data List | Peak List

	Time (min)	Area (counts)	% Area	Height (%)	% Height	Width (min)	Baseline Type
3	2,3164	221,6504	98,2671	51,3514	97,6768	0,2767	Valley
2	2,1901	2,9882	1,3248	0,9336	1,7758	0,1067	Valley
1	0,4191	0,9206	0,4081	0,2877	0,5473	0,0800	Base to Base

Compound: **47**	ChemDiv ID: S051-0072

C21 H20 N4 O5
M.W.=408.41

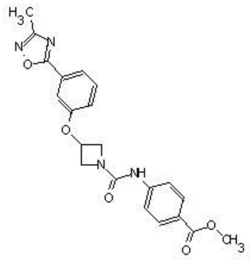

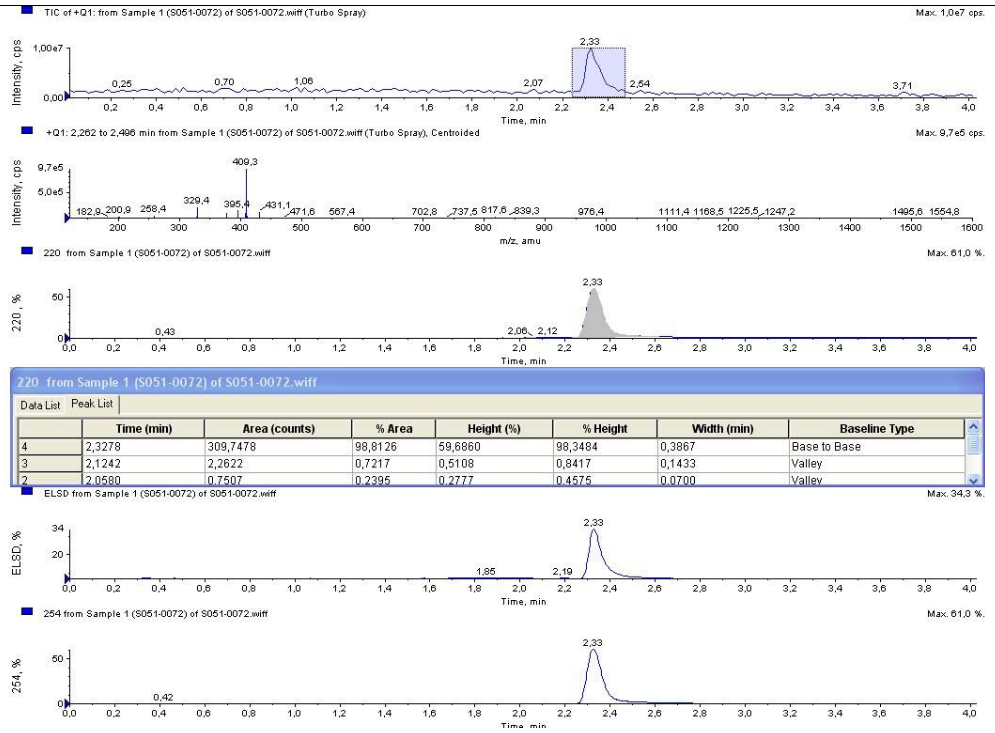

220 from Sample 1 (S051-0072) of S051-0072.wiff

Data List | Peak List

	Time (min)	Area (counts)	% Area	Height (%)	% Height	Width (min)	Baseline Type
4	2,3278	309,7478	98,8126	59,6860	98,3484	0,3867	Base to Base
3	2,1242	2,2622	0,7217	0,5108	0,8417	0,1433	Valley
2	2,0580	0,7507	0,2395	0,2777	0,4575	0,0700	Valley

Compound: **48**	ChemDiv ID: M337-0418

C22 H22 N4 O4
M.W.=406.44

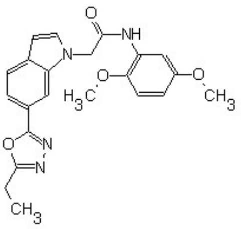

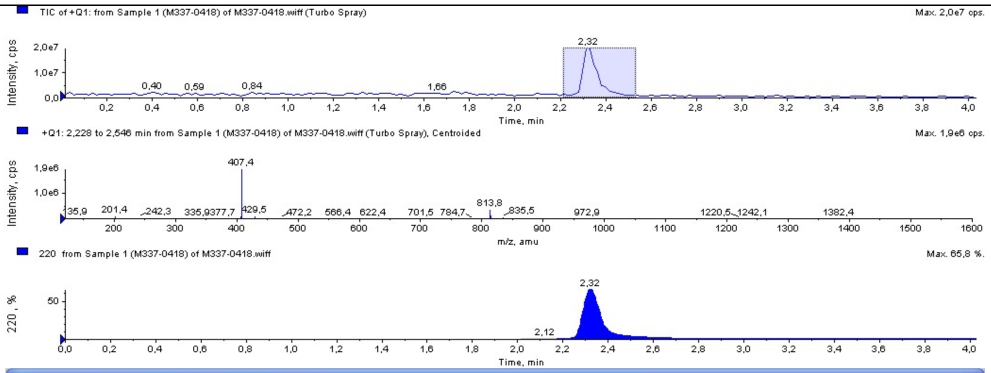

220 from Sample 1 (M337-0418) of M337-0418.wiff

Data List | Peak List

	Time (min)	Area (counts)	% Area	Height (%)	% Height	Width (min)	Baseline Type
3	2,3223	363,8732	98,6122	64,1802	98,1836	0,5167	Valley
2	2,1194	4,5443	1,2315	0,9887	1,5125	0,1700	Valley
1	0,4207	0,5767	0,1563	0,1987	0,3039	0,0800	Base to Base

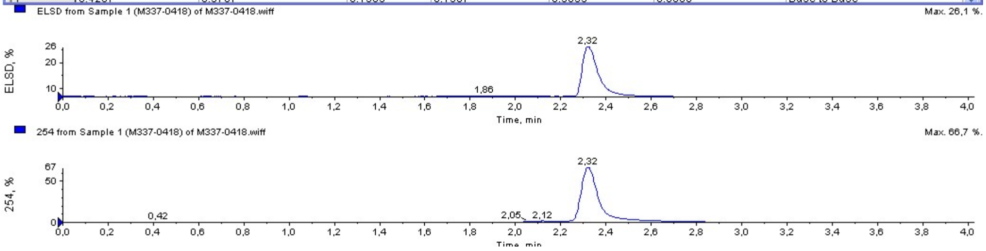

Compound: **49**	ChemDiv ID: F972-0045

C27 H26 N4 O4
M.W.=470.53

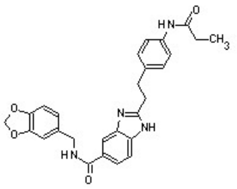

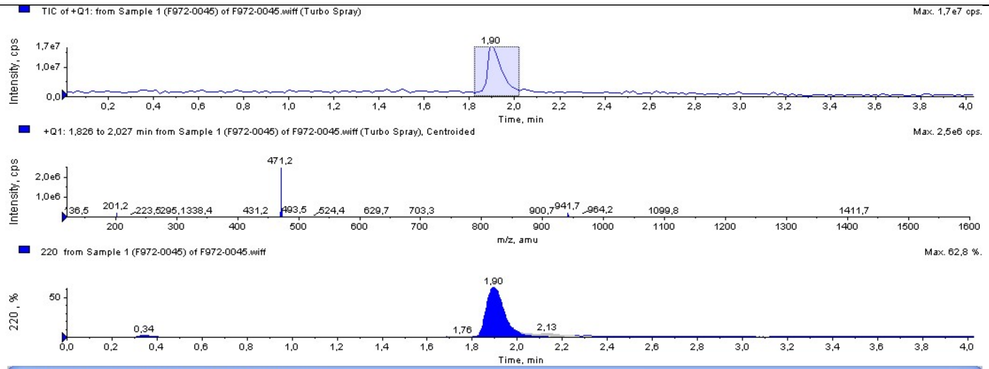

220 from Sample 1 (F972-0045) of F972-0045.wiff

Data List | Peak List

	Time (min)	Area (counts)	% Area	Height (%)	% Height	Width (min)	Baseline Type
3	1,8957	348,4536	90,7062	61,4951	90,2028	0,2100	Expo. Skim
4	2,1325	20,0802	5,2271	3,0799	4,5177	0,2400	Expo. Daughter
1	0,3435	8,0147	2,0863	2,2420	3,2886	0,1267	Base to Base

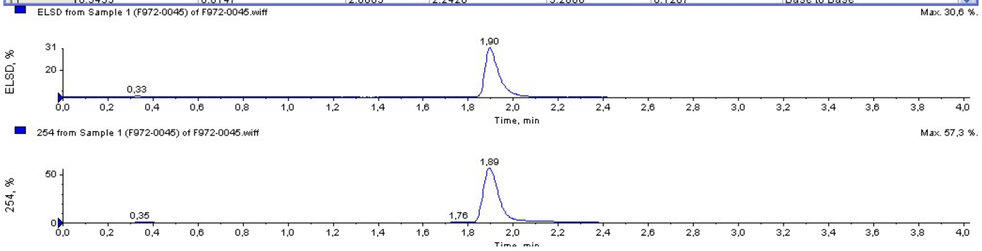

Compound: **50** ChemDiv ID: P006-0346

C19 H18 N4 O2
M.W.=334.38

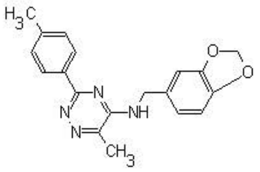

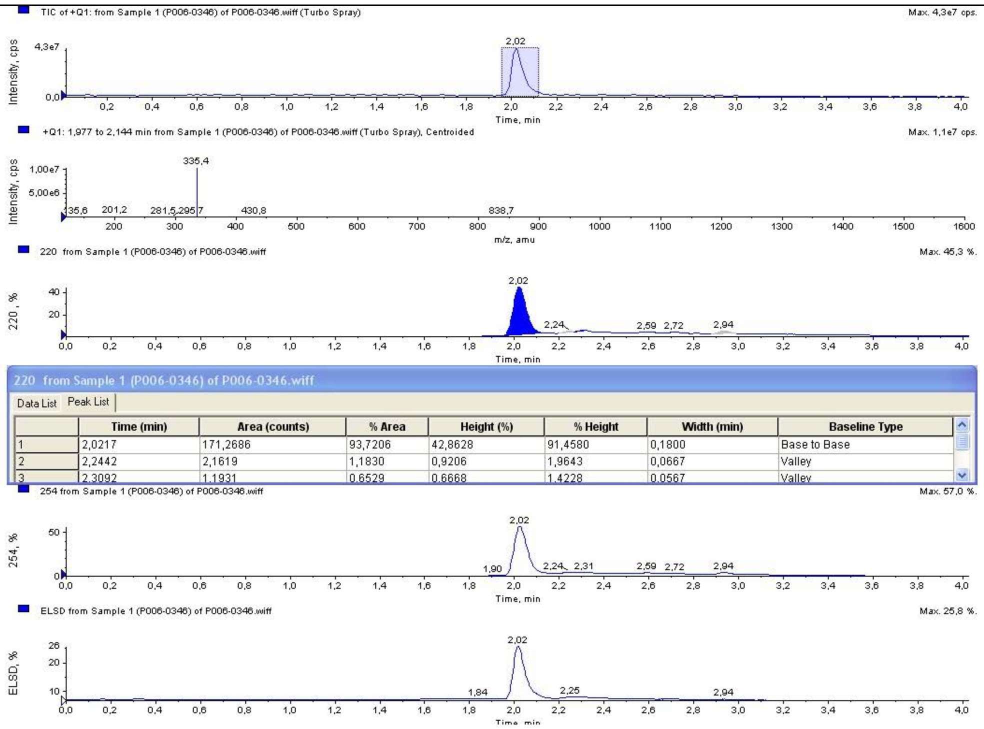

	Time (min)	Area (counts)	% Area	Height (%)	% Height	Width (min)	Baseline Type
1	2,0217	171,2686	93,7206	42,8628	91,4580	0,1800	Base to Base
2	2,2442	2,1619	1,1830	0,9206	1,9643	0,0667	Valley
3	2,3092	1,1931	0,6529	0,6668	1,4228	0,0567	Valley

Compound: **51** ChemDiv ID: D600-0244

C20 H17 Br N4 O2
M.W.=425.28

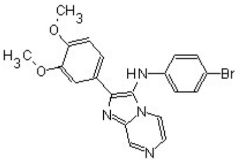

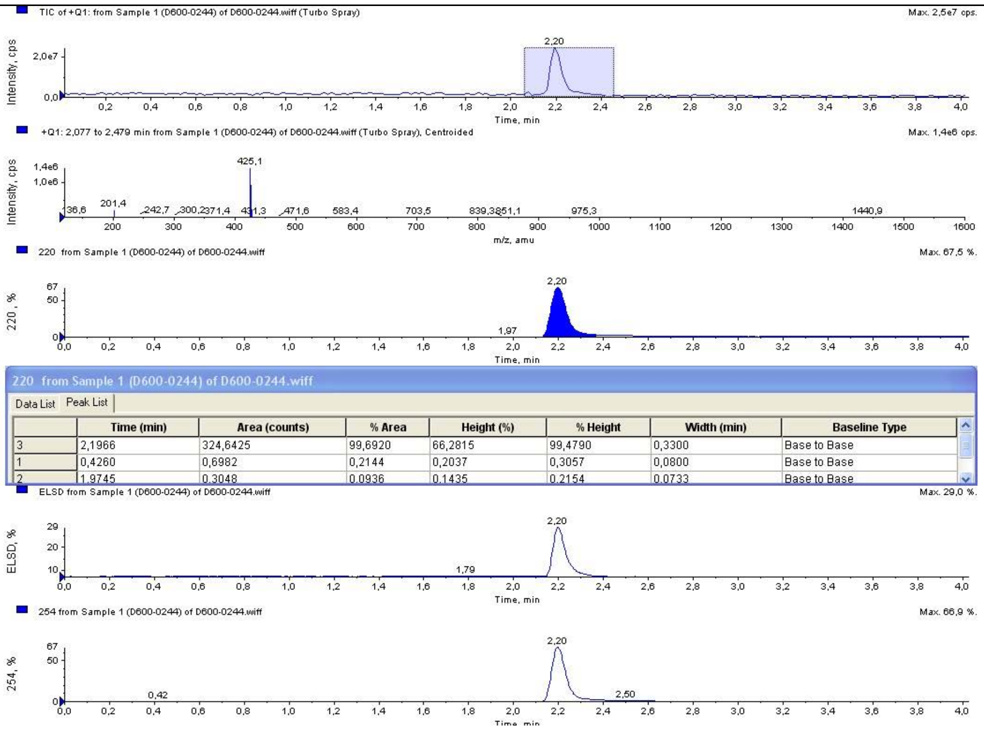

	Time (min)	Area (counts)	% Area	Height (%)	% Height	Width (min)	Baseline Type
3	2,1966	324,6425	99,6920	66,2815	99,4790	0,3300	Base to Base
1	0,4260	0,6982	0,2144	0,2037	0,3057	0,0800	Base to Base
2	1,9745	0,3048	0,0936	0,1435	0,2154	0,0733	Base to Base

Compound: **53**	ChemDiv ID: S333-0330

C22 H18 N4 O3
M.W.=386.41

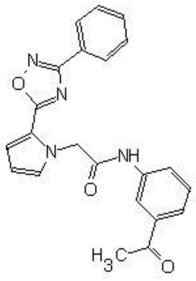

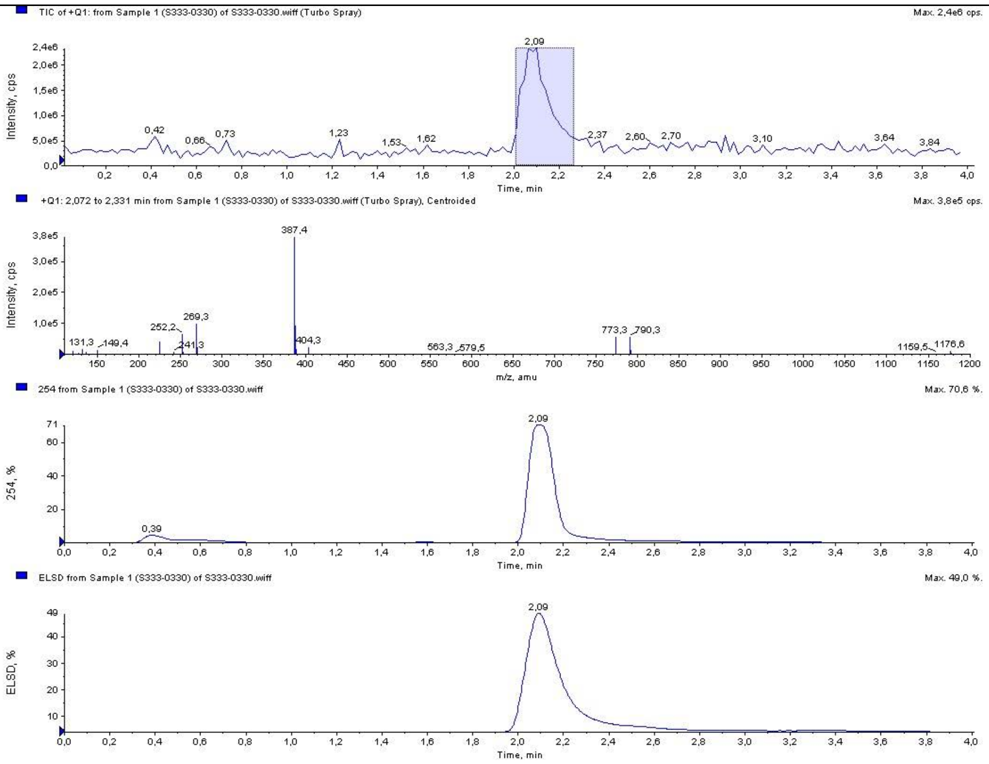

Compound: **54**	ChemDiv ID: M337-0141

C21 H20 N4 O2
M.W.=360.42

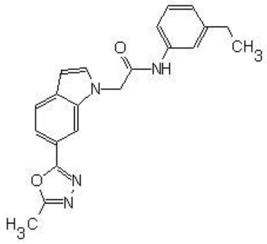

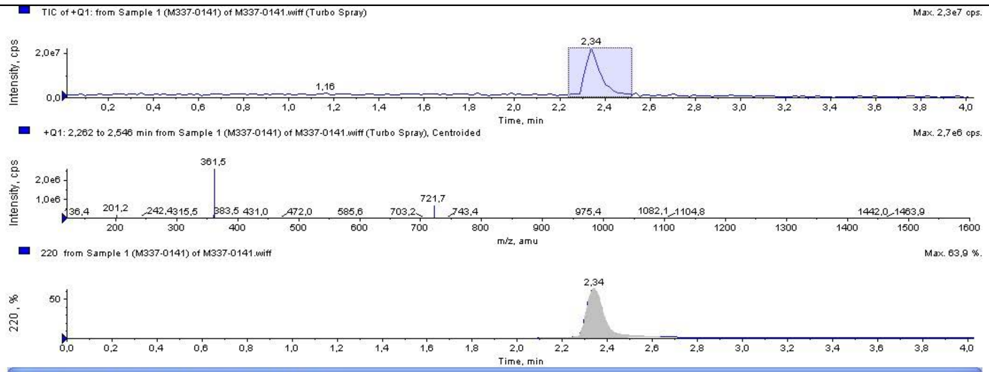

220 from Sample 1 (M337-0141) of M337-0141.wiff

Data List | Peak List

	Time (min)	Area (counts)	% Area	Height (%)	% Height	Width (min)	Baseline Type
2	2,3405	332,1872	99,7888	62,0963	99,6416	0,5500	Base to Base
1	0,4236	0,7029	0,2112	0,2233	0,3584	0,0767	Base to Base

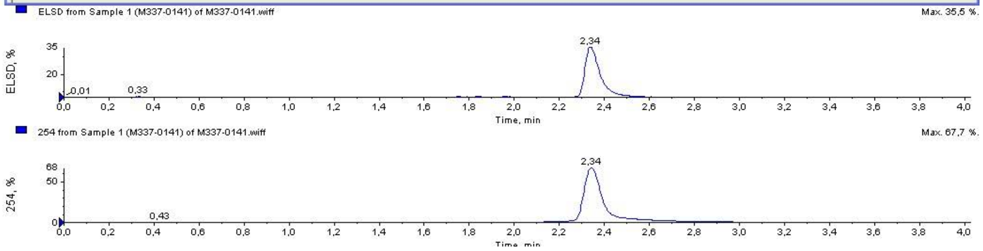

Compound: **55** ChemDiv ID: M976-0157

C23 H23 N3 O5
M.W.=421.45

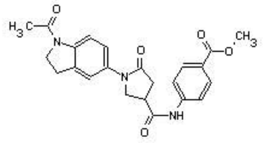

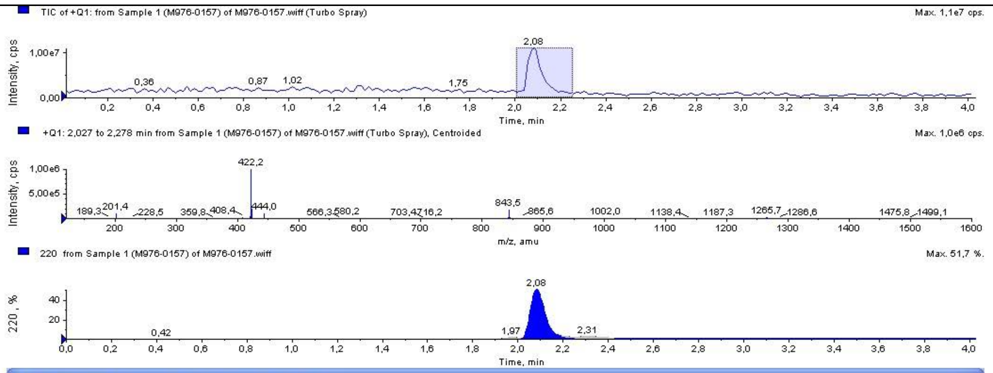

220 from Sample 1 (M976-0157) of M976-0157.wiff

Data List | Peak List

	Time (min)	Area (counts)	% Area	Height (%)	% Height	Width (min)	Baseline Type
3	2,0829	233,1393	93,2825	50,6639	93,4675	0,1800	Expo. Skim
4	2,3106	11,6347	4,6552	1,9966	3,6834	0,2300	Expo. Daughter
2	1,9722	4,4246	1,7703	1,3117	2,4199	0,1033	Valley

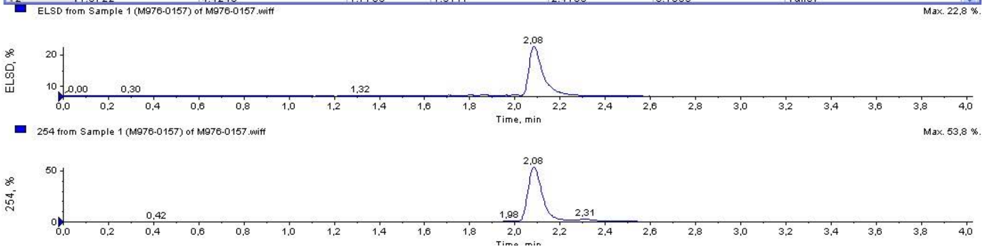

Compound: **56** ChemDiv ID: N124-0015

C29 H37 N O8
M.W.=527.61

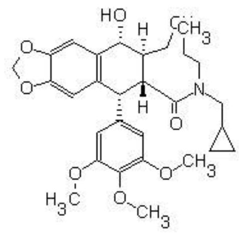

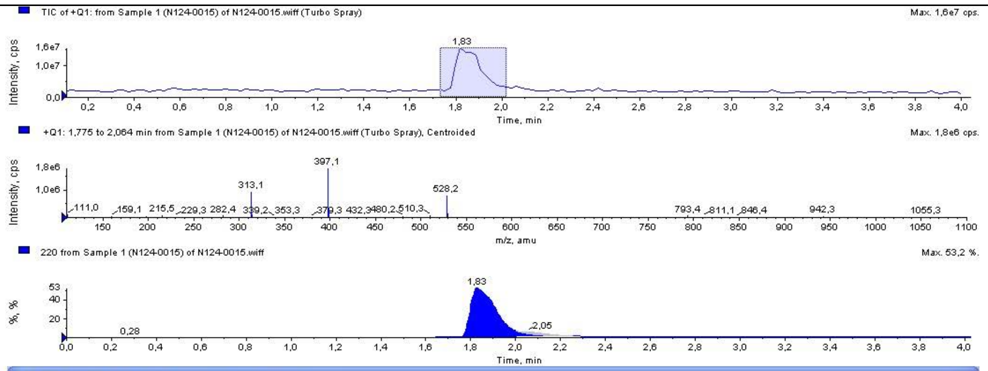

220 from Sample 1 (N124-0015) of N124-0015.wiff

Data List | Peak List

	Time (min)	Area (counts)	% Area	Height (%)	% Height	Width (min)	Baseline Type
3	1,8275	421,2039	95,7481	51,8823	95,1854	0,2567	Expo. Skim
4	2,0548	16,7842	3,8154	1,9118	3,5074	0,2500	Expo. Daughter
2	0,2845	1,1690	0,2657	0,3944	0,7236	0,0833	Base to Base

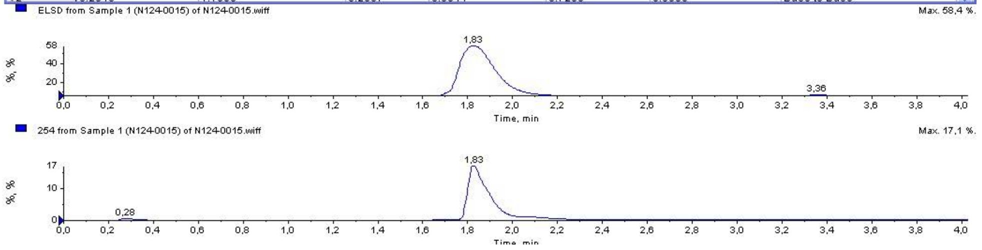

Compound: 57 ChemDiv ID: P165-2563

C23 H20 F N5 O4
M.W.=449.44

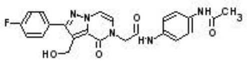

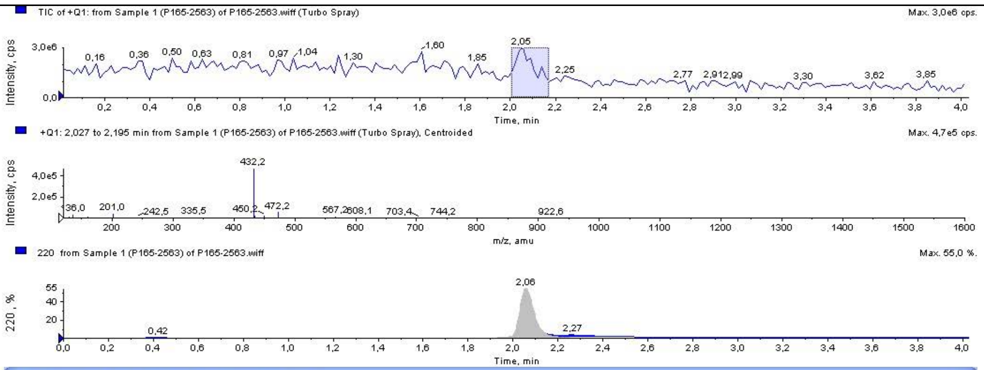

220 from Sample 1 (P165-2563) of P165-2563.wiff

Data List | Peak List

	Time (min)	Area (counts)	% Area	Height (%)	% Height	Width (min)	Baseline Type
2	2,0574	239,1019	93,5647	53,4552	95,4853	0,2367	Expo. Skim
3	2,2653	10,0461	3,9312	1,4533	2,5960	0,2133	Expo. Daughter
1	0,4227	6,3990	2,5040	1,0742	1,9187	0,2033	Base to Base

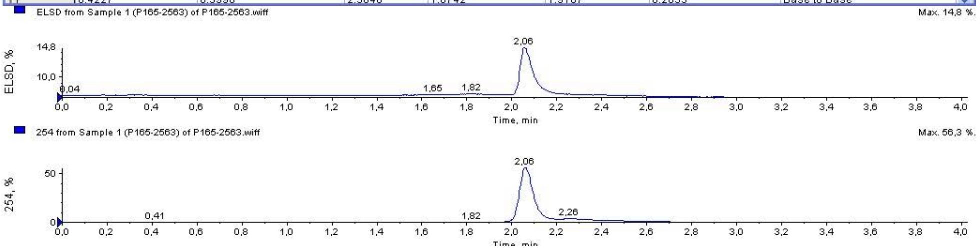

Compound: 59 ChemDiv ID: P018-0789

C18 H14 F N5 O2 S
M.W.=383.41

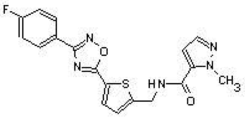

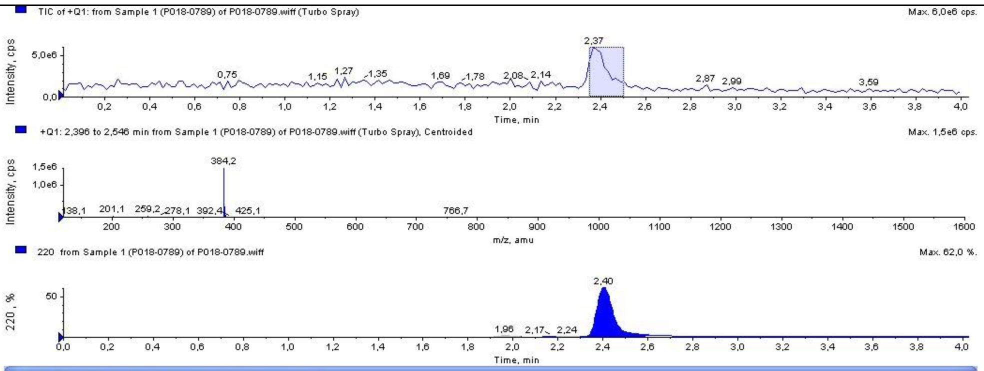

220 from Sample 1 (P018-0789) of P018-0789.wiff

Data List | Peak List

	Time (min)	Area (counts)	% Area	Height (%)	% Height	Width (min)	Baseline Type
5	2,4043	310,6303	95,5815	60,5561	94,4428	0,3800	Base to Base
2	1,9631	8,0291	2,4706	1,6830	2,6248	0,1733	Base to Base
4	2,2409	3,8280	1,1779	1,0520	1,6408	0,1067	Valley

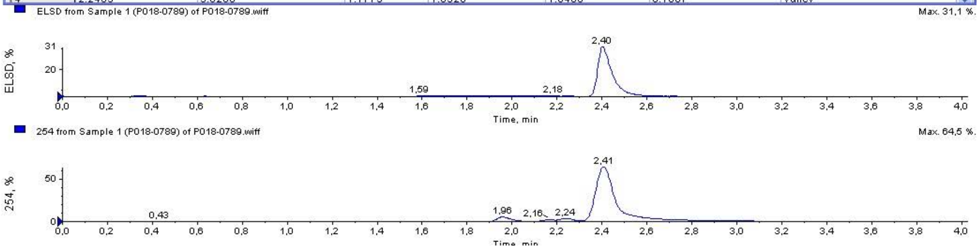

Compound: **60**	ChemDiv ID: D667-0063

C20 H16 N4 O3
M.W.=360.37

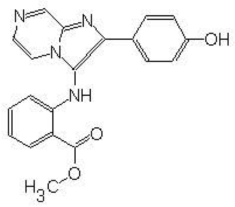

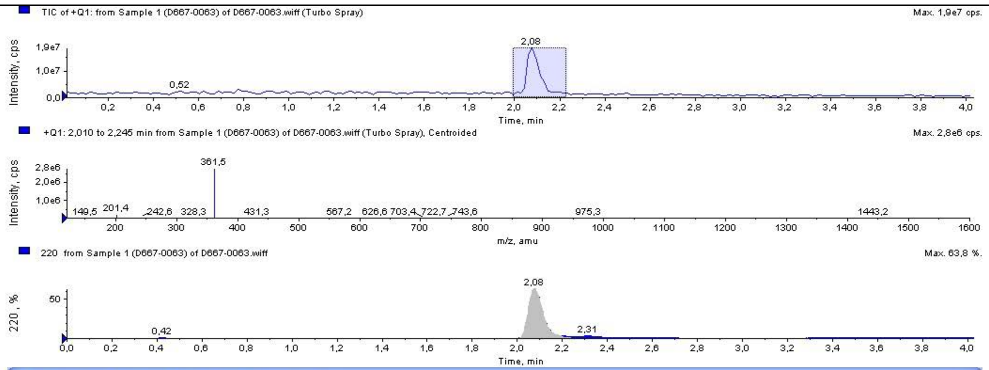

220 from Sample 1 (D667-0063) of D667-0063.wiff

Data List | Peak List

	Time (min)	Area (counts)	% Area	Height (%)	% Height	Width (min)	Baseline Type
2	2,0761	286,9922	94,9074	62,7663	95,9896	0,2067	Expo. Skim
3	2,3112	12,4918	4,1310	1,9260	2,9455	0,2200	Expo. Daughter
1	0,4239	2,9077	0,9616	0,6963	1,0649	0,1400	Base to Base

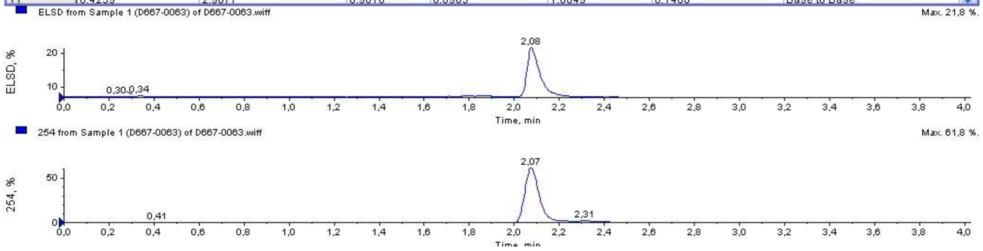

Compound: **61**	ChemDiv ID: S333-0480

C23 H18 N4 O5
M.W.=430.42

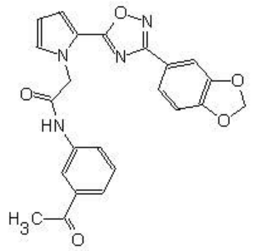

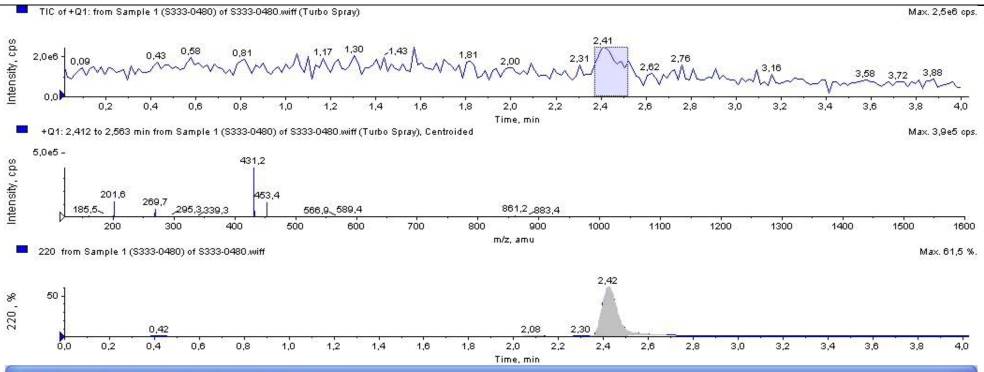

220 from Sample 1 (S333-0480) of S333-0480.wiff

Data List | Peak List

	Time (min)	Area (counts)	% Area	Height (%)	% Height	Width (min)	Baseline Type
4	2,4238	292,0144	97,0067	59,9997	97,0986	0,3367	Base to Base
1	0,4219	6,3717	2,1167	1,0719	1,7347	0,2133	Base to Base
2	2,0801	2,1210	0,7046	0,5475	0,8861	0,1267	Base to Base

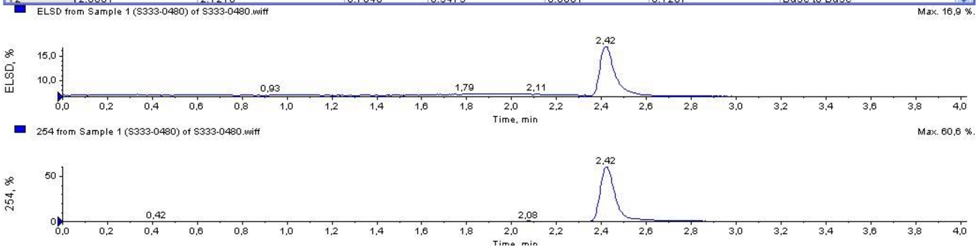

Compound: **62**	ChemDiv ID: M321-0040

C20 H17 N5 O2
M.W.=359.39

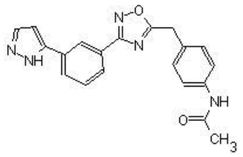

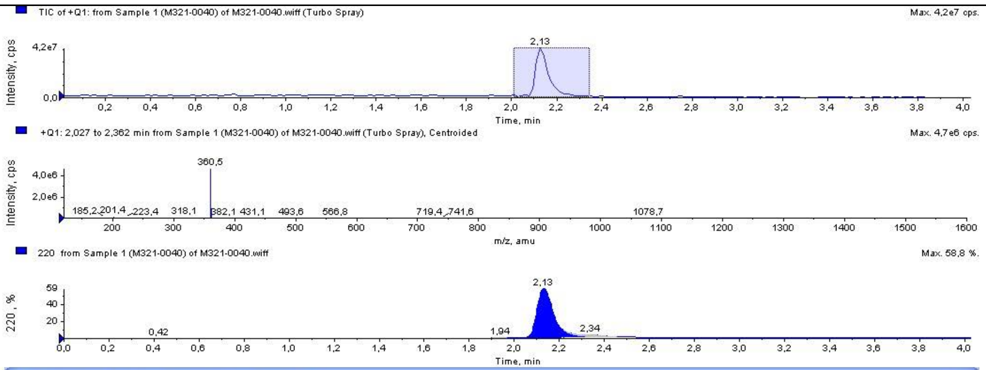

220 from Sample 1 (M321-0040) of M321-0040.wiff

Data List | Peak List

	Time (min)	Area (counts)	% Area	Height (%)	% Height	Width (min)	Baseline Type
3	2,1312	299,7942	94,8232	57,1659	95,3165	0,2500	Expo. Skim
4	2,3374	13,8673	4,3862	2,0678	3,4478	0,2333	Expo. Daughter
2	1,9376	1,7821	0,5637	0,5016	0,8363	0,1033	Valley

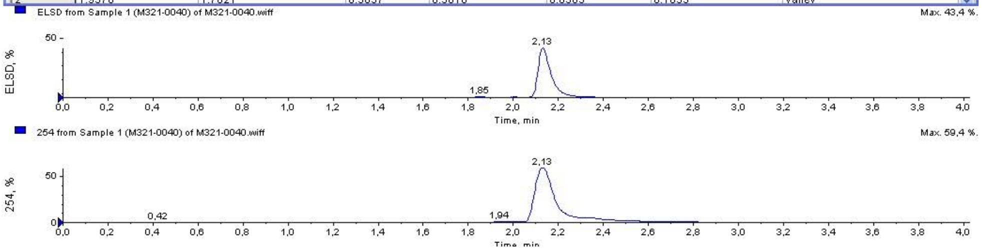

Compound: **63**	ChemDiv ID: P018-1139

C16 H13 N5 O2 S2
M.W.=371.44

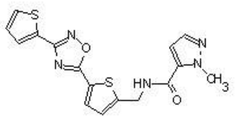

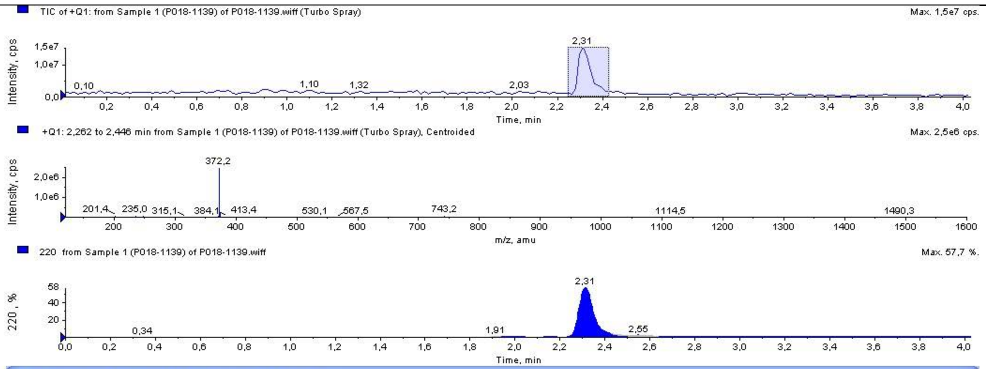

220 from Sample 1 (P018-1139) of P018-1139.wiff

Data List | Peak List

	Time (min)	Area (counts)	% Area	Height (%)	% Height	Width (min)	Baseline Type
3	2,3124	262,9313	96,2694	56,5600	96,7661	0,2067	Expo. Skim
4	2,5492	8,7835	3,2160	1,3484	2,3070	0,2100	Expo. Daughter
1	0,3431	0,9519	0,3485	0,3560	0,6091	0,0933	Base to Base

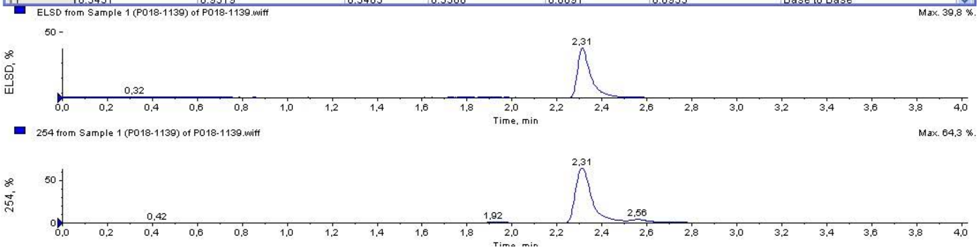

Supplier-provided NMR spectra for commercially purchased compounds

1

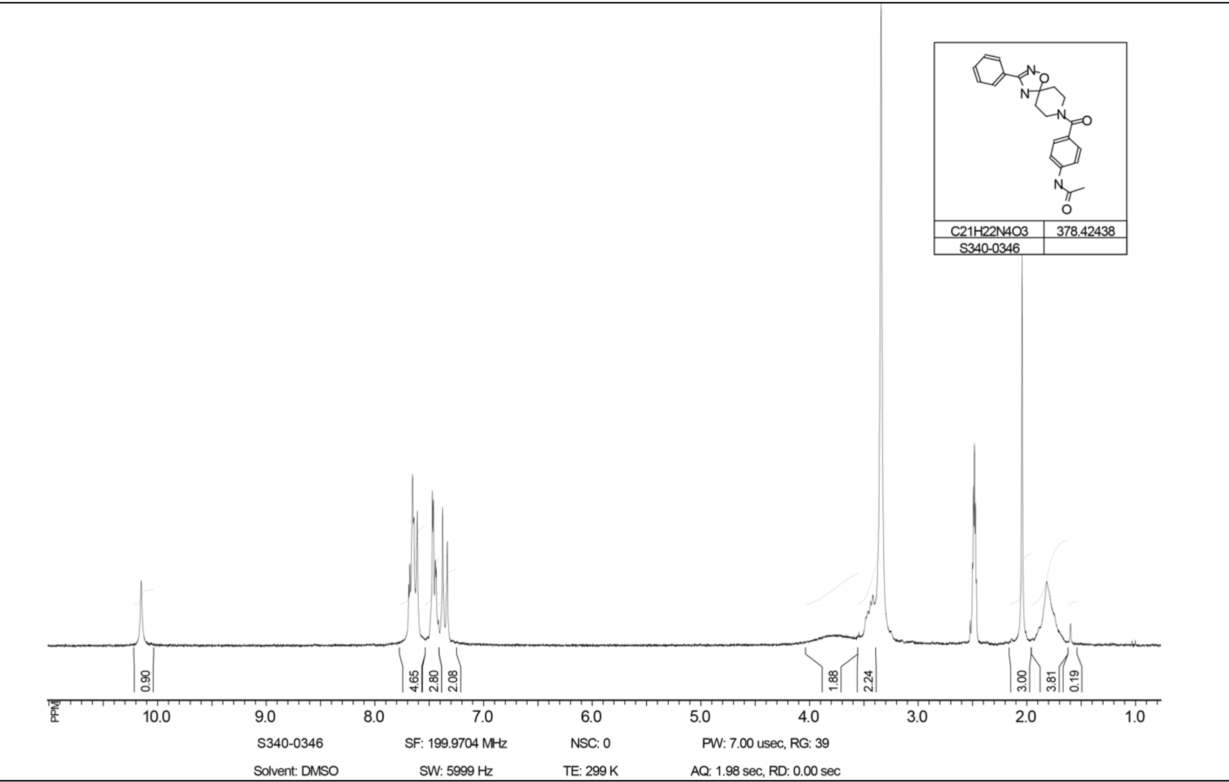

2

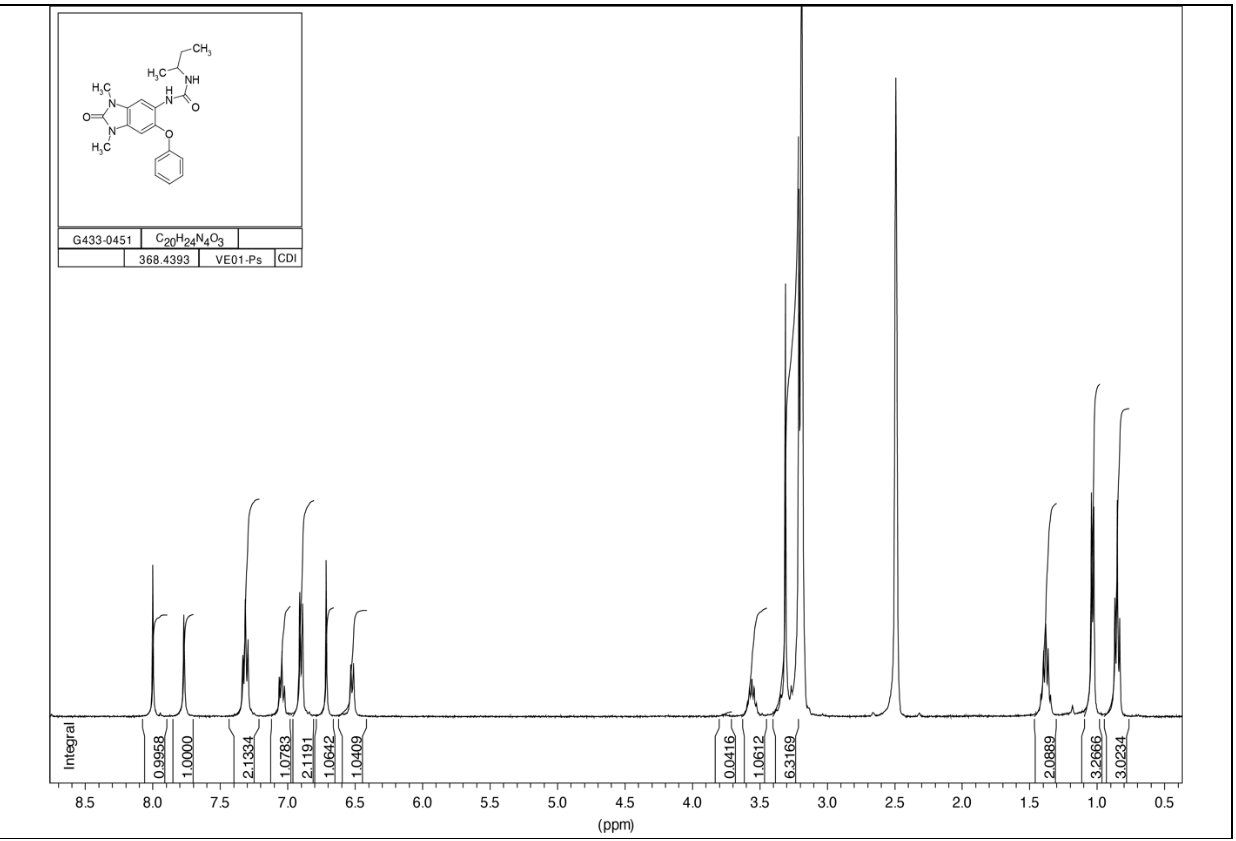

3

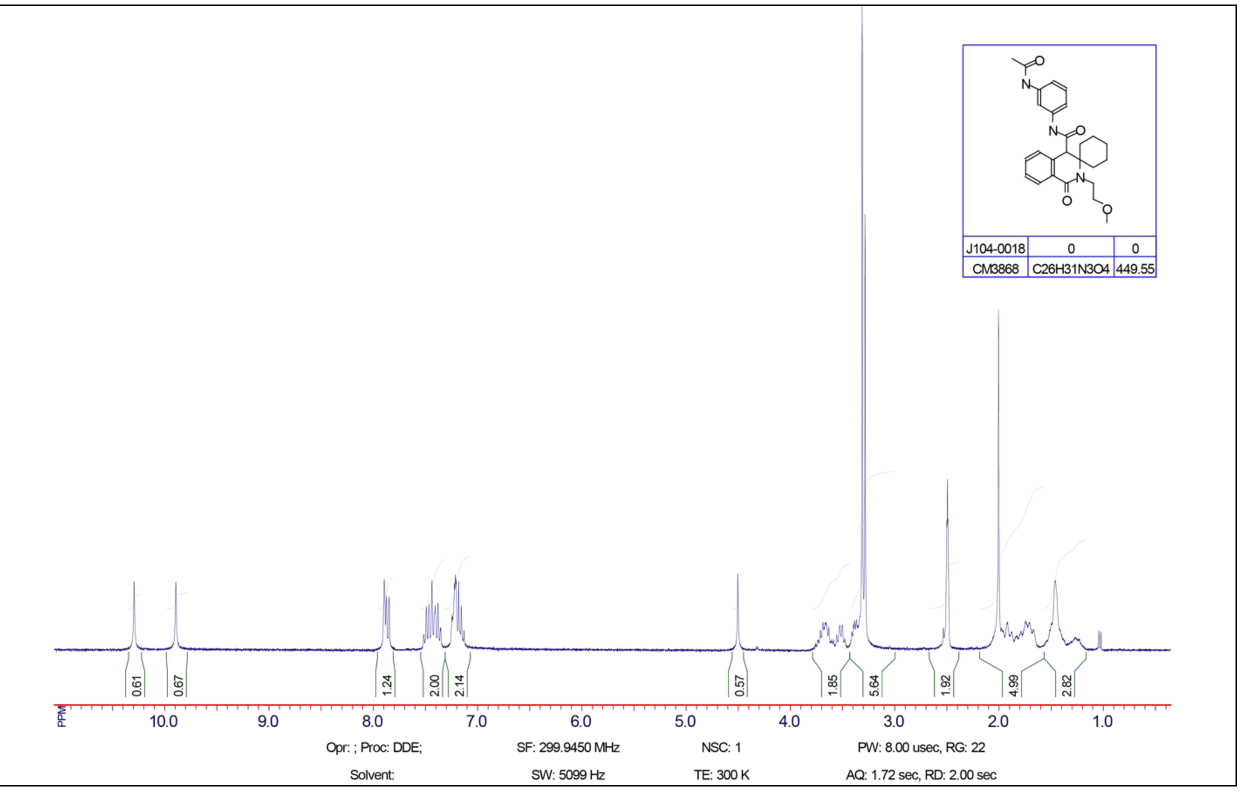
J104-0018 | 0 | 0
CM3868 | C26H31N3O4 | 449.55
0.61 0.67 1.24 2.00 2.14 0.57 1.85 5.64 1.92 4.99 2.82
PPM 10.0 9.0 8.0 7.0 6.0 5.0 4.0 3.0 2.0 1.0
Opr: ; Proc: DDE;
SF: 299.9450 MHz
NSC: 1
PW: 8.00 usec, RG: 22
Solvent:
SW: 5099 Hz
TE: 300 K
AQ: 1.72 sec, RD: 2.00 sec

4

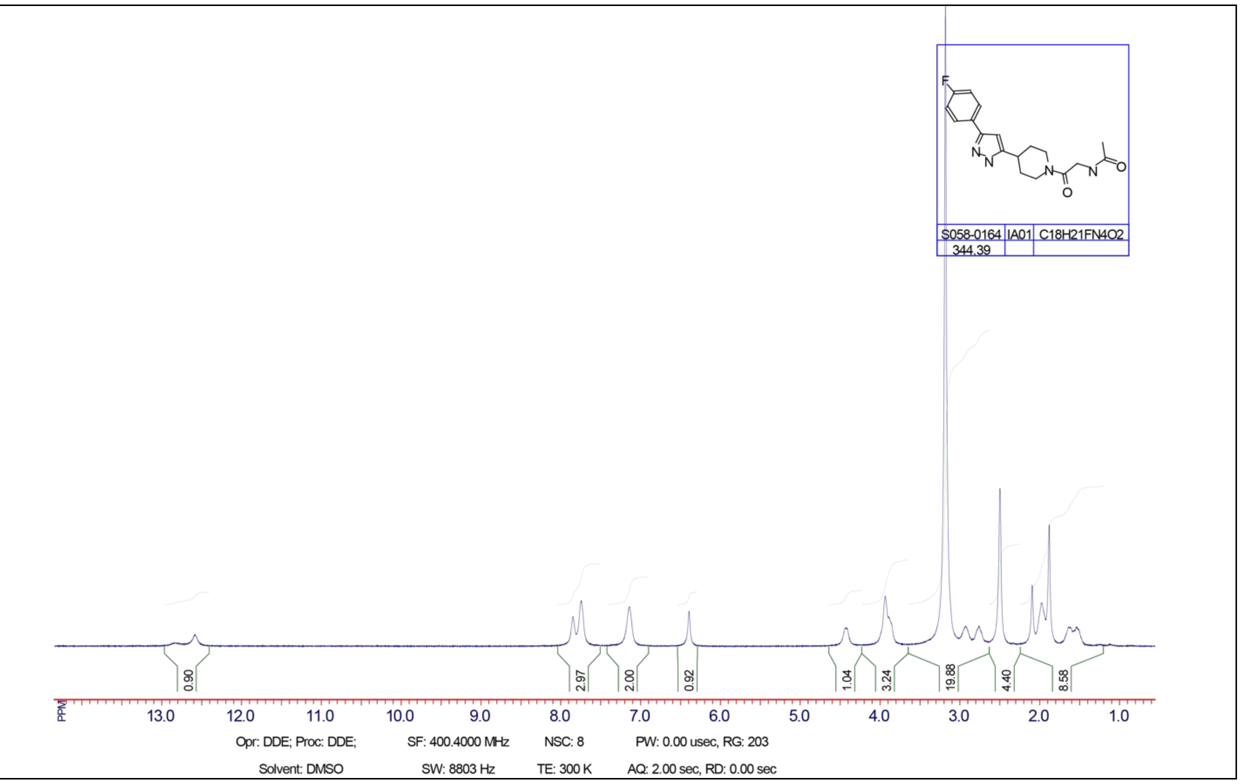
S058-0164 | IA01 | C18H21FN4O2
344.39
0.90 2.97 2.00 0.92 1.04 3.24 19.88 4.40 8.58
PPM 13.0 12.0 11.0 10.0 9.0 8.0 7.0 6.0 5.0 4.0 3.0 2.0 1.0
Opr: DDE; Proc: DDE;
SF: 400.4000 MHz
NSC: 8
PW: 0.00 usec, RG: 203
Solvent: DMSO
SW: 8803 Hz
TE: 300 K
AQ: 2.00 sec, RD: 0.00 sec

7

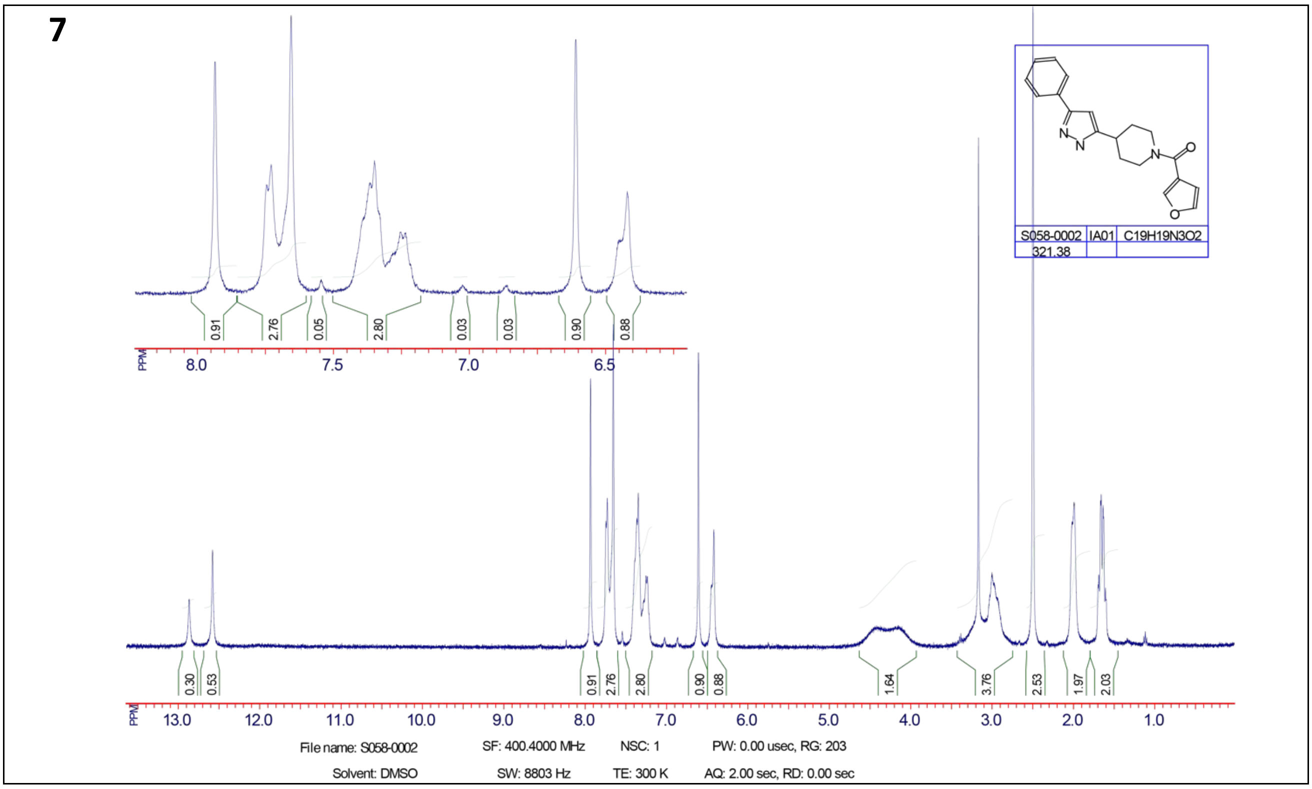
S058-0002 | IA01 | C19H19N3O2
321.38
0.91 2.76 0.05 2.80 0.03 0.03 0.90 0.88
PPM 8.0 7.5 7.0 6.5
0.30 0.53 0.91 2.76 2.80 0.90 0.88 1.64 3.76 2.53 1.97 2.03
PPM 13.0 12.0 11.0 10.0 9.0 8.0 7.0 6.0 5.0 4.0 3.0 2.0 1.0
File name: S058-0002
Solvent: DMSO
SF: 400.4000 MHz
SW: 8803 Hz
NSC: 1
TE: 300 K
PW: 0.00 usec, RG: 203
AQ: 2.00 sec, RD: 0.00 sec

8

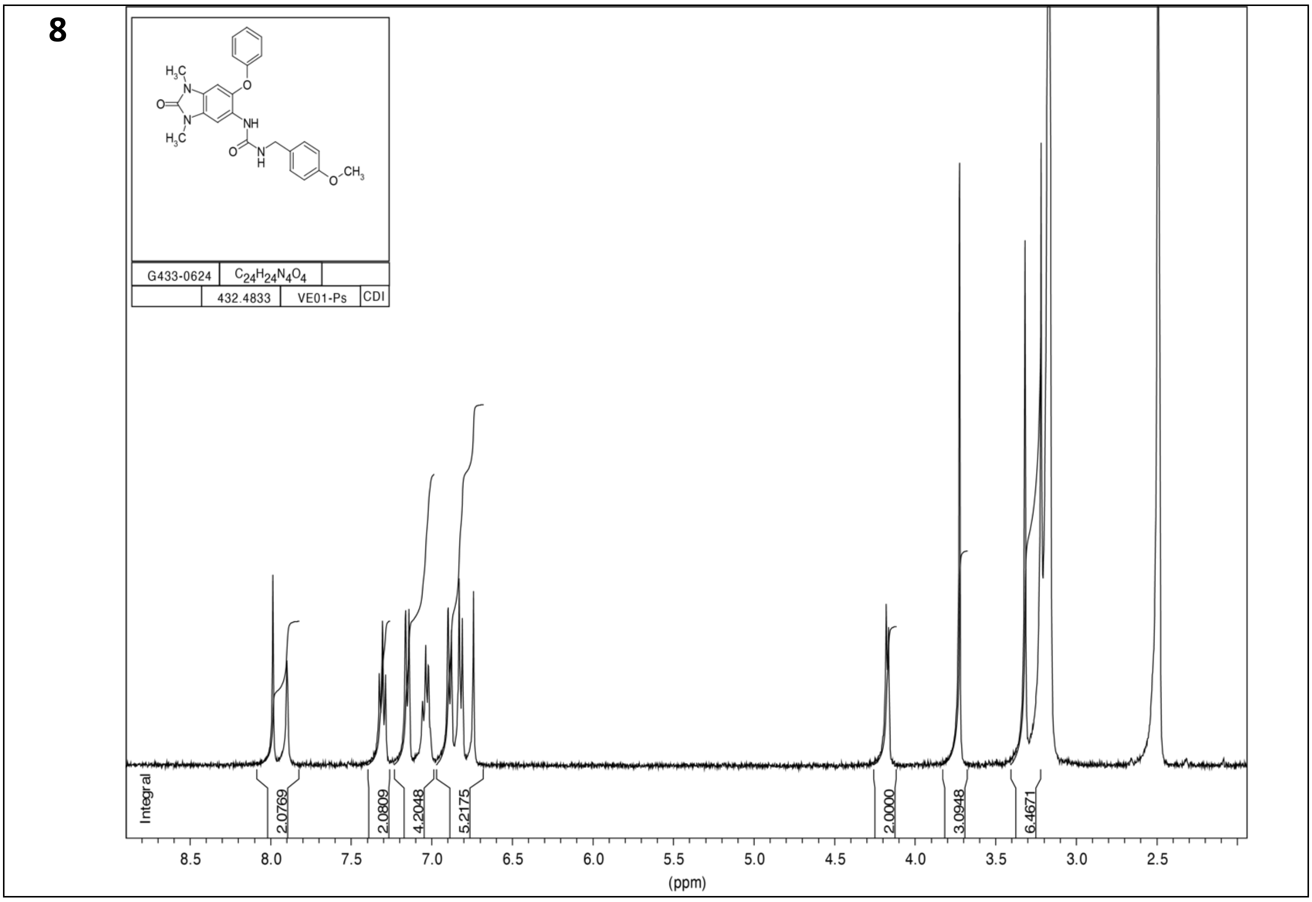
H3C
N
O
N
H3C
NH
O
N
H
O
CH3
G433-0624 | C24H24N4O4
432.4833 | VE01-Ps | CDI
Integral
2.0769 2.0809 4.2048 5.2175 2.0000 3.0948 6.4671
8.5 8.0 7.5 7.0 6.5 6.0 5.5 5.0 4.5 4.0 3.5 3.0 2.5
(ppm)

9

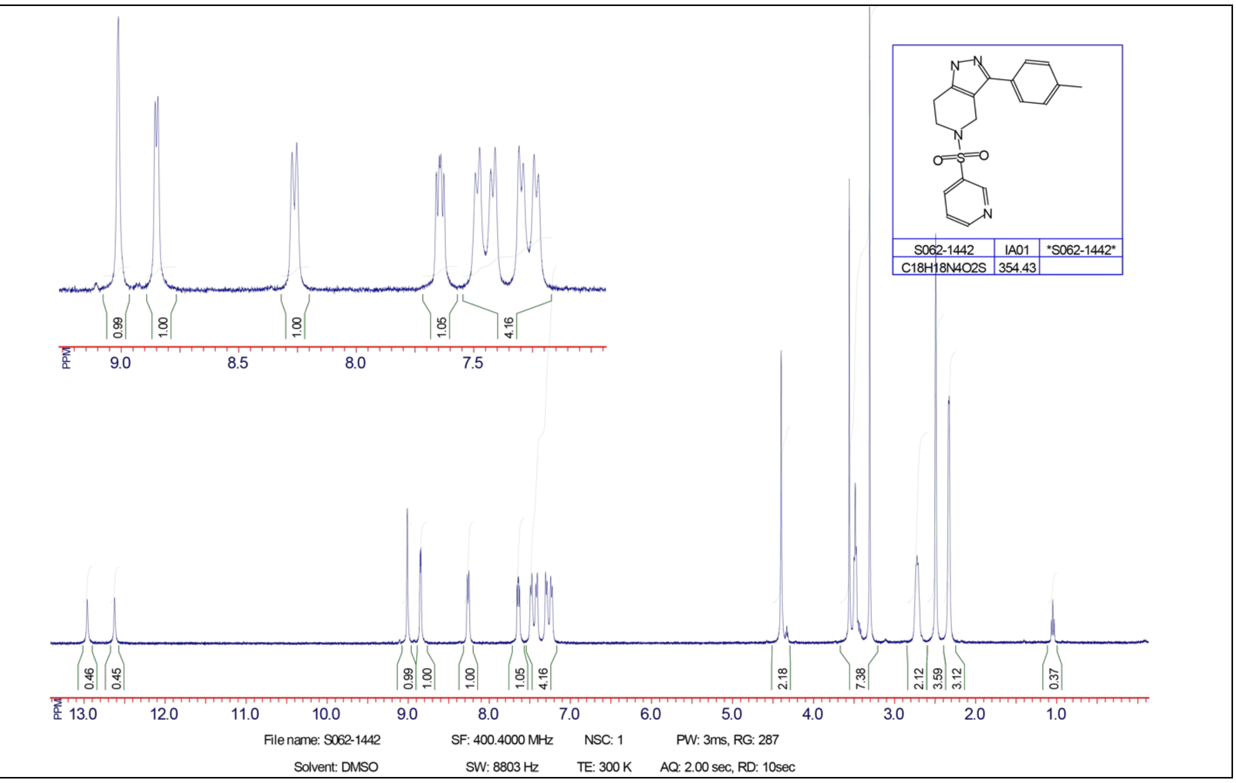
S062-1442
IA01
S062-1442
C18H18N4O2S
354.43
File name: S062-1442
SF: 400.4000 MHz
NSC: 1
PW: 3ms, RG: 287
Solvent: DMSO
SW: 8803 Hz
TE: 300 K
AQ: 2.00 sec, RD: 10sec

10

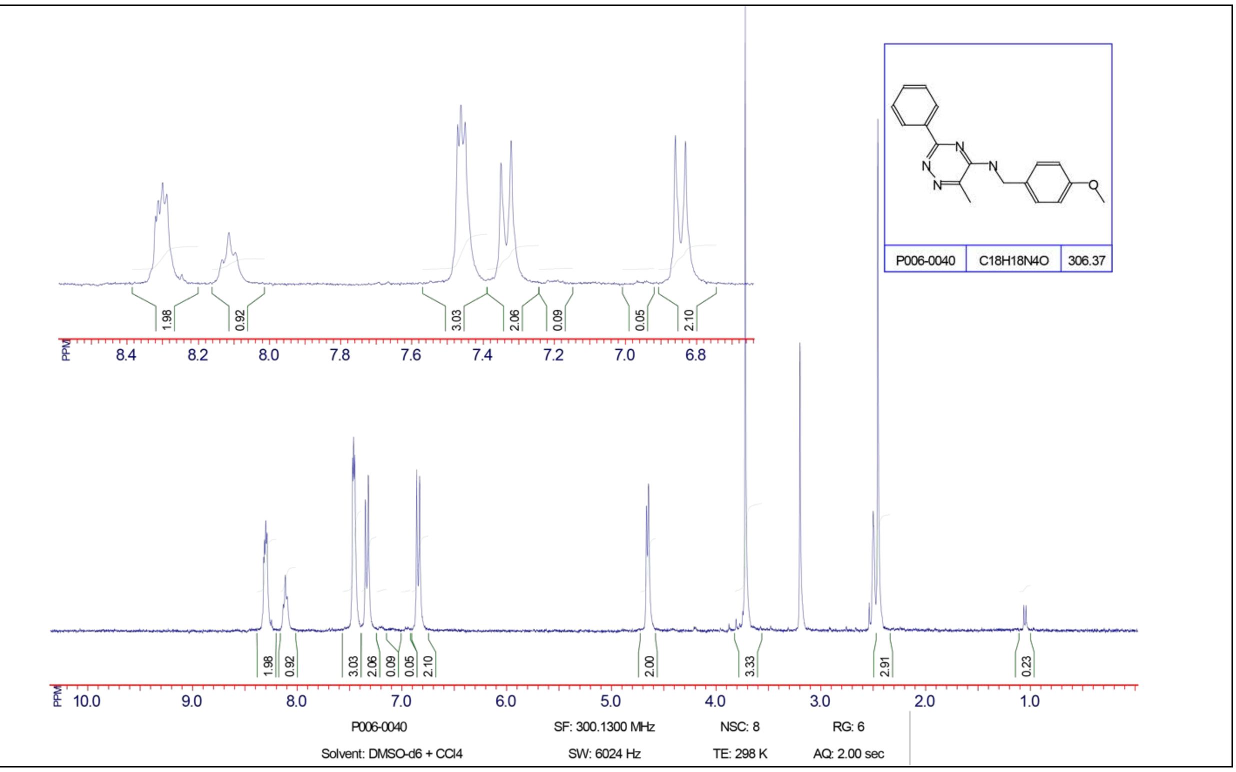
P006-0040
C18H18N4O
306.37
P006-0040
SF: 300.1300 MHz
NSC: 8
RG: 6
Solvent: DMSO-d6 + CCl4
SW: 6024 Hz
TE: 298 K
AQ: 2.00 sec

11

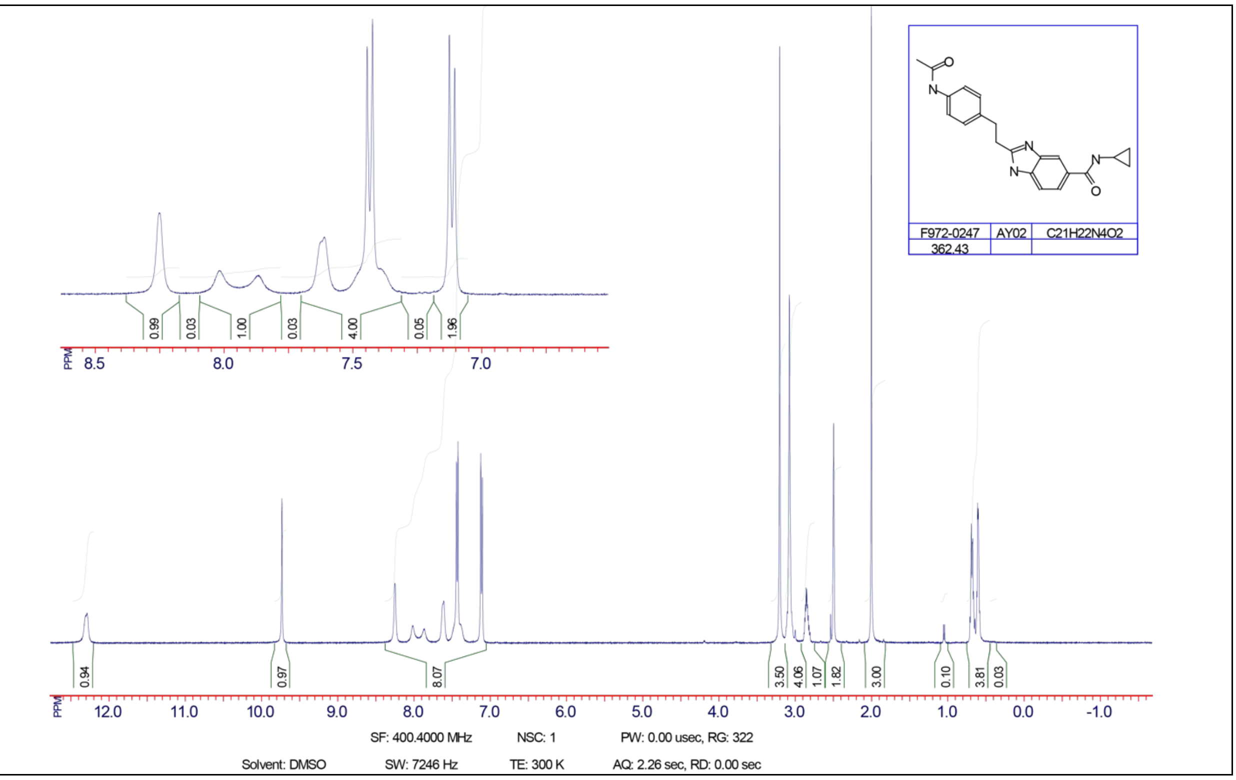
F972-0247
AY02
C21H22N4O2
362.43
PPM
SF: 400.4000 MHz
NSC: 1
PW: 0.00 usec, RG: 322
Solvent: DMSO
SW: 7246 Hz
TE: 300 K
AQ: 2.26 sec, RD: 0.00 sec

12

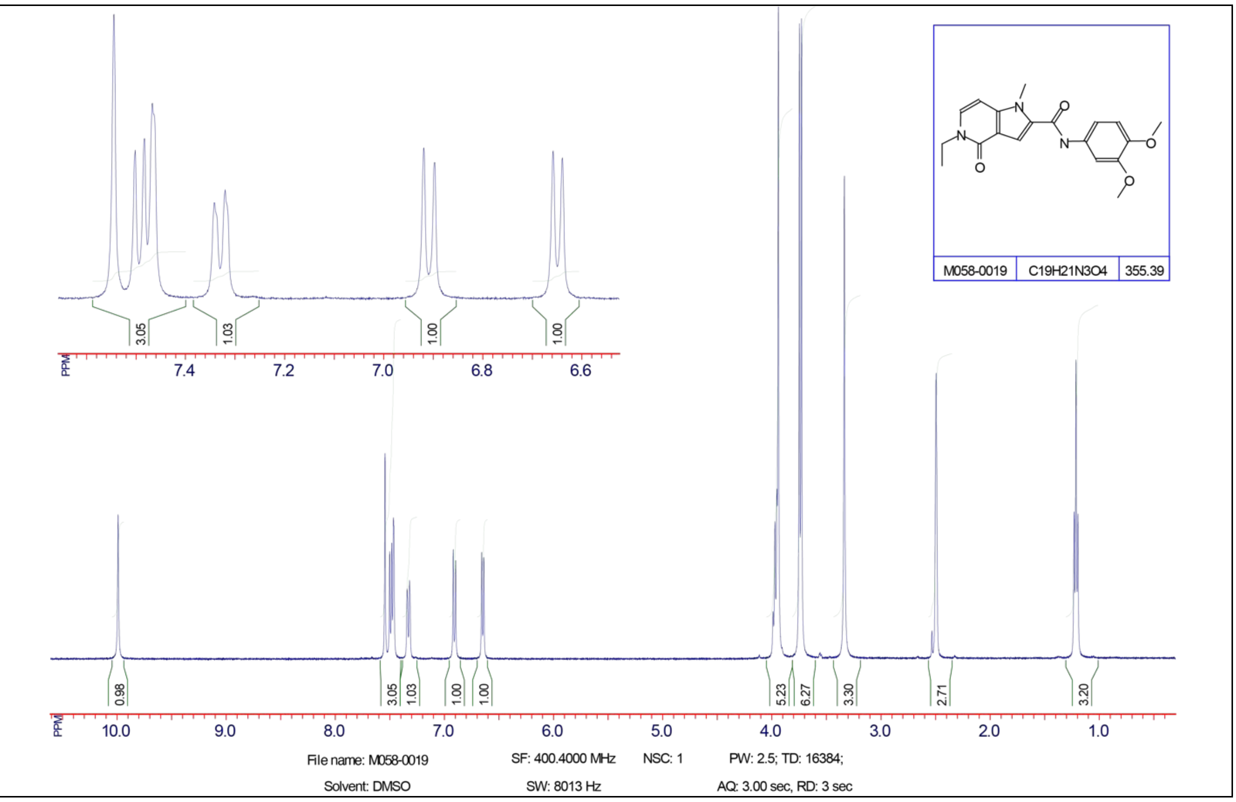
M058-0019
C19H21N3O4
355.39
PPM
File name: M058-0019
SF: 400.4000 MHz
NSC: 1
PW: 2.5; TD: 16384;
Solvent: DMSO
SW: 8013 Hz
AQ: 3.00 sec, RD: 3 sec

13

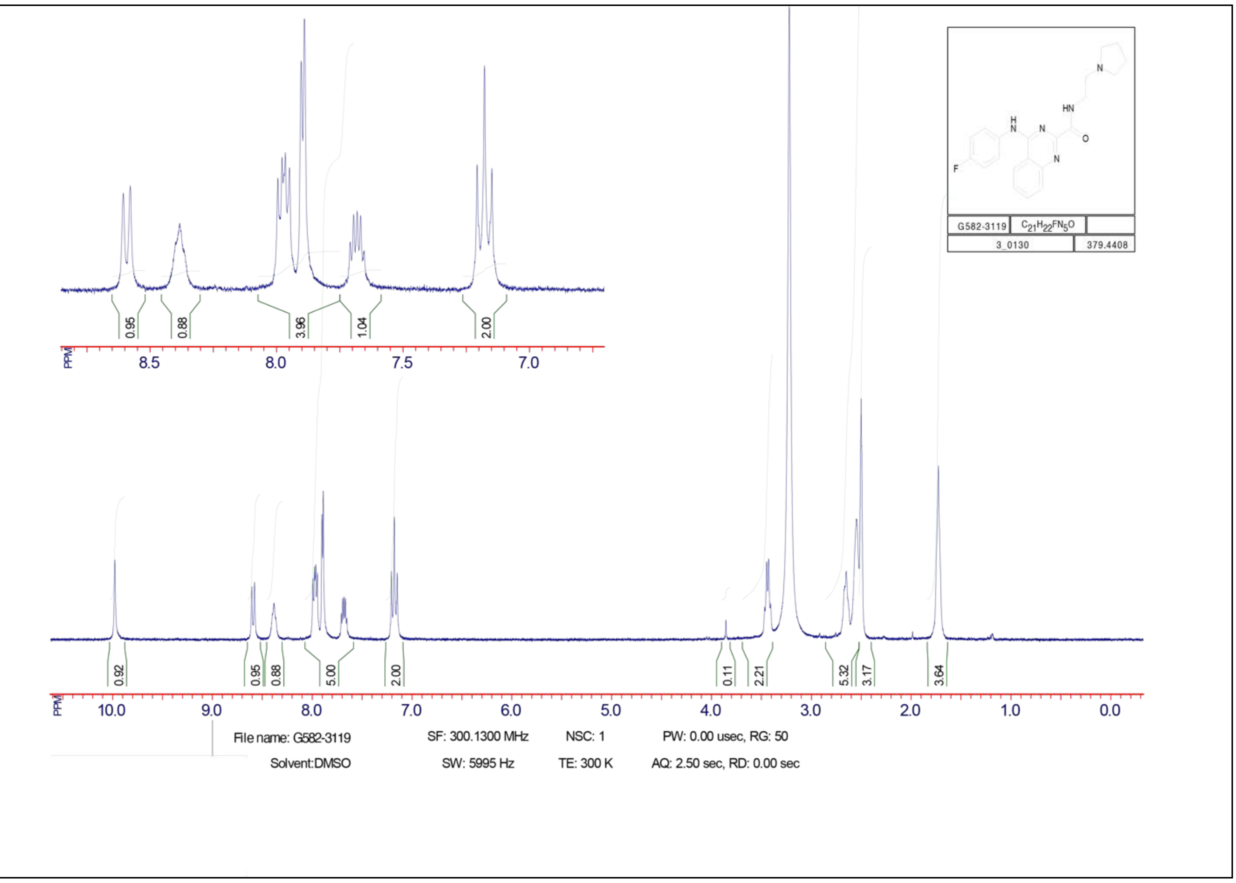
G582-3119
$C_{21}H_{22}FN_5O$
3_0130
379.4408
File name: G582-3119
Solvent:DMSO
SF: 300.1300 MHz
SW: 5995 Hz
NSC: 1
TE: 300 K
PW: 0.00 usec, RG: 50
AQ: 2.50 sec, RD: 0.00 sec

14

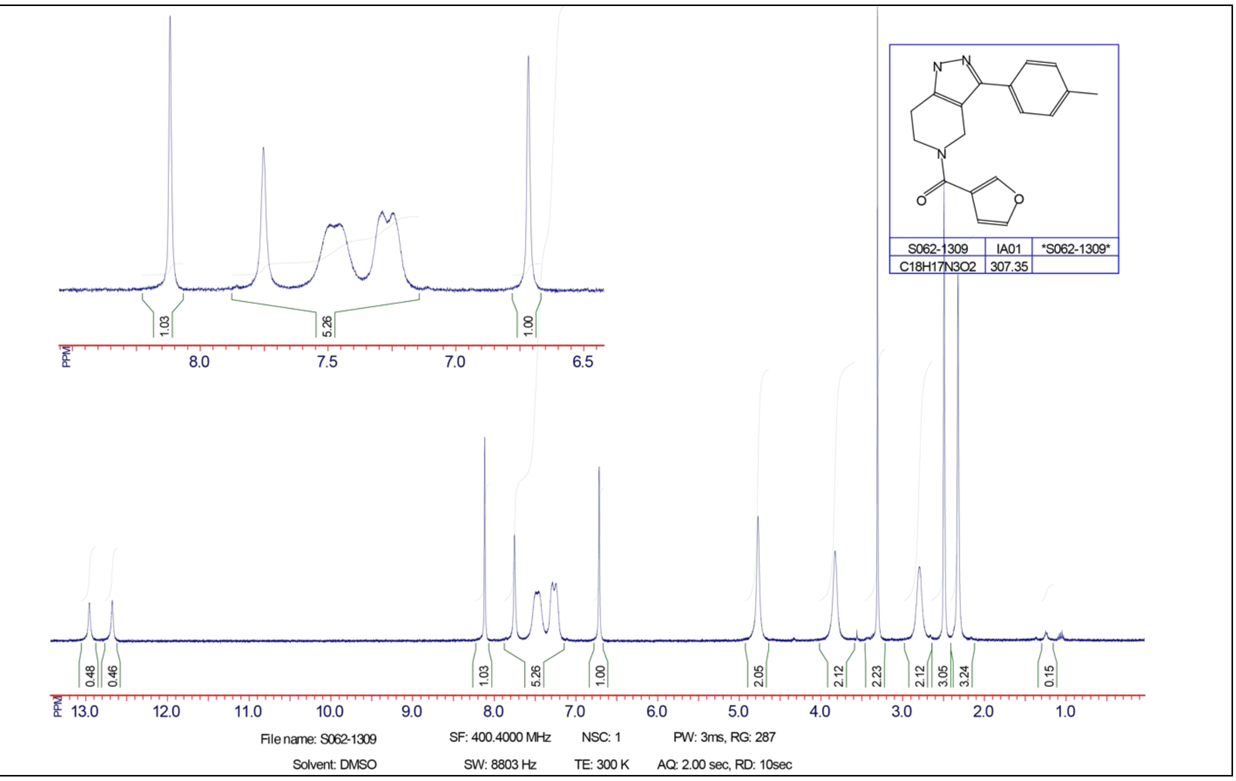
S062-1309
IA01
S062-1309
C18H17N3O2
307.35
File name: S062-1309
Solvent: DMSO
SF: 400.4000 MHz
SW: 8803 Hz
NSC: 1
TE: 300 K
PW: 3ms, RG: 287
AQ: 2.00 sec, RD: 10sec

18

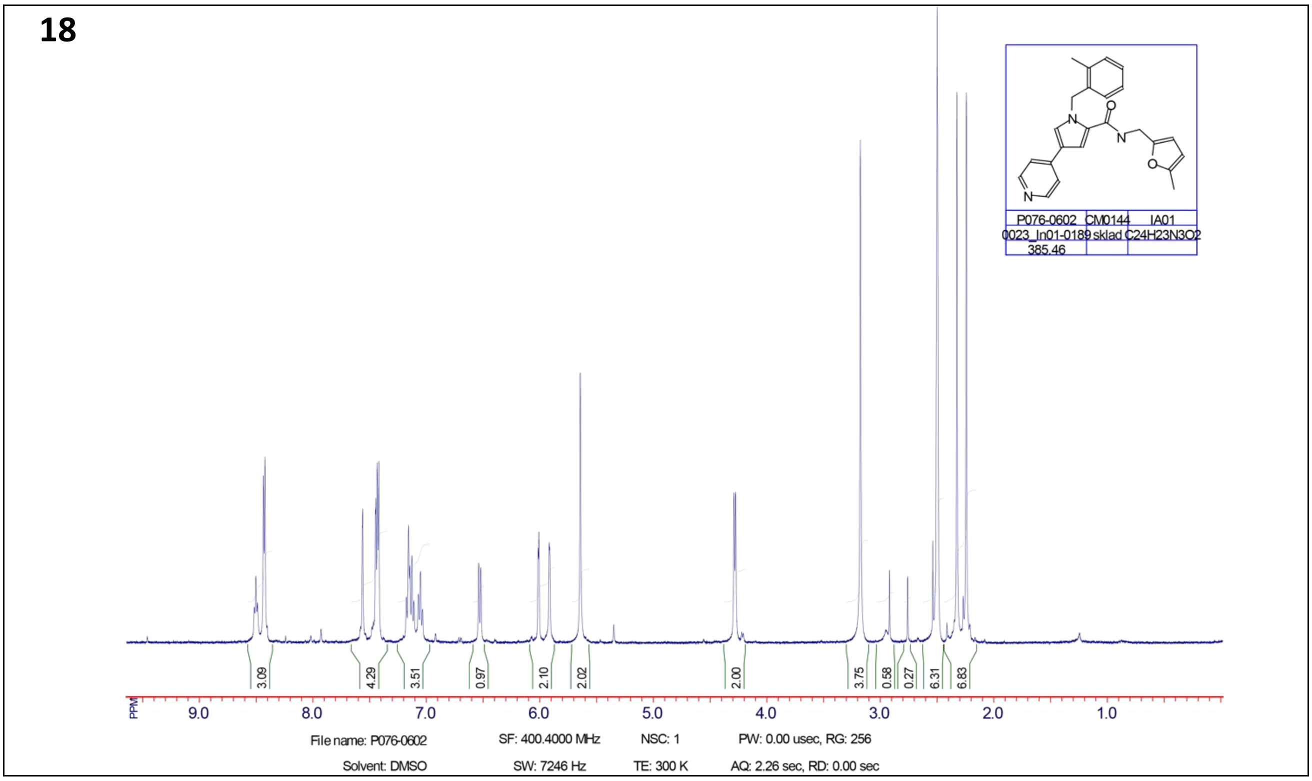
P076-0602 CM0144 IA01
0023_In01-0189 sklad C24H23N3O2
385.46
3.09
4.29
3.51
0.97
2.10
2.02
2.00
3.75
0.58
0.27
6.31
6.83
PPM 9.0 8.0 7.0 6.0 5.0 4.0 3.0 2.0 1.0
File name: P076-0602
SF: 400.4000 MHz
NSC: 1
PW: 0.00 usec, RG: 256
Solvent: DMSO
SW: 7246 Hz
TE: 300 K
AQ: 2.26 sec, RD: 0.00 sec

19

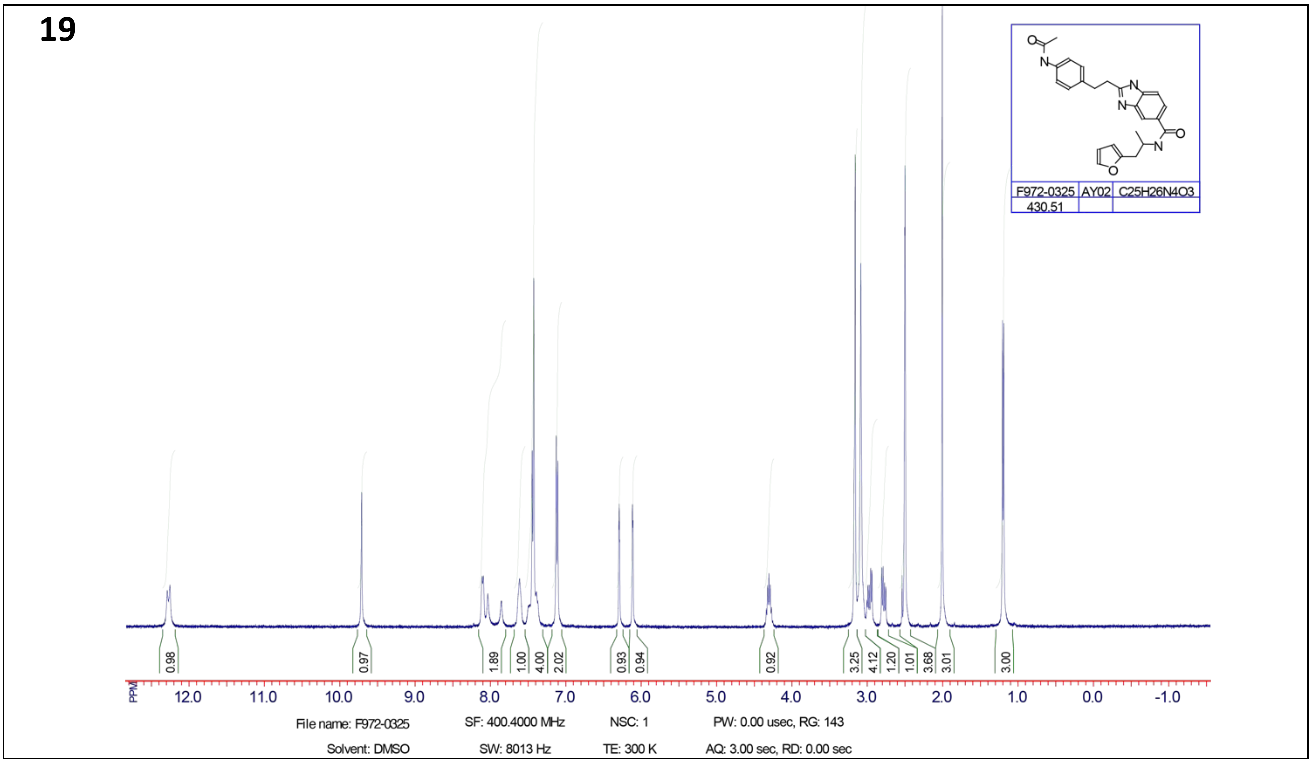
F972-0325 AY02 C25H26N4O3
430.51
0.98
0.97
1.89
1.00
4.00
2.02
0.93
0.94
0.92
3.25
4.12
1.20
1.01
3.68
3.01
3.00
PPM 12.0 11.0 10.0 9.0 8.0 7.0 6.0 5.0 4.0 3.0 2.0 1.0 0.0 -1.0
File name: F972-0325
SF: 400.4000 MHz
NSC: 1
PW: 0.00 usec, RG: 143
Solvent: DMSO
SW: 8013 Hz
TE: 300 K
AQ: 3.00 sec, RD: 0.00 sec

21

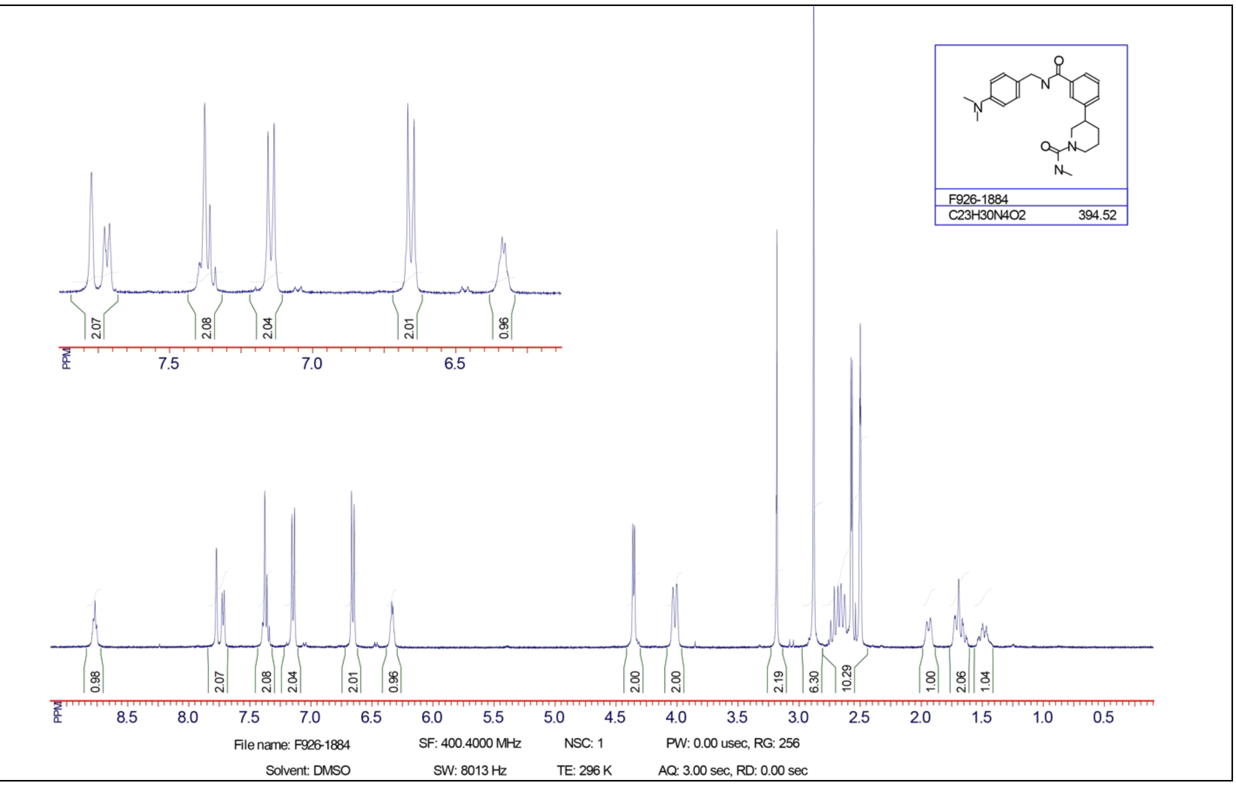
F926-1884
C23H30N4O2
394.52
File name: F926-1884
SF: 400.4000 MHz
NSC: 1
PW: 0.00 usec, RG: 256
Solvent: DMSO
SW: 8013 Hz
TE: 296 K
AQ: 3.00 sec, RD: 0.00 sec
PPM

22

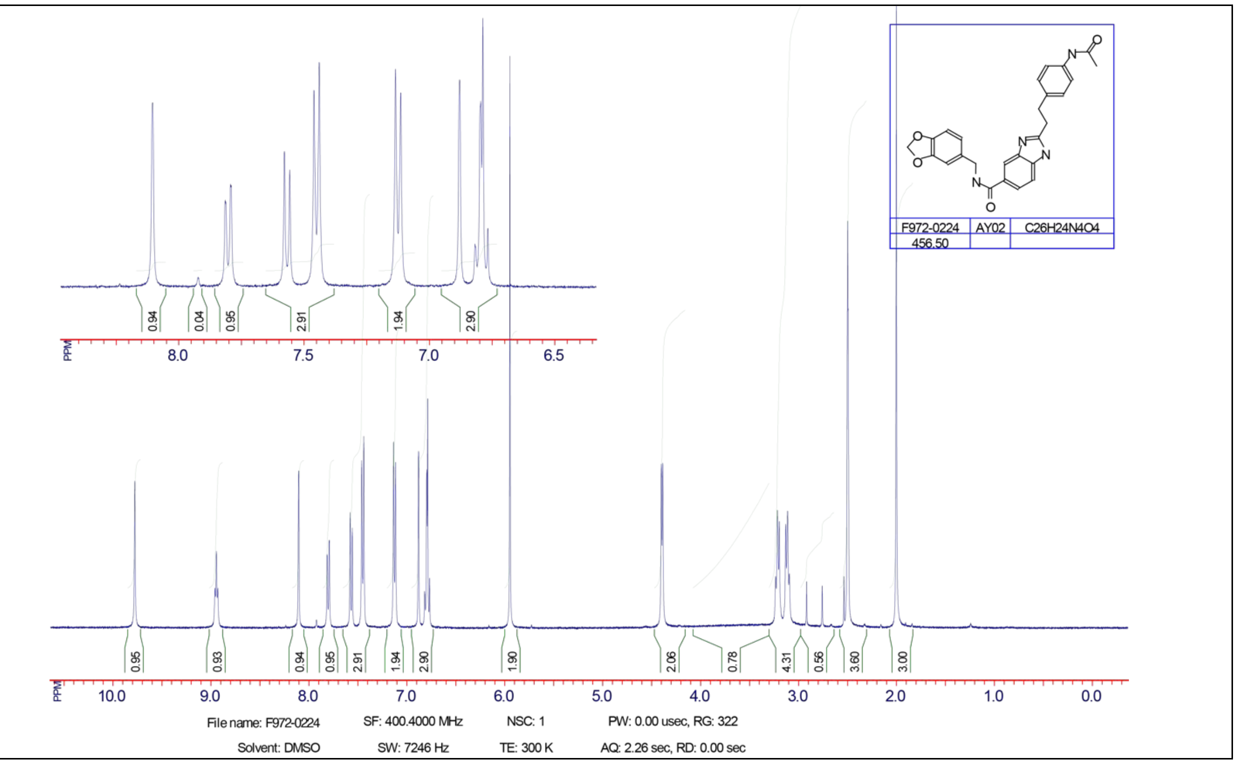
F972-0224
AY02
C26H24N4O4
456.50
File name: F972-0224
SF: 400.4000 MHz
NSC: 1
PW: 0.00 usec, RG: 322
Solvent: DMSO
SW: 7246 Hz
TE: 300 K
AQ: 2.26 sec, RD: 0.00 sec
PPM

23

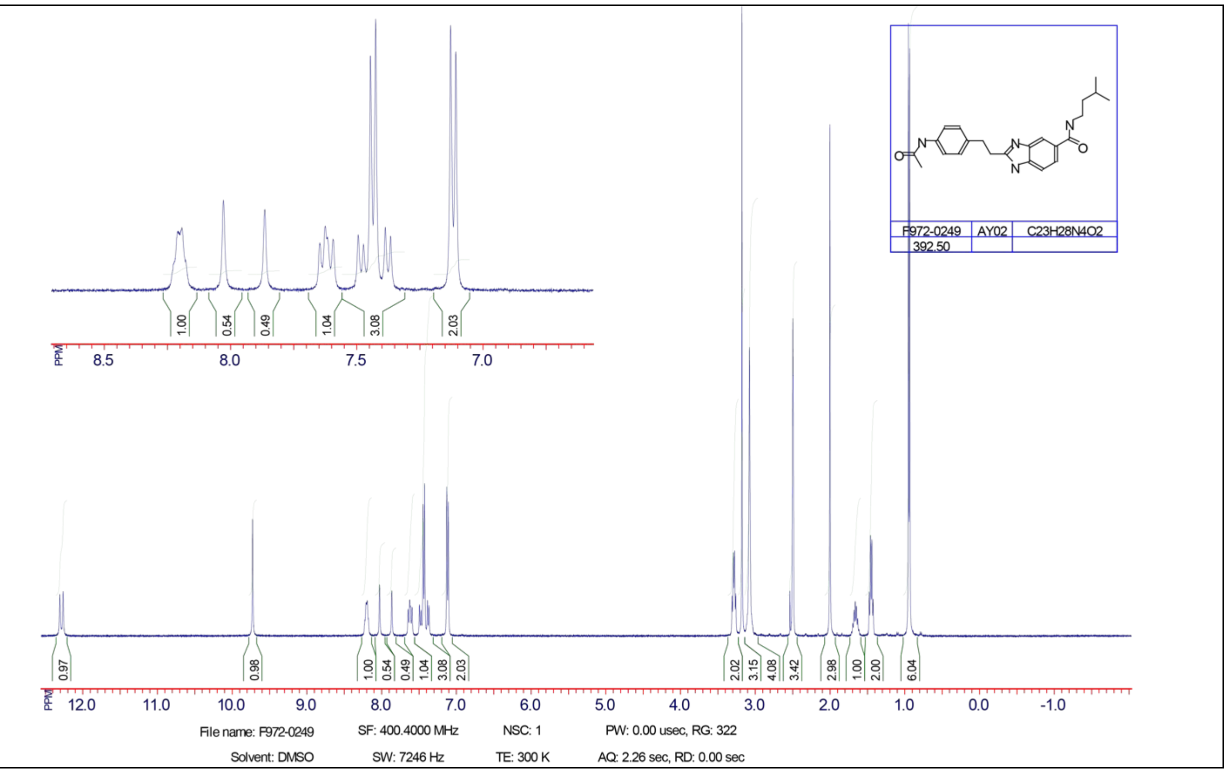
F972-0249
AY02
C23H28N4O2
392.50
PPM
File name: F972-0249
Solvent: DMSO
SF: 400.4000 MHz
SW: 7246 Hz
NSC: 1
TE: 300 K
PW: 0.00 usec, RG: 322
AQ: 2.26 sec, RD: 0.00 sec

24

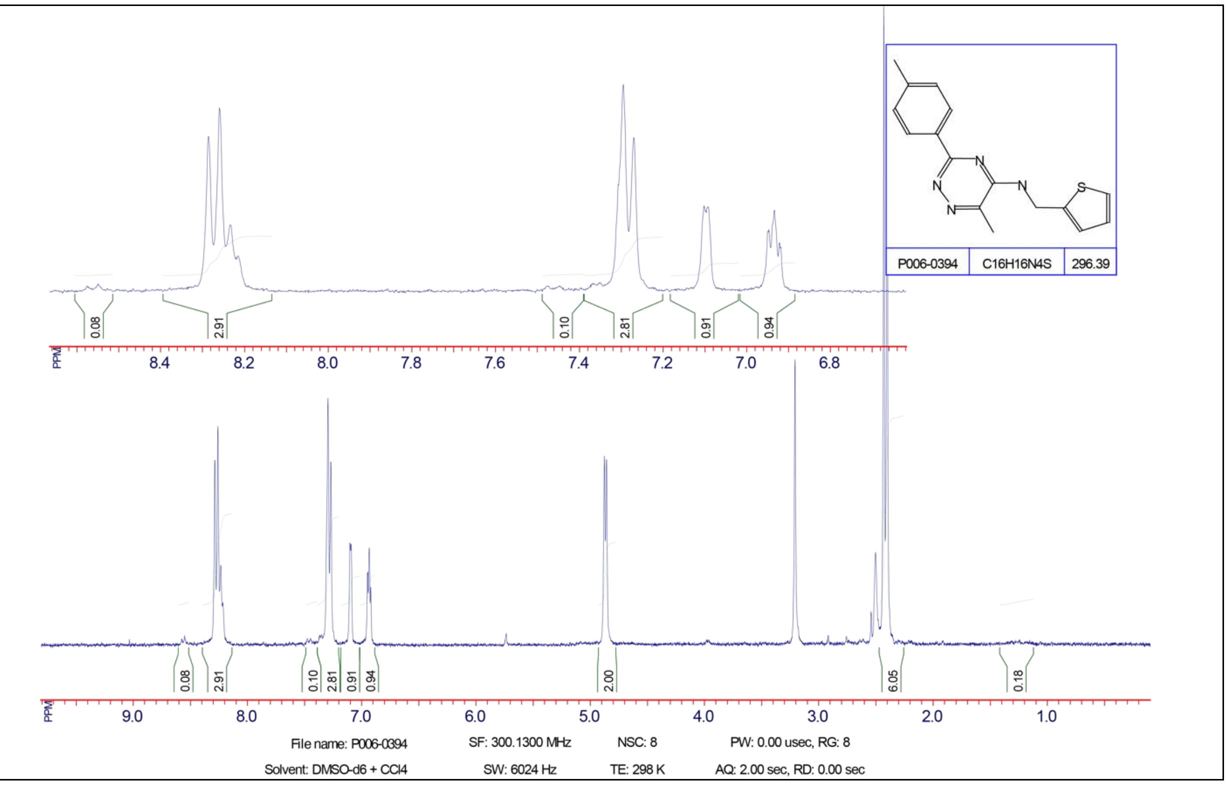
P006-0394
C16H16N4S
296.39
PPM
File name: P006-0394
Solvent: DMSO-d6 + CCl4
SF: 300.1300 MHz
SW: 6024 Hz
NSC: 8
TE: 298 K
PW: 0.00 usec, RG: 8
AQ: 2.00 sec, RD: 0.00 sec

24a

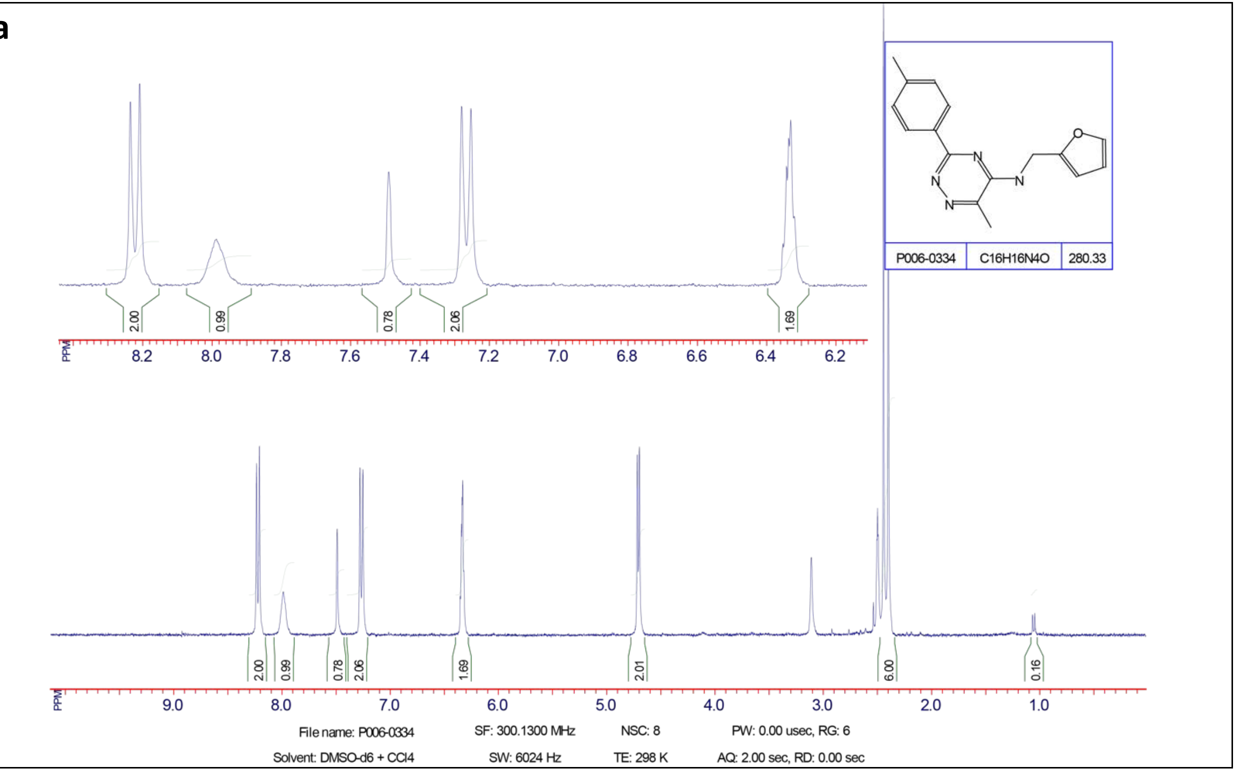
P006-0334
C16H16N4O
280.33
File name: P006-0334
SF: 300.1300 MHz
NSC: 8
PW: 0.00 usec, RG: 6
Solvent: DMSO-d6 + CCl4
SW: 6024 Hz
TE: 298 K
AQ: 2.00 sec, RD: 0.00 sec

24b

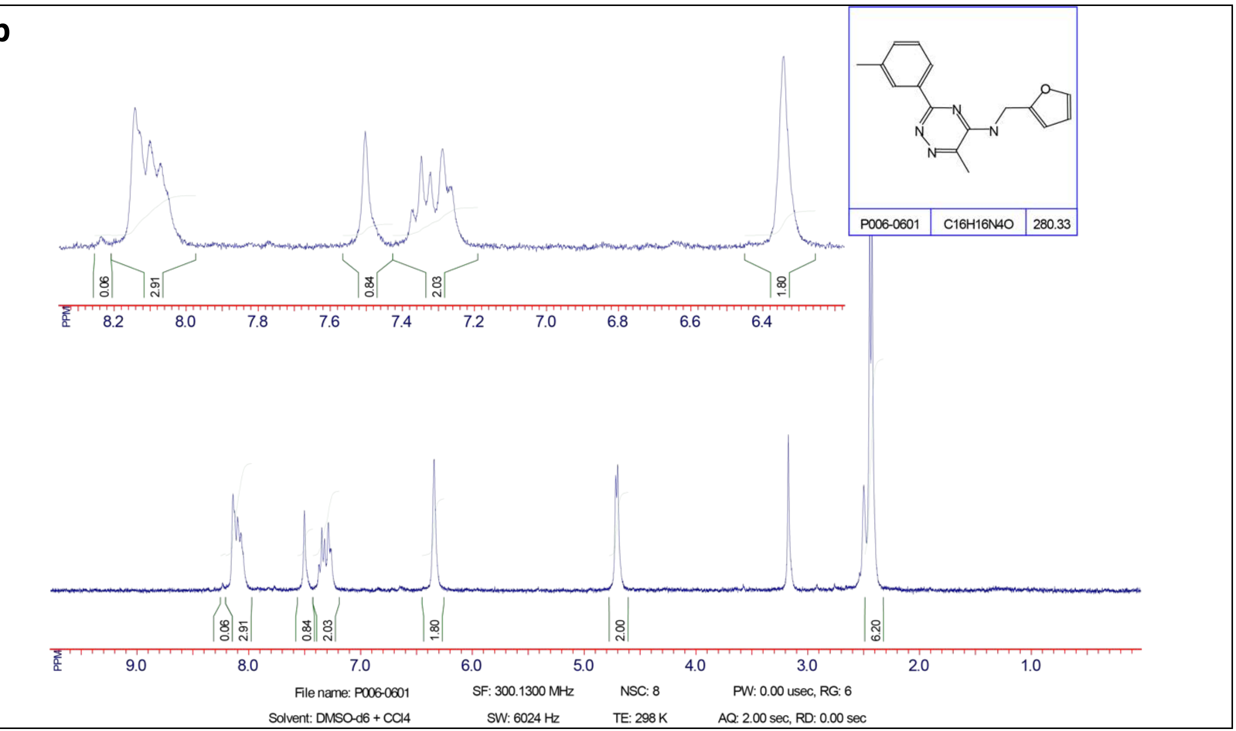
P006-0601
C16H16N4O
280.33
File name: P006-0601
SF: 300.1300 MHz
NSC: 8
PW: 0.00 usec, RG: 6
Solvent: DMSO-d6 + CCl4
SW: 6024 Hz
TE: 298 K
AQ: 2.00 sec, RD: 0.00 sec

24c

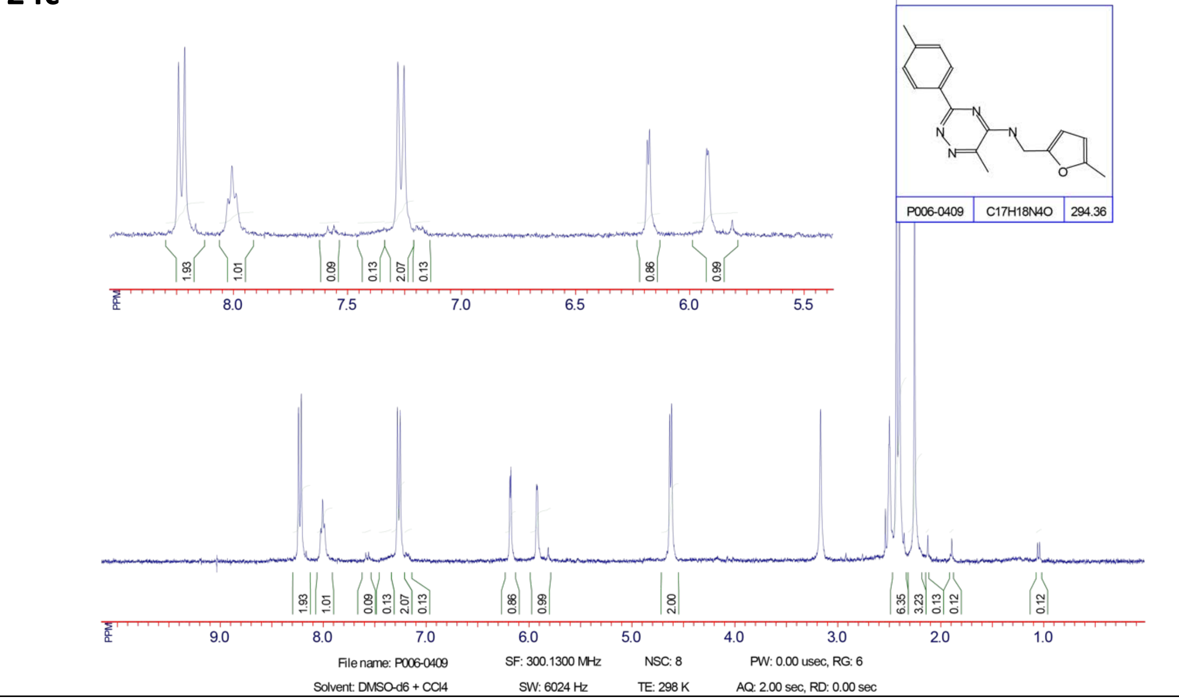

24d

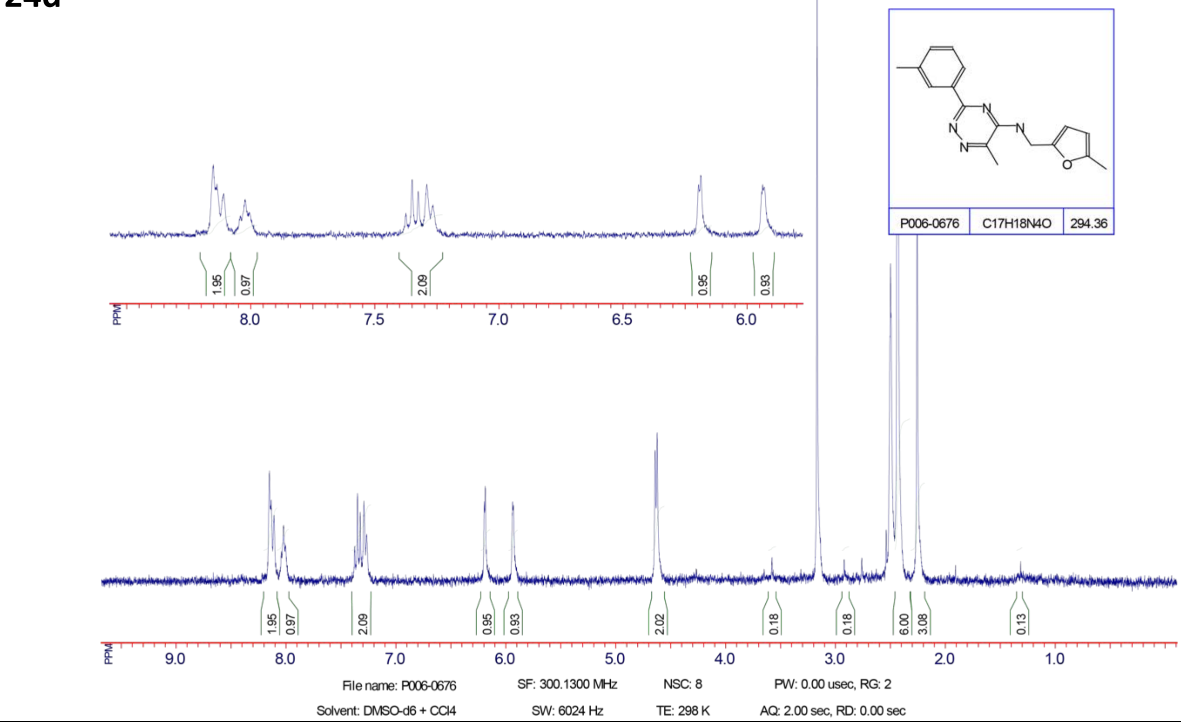

24e See compound **10**

24g

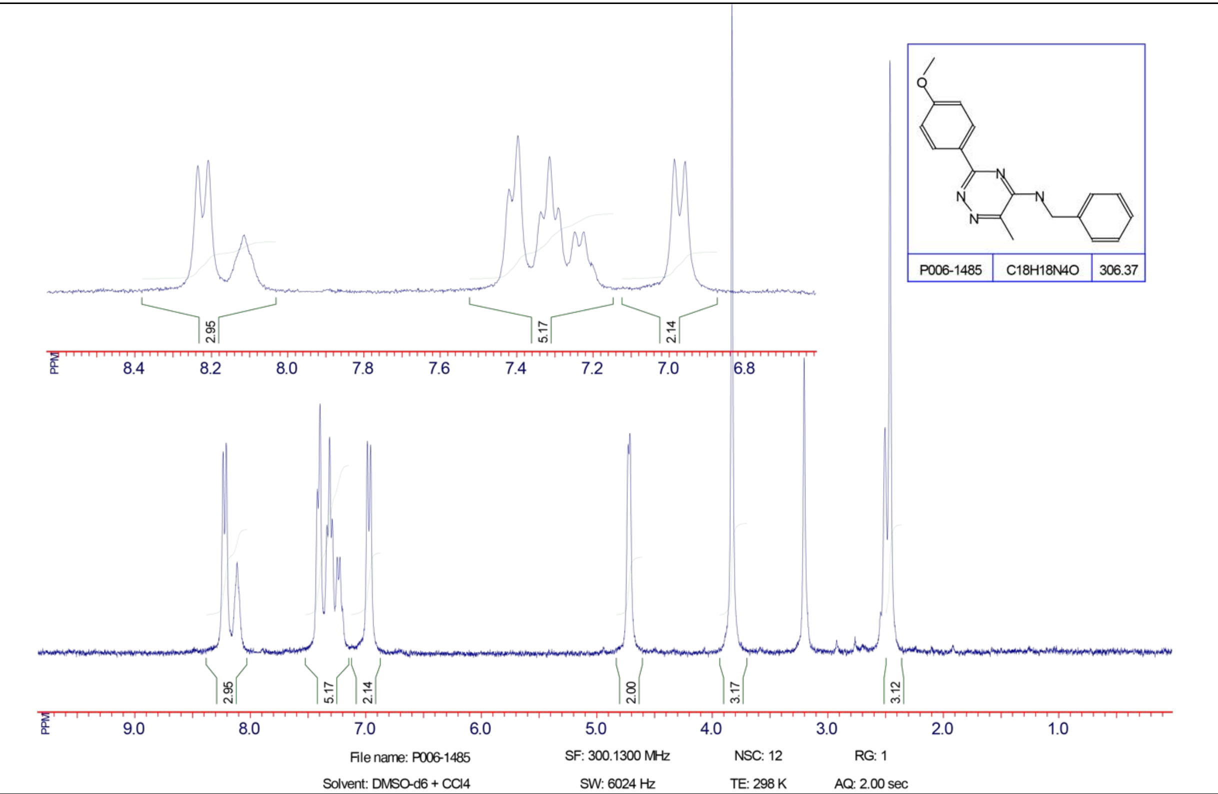
P006-1485
C18H18N4O
306.37
2.95
5.17
2.14
PPM
8.4
8.2
8.0
7.8
7.6
7.4
7.2
7.0
6.8
2.00
3.17
3.12
9.0
6.0
5.0
4.0
3.0
2.0
1.0
File name: P006-1485
SF: 300.1300 MHz
NSC: 12
RG: 1
Solvent: DMSO-d6 + CCl4
SW: 6024 Hz
TE: 298 K
AQ: 2.00 sec

24i

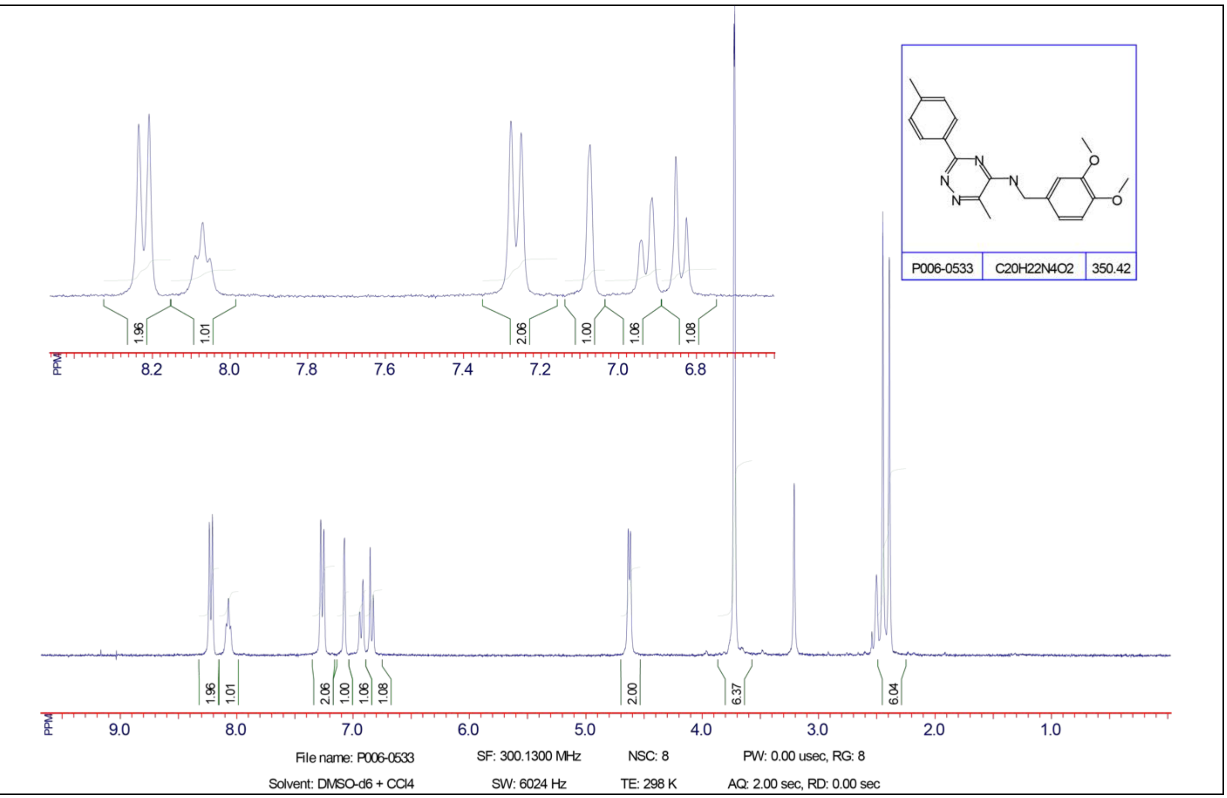
P006-0533
C20H22N4O2
350.42
1.96
1.01
2.06
1.00
1.06
1.08
PPM
8.2
8.0
7.8
7.6
7.4
7.2
7.0
6.8
2.00
6.37
6.04
9.0
6.0
5.0
4.0
3.0
2.0
1.0
File name: P006-0533
SF: 300.1300 MHz
NSC: 8
PW: 0.00 usec, RG: 8
Solvent: DMSO-d6 + CCl4
SW: 6024 Hz
TE: 298 K
AQ: 2.00 sec, RD: 0.00 sec

24j

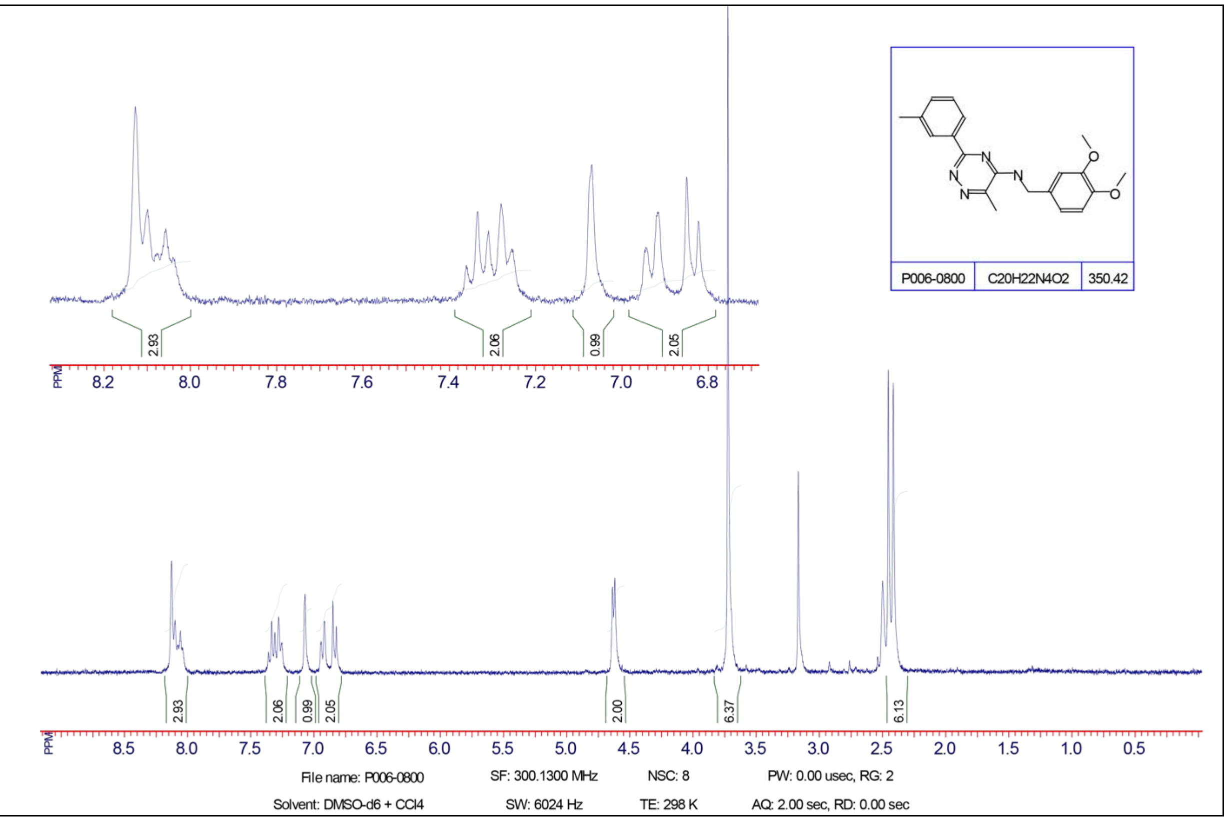
P006-0800
C20H22N4O2
350.42
2.93
2.06
0.99
2.05
2.00
6.37
6.13
PPM
File name: P006-0800
SF: 300.1300 MHz
NSC: 8
PW: 0.00 usec, RG: 2
Solvent: DMSO-d6 + CCl4
SW: 6024 Hz
TE: 298 K
AQ: 2.00 sec, RD: 0.00 sec

25

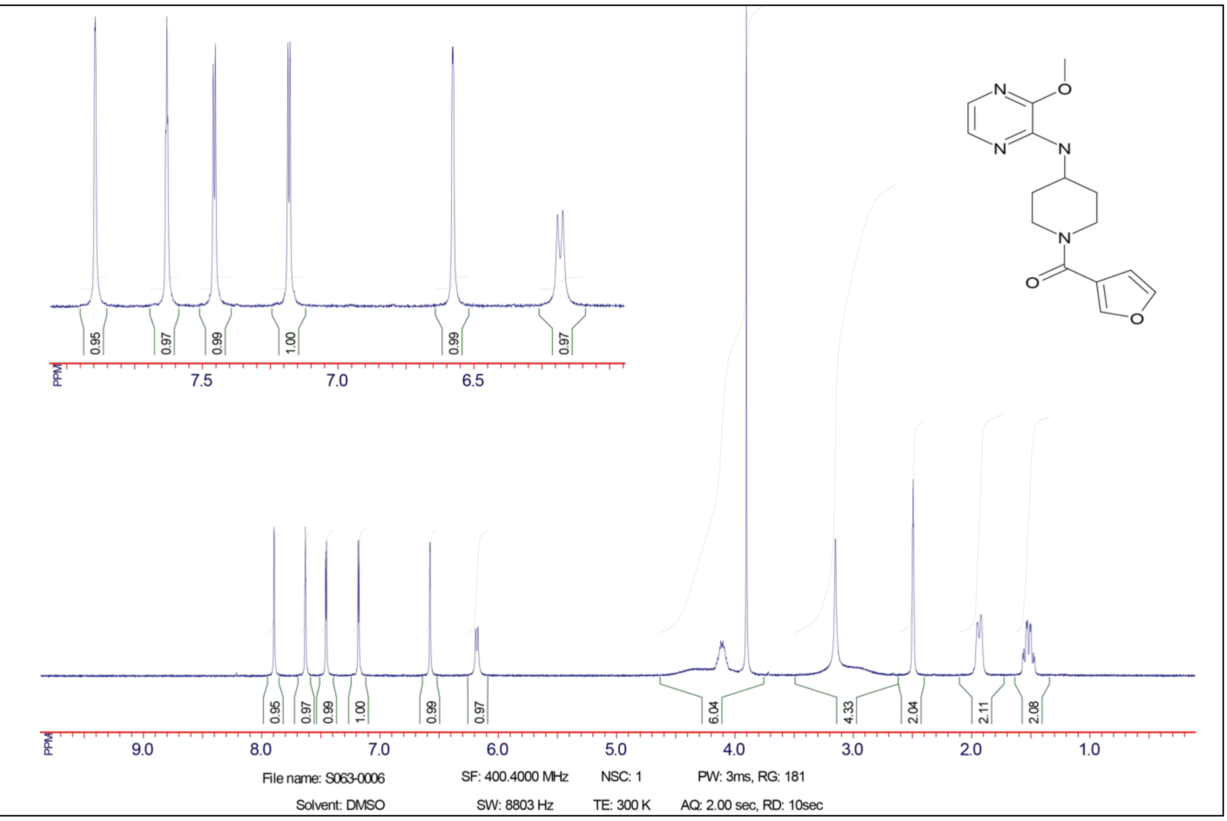
0.95
0.97
0.99
1.00
0.99
0.97
6.04
4.33
2.04
2.11
2.08
PPM
File name: S063-0006
SF: 400.4000 MHz
NSC: 1
PW: 3ms, RG: 181
Solvent: DMSO
SW: 8803 Hz
TE: 300 K
AQ: 2.00 sec, RD: 10sec

26

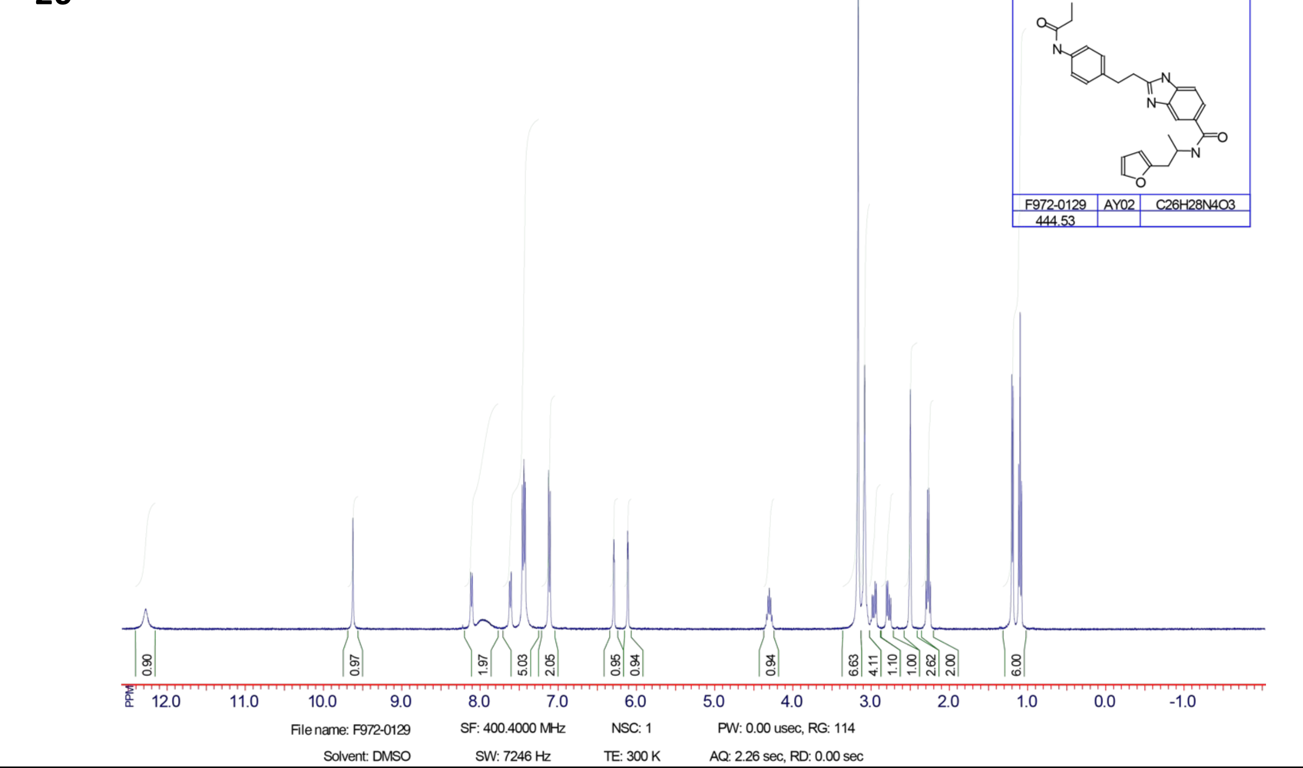
F972-0129
AY02
C26H28N4O3
444.53
0.90
0.97
1.97
5.03
2.05
0.95
0.94
0.94
6.63
4.11
1.10
1.00
2.62
2.00
6.00
PPM
12.0
11.0
10.0
9.0
8.0
7.0
6.0
5.0
4.0
3.0
2.0
1.0
0.0
-1.0
File name: F972-0129
SF: 400.4000 MHz
NSC: 1
PW: 0.00 usec, RG: 114
Solvent: DMSO
SW: 7246 Hz
TE: 300 K
AQ: 2.26 sec, RD: 0.00 sec

27

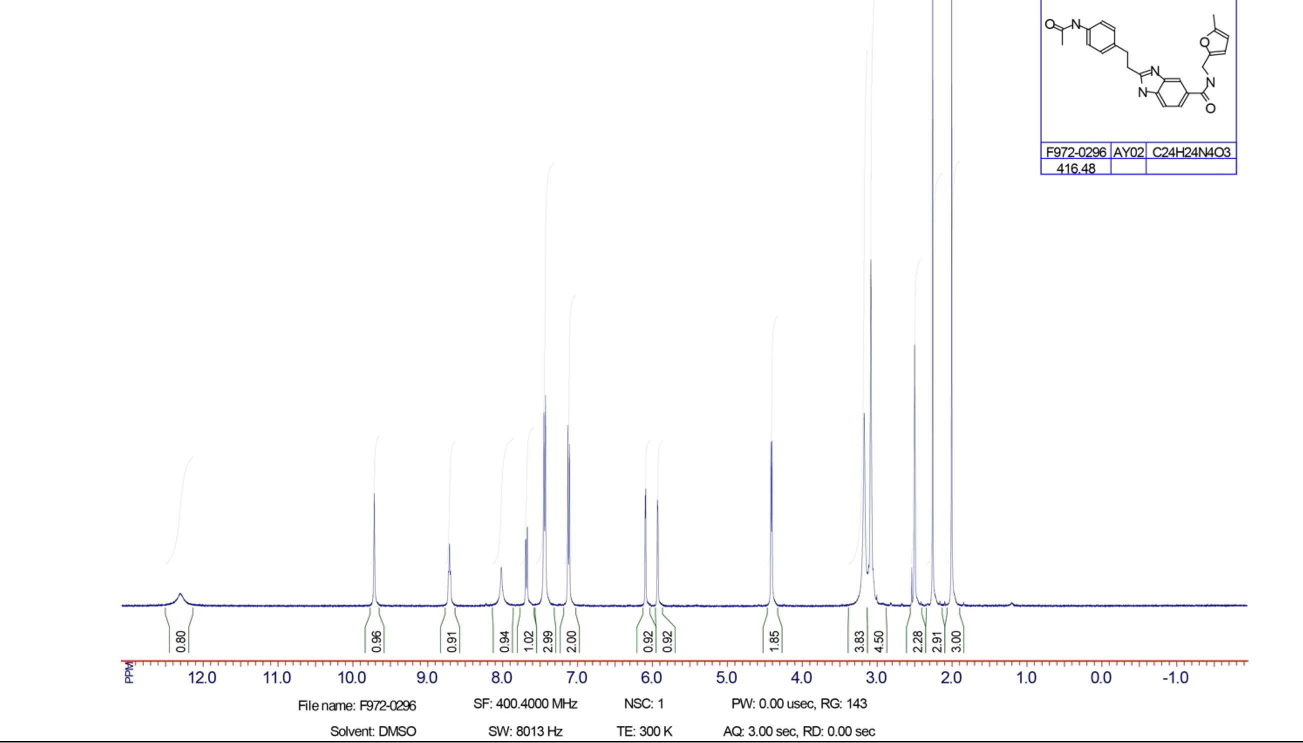
F972-0296
AY02
C24H24N4O3
416.48
0.80
0.96
0.91
0.94
1.02
2.99
2.00
0.92
0.92
1.85
3.83
4.50
2.28
2.91
3.00
PPM
12.0
11.0
10.0
9.0
8.0
7.0
6.0
5.0
4.0
3.0
2.0
1.0
0.0
-1.0
File name: F972-0296
SF: 400.4000 MHz
NSC: 1
PW: 0.00 usec, RG: 143
Solvent: DMSO
SW: 8013 Hz
TE: 300 K
AQ: 3.00 sec, RD: 0.00 sec

28

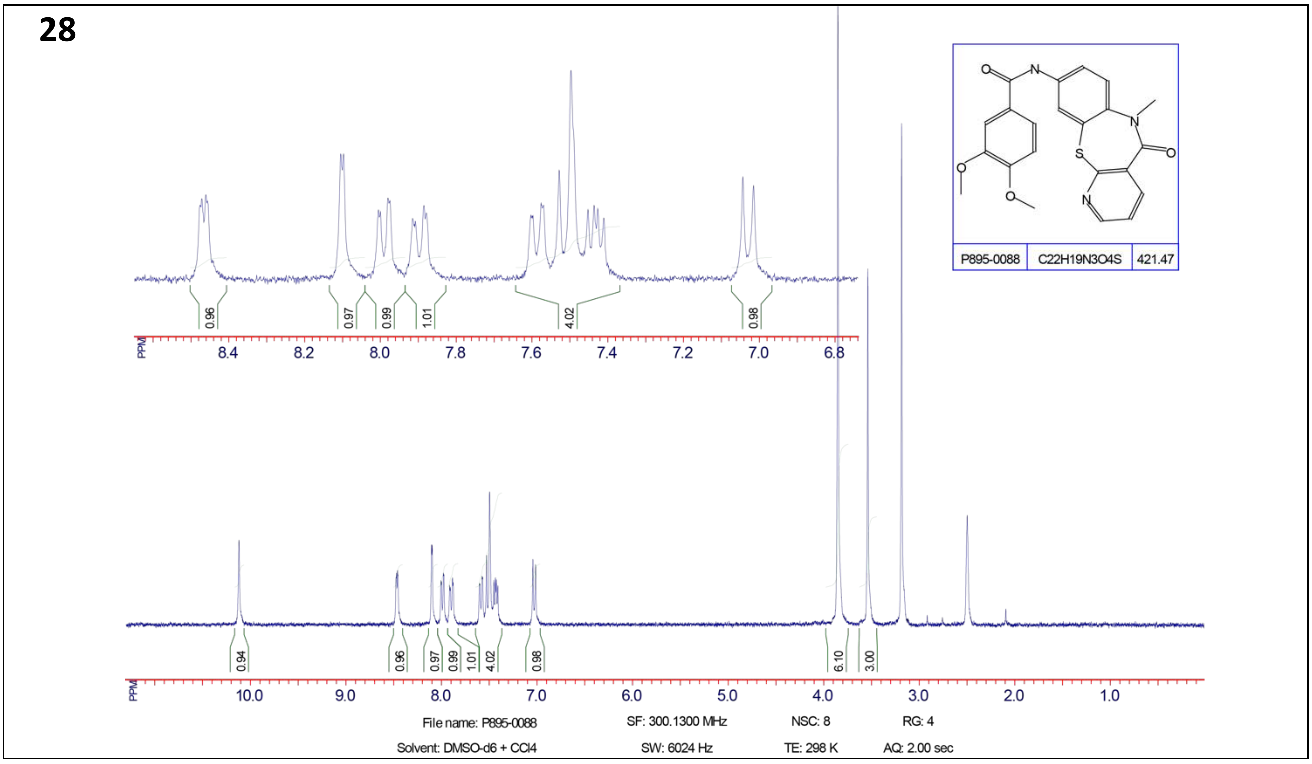

P895-0088
C22H19N3O4S
421.47
File name: P895-0088
SF: 300.1300 MHz
NSC: 8
RG: 4
Solvent: DMSO-d6 + CCl4
SW: 6024 Hz
TE: 298 K
AQ: 2.00 sec

30

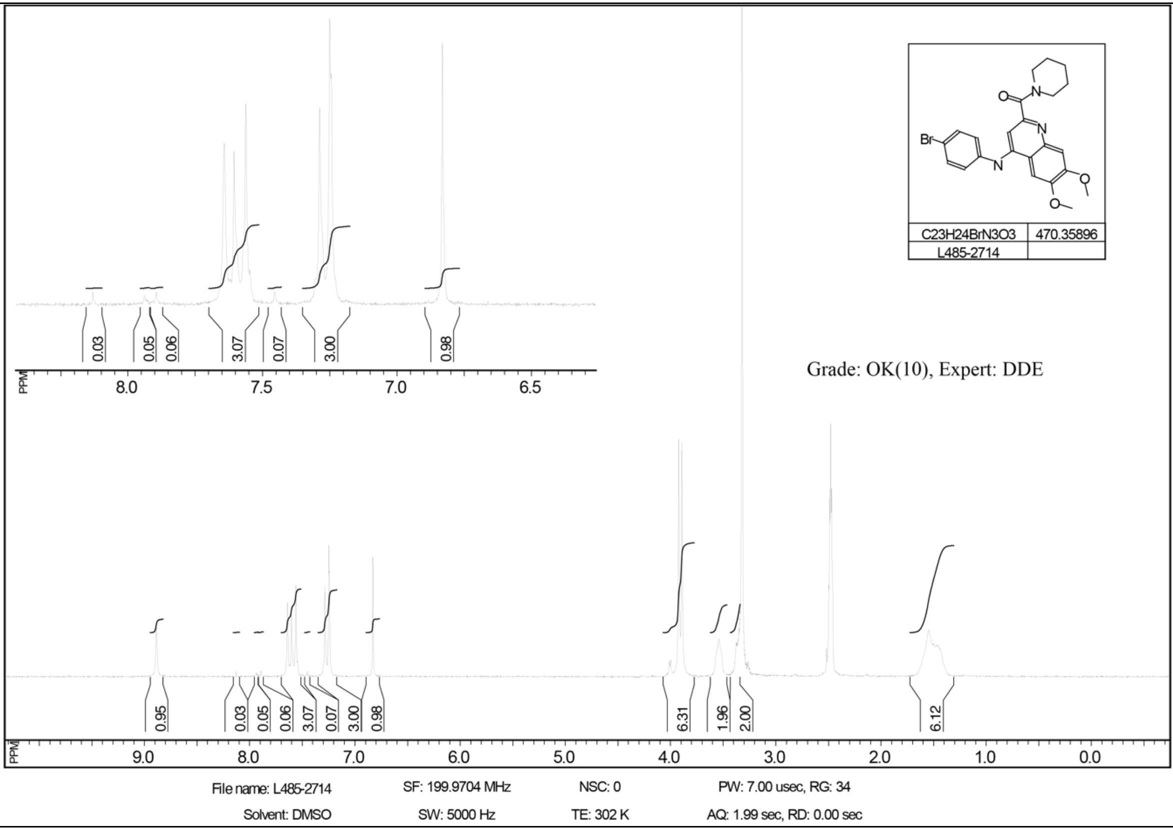

C23H24BrN3O3
470.35896
L485-2714
Grade: OK(10), Expert: DDE
File name: L485-2714
SF: 199.9704 MHz
NSC: 0
PW: 7.00 usec, RG: 34
Solvent: DMSO
SW: 5000 Hz
TE: 302 K
AQ: 1.99 sec, RD: 0.00 sec

32

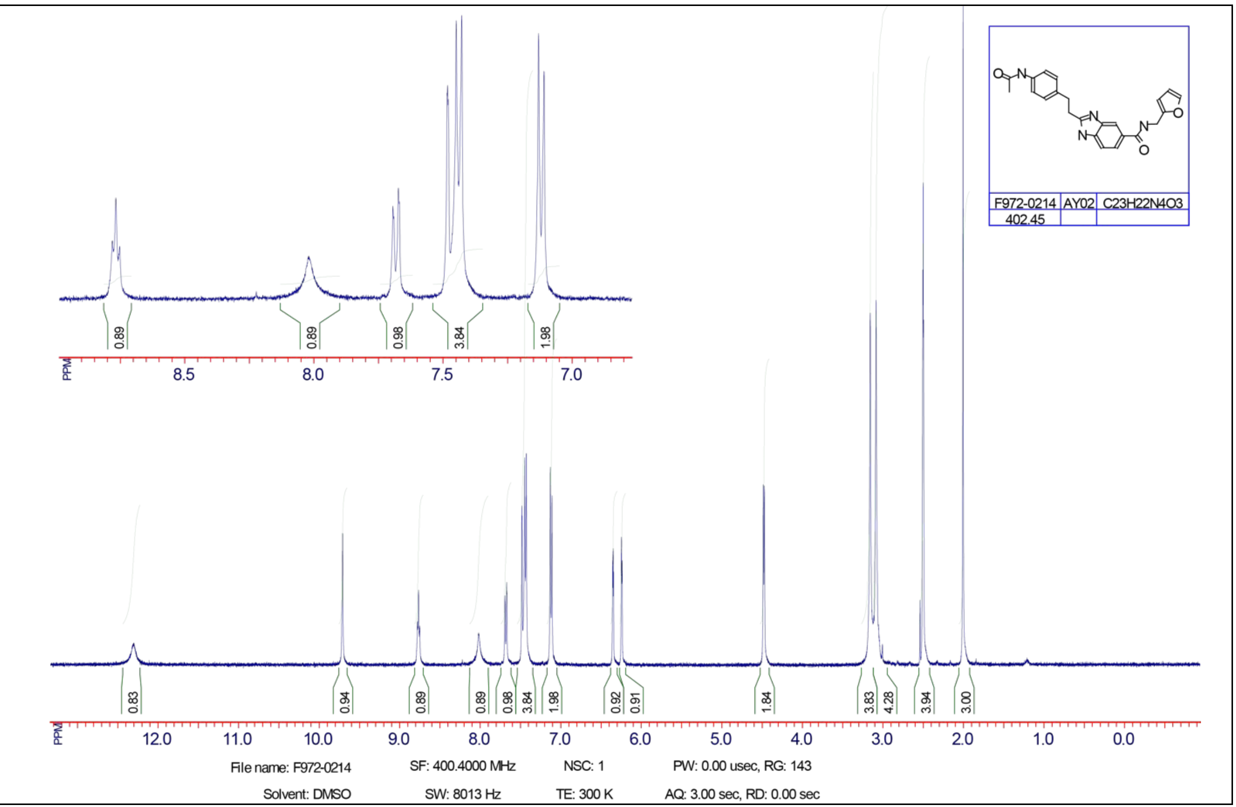
F972-0214
AY02
C23H22N4O3
402.45
File name: F972-0214
SF: 400.4000 MHz
NSC: 1
PW: 0.00 usec, RG: 143
Solvent: DMSO
SW: 8013 Hz
TE: 300 K
AQ: 3.00 sec, RD: 0.00 sec

33

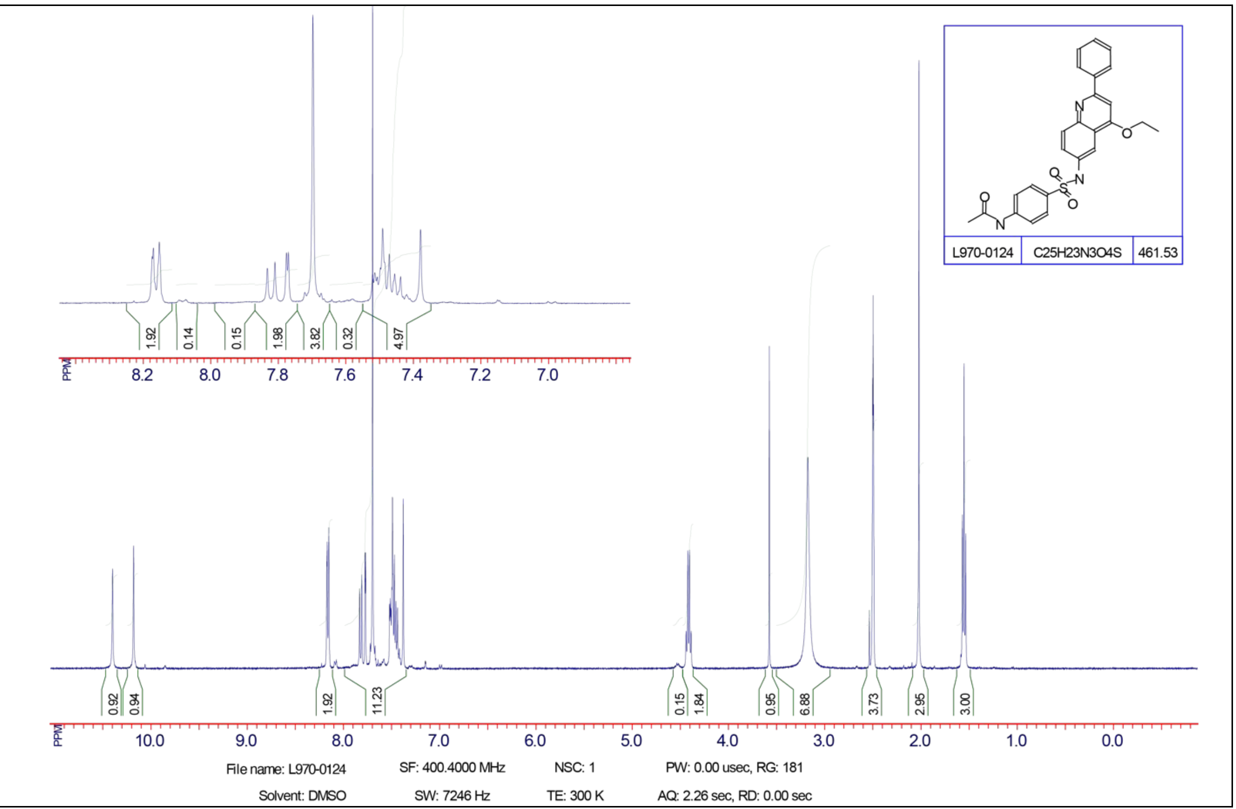
L970-0124
C25H23N3O4S
461.53
File name: L970-0124
SF: 400.4000 MHz
NSC: 1
PW: 0.00 usec, RG: 181
Solvent: DMSO
SW: 7246 Hz
TE: 300 K
AQ: 2.26 sec, RD: 0.00 sec

34

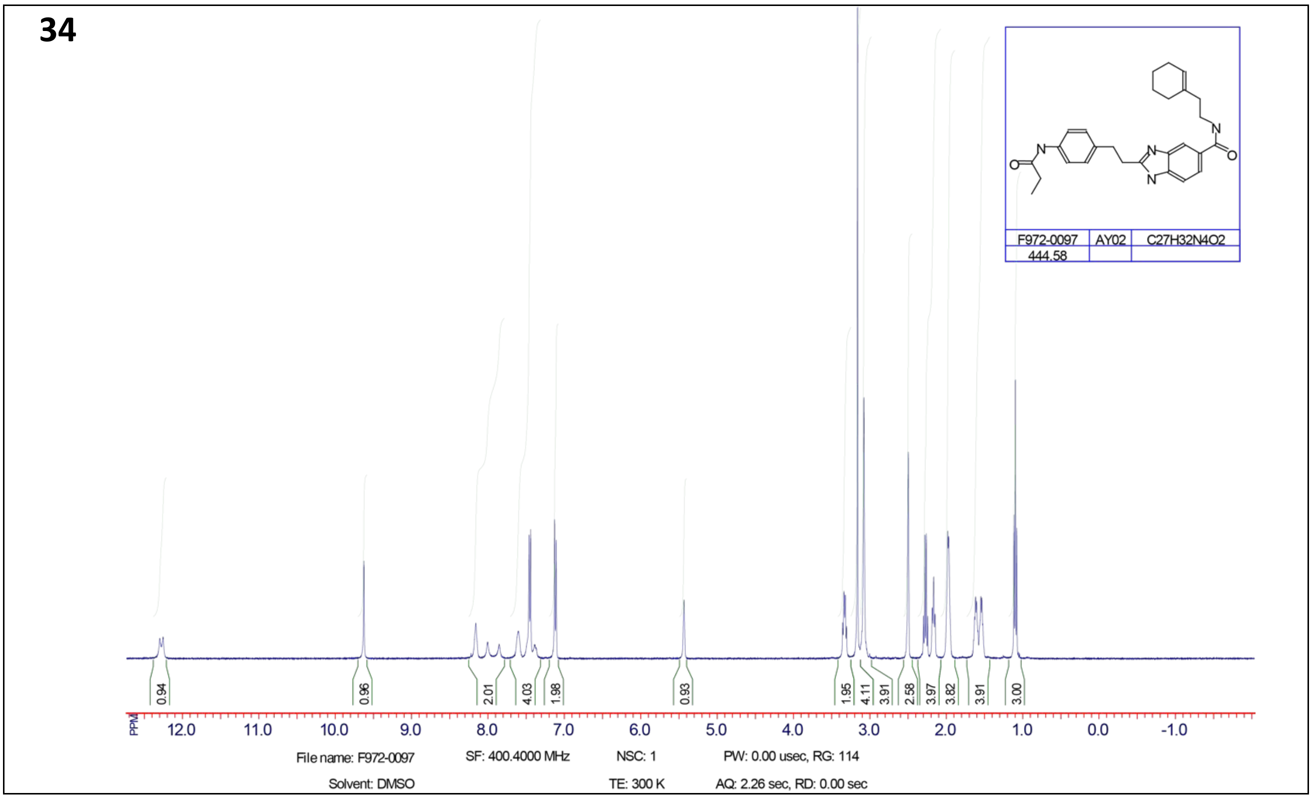
F972-0097
AY02
C27H32N4O2
444.58
0.94
0.96
2.01
4.03
1.98
0.93
1.95
4.11
3.91
2.58
3.97
3.82
3.91
3.00
PPM
12.0
11.0
10.0
9.0
8.0
7.0
6.0
5.0
4.0
3.0
2.0
1.0
0.0
-1.0
File name: F972-0097
SF: 400.4000 MHz
NSC: 1
PW: 0.00 usec, RG: 114
Solvent: DMSO
TE: 300 K
AQ: 2.26 sec, RD: 0.00 sec

35

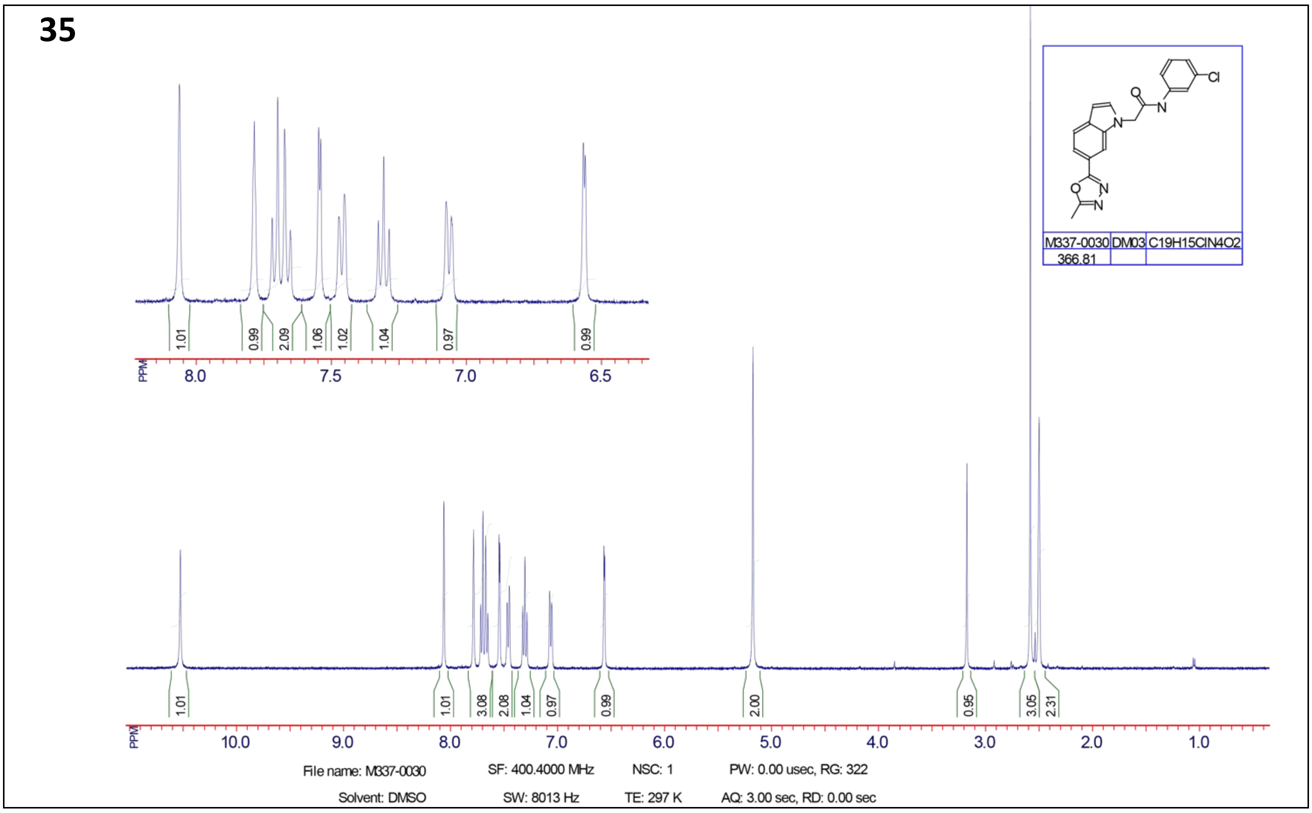
M337-0030
DM03
C19H15ClN4O2
366.81
1.01
0.99
2.09
1.06
1.02
1.04
0.97
0.99
PPM
8.0
7.5
7.0
6.5
1.01
1.01
3.08
2.08
1.04
0.97
0.99
2.00
0.95
3.05
2.31
PPM
10.0
9.0
8.0
7.0
6.0
5.0
4.0
3.0
2.0
1.0
File name: M337-0030
SF: 400.4000 MHz
NSC: 1
PW: 0.00 usec, RG: 322
Solvent: DMSO
SW: 8013 Hz
TE: 297 K
AQ: 3.00 sec, RD: 0.00 sec

36

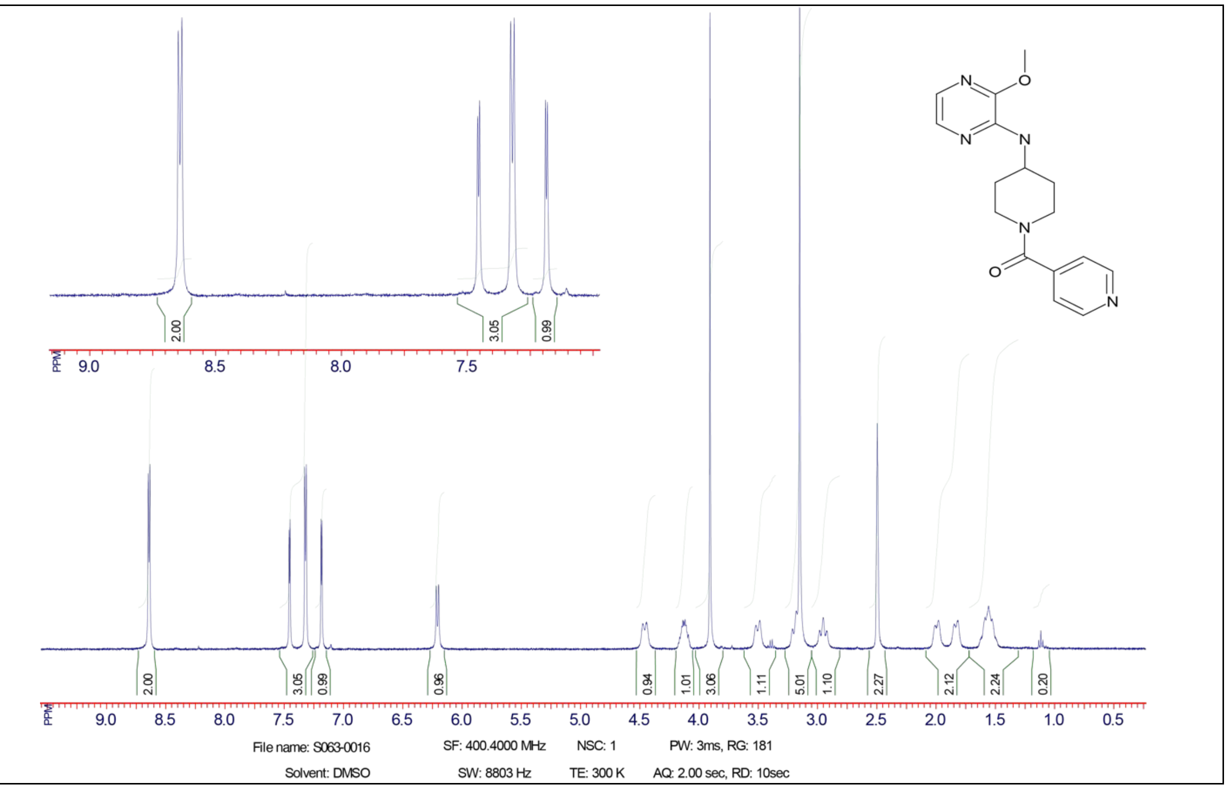
File name: S063-0016
SF: 400.4000 MHz
NSC: 1
PW: 3ms, RG: 181
Solvent: DMSO
SW: 8803 Hz
TE: 300 K
AQ: 2.00 sec, RD: 10sec

37

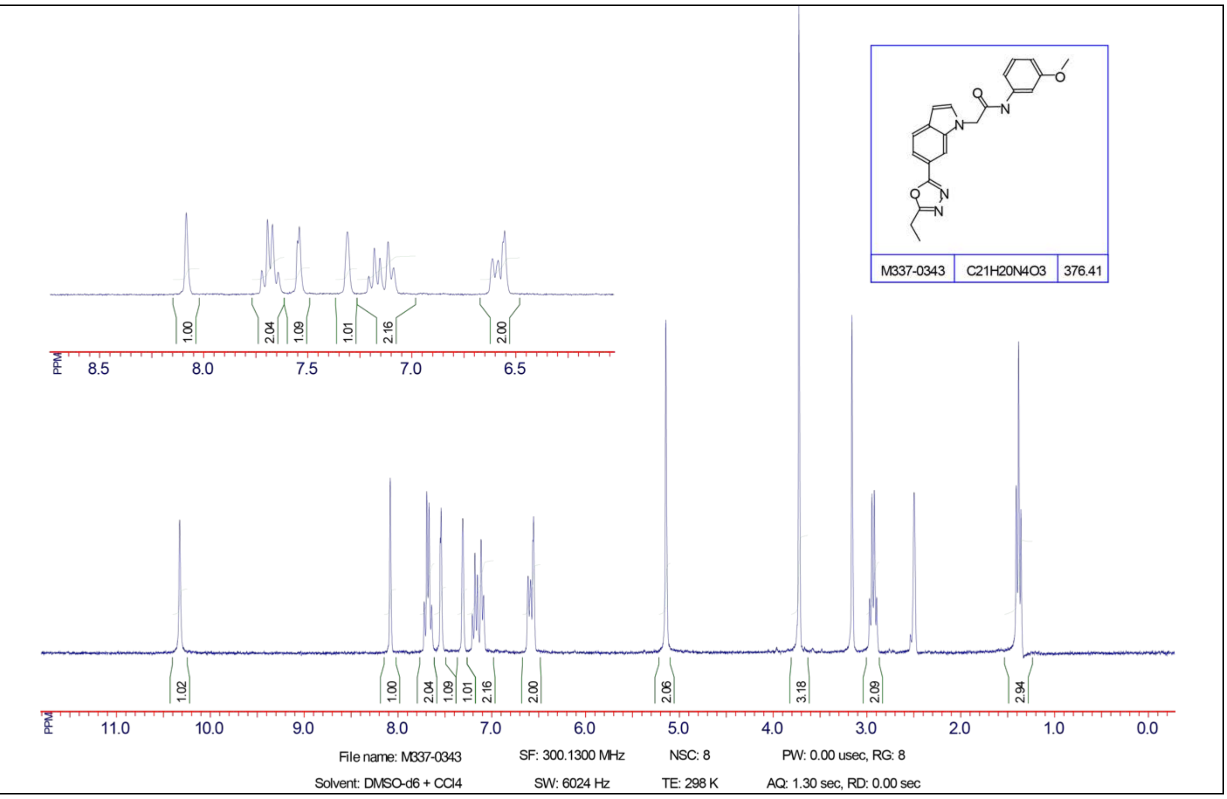
M337-0343
C21H20N4O3
376.41
File name: M337-0343
SF: 300.1300 MHz
NSC: 8
PW: 0.00 usec, RG: 8
Solvent: DMSO-d6 + CCl4
SW: 6024 Hz
TE: 298 K
AQ: 1.30 sec, RD: 0.00 sec

38

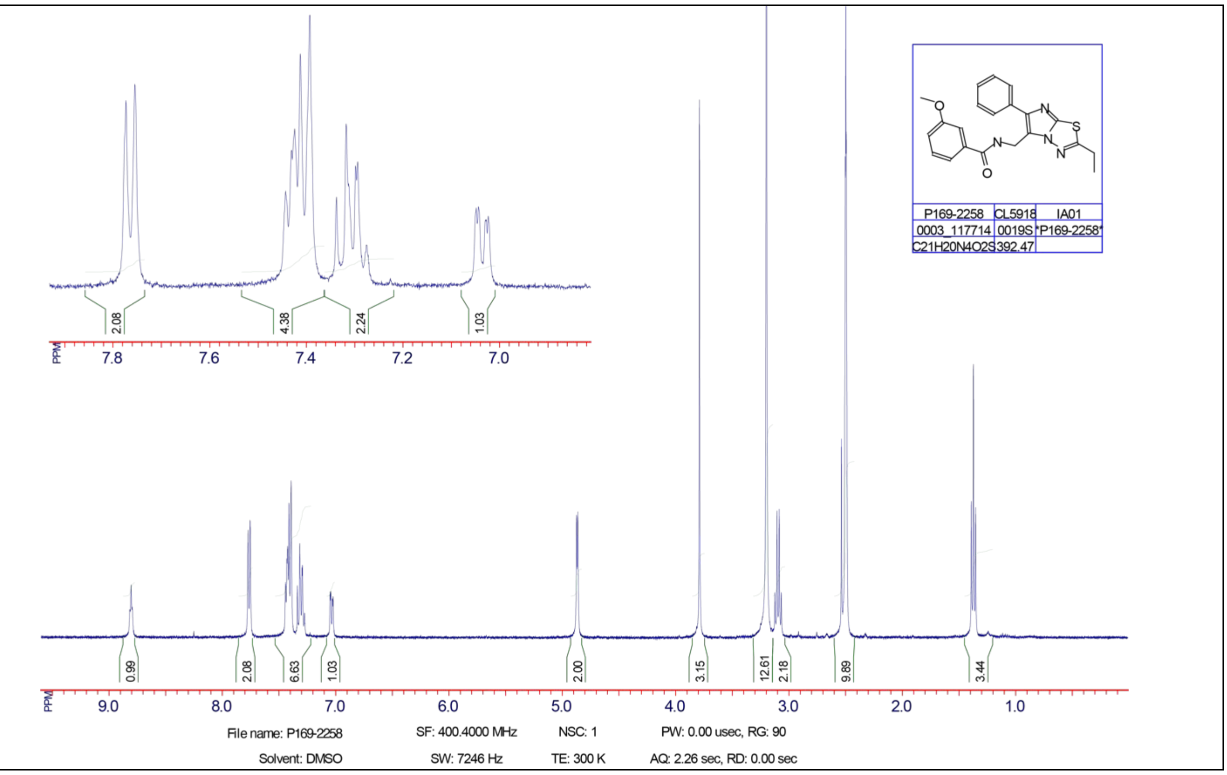
P169-2258 CL5918 IA01
0003_117714 0019S *P169-2258*
C21H20N4O2S 392.47
PPM
File name: P169-2258
SF: 400.4000 MHz
NSC: 1
PW: 0.00 usec, RG: 90
Solvent: DMSO
SW: 7246 Hz
TE: 300 K
AQ: 2.26 sec, RD: 0.00 sec

39

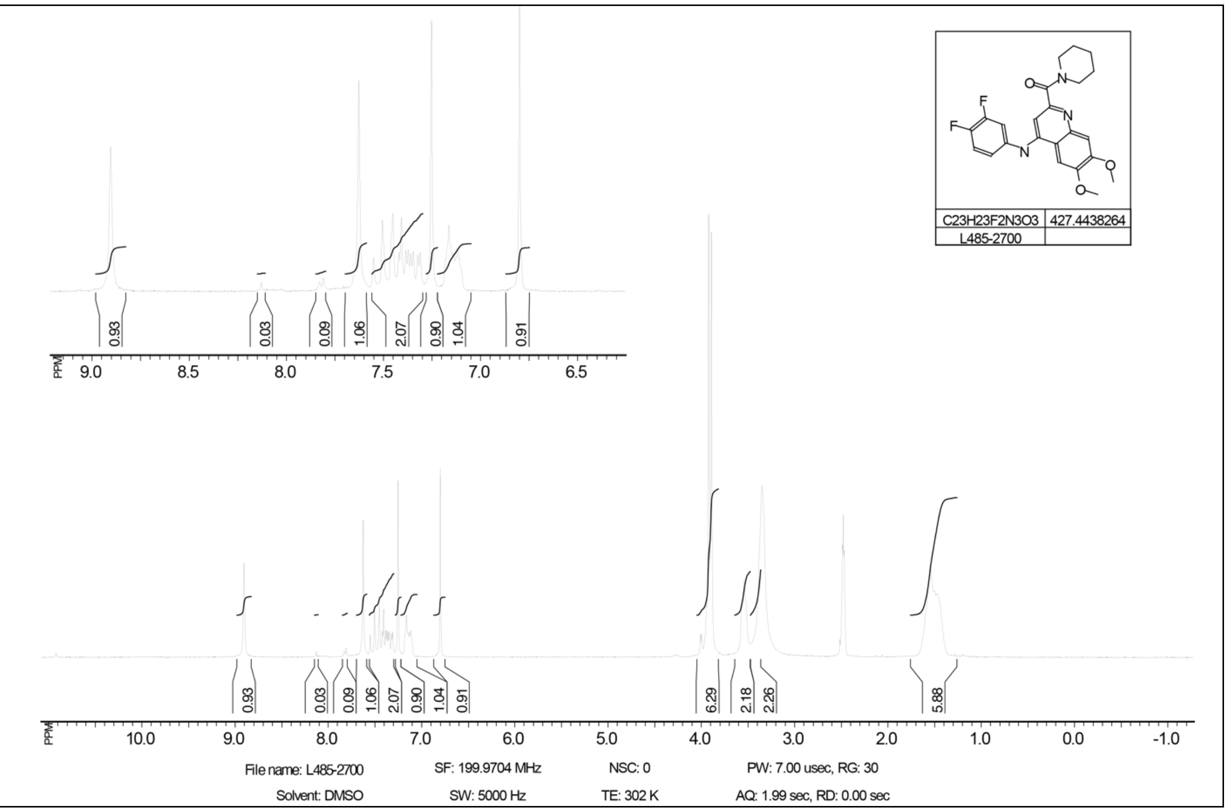
C23H23F2N3O3 427.4438264
L485-2700
PPM
File name: L485-2700
SF: 199.9704 MHz
NSC: 0
PW: 7.00 usec, RG: 30
Solvent: DMSO
SW: 5000 Hz
TE: 302 K
AQ: 1.99 sec, RD: 0.00 sec

40

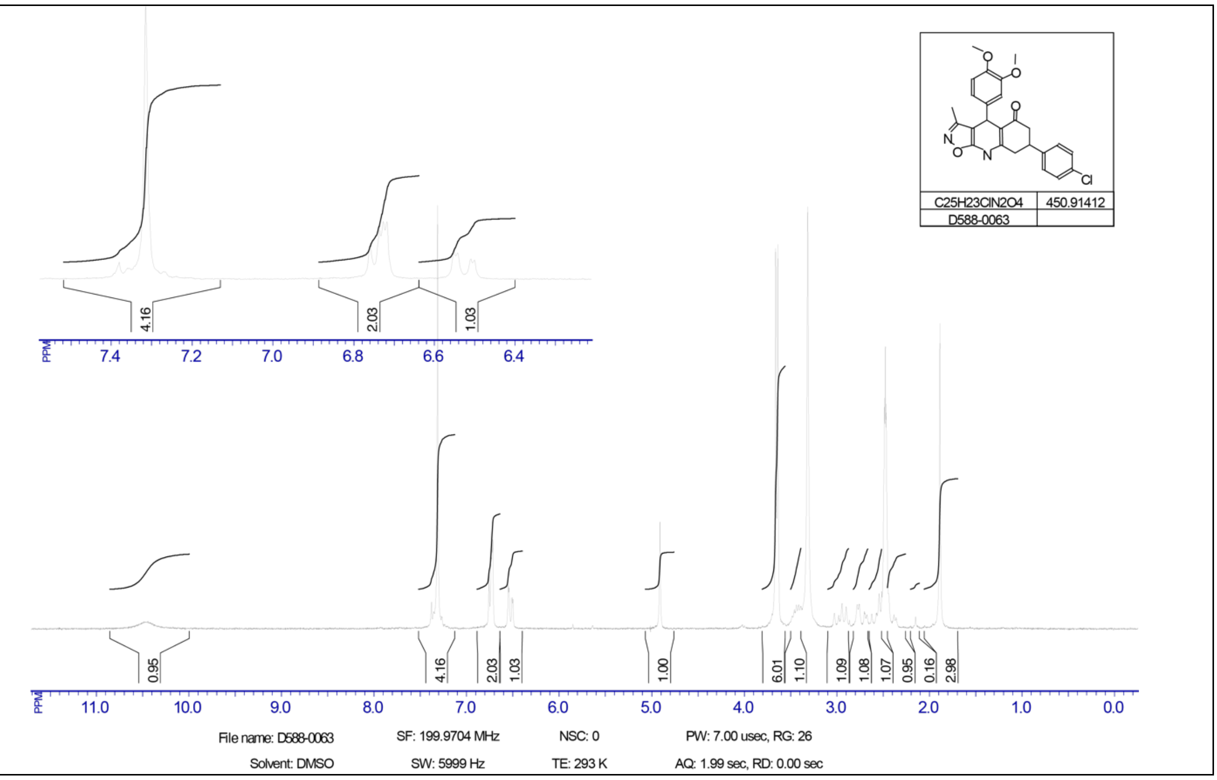
C25H23ClN2O4
450.91412
D588-0063
File name: D588-0063
Solvent: DMSO
SF: 199.9704 MHz
SW: 5999 Hz
NSC: 0
TE: 293 K
PW: 7.00 usec, RG: 26
AQ: 1.99 sec, RD: 0.00 sec

41

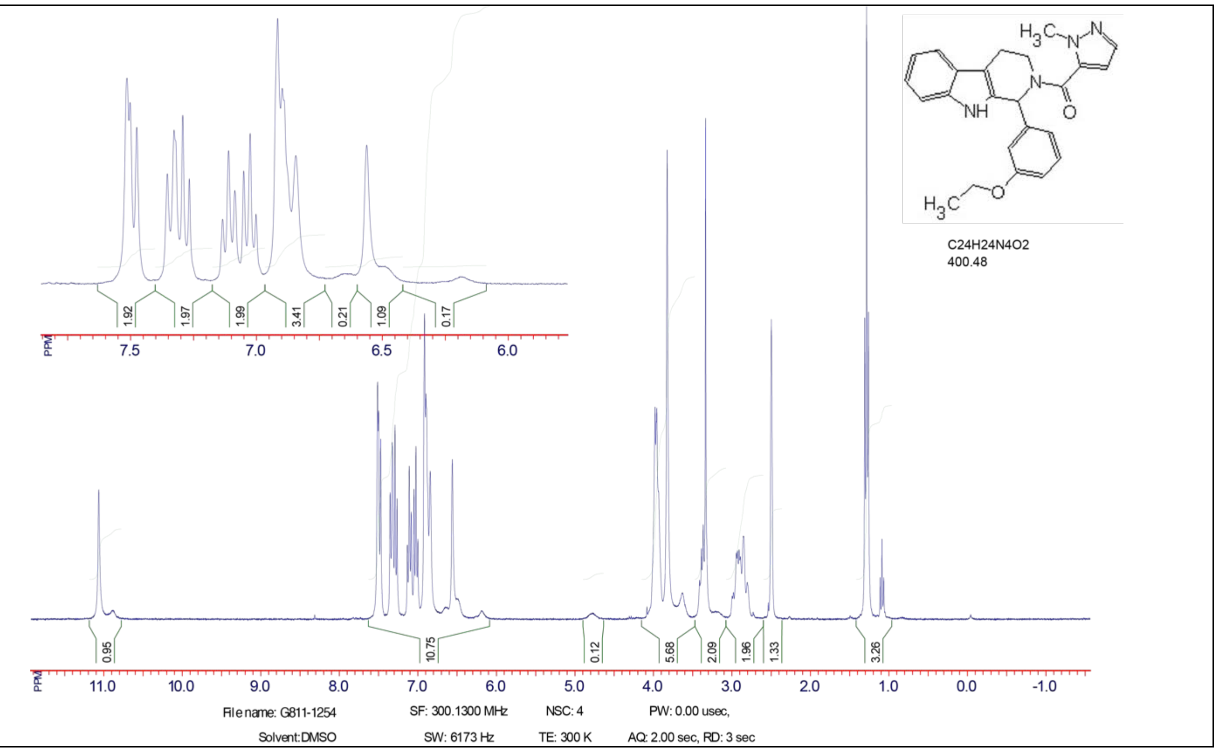
C24H24N4O2
400.48
File name: G811-1254
Solvent:DMSO
SF: 300.1300 MHz
SW: 6173 Hz
NSC: 4
TE: 300 K
PW: 0.00 usec,
AQ: 2.00 sec, RD: 3 sec

42

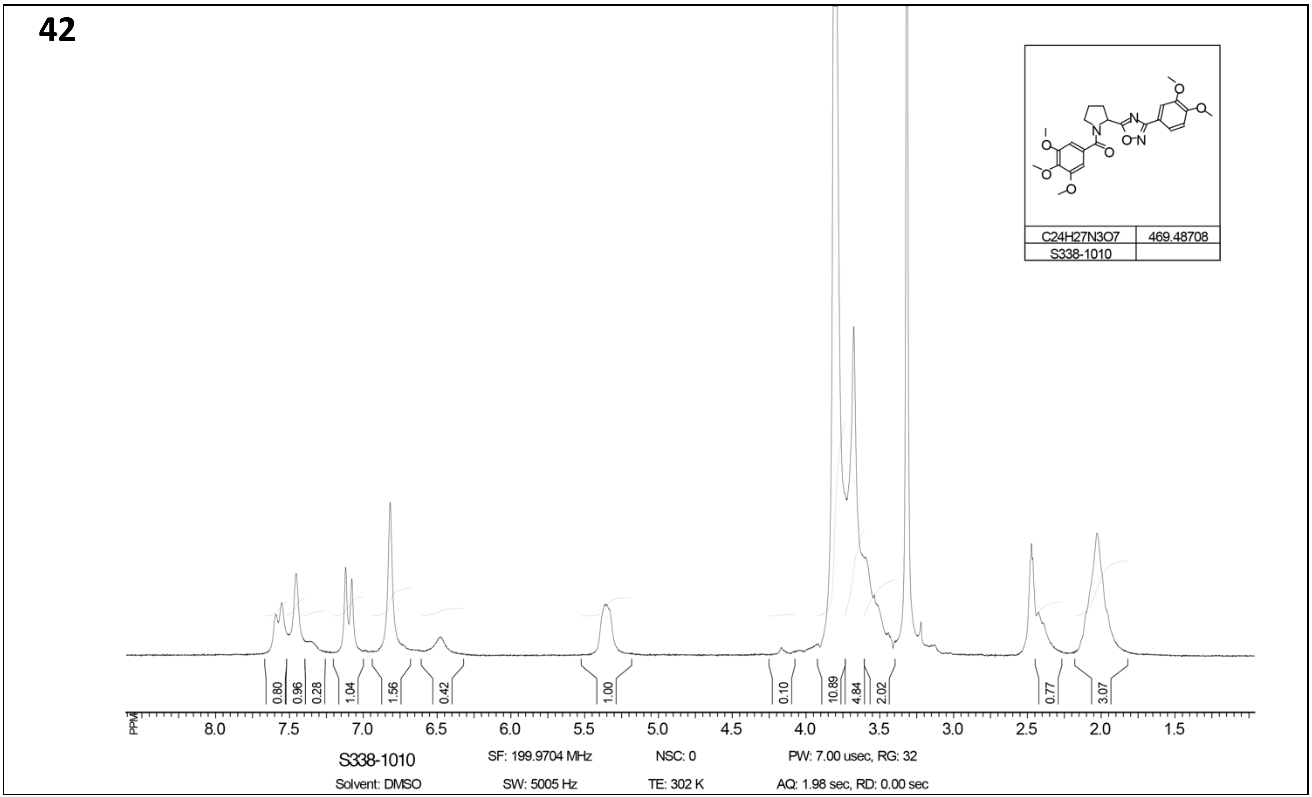
C24H27N3O7
469.48708
S338-1010
0.80
0.96
0.28
1.04
1.56
0.42
1.00
0.10
10.89
4.84
2.02
0.77
3.07
PPM
8.0 7.5 7.0 6.5 6.0 5.5 5.0 4.5 4.0 3.5 3.0 2.5 2.0 1.5
S338-1010
SF: 199.9704 MHz
NSC: 0
PW: 7.00 usec, RG: 32
Solvent: DMSO
SW: 5005 Hz
TE: 302 K
AQ: 1.98 sec, RD: 0.00 sec

43

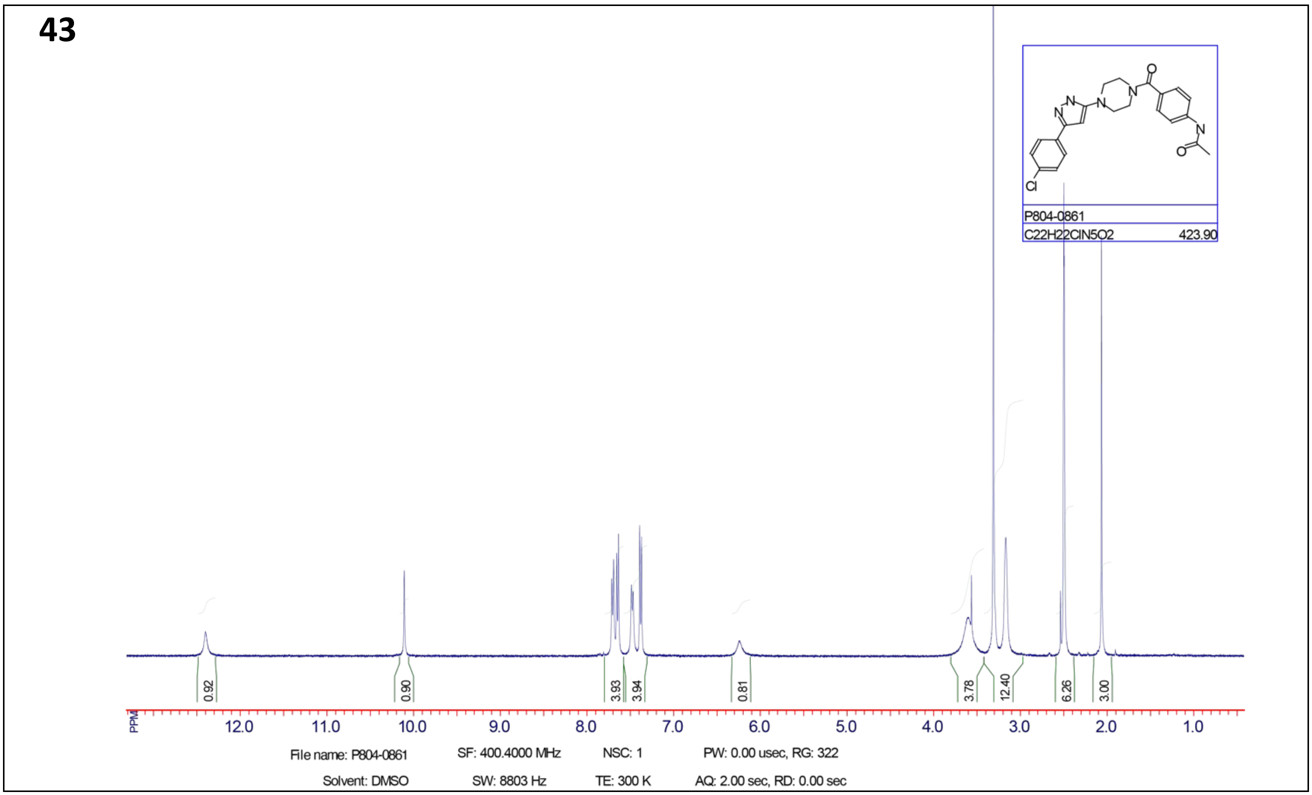
P804-0861
C22H22ClN5O2
423.90
0.92
0.90
3.93
3.94
0.81
3.78
12.40
6.26
3.00
PPM
12.0 11.0 10.0 9.0 8.0 7.0 6.0 5.0 4.0 3.0 2.0 1.0
File name: P804-0861
SF: 400.4000 MHz
NSC: 1
PW: 0.00 usec, RG: 322
Solvent: DMSO
SW: 8803 Hz
TE: 300 K
AQ: 2.00 sec, RD: 0.00 sec

44

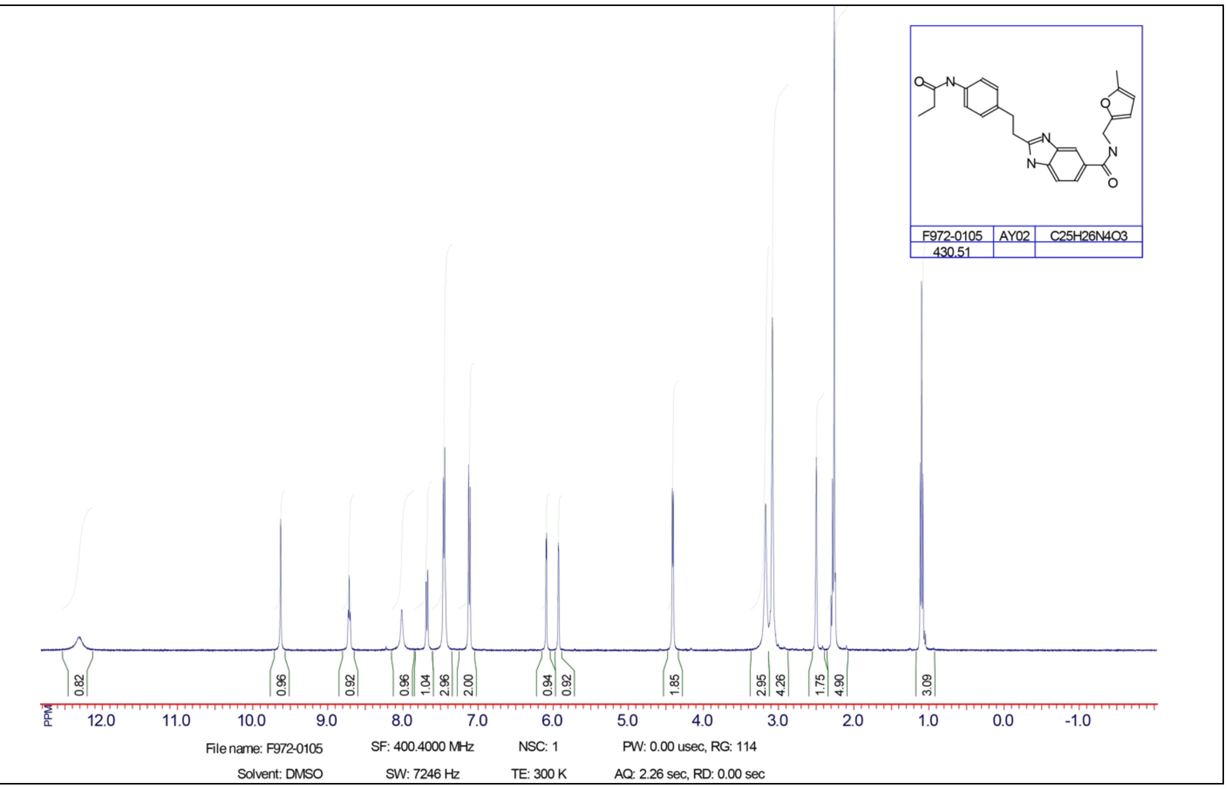
F972-0105 AY02 C25H26N4O3
430.51
0.82 0.96 0.92 0.96 1.04 2.96 2.00 0.94 0.92 1.85 2.95 4.26 1.75 4.90 3.09
PPM 12.0 11.0 10.0 9.0 8.0 7.0 6.0 5.0 4.0 3.0 2.0 1.0 0.0 -1.0
File name: F972-0105 SF: 400.4000 MHz NSC: 1 PW: 0.00 usec, RG: 114
Solvent: DMSO SW: 7246 Hz TE: 300 K AQ: 2.26 sec, RD: 0.00 sec

45

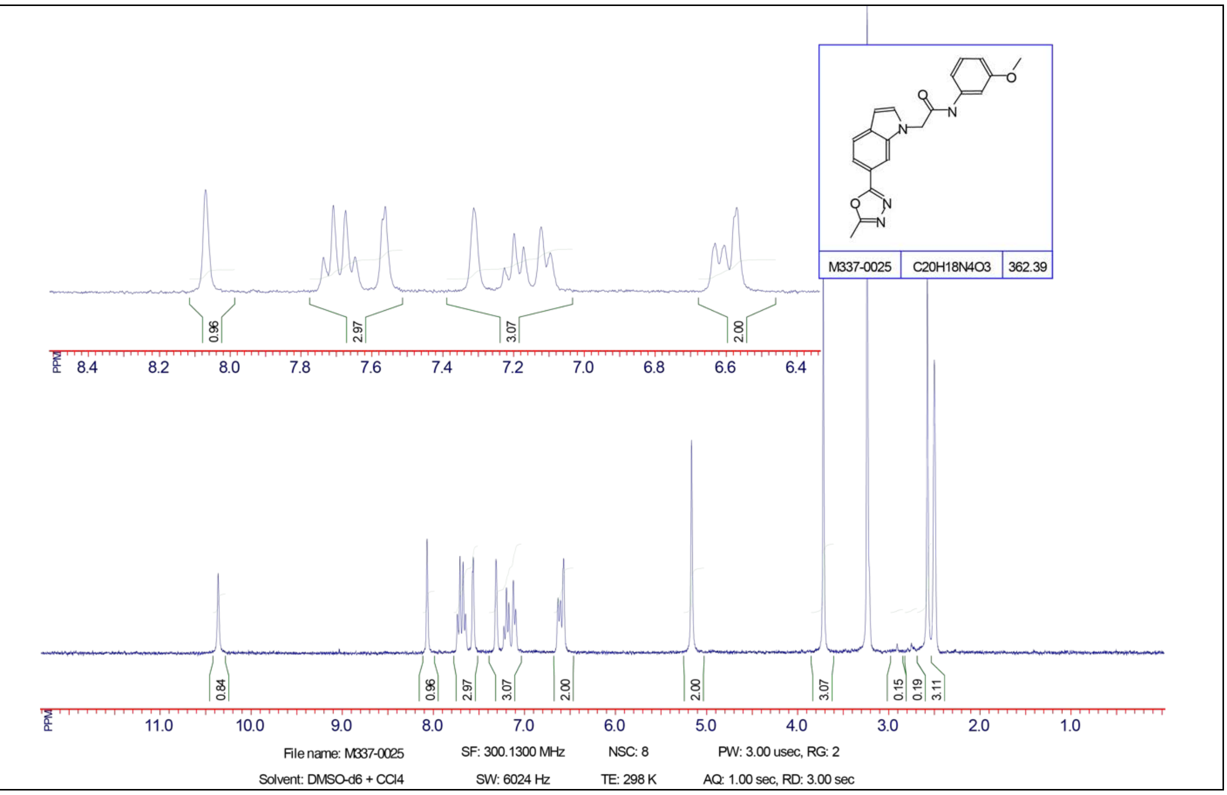
M337-0025 C20H18N4O3 362.39
0.96 2.97 3.07 2.00
PPM 8.4 8.2 8.0 7.8 7.6 7.4 7.2 7.0 6.8 6.6 6.4
0.84 0.96 2.97 3.07 2.00 2.00 3.07 0.15 0.19 3.11
PPM 11.0 10.0 9.0 8.0 7.0 6.0 5.0 4.0 3.0 2.0 1.0
File name: M337-0025 SF: 300.1300 MHz NSC: 8 PW: 3.00 usec, RG: 2
Solvent: DMSO-d6 + CCl4 SW: 6024 Hz TE: 298 K AQ: 1.00 sec, RD: 3.00 sec

46

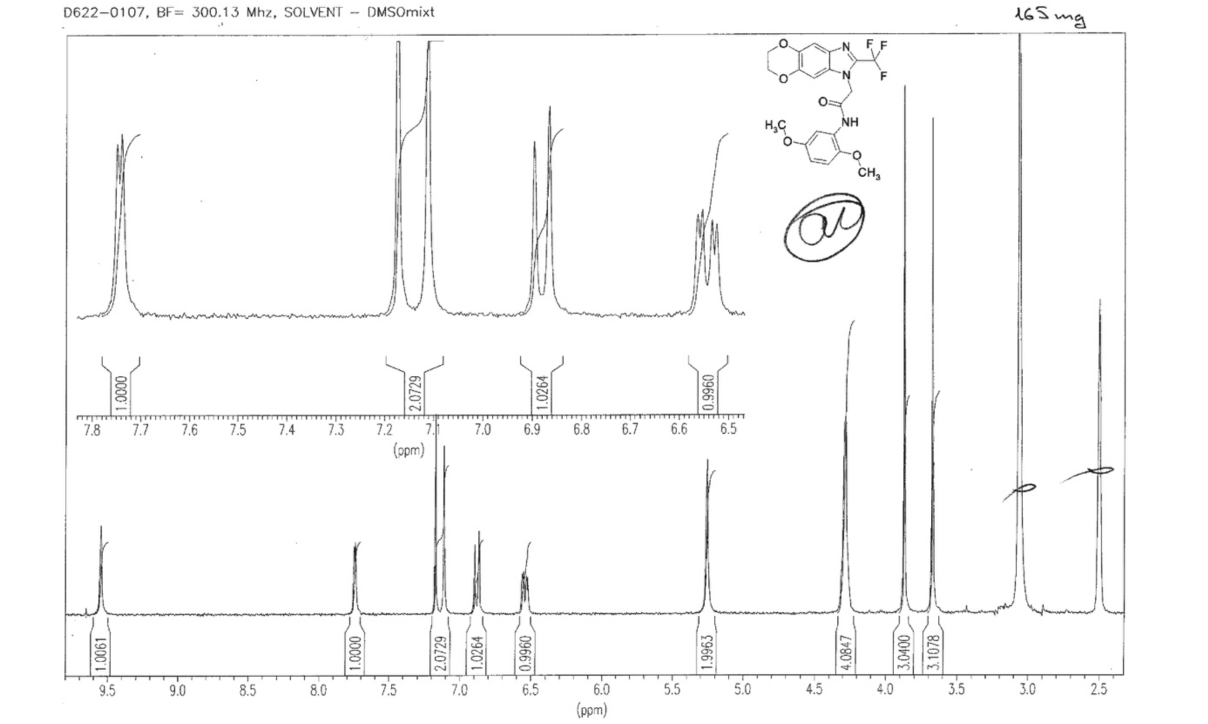

D622-0107, BF= 300.13 Mhz, SOLVENT – DMSOmixt
165 mg
(ppm)
(ppm)

48

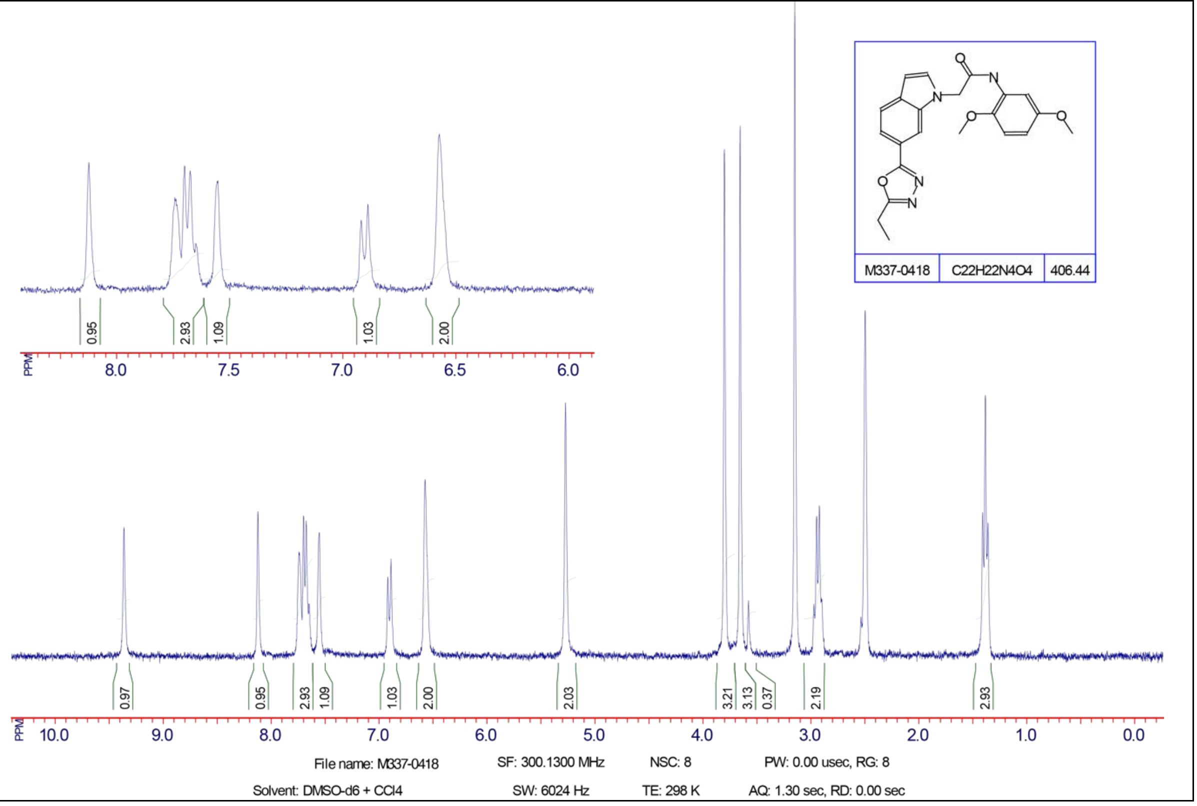

M337-0418
C22H22N4O4
406.44
PPM
File name: M337-0418
SF: 300.1300 MHz
NSC: 8
PW: 0.00 usec, RG: 8
Solvent: DMSO-d6 + CCl4
SW: 6024 Hz
TE: 298 K
AQ: 1.30 sec, RD: 0.00 sec

49

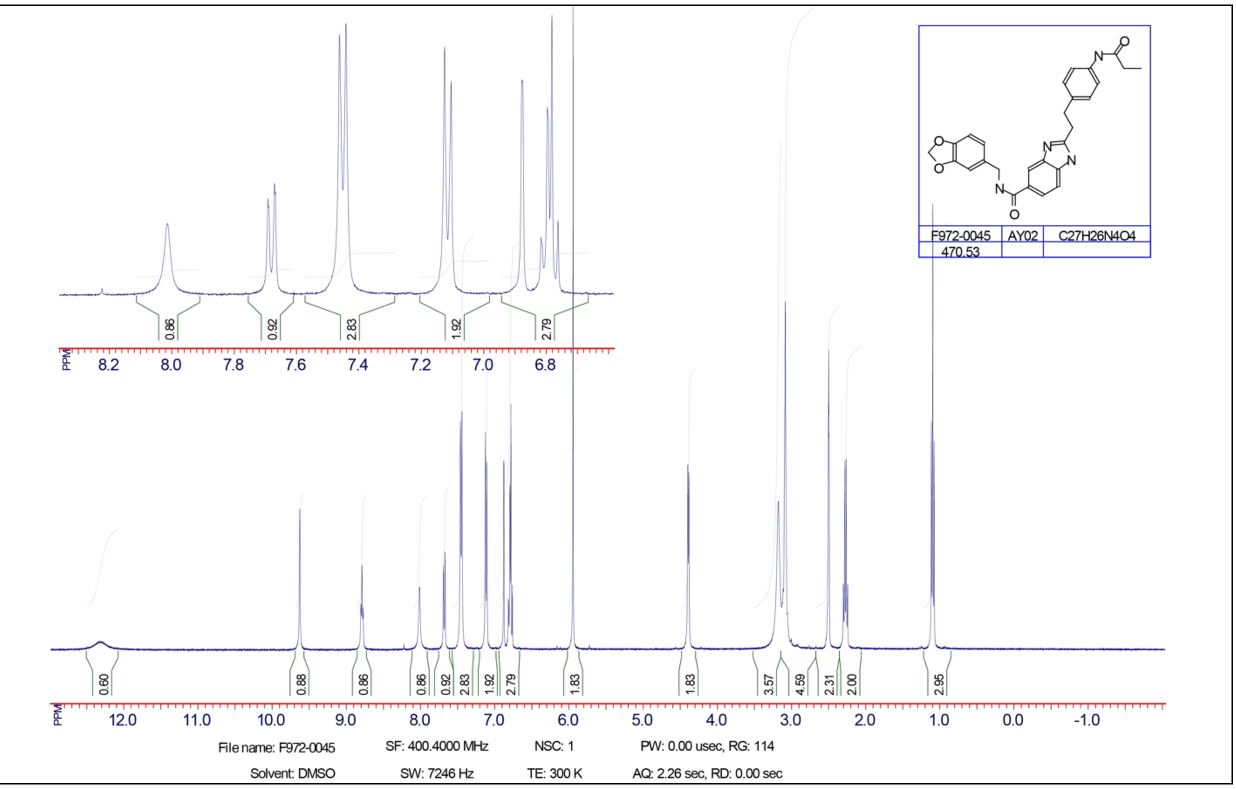
F972-0045
AY02
C27H26N4O4
470.53
File name: F972-0045
SF: 400.4000 MHz
NSC: 1
PW: 0.00 usec, RG: 114
Solvent: DMSO
SW: 7246 Hz
TE: 300 K
AQ: 2.26 sec, RD: 0.00 sec

50

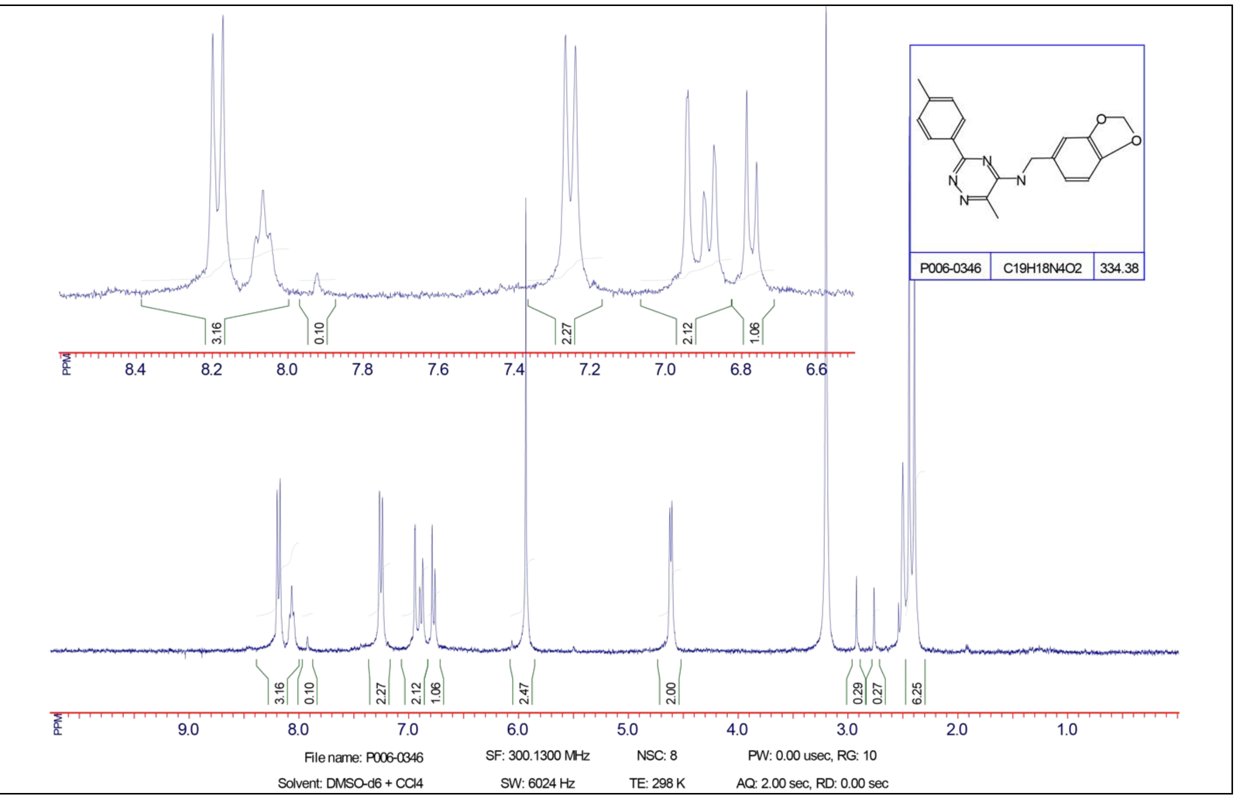
P006-0346
C19H18N4O2
334.38
File name: P006-0346
SF: 300.1300 MHz
NSC: 8
PW: 0.00 usec, RG: 10
Solvent: DMSO-d6 + CCl4
SW: 6024 Hz
TE: 298 K
AQ: 2.00 sec, RD: 0.00 sec

51

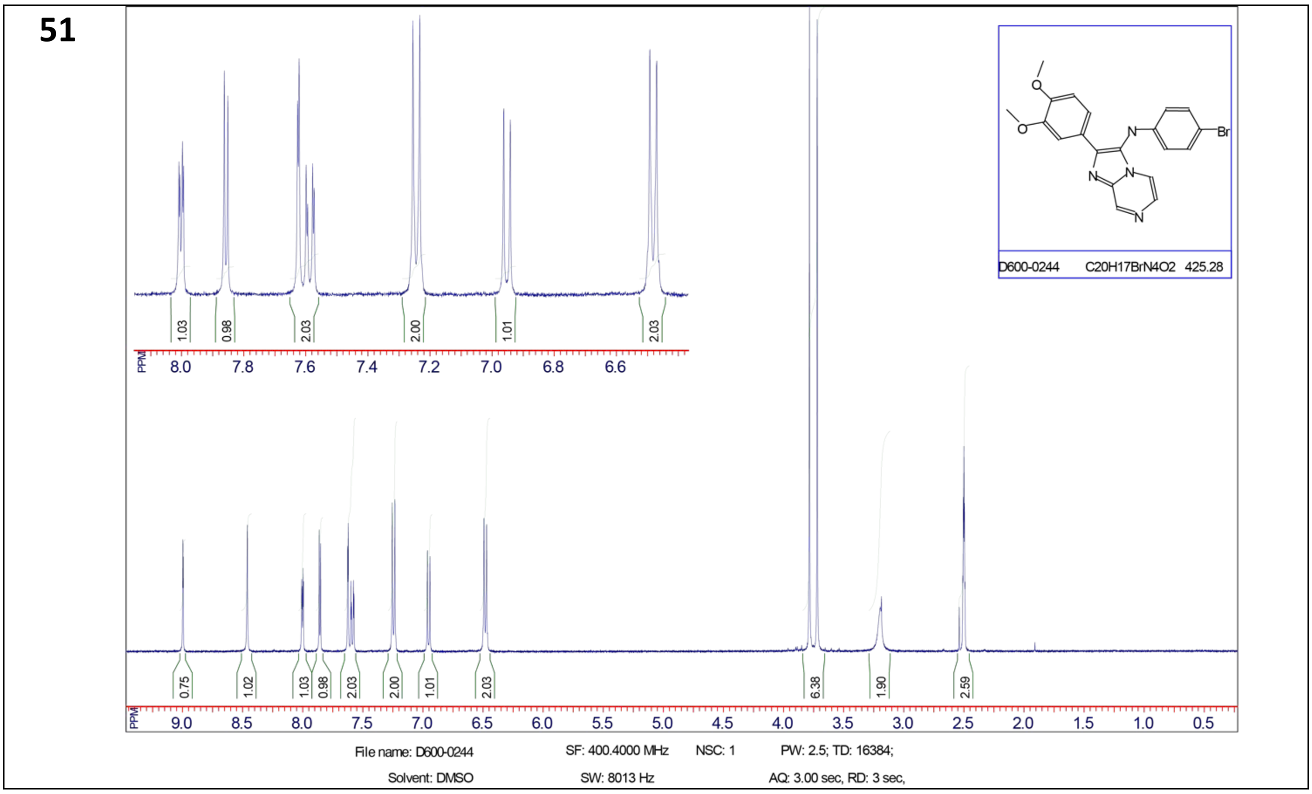
D600-0244 C20H17BrN4O2 425.28
File name: D600-0244
SF: 400.4000 MHz
NSC: 1
PW: 2.5; TD: 16384;
Solvent: DMSO
SW: 8013 Hz
AQ: 3.00 sec, RD: 3 sec,

52

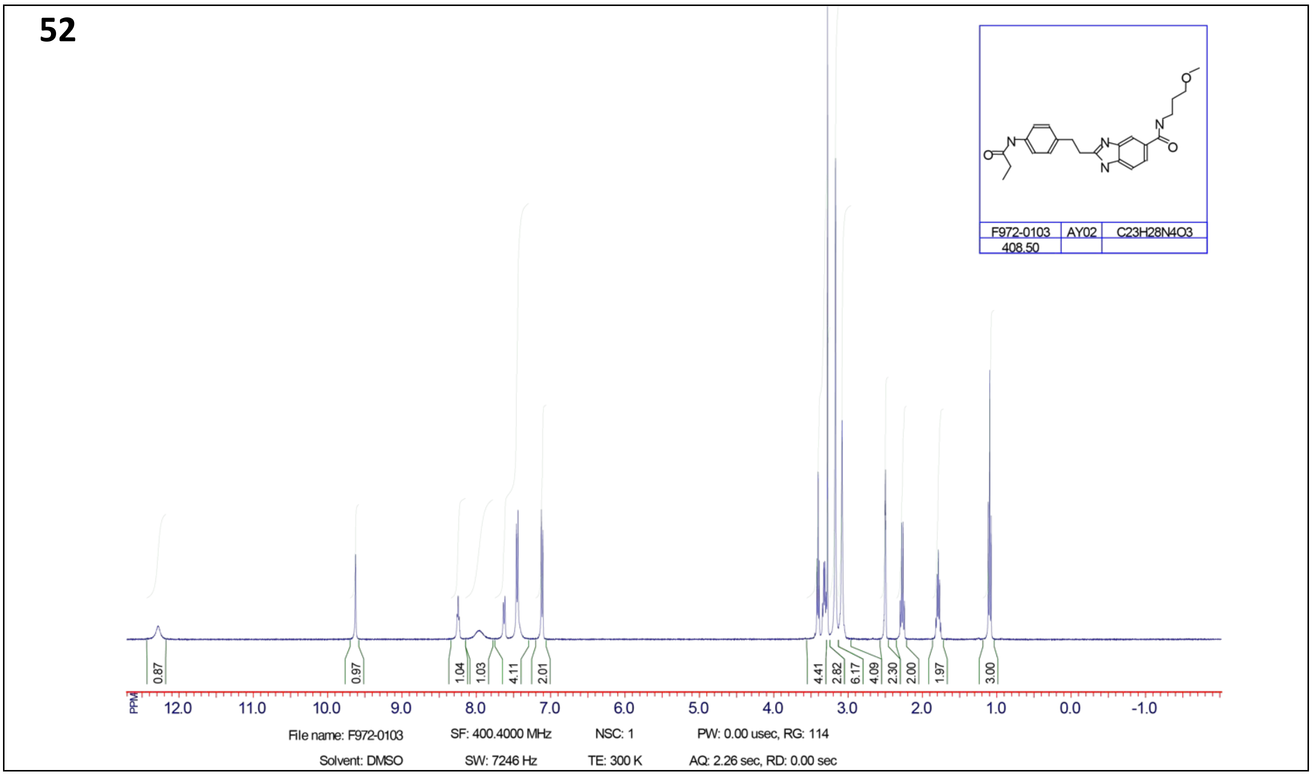
F972-0103 AY02 C23H28N4O3
408.50
File name: F972-0103
SF: 400.4000 MHz
NSC: 1
PW: 0.00 usec, RG: 114
Solvent: DMSO
SW: 7246 Hz
TE: 300 K
AQ: 2.26 sec, RD: 0.00 sec

53

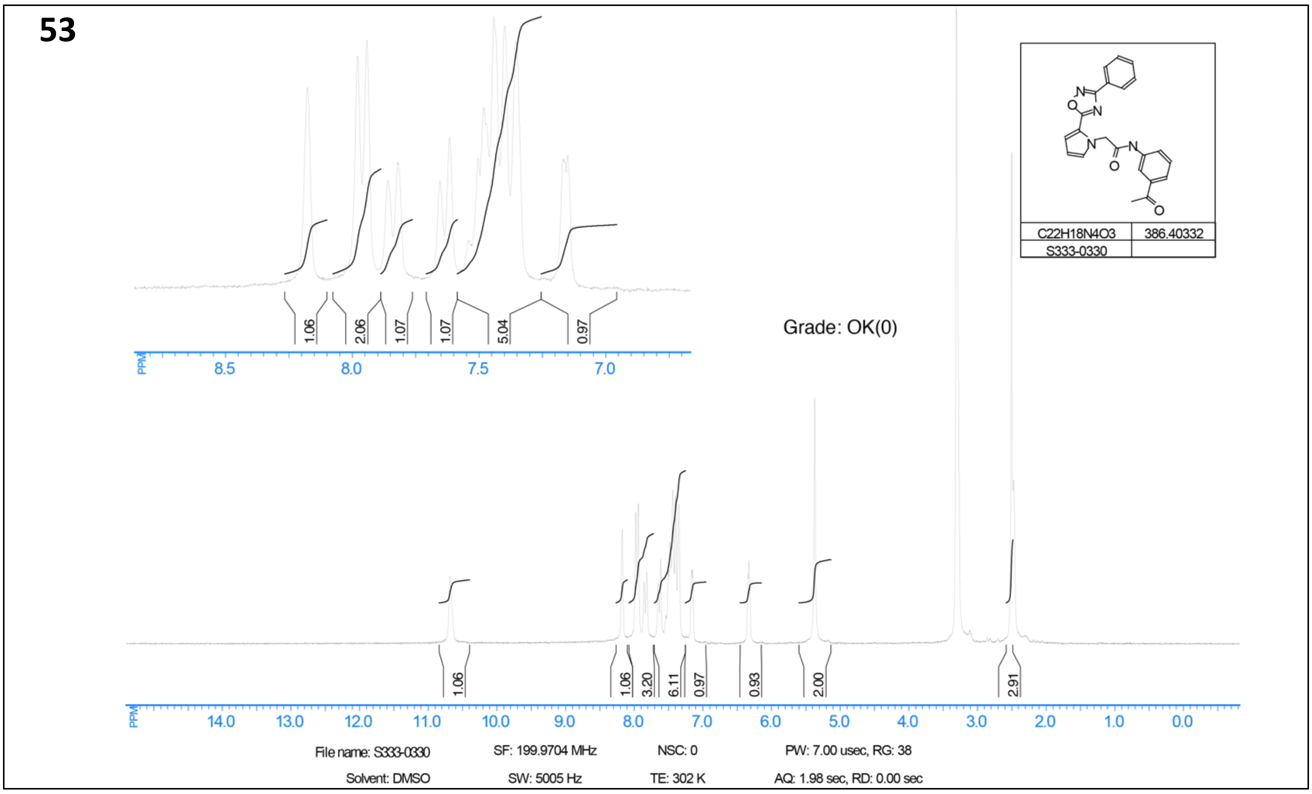
Grade: OK(0)
C22H18N4O3
386.40332
S333-0330
File name: S333-0330
SF: 199.9704 MHz
NSC: 0
PW: 7.00 usec, RG: 38
Solvent: DMSO
SW: 5005 Hz
TE: 302 K
AQ: 1.98 sec, RD: 0.00 sec

54

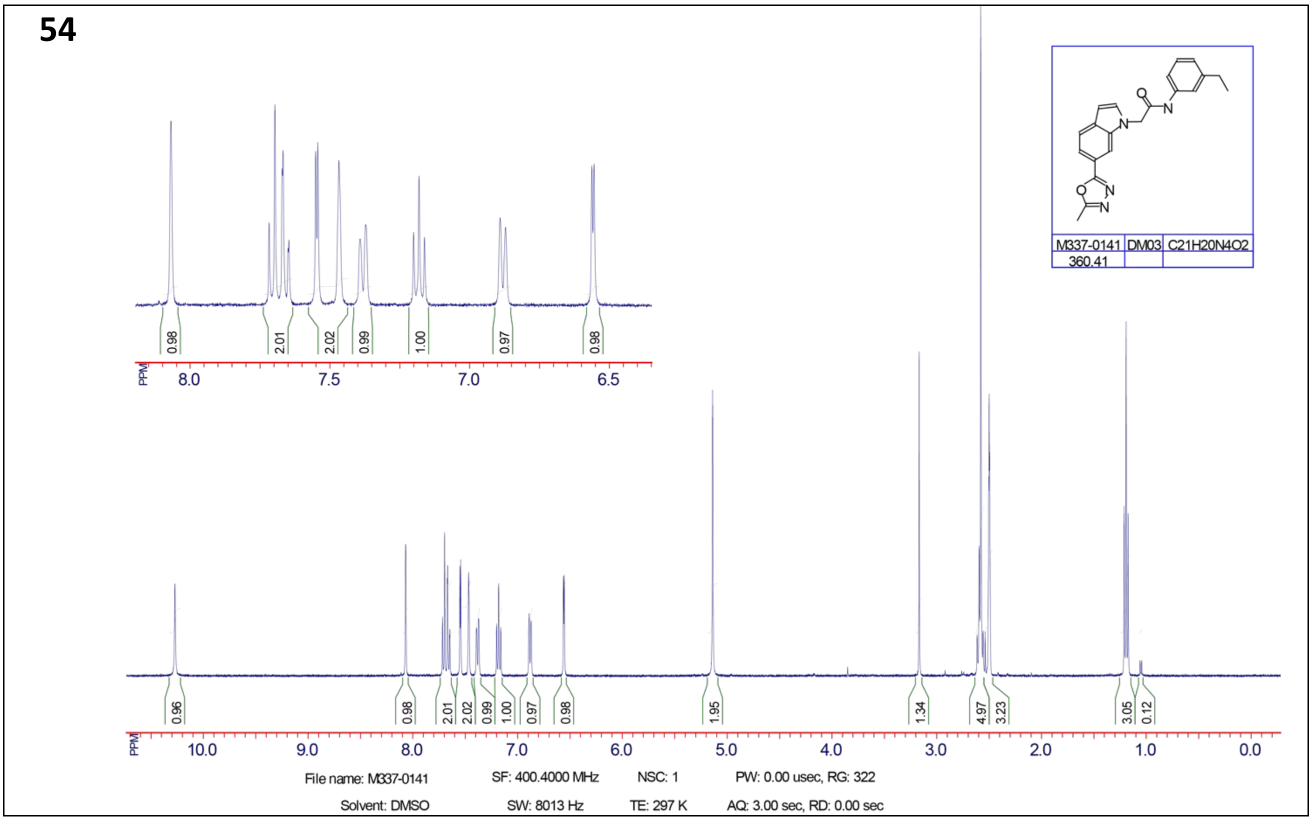
M337-0141
DM03
C21H20N4O2
360.41
File name: M337-0141
SF: 400.4000 MHz
NSC: 1
PW: 0.00 usec, RG: 322
Solvent: DMSO
SW: 8013 Hz
TE: 297 K
AQ: 3.00 sec, RD: 0.00 sec

55

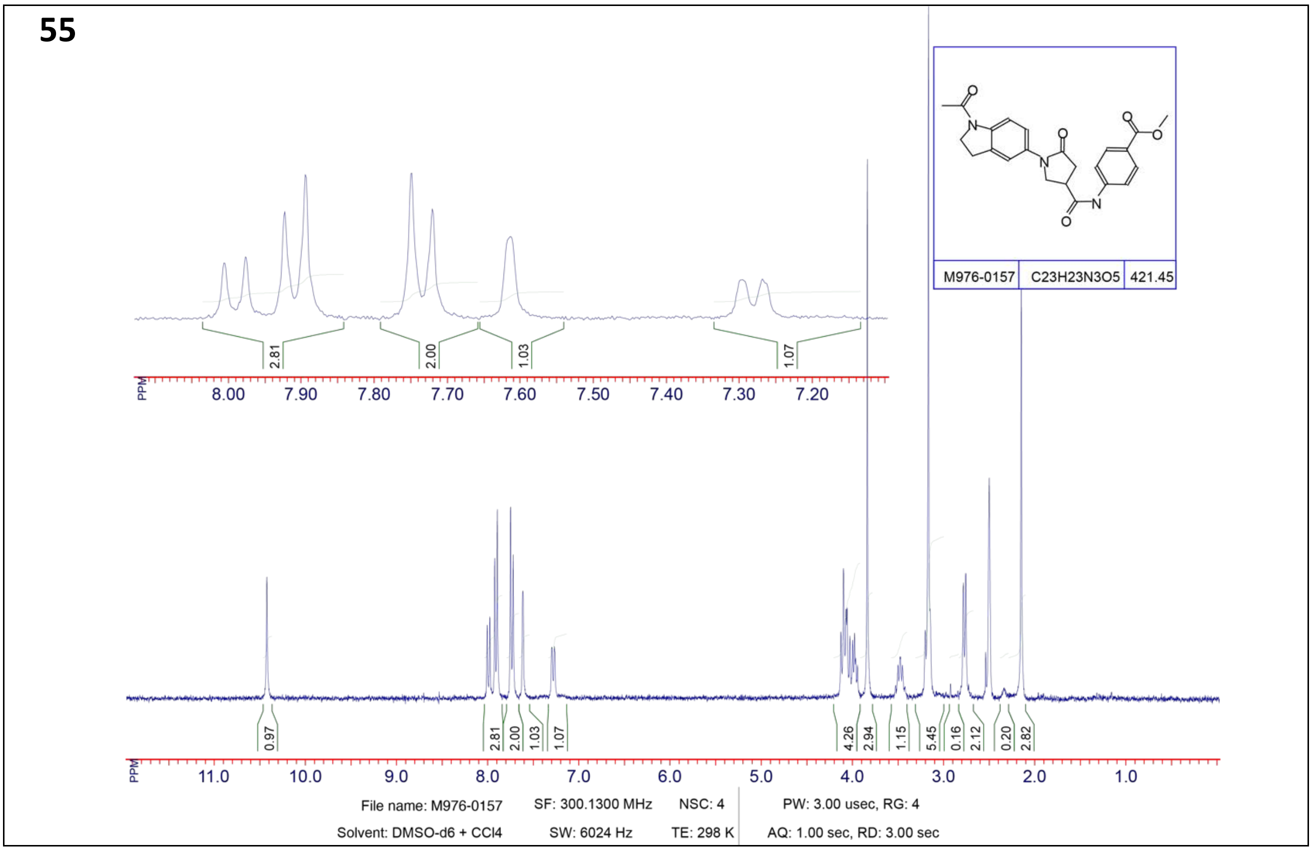
M976-0157
C23H23N3O5
421.45
2.81
2.00
1.03
1.07
PPM
8.00
7.90
7.80
7.70
7.60
7.50
7.40
7.30
7.20
0.97
2.81
2.00
1.03
1.07
4.26
2.94
1.15
5.45
0.16
2.12
0.20
2.82
11.0
10.0
9.0
8.0
7.0
6.0
5.0
4.0
3.0
2.0
1.0
File name: M976-0157
SF: 300.1300 MHz
NSC: 4
PW: 3.00 usec, RG: 4
Solvent: DMSO-d6 + CCl4
SW: 6024 Hz
TE: 298 K
AQ: 1.00 sec, RD: 3.00 sec

57

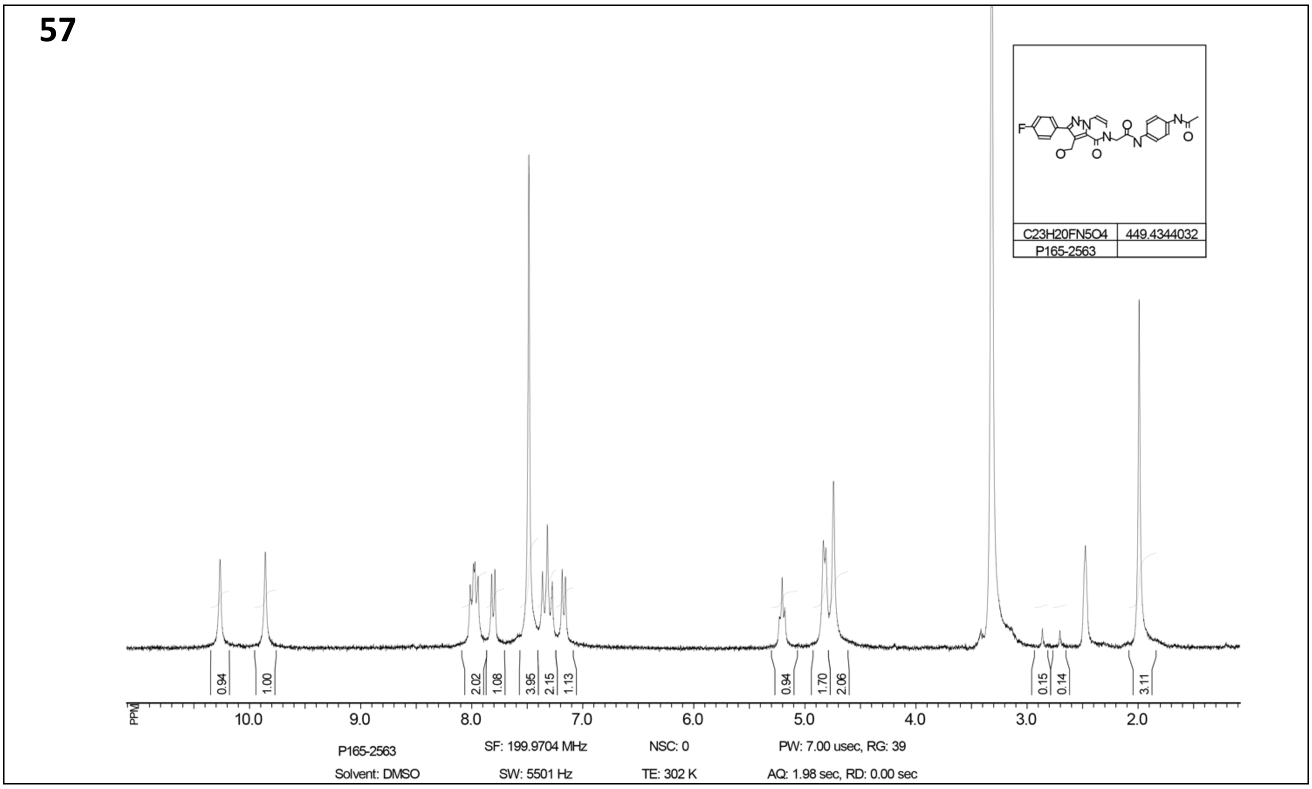
C23H20FN5O4
449.4344032
P165-2563
0.94
1.00
2.02
1.08
3.95
2.15
1.13
0.94
1.70
2.06
0.15
0.14
3.11
PPM
10.0
9.0
8.0
7.0
6.0
5.0
4.0
3.0
2.0
P165-2563
SF: 199.9704 MHz
NSC: 0
PW: 7.00 usec, RG: 39
Solvent: DMSO
SW: 5501 Hz
TE: 302 K
AQ: 1.98 sec, RD: 0.00 sec

58

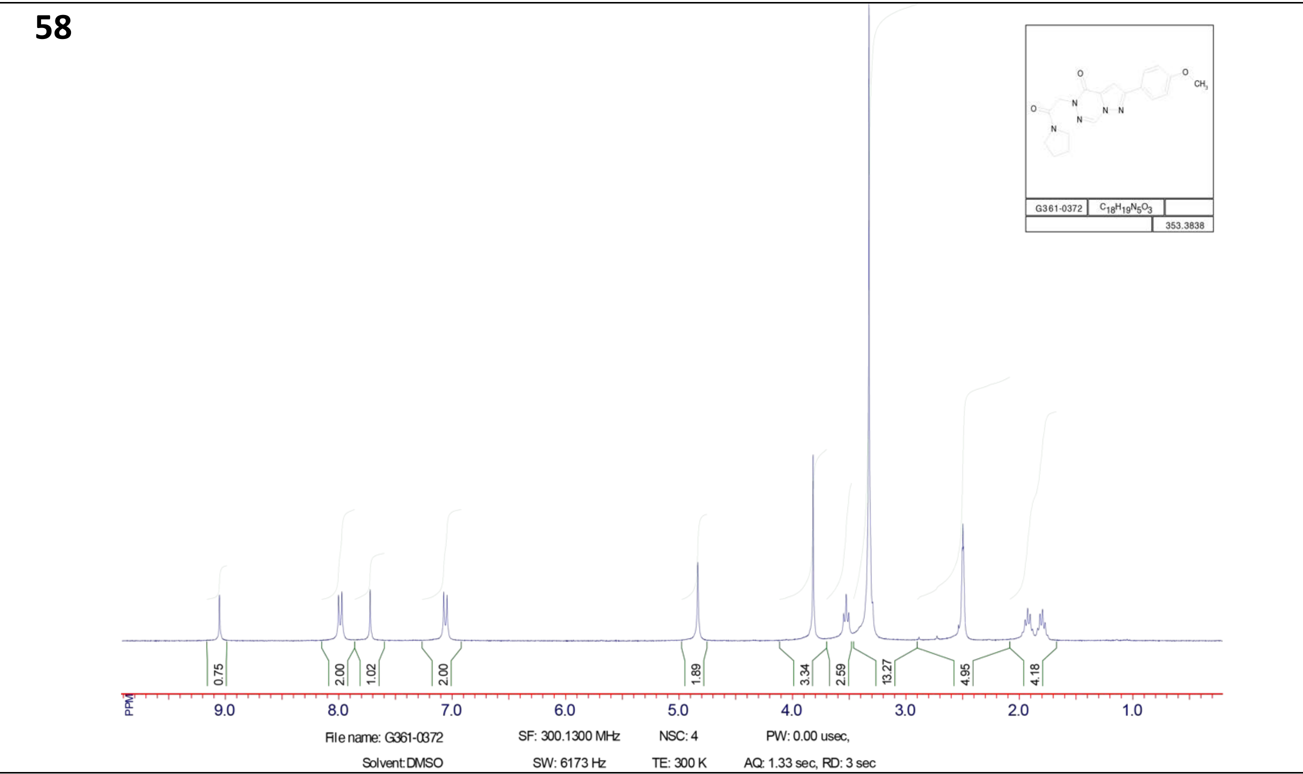
G361-0372
$C_{18}H_{19}N_5O_3$
353.3838
0.75
2.00
1.02
2.00
1.89
3.34
2.59
13.27
4.95
4.18
PPM
9.0
8.0
7.0
6.0
5.0
4.0
3.0
2.0
1.0
File name: G361-0372
SF: 300.1300 MHz
NSC: 4
PW: 0.00 usec,
Solvent:DMSO
SW: 6173 Hz
TE: 300 K
AQ: 1.33 sec, RD: 3 sec

59

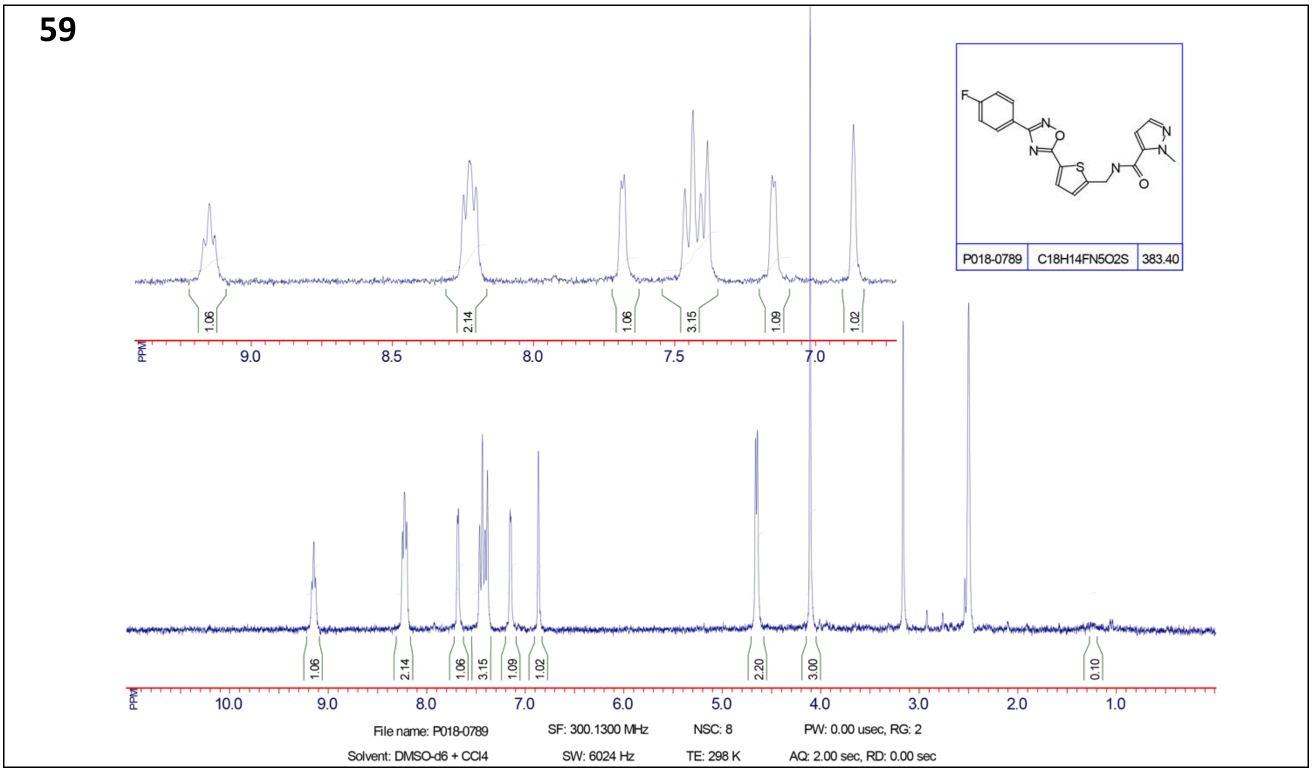
P018-0789
C18H14FN5O2S
383.40
1.06
2.14
1.06
3.15
1.09
1.02
2.20
3.00
0.10
PPM
9.0
8.5
8.0
7.5
7.0
10.0
6.0
5.0
4.0
3.0
2.0
1.0
File name: P018-0789
SF: 300.1300 MHz
NSC: 8
PW: 0.00 usec, RG: 2
Solvent: DMSO-d6 + CCl4
SW: 6024 Hz
TE: 298 K
AQ: 2.00 sec, RD: 0.00 sec

60

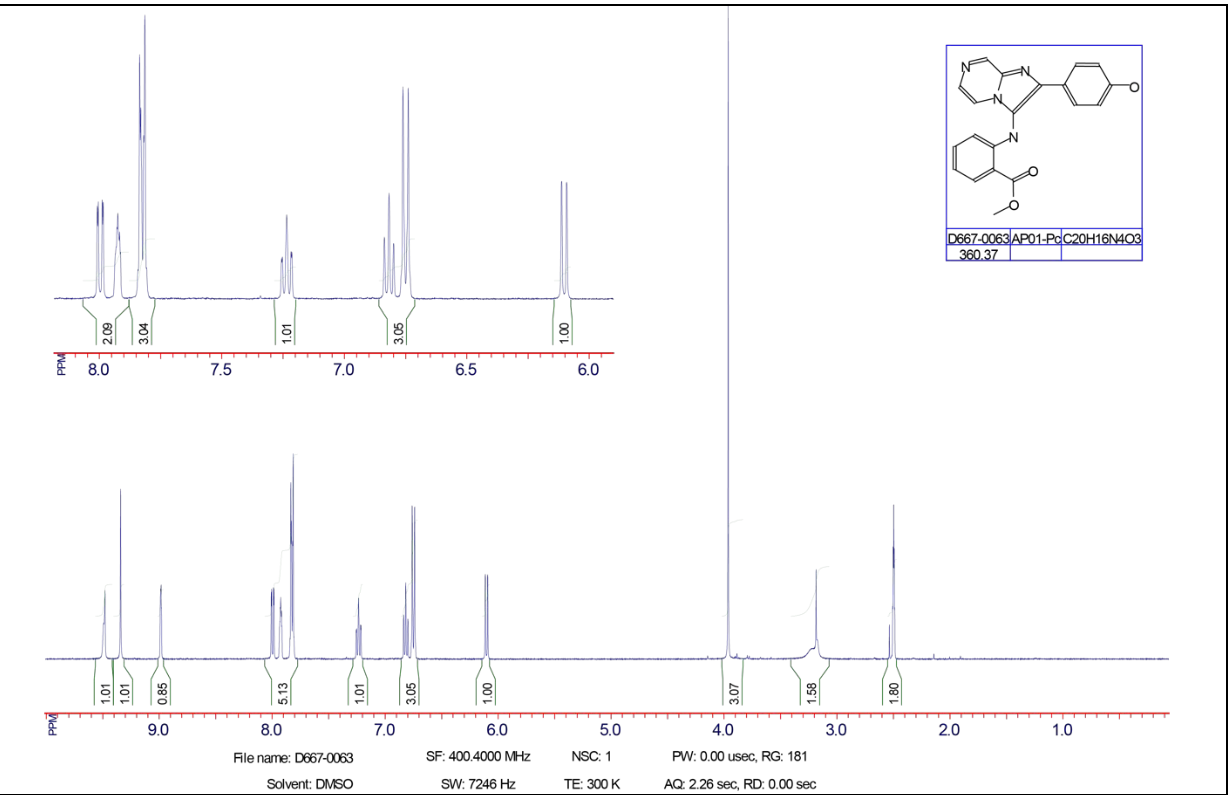
D667-0063
AP01-Pc
C20H16N4O3
360.37
File name: D667-0063
SF: 400.4000 MHz
NSC: 1
PW: 0.00 usec, RG: 181
Solvent: DMSO
SW: 7246 Hz
TE: 300 K
AQ: 2.26 sec, RD: 0.00 sec

61

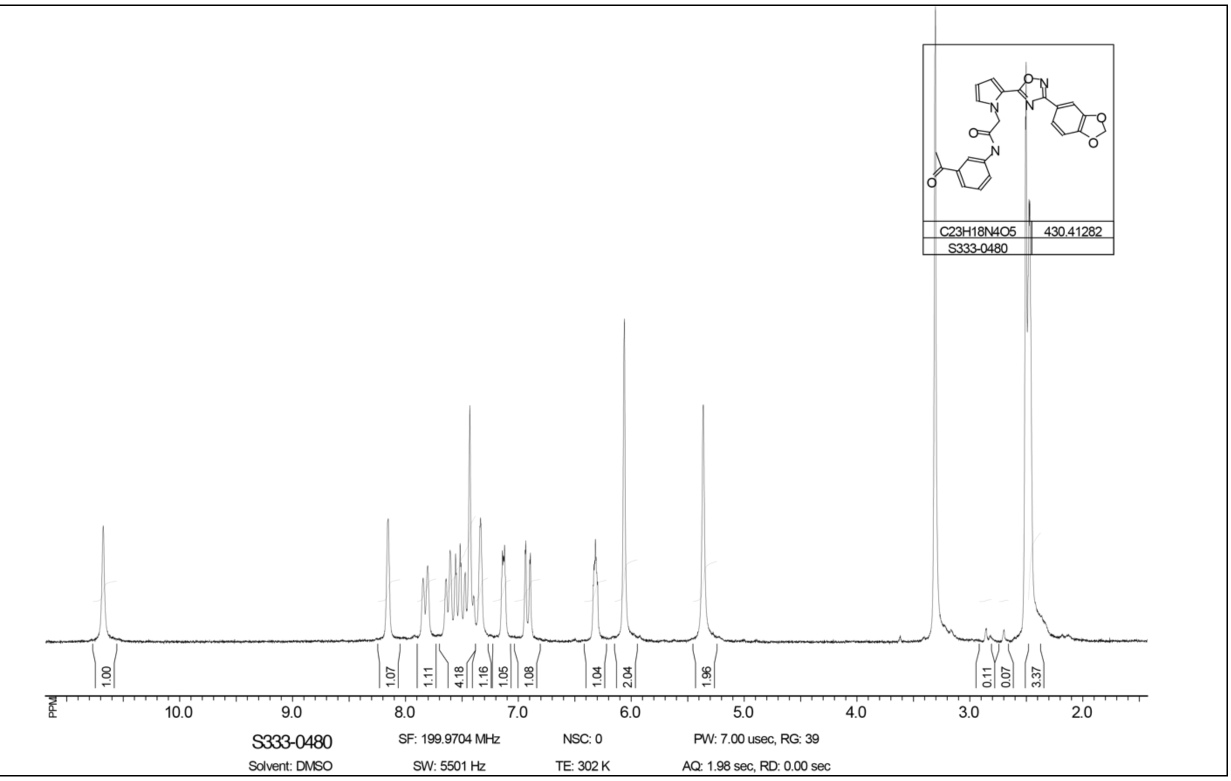
C23H18N4O5
430.41282
S333-0480
S333-0480
SF: 199.9704 MHz
NSC: 0
PW: 7.00 usec, RG: 39
Solvent: DMSO
SW: 5501 Hz
TE: 302 K
AQ: 1.98 sec, RD: 0.00 sec

62

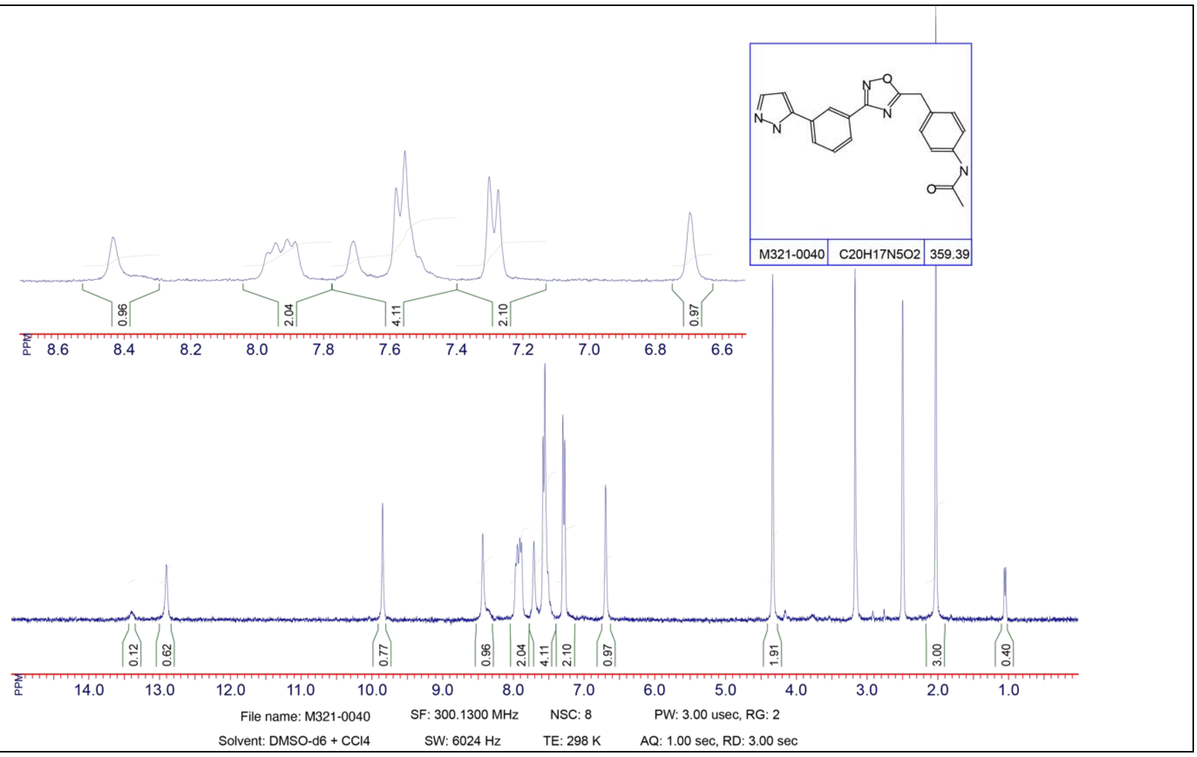
M321-0040
C20H17N5O2
359.39
File name: M321-0040
SF: 300.1300 MHz
NSC: 8
PW: 3.00 usec, RG: 2
Solvent: DMSO-d6 + CCl4
SW: 6024 Hz
TE: 298 K
AQ: 1.00 sec, RD: 3.00 sec

63

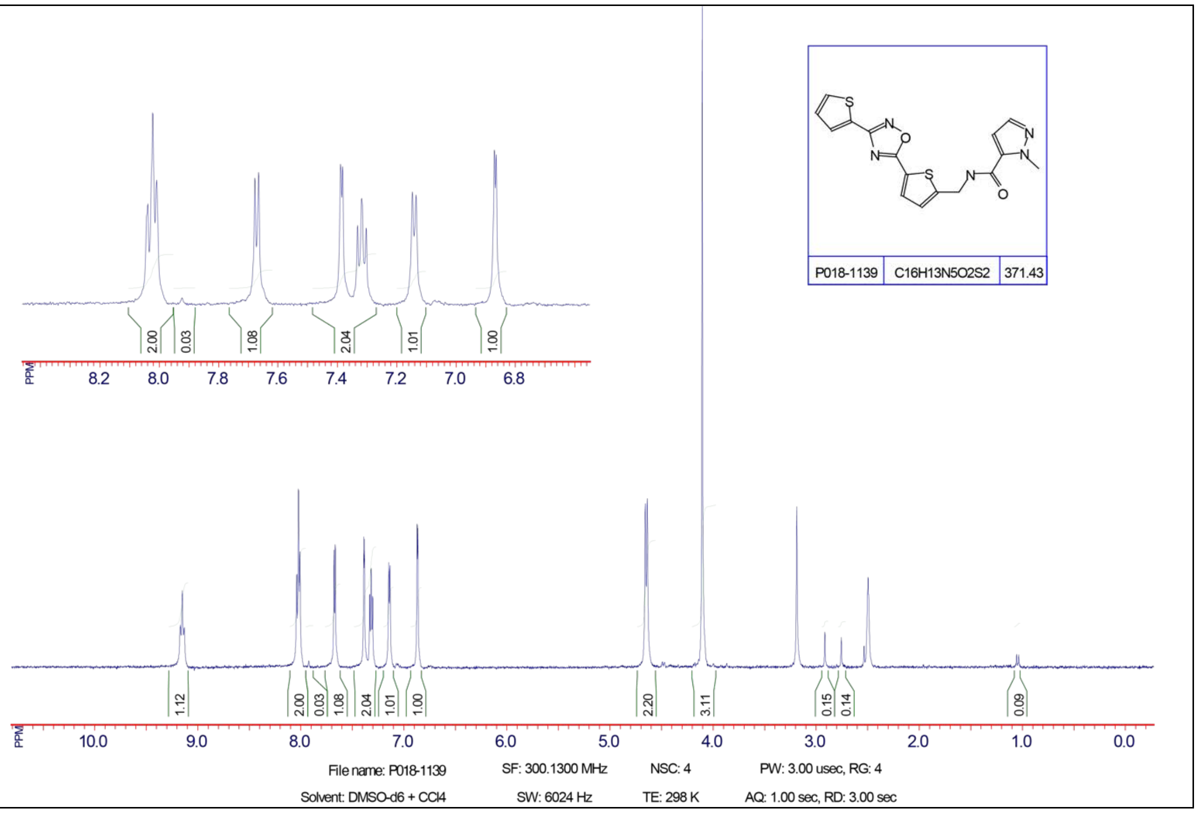
P018-1139
C16H13N5O2S2
371.43
File name: P018-1139
SF: 300.1300 MHz
NSC: 4
PW: 3.00 usec, RG: 4
Solvent: DMSO-d6 + CCl4
SW: 6024 Hz
TE: 298 K
AQ: 1.00 sec, RD: 3.00 sec
