# Supplementary material for: Humanized Candida and NanoBiT Assays Expedite Discovery of Bdf1 Bromodomain Inhibitors With Antifungal Potential
Source: Adv Sci (Weinh). 2025 Jan 16;12(10):2404260. doi: 10.1002/advs.202404260 (PMC11904993; doi:10.1002/advs.202404260)

ADVANCED SCIENCE
Open Access

Supporting Information

for *Adv. Sci.*, DOI 10.1002/advs.202404260

Humanized *Candida* and NanoBiT Assays Expedite Discovery of Bdf1 Bromodomain Inhibitors With Antifungal Potential

Kaiyao Wei, Marie Arlotto, Justin M. Overhulse, Tuan-Anh Dinh, Yingsheng Zhou, Nathan J. Dupper, Jiayi Yang, Boris A. Kashemirov, Hasan Dawi, Cécile Garnaud, Gaëlle Bourgine, Flore Mietton, Morgane Champleboux, Amédé Larabi, Yordan Hayat, Rose-Laure Indorato, Marjolaine Noirclerc-Savoye, Dimitrios Skoufias, Muriel Cornet, Gwenaël Rabut, Charles E. McKenna, Carlo Petosa* and Jérôme Govin**

Supplementary Data : Original images of gels and Western blots

Figure 3

C

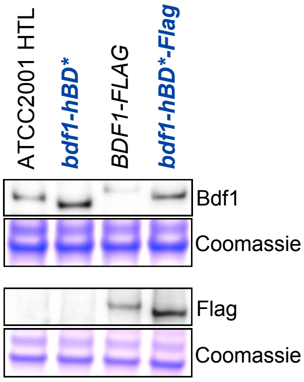

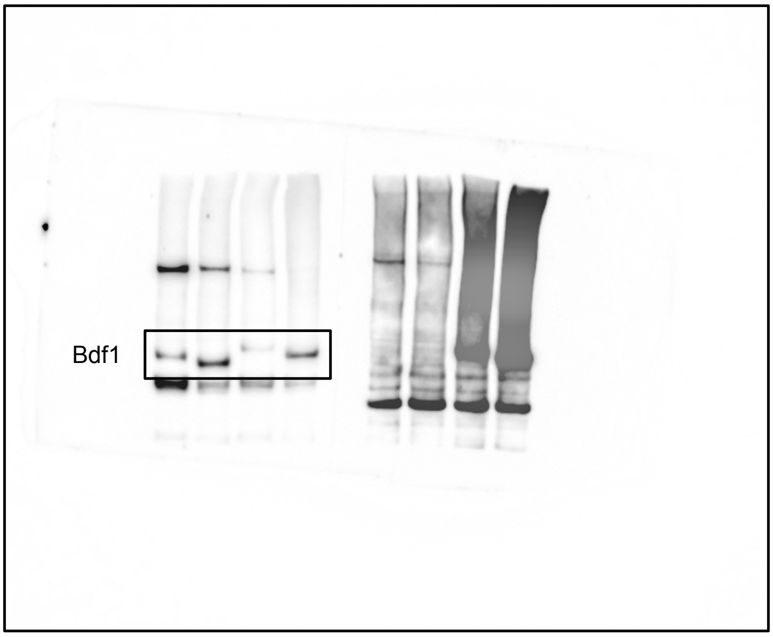

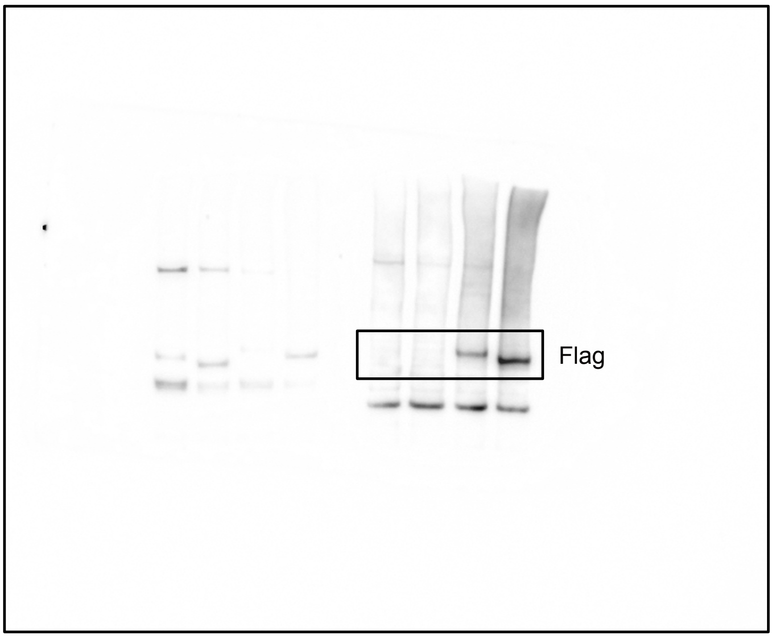

Same WB, with two different exposures

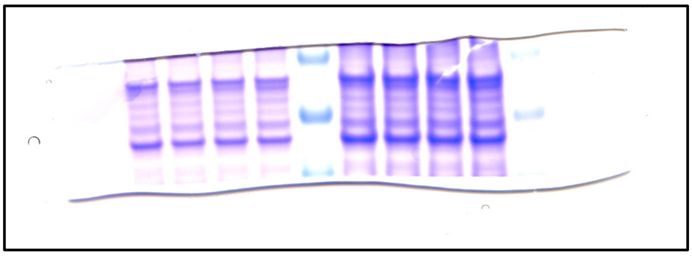

The bottom of the gel has been cut before transfer and Coomassie stained.

Figure S1

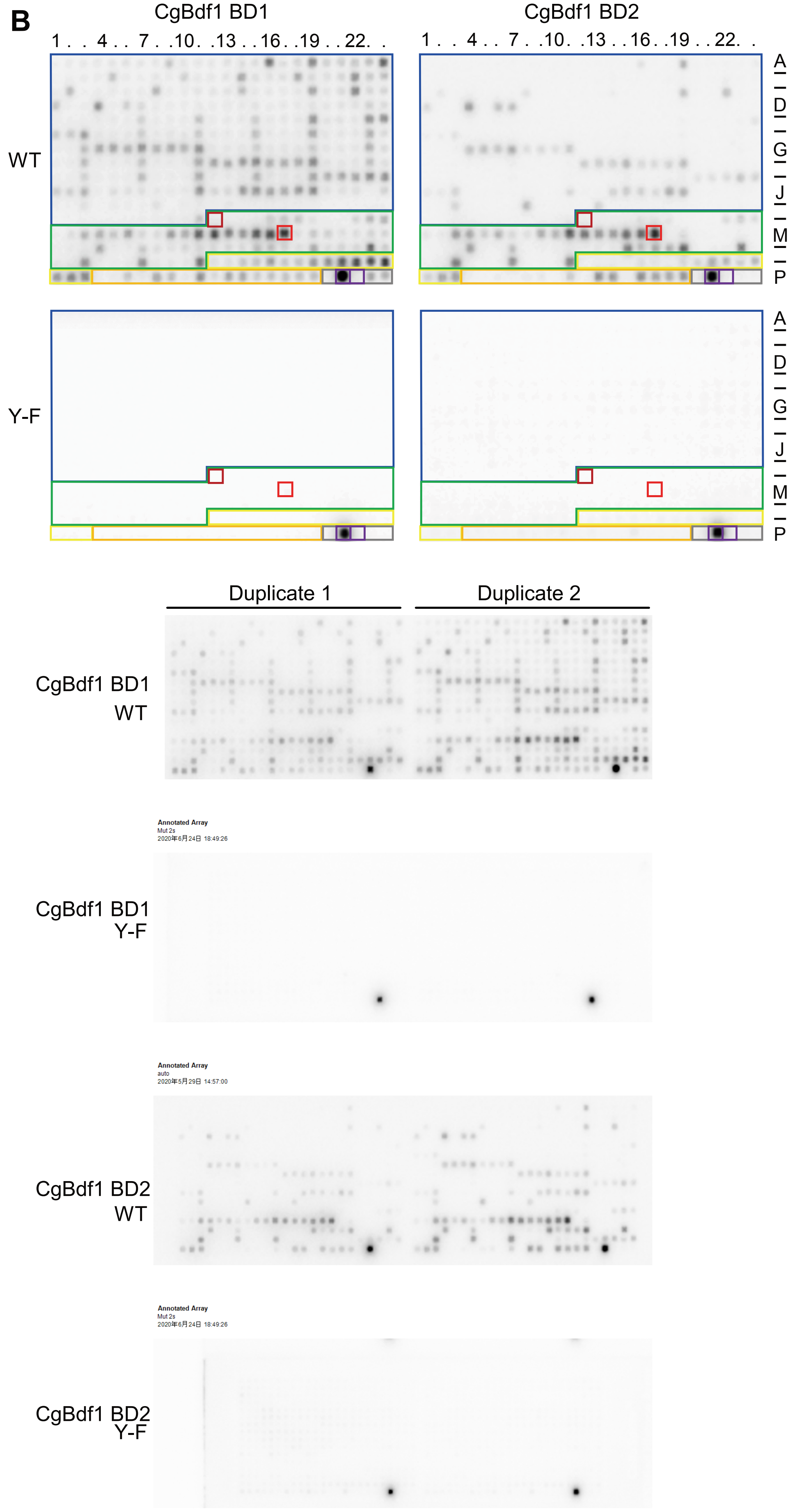
B
CgBdf1 BD1
CgBdf1 BD2
1 . . 4 . . 7 . .10. .13 . .16 . .19 . . 22. .
WT
Y-F
A
D
G
J
M
P
Duplicate 1
Duplicate 2
CgBdf1 BD1 WT
Annotated Array
Mut 2s
2020年6月24日 18:49:26
CgBdf1 BD1 Y-F
Annotated Array
auto
2020年5月29日 14:57:00
CgBdf1 BD2 WT
Annotated Array
Mut 2s
2020年6月24日 18:49:26
CgBdf1 BD2 Y-F

Figure S1

D

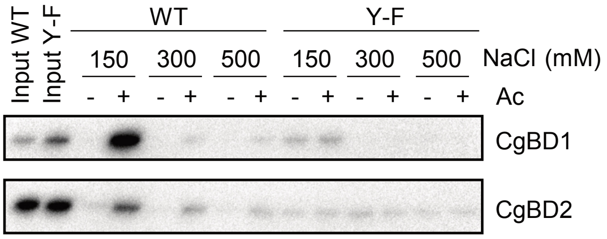
Input WT
Input Y-F
WT
Y-F
150
300
500
150
300
500
NaCl (mM)
- + - + - + - + - + - +
Ac
CgBD1
CgBD2

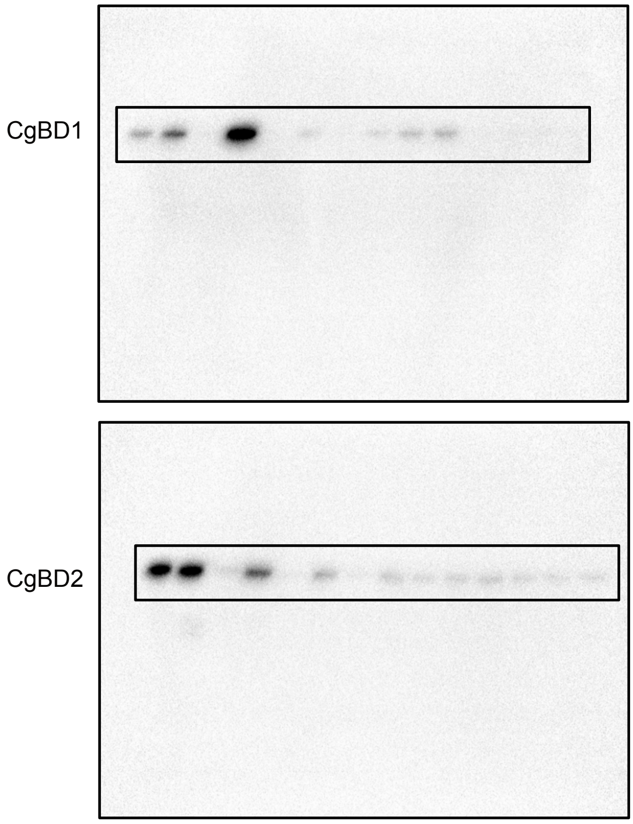
CgBD1
CgBD2

Figure S3

B

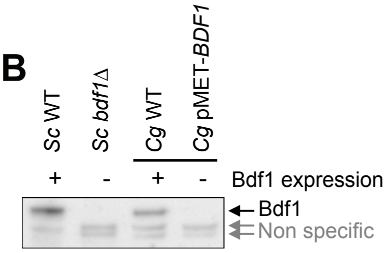

Sc WT
Sc bdf1Δ
Cg WT
Cg pMET-BDF1
+ - + -
Bdf1 expression
Bdf1
Non specific

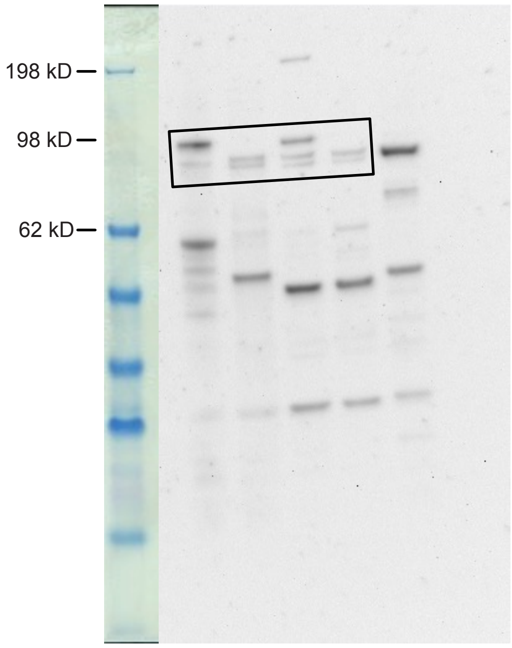

198 kD
98 kD
62 kD

C

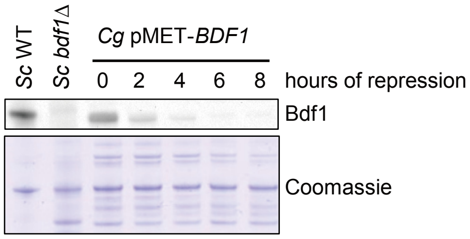

Sc WT
Sc bdf1Δ
Cg pMET-BDF1
0 2 4 6 8
hours of repression
Bdf1
Coomassie

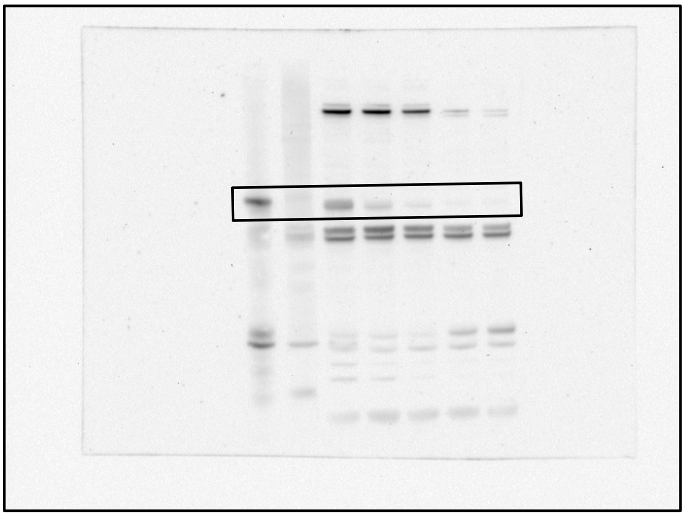

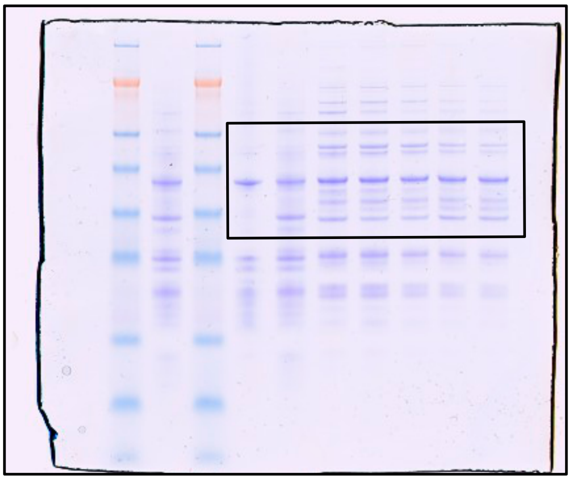

Figure S3

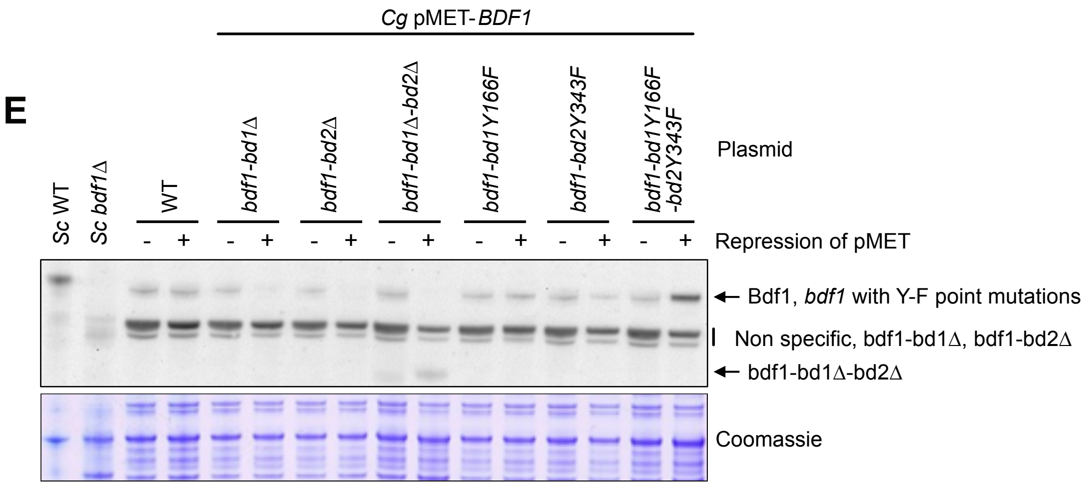

E
Cg pMET-BDF1
Sc WT
Sc bdf1Δ
WT
bdf1-bd1Δ
bdf1-bd2Δ
bdf1-bd1Δ-bd2Δ
bdf1-bd1Y166F
bdf1-bd2Y343F
bdf1-bd1Y166F -bd2Y343F
Plasmid
- + - + - + - + - + - + - +
Repression of pMET
Bdf1, bdf1 with Y-F point mutations
Non specific, bdf1-bd1Δ, bdf1-bd2Δ
bdf1-bd1Δ-bd2Δ
Coomassie

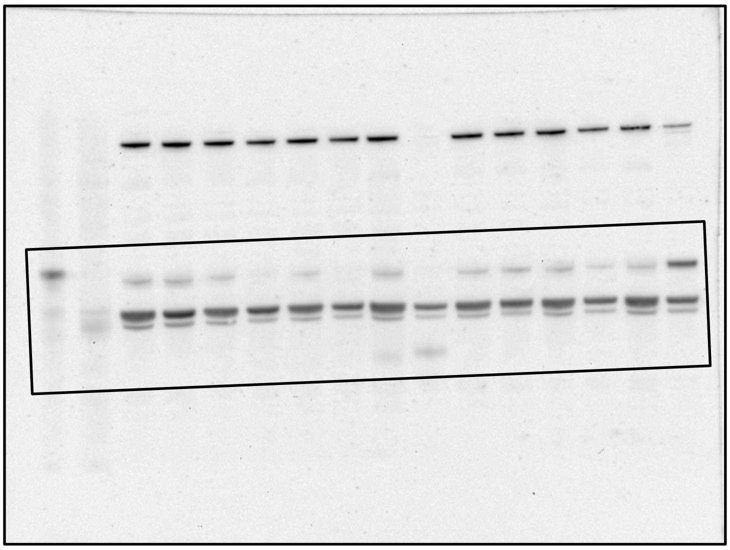

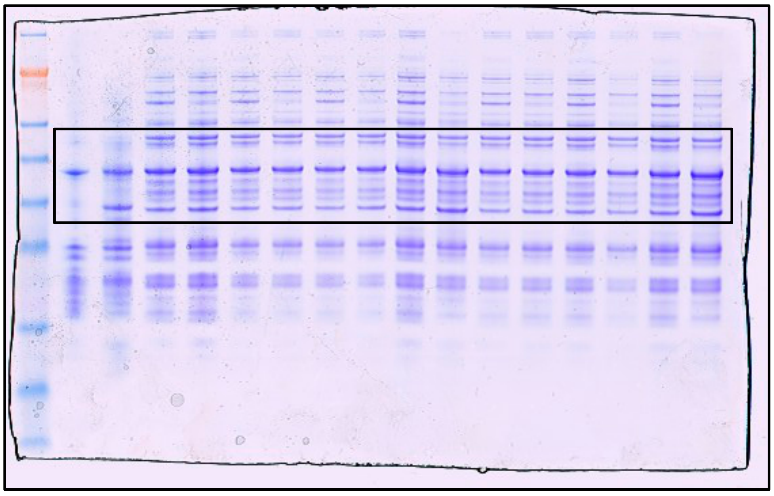

Figure S11

A

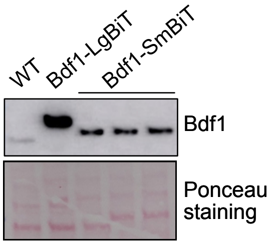

Bdf1

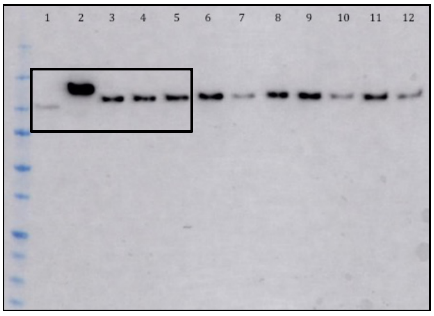

Ponceau
staining

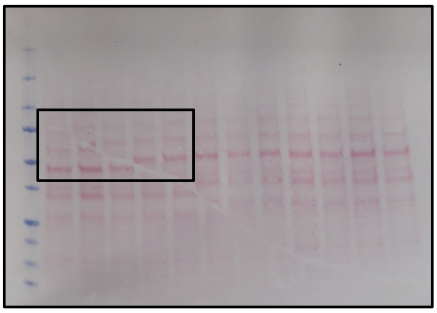

Supplement: Supplementary file 2 — Supporting Information [file ADVS-12-2404260-s003.pdf]
