# Supplementary material for: Humanized Candida and NanoBiT Assays Expedite Discovery of Bdf1 Bromodomain Inhibitors With Antifungal Potential
Source: Adv Sci (Weinh). 2025 Jan 16;12(10):2404260. doi: 10.1002/advs.202404260 (PMC11904993; doi:10.1002/advs.202404260)
Supplement: Supplementary file 3 — Supporting Information [file ADVS-12-2404260-s001.pdf]

ADVANCED SCIENCE
Open Access

Supporting Information

for *Adv. Sci.*, DOI 10.1002/advs.202404260

Humanized *Candida* and NanoBiT Assays Expedite Discovery of Bdf1 Bromodomain Inhibitors With Antifungal Potential

Kaiyao Wei, Marie Arlotto, Justin M. Overhulse, Tuan-Anh Dinh, Yingsheng Zhou, Nathan J. Dupper, Jiayi Yang, Boris A. Kashemirov, Hasan Dawi, Cécile Garnaud, Gaëlle Bourgine, Flore Mietton, Morgane Champleboux, Amédé Larabi, Yordan Hayat, Rose-Laure Indorato, Marjolaine Noirclerc-Savoye, Dimitrios Skoufias, Muriel Cornet, Gwenaël Rabut, Charles E. McKenna, Carlo Petosa* and Jérôme Govin**

PDB validation report for CgBD1 in the unbound state (8R6I)

Full wwPDB X-ray Structure Validation Report (i)

Nov 23, 2023 – 02:27 pm GMT

PDB ID	:	8R6I
Title	:	Crystal structure of Candida glabrata Bdf1 bromodomain 1 in the unbound state
Deposited on	:	2023-11-22
Resolution	:	1.08 Å(reported)

This wwPDB validation report is for manuscript review

This is a Full wwPDB X-ray Structure Validation Report.

This report is produced by the wwPDB biocuration pipeline after annotation of the structure.

We welcome your comments at *validation@mail.wwpdb.org*
A user guide is available at
https://www.wwpdb.org/validation/2017/XrayValidationReportHelp
with specific help available everywhere you see the (i) symbol.

The types of validation reports are described at
http://www.wwpdb.org/validation/2017/FAQs#types.

The following versions of software and data (see references (i)) were used in the production of this report:

MolProbity	:	4.02b-467
Xtriage (Phenix)	:	1.13
EDS	:	2.36
Percentile statistics	:	20191225.v01 (using entries in the PDB archive December 25th 2019)
Refmac	:	5.8.0158
CCP4	:	7.0.044 (Gargrove)
Ideal geometry (proteins)	:	Engh & Huber (2001)
Ideal geometry (DNA, RNA)	:	Parkinson et al. (1996)
Validation Pipeline (wwPDB-VP)	:	2.36

For Manuscript Review

1 Overall quality at a glance (i)

The following experimental techniques were used to determine the structure:
X-RAY DIFFRACTION

The reported resolution of this entry is 1.08 Å.

Percentile scores (ranging between 0-100) for global validation metrics of the entry are shown in the following graphic. The table shows the number of entries on which the scores are based.

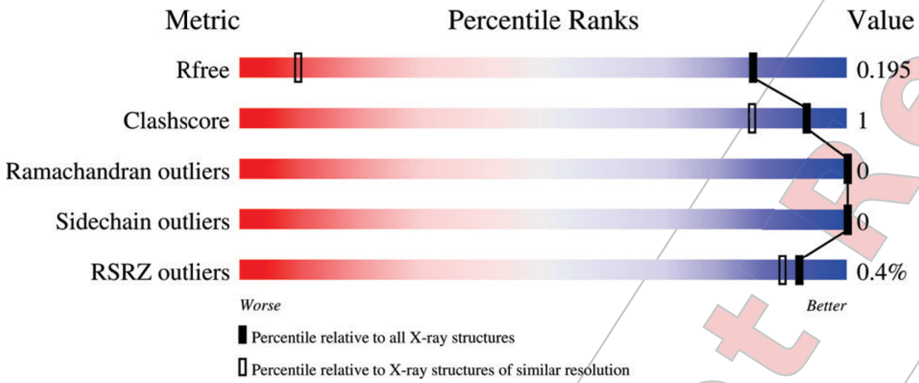

Metric	Whole archive (#Entries)	Similar resolution (#Entries, resolution range(Å))
R_{free}	130704	1386 (1.12-1.04)
Clashscore	141614	1021 (1.10-1.06)
Ramachandran outliers	138981	1381 (1.12-1.04)
Sidechain outliers	138945	1379 (1.12-1.04)
RSRZ outliers	127900	1359 (1.12-1.04)

The table below summarises the geometric issues observed across the polymeric chains and their fit to the electron density. The red, orange, yellow and green segments of the lower bar indicate the fraction of residues that contain outliers for >=3, 2, 1 and 0 types of geometric quality criteria respectively. A grey segment represents the fraction of residues that are not modelled. The numeric value for each fraction is indicated below the corresponding segment, with a dot representing fractions <=5% The upper red bar (where present) indicates the fraction of residues that have poor fit to the electron density. The numeric value is given above the bar.

Mol	Chain	Length	Quality of chain
1	A	114	97% •
1	B	114	% 99% •

For Manuscript Review

2 Entry composition (i)

There are 2 unique types of molecules in this entry. The entry contains 2200 atoms, of which 0 are hydrogens and 0 are deuteriums.

In the tables below, the ZeroOcc column contains the number of atoms modelled with zero occupancy, the AltConf column contains the number of residues with at least one atom in alternate conformation and the Trace column contains the number of residues modelled with at most 2 atoms.

- Molecule 1 is a protein called Candida glabrata strain CBS138 chromosome C complete sequence.

Mol	Chain	Residues	Atoms					ZeroOcc	AltConf	Trace
1	A	114	Total 907	C 577	N 161	O 162	S 7	0	0	0
1	B	113	Total 903	C 575	N 160	O 161	S 7	0	0	0

There are 8 discrepancies between the modelled and reference sequences:

Chain	Residue	Modelled	Actual	Comment	Reference
A	124	GLY	-	expression tag	UNP Q6FWV7
A	125	ALA	-	expression tag	UNP Q6FWV7
A	126	MET	-	expression tag	UNP Q6FWV7
A	127	GLY	-	expression tag	UNP Q6FWV7
B	124	GLY	-	expression tag	UNP Q6FWV7
B	125	ALA	-	expression tag	UNP Q6FWV7
B	126	MET	-	expression tag	UNP Q6FWV7
B	127	GLY	-	expression tag	UNP Q6FWV7

- Molecule 2 is water.

Mol	Chain	Residues	Atoms		ZeroOcc	AltConf
2	A	188	Total 188	O 188	0	0
2	B	202	Total 202	O 202	0	0

For Manuscript Review

3 Residue-property plots (i)

These plots are drawn for all protein, RNA, DNA and oligosaccharide chains in the entry. The first graphic for a chain summarises the proportions of the various outlier classes displayed in the second graphic. The second graphic shows the sequence view annotated by issues in geometry and electron density. Residues are color-coded according to the number of geometric quality criteria for which they contain at least one outlier: green = 0, yellow = 1, orange = 2 and red = 3 or more. A red dot above a residue indicates a poor fit to the electron density (RSRZ > 2). Stretches of 2 or more consecutive residues without any outlier are shown as a green connector. Residues present in the sample, but not in the model, are shown in grey.

- Molecule 1: Candida glabrata strain CBS138 chromosome C complete sequence

Chain A:

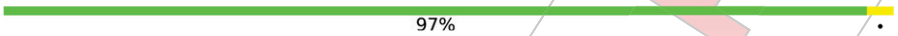

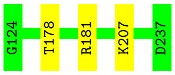

- Molecule 1: Candida glabrata strain CBS138 chromosome C complete sequence

Chain B:

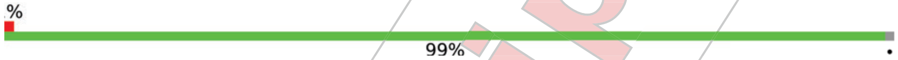

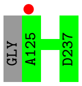

For Manuscript Review

4 Data and refinement statistics (i)

Property	Value	Source
Space group	P 1	Depositor
Cell constants<br>a, b, c, α, β, γ	22.89Å 40.59Å 48.87Å<br>98.14° 94.37° 94.77°	Depositor
Resolution (Å)	48.19 – 1.08<br>48.19 – 1.08	Depositor<br>EDS
% Data completeness<br>(in resolution range)	88.5 (48.19-1.08)<br>88.5 (48.19-1.08)	Depositor<br>EDS
R_{merge}	(Not available)	Depositor
R_{sym}	(Not available)	Depositor
$< I/\sigma(I) >$ [1]	1.03 (at 1.08Å)	Xtriage
Refinement program	PHENIX 1.20.1_4487	Depositor
R, R_{free}	0.174 , 0.195<br>0.173 , 0.195	Depositor<br>DCC
R_{free} test set	2100 reflections (3.17%)	wwPDB-VP
Wilson B-factor (Å^2)	9.9	Xtriage
Anisotropy	0.390	Xtriage
Bulk solvent k_{sol}(e/Å^3), B_{sol}(Å^2)	0.27 , 30.2	EDS
L-test for twinning[2]	$< \|L\| > = 0.49$, $< L^2 > = 0.32$	Xtriage
Estimated twinning fraction	No twinning to report.	Xtriage
F_o,F_c correlation	0.97	EDS
Total number of atoms	2200	wwPDB-VP
Average B, all atoms (Å^2)	13.0	wwPDB-VP

Xtriage's analysis on translational NCS is as follows: *The largest off-origin peak in the Patterson function is 11.91% of the height of the origin peak. No significant pseudotranslation is detected.*

[1] Intensities estimated from amplitudes.

[2] Theoretical values of $< |L| >$, $< L^2 >$ for acentric reflections are 0.5, 0.333 respectively for untwinned datasets, and 0.375, 0.2 for perfectly twinned datasets.

For Manuscript Review

5 Model quality (i)

5.1 Standard geometry (i)

The Z score for a bond length (or angle) is the number of standard deviations the observed value is removed from the expected value. A bond length (or angle) with $|Z| > 5$ is considered an outlier worth inspection. RMSZ is the root-mean-square of all Z scores of the bond lengths (or angles).

Mol	Chain	Bond lengths		Bond angles	
		RMSZ	#$\|Z\|$ >5	RMSZ	#$\|Z\|$ >5
1	A	0.43	0/926	0.62	0/1249
1	B	0.45	0/922	0.65	0/1244
All	All	0.44	0/1848	0.63	0/2493

There are no bond length outliers.

There are no bond angle outliers.

There are no chirality outliers.

There are no planarity outliers.

5.2 Too-close contacts (i)

In the following table, the Non-H and H(model) columns list the number of non-hydrogen atoms and hydrogen atoms in the chain respectively. The H(added) column lists the number of hydrogen atoms added and optimized by MolProbity. The Clashes column lists the number of clashes within the asymmetric unit, whereas Symm-Clashes lists symmetry-related clashes.

Mol	Chain	Non-H	H(model)	H(added)	Clashes	Symm-Clashes
1	A	907	0	923	5	0
1	B	903	0	920	0	0
2	A	188	0	0	3	2
2	B	202	0	0	0	3
All	All	2200	0	1843	5	3

The all-atom clashscore is defined as the number of clashes found per 1000 atoms (including hydrogen atoms). The all-atom clashscore for this structure is 1.

All (5) close contacts within the same asymmetric unit are listed below, sorted by their clash magnitude.

Atom-1	Atom-2	Interatomic distance (Å)	Clash overlap (Å)
1:A:181:ARG:NH1	2:A:301:HOH:O	2.29	0.64

Continued on next page...

For Manuscript Review

Continued from previous page...

Atom-1	Atom-2	Interatomic distance (Å)	Clash overlap (Å)
1:A:207:LYS:HE2	2:A:358:HOH:O	2.02	0.58
1:A:178:THR:OG1	1:A:181:ARG:NH2	2.47	0.48
1:A:178:THR:HA	1:A:181:ARG:HE	1.80	0.47
1:A:207:LYS:HG3	2:A:340:HOH:O	2.17	0.45

All (3) symmetry-related close contacts are listed below. The label for Atom-2 includes the symmetry operator and encoded unit-cell translations to be applied.

Atom-1	Atom-2	Interatomic distance (Å)	Clash overlap (Å)
2:A:434:HOH:O	2:B:452:HOH:O[1_556]	2.10	0.10
2:A:483:HOH:O	2:B:499:HOH:O[1_545]	2.11	0.09
2:B:344:HOH:O	2:B:392:HOH:O[1_655]	2.17	0.03

5.3 Torsion angles (i)

5.3.1 Protein backbone (i)

In the following table, the Percentiles column shows the percent Ramachandran outliers of the chain as a percentile score with respect to all X-ray entries followed by that with respect to entries of similar resolution.

The Analysed column shows the number of residues for which the backbone conformation was analysed, and the total number of residues.

Mol	Chain	Analysed	Favoured	Allowed	Outliers	Percentiles	
1	A	112/114 (98%)	112 (100%)	0	0	100	100
1	B	111/114 (97%)	111 (100%)	0	0	100	100
All	All	223/228 (98%)	223 (100%)	0	0	100	100

There are no Ramachandran outliers to report.

5.3.2 Protein sidechains (i)

In the following table, the Percentiles column shows the percent sidechain outliers of the chain as a percentile score with respect to all X-ray entries followed by that with respect to entries of similar resolution.

The Analysed column shows the number of residues for which the sidechain conformation was analysed, and the total number of residues.

For Manuscript Review

Mol	Chain	Analysed	Rotameric	Outliers	Percentiles	
1	A	97/97 (100%)	97 (100%)	0	100	100
1	B	97/97 (100%)	97 (100%)	0	100	100
All	All	194/194 (100%)	194 (100%)	0	100	100

There are no protein residues with a non-rotameric sidechain to report.

Sometimes sidechains can be flipped to improve hydrogen bonding and reduce clashes. There are no such sidechains identified.

5.3.3 RNA (i)

There are no RNA molecules in this entry.

5.4 Non-standard residues in protein, DNA, RNA chains (i)

There are no non-standard protein/DNA/RNA residues in this entry.

5.5 Carbohydrates (i)

There are no monosaccharides in this entry.

5.6 Ligand geometry (i)

There are no ligands in this entry.

5.7 Other polymers (i)

There are no such residues in this entry.

5.8 Polymer linkage issues (i)

There are no chain breaks in this entry.

For Manuscript Review

6 Fit of model and data (i)

6.1 Protein, DNA and RNA chains (i)

In the following table, the column labelled '#RSRZ> 2' contains the number (and percentage) of RSRZ outliers, followed by percent RSRZ outliers for the chain as percentile scores relative to all X-ray entries and entries of similar resolution. The OWAB column contains the minimum, median, 95th percentile and maximum values of the occupancy-weighted average B-factor per residue. The column labelled 'Q< 0.9' lists the number of (and percentage) of residues with an average occupancy less than 0.9.

Mol	Chain	Analysed	<RSRZ>	#RSRZ>2			OWAB(Å^2)	Q<0.9
1	A	114/114 (100%)	-0.22	0	100	100	8, 11, 18, 23	0
1	B	113/114 (99%)	-0.09	1 (0%)	84	80	7, 11, 19, 36	0
All	All	227/228 (99%)	-0.16	1 (0%)	92	89	7, 11, 19, 36	0

All (1) RSRZ outliers are listed below:

Mol	Chain	Res	Type	RSRZ
1	B	125	ALA	5.5

6.2 Non-standard residues in protein, DNA, RNA chains (i)

There are no non-standard protein/DNA/RNA residues in this entry.

6.3 Carbohydrates (i)

There are no monosaccharides in this entry.

6.4 Ligands (i)

There are no ligands in this entry.

6.5 Other polymers (i)

There are no such residues in this entry.

For Manuscript Review

PDB validation report for CgBD1 bound to 63 (8R6J)

Full wwPDB X-ray Structure Validation Report (i)

Nov 23, 2023 – 08:25 pm GMT

PDB ID	:	8R6J
Title	:	Crystal structure of Candida glabrata Bdf1 bromodomain 1 bound to a pyrazole ligand
Deposited on	:	2023-11-22
Resolution	:	1.82 Å(reported)

This wwPDB validation report is for manuscript review

This is a Full wwPDB X-ray Structure Validation Report.

This report is produced by the wwPDB biocuration pipeline after annotation of the structure.

We welcome your comments at *validation@mail.wwpdb.org*
A user guide is available at
https://www.wwpdb.org/validation/2017/XrayValidationReportHelp
with specific help available everywhere you see the (i) symbol.

The types of validation reports are described at
http://www.wwpdb.org/validation/2017/FAQs#types.

The following versions of software and data (see references (i)) were used in the production of this report:

MolProbity	:	4.02b-467
Mogul	:	1.8.4, CSD as541be (2020)
Xtriage (Phenix)	:	1.13
EDS	:	2.36
buster-report	:	1.1.7 (2018)
Percentile statistics	:	20191225.v01 (using entries in the PDB archive December 25th 2019)
Refmac	:	5.8.0158
CCP4	:	7.0.044 (Gargrove)
Ideal geometry (proteins)	:	Engh & Huber (2001)

For Manuscript Review

1 Overall quality at a glance (i)

The following experimental techniques were used to determine the structure:
X-RAY DIFFRACTION

The reported resolution of this entry is 1.82 Å.

Percentile scores (ranging between 0-100) for global validation metrics of the entry are shown in the following graphic. The table shows the number of entries on which the scores are based.

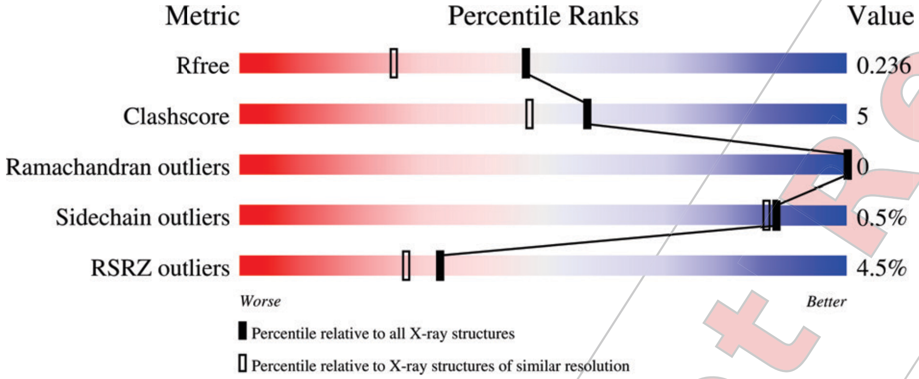

Metric	Whole archive (#Entries)	Similar resolution (#Entries, resolution range(Å))
R_{free}	130704	7484 (1.84-1.80)
Clashscore	141614	8401 (1.84-1.80)
Ramachandran outliers	138981	8290 (1.84-1.80)
Sidechain outliers	138945	8290 (1.84-1.80)
RSRZ outliers	127900	7371 (1.84-1.80)

The table below summarises the geometric issues observed across the polymeric chains and their fit to the electron density. The red, orange, yellow and green segments of the lower bar indicate the fraction of residues that contain outliers for >=3, 2, 1 and 0 types of geometric quality criteria respectively. A grey segment represents the fraction of residues that are not modelled. The numeric value for each fraction is indicated below the corresponding segment, with a dot representing fractions <=5% The upper red bar (where present) indicates the fraction of residues that have poor fit to the electron density. The numeric value is given above the bar.

Mol	Chain	Length	Quality of chain
1	A	114	2% / 85% 12% •
1	B	114	7% / 87% 11% •

Ideal geometry (DNA, RNA) : Parkinson et al. (1996)
Validation Pipeline (wwPDB-VP) : 2.36

For Manuscript Review

2 Entry composition (i)

There are 4 unique types of molecules in this entry. The entry contains 2027 atoms, of which 26 are hydrogens and 0 are deuteriums.

In the tables below, the ZeroOcc column contains the number of atoms modelled with zero occupancy, the AltConf column contains the number of residues with at least one atom in alternate conformation and the Trace column contains the number of residues modelled with at most 2 atoms.

- Molecule 1 is a protein called Candida glabrata strain CBS138 chromosome C complete sequence.

Mol	Chain	Residues	Atoms					ZeroOcc	AltConf	Trace
1	A	111	Total 890	C 567	N 158	O 159	S 6	0	0	0
1	B	111	Total 890	C 567	N 158	O 159	S 6	0	0	0

There are 8 discrepancies between the modelled and reference sequences:

Chain	Residue	Modelled	Actual	Comment	Reference
A	124	GLY	-	expression tag	UNP Q6FWV7
A	125	ALA	-	expression tag	UNP Q6FWV7
A	126	MET	-	expression tag	UNP Q6FWV7
A	127	GLY	-	expression tag	UNP Q6FWV7
B	124	GLY	-	expression tag	UNP Q6FWV7
B	125	ALA	-	expression tag	UNP Q6FWV7
B	126	MET	-	expression tag	UNP Q6FWV7
B	127	GLY	-	expression tag	UNP Q6FWV7

- Molecule 2 is 2-methyl- {N}-[[5-(3-thiophen-2-yl-1,2,4-oxadiazol-5-yl)thiophen-2-yl]met hyl]pyrazole-3-carboxamide (three-letter code: Y7F) (formula: $C_{16}H_{13}N_5O_2S_2$) (labeled as "Ligand of Interest" by depositor).

For Manuscript Review

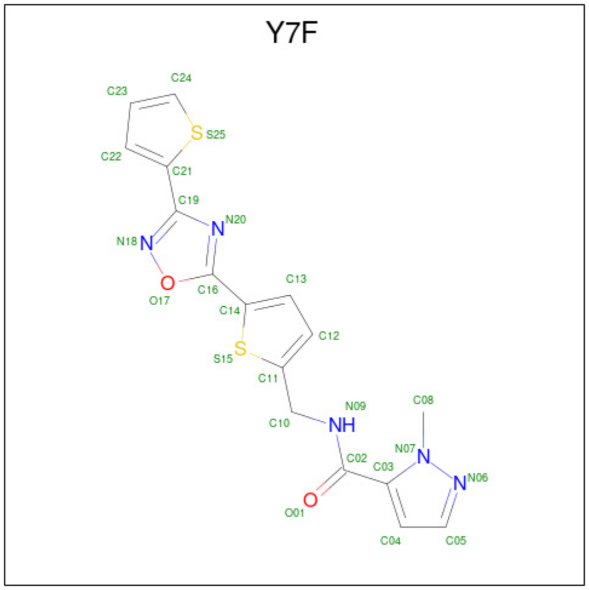

Mol	Chain	Residues	Atoms						ZeroOcc	AltConf
2	A	1	Total<br>38	C<br>16	H<br>13	N<br>5	O<br>2	S<br>2	0	0
2	B	1	Total<br>38	C<br>16	H<br>13	N<br>5	O<br>2	S<br>2	0	0

- Molecule 3 is SULFATE ION (three-letter code: SO4) (formula: O_4S).

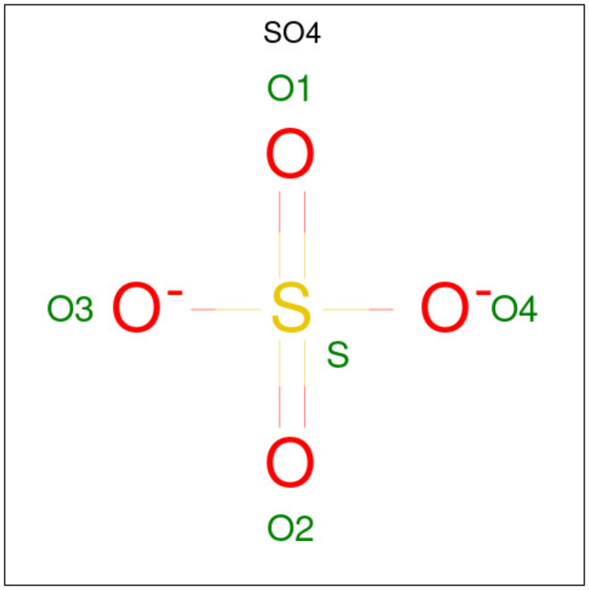

Mol	Chain	Residues	Atoms			ZeroOcc	AltConf
3	A	1	Total<br>5	O<br>4	S<br>1	0	0
3	B	1	Total<br>5	O<br>4	S<br>1	0	0

For Manuscript Review

- Molecule 4 is water.

Mol	Chain	Residues	Atoms	ZeroOcc	AltConf
4	A	87	Total O<br>87 87	0	0
4	B	74	Total O<br>74 74	0	0

For Manuscript Review

3 Residue-property plots (i)

These plots are drawn for all protein, RNA, DNA and oligosaccharide chains in the entry. The first graphic for a chain summarises the proportions of the various outlier classes displayed in the second graphic. The second graphic shows the sequence view annotated by issues in geometry and electron density. Residues are color-coded according to the number of geometric quality criteria for which they contain at least one outlier: green = 0, yellow = 1, orange = 2 and red = 3 or more. A red dot above a residue indicates a poor fit to the electron density (RSRZ > 2). Stretches of 2 or more consecutive residues without any outlier are shown as a green connector. Residues present in the sample, but not in the model, are shown in grey.

- Molecule 1: Candida glabrata strain CBS138 chromosome C complete sequence

Chain A:

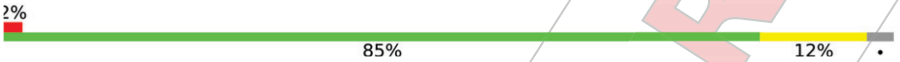

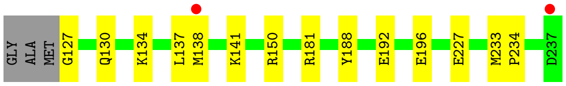

- Molecule 1: Candida glabrata strain CBS138 chromosome C complete sequence

Chain B:

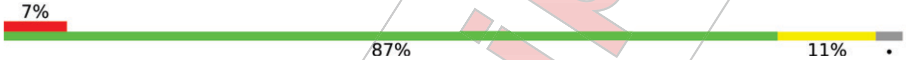

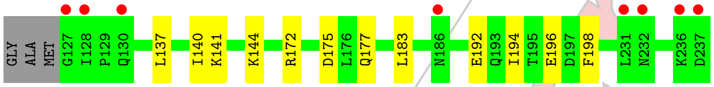

For Manuscript Review

4 Data and refinement statistics (i)

Property	Value	Source
Space group	C 2 2 21	Depositor
Cell constants<br>a, b, c, α, β, γ	32.84Å 142.62Å 93.55Å<br>90.00° 90.00° 90.00°	Depositor
Resolution (Å)	39.11 – 1.82<br>39.11 – 1.82	Depositor<br>EDS
% Data completeness<br>(in resolution range)	98.6 (39.11-1.82)<br>98.7 (39.11-1.82)	Depositor<br>EDS
R_{merge}	(Not available)	Depositor
R_{sym}	(Not available)	Depositor
$< I/\sigma(I) >$ [1]	1.56 (at 1.82Å)	Xtriage
Refinement program	PHENIX 1.20.1_4487	Depositor
R, R_{free}	0.199 , 0.234<br>0.198 , 0.236	Depositor<br>DCC
R_{free} test set	1001 reflections (5.00%)	wwPDB-VP
Wilson B-factor (Å^2)	22.8	Xtriage
Anisotropy	0.566	Xtriage
Bulk solvent k_{sol}(e/Å^3), B_{sol}(Å^2)	0.41 , 45.6	EDS
L-test for twinning[2]	$< \|L\| > = 0.49$, $< L^2 > = 0.33$	Xtriage
Estimated twinning fraction	No twinning to report.	Xtriage
F_o,F_c correlation	0.95	EDS
Total number of atoms	2027	wwPDB-VP
Average B, all atoms (Å^2)	31.0	wwPDB-VP

Xtriage's analysis on translational NCS is as follows: *The largest off-origin peak in the Patterson function is 8.90% of the height of the origin peak. No significant pseudotranslation is detected.*

[1] Intensities estimated from amplitudes.

[2] Theoretical values of $< |L| >$, $< L^2 >$ for acentric reflections are 0.5, 0.333 respectively for untwinned datasets, and 0.375, 0.2 for perfectly twinned datasets.

For Manuscript Review

5 Model quality (i)

5.1 Standard geometry (i)

Bond lengths and bond angles in the following residue types are not validated in this section: SO4, Y7F

The Z score for a bond length (or angle) is the number of standard deviations the observed value is removed from the expected value. A bond length (or angle) with $|Z| > 5$ is considered an outlier worth inspection. RMSZ is the root-mean-square of all Z scores of the bond lengths (or angles).

Mol	Chain	Bond lengths		Bond angles	
		RMSZ	#$\|Z\|$ >5	RMSZ	#$\|Z\|$ >5
1	A	0.28	0/909	0.48	0/1227
1	B	0.27	0/909	0.46	0/1227
All	All	0.27	0/1818	0.47	0/2454

There are no bond length outliers.

There are no bond angle outliers.

There are no chirality outliers.

There are no planarity outliers.

5.2 Too-close contacts (i)

In the following table, the Non-H and H(model) columns list the number of non-hydrogen atoms and hydrogen atoms in the chain respectively. The H(added) column lists the number of hydrogen atoms added and optimized by MolProbity. The Clashes column lists the number of clashes within the asymmetric unit, whereas Symm-Clashes lists symmetry-related clashes.

Mol	Chain	Non-H	H(model)	H(added)	Clashes	Symm-Clashes
1	A	890	0	906	9	0
1	B	890	0	906	8	0
2	A	25	13	0	0	0
2	B	25	13	0	1	0
3	A	5	0	0	0	0
3	B	5	0	0	0	0
4	A	87	0	0	3	1
4	B	74	0	0	4	0
All	All	2001	26	1812	18	1

The all-atom clashscore is defined as the number of clashes found per 1000 atoms (including hydrogen atoms). The all-atom clashscore for this structure is 5.

For Manuscript Review

All (18) close contacts within the same asymmetric unit are listed below, sorted by their clash magnitude.

Atom-1	Atom-2	Interatomic distance (Å)	Clash overlap (Å)
2:B:802:Y7F:S25	4:B:921:HOH:O	2.28	0.92
1:A:181:ARG:NH2	4:A:402:HOH:O	2.19	0.76
1:A:138:MET:SD	4:A:418:HOH:O	2.54	0.66
1:B:140:ILE:O	1:B:144:LYS:HG3	2.04	0.58
1:A:227:GLU:OE2	4:A:401:HOH:O	2.17	0.56
1:B:172:ARG:NH1	4:B:903:HOH:O	2.37	0.56
1:A:192:GLU:O	1:A:196:GLU:HG2	2.09	0.53
1:B:137:LEU:O	1:B:141:LYS:HG2	2.10	0.52
1:A:130:GLN:HG2	1:A:134:LYS:HE3	1.93	0.50
1:A:127:GLY:HA2	1:A:188:TYR:O	2.11	0.49
1:B:141:LYS:HD2	1:B:144:LYS:HE2	1.96	0.47
1:B:183:LEU:HD21	1:B:194:ILE:CD1	2.45	0.47
1:A:233:MET:SD	1:A:234:PRO:HD2	2.56	0.45
1:B:141:LYS:NZ	4:B:902:HOH:O	2.36	0.45
1:B:175:ASP:OD2	1:B:177:GLN:HB3	2.17	0.43
1:A:137:LEU:O	1:A:141:LYS:HG3	2.21	0.41
1:B:192:GLU:O	1:B:196:GLU:HG3	2.21	0.40
1:A:150:ARG:HD2	4:B:910:HOH:O	2.21	0.40

All (1) symmetry-related close contacts are listed below. The label for Atom-2 includes the symmetry operator and encoded unit-cell translations to be applied.

Atom-1	Atom-2	Interatomic distance (Å)	Clash overlap (Å)
4:A:402:HOH:O	4:A:469:HOH:O[3_655]	2.09	0.11

5.3 Torsion angles (i)

5.3.1 Protein backbone (i)

In the following table, the Percentiles column shows the percent Ramachandran outliers of the chain as a percentile score with respect to all X-ray entries followed by that with respect to entries of similar resolution.

The Analysed column shows the number of residues for which the backbone conformation was analysed, and the total number of residues.

Mol	Chain	Analysed	Favoured	Allowed	Outliers	Percentiles	
1	A	109/114 (96%)	109 (100%)	0	0	100	100

Continued on next page...

For Manuscript Review

Continued from previous page...

Mol	Chain	Analysed	Favoured	Allowed	Outliers	Percentiles	
1	B	109/114 (96%)	108 (99%)	1 (1%)	0	100	100
All	All	218/228 (96%)	217 (100%)	1 (0%)	0	100	100

There are no Ramachandran outliers to report.

5.3.2 Protein sidechains ⓘ

In the following table, the Percentiles column shows the percent sidechain outliers of the chain as a percentile score with respect to all X-ray entries followed by that with respect to entries of similar resolution.

The Analysed column shows the number of residues for which the sidechain conformation was analysed, and the total number of residues.

Mol	Chain	Analysed	Rotameric	Outliers	Percentiles	
1	A	96/97 (99%)	96 (100%)	0	100	100
1	B	96/97 (99%)	95 (99%)	1 (1%)	76	70
All	All	192/194 (99%)	191 (100%)	1 (0%)	88	87

All (1) residues with a non-rotameric sidechain are listed below:

Mol	Chain	Res	Type
1	B	198	PHE

Sometimes sidechains can be flipped to improve hydrogen bonding and reduce clashes. There are no such sidechains identified.

5.3.3 RNA ⓘ

There are no RNA molecules in this entry.

5.4 Non-standard residues in protein, DNA, RNA chains ⓘ

There are no non-standard protein/DNA/RNA residues in this entry.

5.5 Carbohydrates ⓘ

There are no monosaccharides in this entry.

For Manuscript Review

5.6 Ligand geometry (i)

4 ligands are modelled in this entry.

In the following table, the Counts columns list the number of bonds (or angles) for which Mogul statistics could be retrieved, the number of bonds (or angles) that are observed in the model and the number of bonds (or angles) that are defined in the Chemical Component Dictionary. The Link column lists molecule types, if any, to which the group is linked. The Z score for a bond length (or angle) is the number of standard deviations the observed value is removed from the expected value. A bond length (or angle) with $|Z| > 2$ is considered an outlier worth inspection. RMSZ is the root-mean-square of all Z scores of the bond lengths (or angles).

Mol	Type	Chain	Res	Link	Bond lengths			Bond angles		
					Counts	RMSZ	#$\|Z\| > 2$	Counts	RMSZ	#$\|Z\| > 2$
2	Y7F	B	802	-	22,28,28	1.66	5 (22%)	11,39,39	4.28	5 (45%)
2	Y7F	A	301	-	22,28,28	1.70	5 (22%)	11,39,39	4.46	6 (54%)
3	SO4	B	801	-	4,4,4	0.14	0	6,6,6	0.07	0
3	SO4	A	302	-	4,4,4	0.15	0	6,6,6	0.07	0

In the following table, the Chirals column lists the number of chiral outliers, the number of chiral centers analysed, the number of these observed in the model and the number defined in the Chemical Component Dictionary. Similar counts are reported in the Torsion and Rings columns. '-' means no outliers of that kind were identified.

Mol	Type	Chain	Res	Link	Chirals	Torsions	Rings
2	Y7F	B	802	-	-	0/4/17/17	0/4/4/4
2	Y7F	A	301	-	-	0/4/17/17	0/4/4/4

All (10) bond length outliers are listed below:

Mol	Chain	Res	Type	Atoms	Z	Observed(Å)	Ideal(Å)
2	A	301	Y7F	C02-N09	5.38	1.45	1.33
2	B	802	Y7F	C02-N09	5.21	1.45	1.33
2	A	301	Y7F	C19-C21	-3.36	1.38	1.48
2	B	802	Y7F	C19-C21	-3.36	1.38	1.48
2	B	802	Y7F	C19-N20	-2.47	1.32	1.35
2	A	301	Y7F	C10-C11	2.27	1.54	1.51
2	A	301	Y7F	C19-N20	-2.25	1.33	1.35
2	B	802	Y7F	C03-N07	-2.11	1.32	1.36
2	A	301	Y7F	O01-C02	-2.05	1.19	1.23
2	B	802	Y7F	O01-C02	-2.03	1.19	1.23

All (11) bond angle outliers are listed below:

For Manuscript Review

Mol	Chain	Res	Type	Atoms	Z	Observed(°)	Ideal(°)
2	A	301	Y7F	C19-N20-C16	9.38	108.72	101.13
2	B	802	Y7F	C19-N20-C16	9.24	108.61	101.13
2	A	301	Y7F	C23-C24-S25	-8.40	106.17	112.98
2	B	802	Y7F	C23-C24-S25	-8.22	106.31	112.98
2	A	301	Y7F	C05-N06-N07	4.98	109.41	104.23
2	B	802	Y7F	C05-N06-N07	4.55	108.97	104.23
2	A	301	Y7F	C03-N07-N06	-4.41	108.58	111.96
2	B	802	Y7F	C03-N07-N06	-3.90	108.97	111.96
2	A	301	Y7F	C11-C10-N09	-2.59	107.10	112.71
2	B	802	Y7F	C21-C19-N20	2.42	126.71	123.61
2	A	301	Y7F	C21-C19-N20	2.39	126.67	123.61

There are no chirality outliers.

There are no torsion outliers.

There are no ring outliers.

1 monomer is involved in 1 short contact:

Mol	Chain	Res	Type	Clashes	Symm-Clashes
2	B	802	Y7F	1	0

The following is a two-dimensional graphical depiction of Mogul quality analysis of bond lengths, bond angles, torsion angles, and ring geometry for all instances of the Ligand of Interest. In addition, ligands with molecular weight > 250 and outliers as shown on the validation Tables will also be included. For torsion angles, if less then 5% of the Mogul distribution of torsion angles is within 10 degrees of the torsion angle in question, then that torsion angle is considered an outlier. Any bond that is central to one or more torsion angles identified as an outlier by Mogul will be highlighted in the graph. For rings, the root-mean-square deviation (RMSD) between the ring in question and similar rings identified by Mogul is calculated over all ring torsion angles. If the average RMSD is greater than 60 degrees and the minimal RMSD between the ring in question and any Mogul-identified rings is also greater than 60 degrees, then that ring is considered an outlier. The outliers are highlighted in purple. The color gray indicates Mogul did not find sufficient equivalents in the CSD to analyse the geometry.

For Manuscript Review

Ligand Y7F B 802

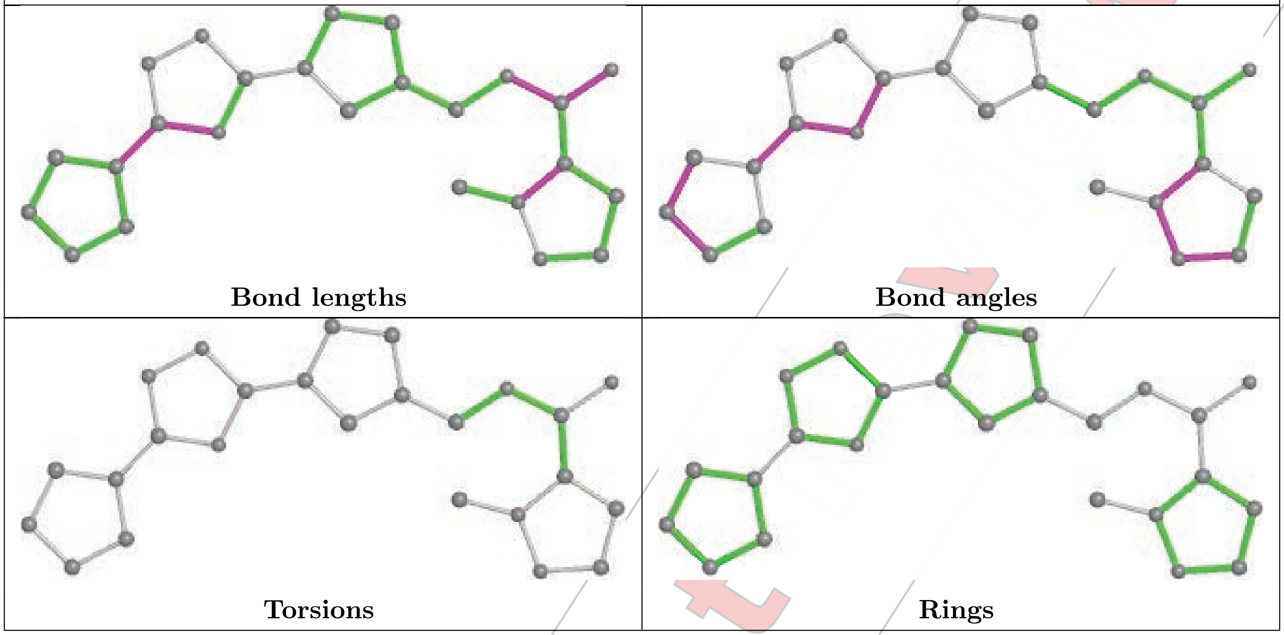

Ligand Y7F A 301

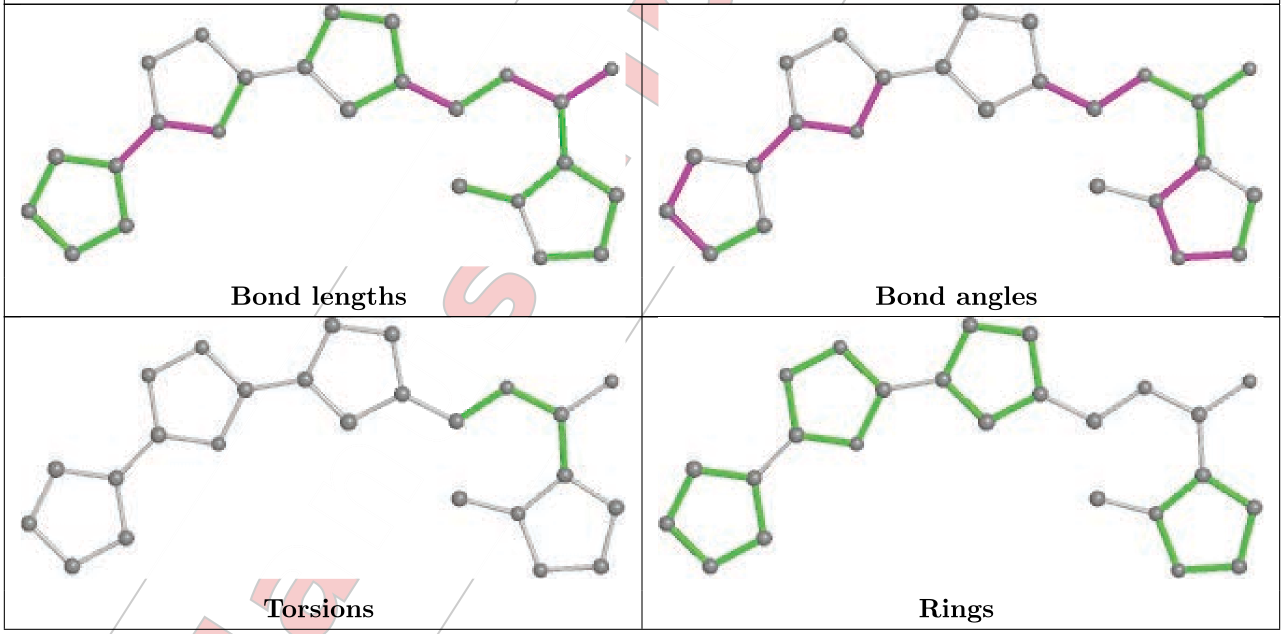

5.7 Other polymers (i)

There are no such residues in this entry.

5.8 Polymer linkage issues (i)

There are no chain breaks in this entry.

For Manuscript Review

6 Fit of model and data (i)

6.1 Protein, DNA and RNA chains (i)

In the following table, the column labelled '#RSRZ> 2' contains the number (and percentage) of RSRZ outliers, followed by percent RSRZ outliers for the chain as percentile scores relative to all X-ray entries and entries of similar resolution. The OWAB column contains the minimum, median, 95th percentile and maximum values of the occupancy-weighted average B-factor per residue. The column labelled 'Q< 0.9' lists the number of (and percentage) of residues with an average occupancy less than 0.9.

Mol	Chain	Analysed	<RSRZ>	#RSRZ>2			OWAB(Å^2)	Q<0.9
1	A	111/114 (97%)	0.02	2 (1%)	68	64	17, 24, 42, 58	0
1	B	111/114 (97%)	0.24	8 (7%)	15	12	18, 32, 55, 59	0
All	All	222/228 (97%)	0.13	10 (4%)	33	27	17, 28, 52, 59	0

All (10) RSRZ outliers are listed below:

Mol	Chain	Res	Type	RSRZ
1	A	237	ASP	3.6
1	B	231	LEU	3.5
1	B	186	ASN	3.0
1	B	232	ASN	2.4
1	B	130	GLN	2.3
1	B	237	ASP	2.2
1	A	138	MET	2.1
1	B	127	GLY	2.1
1	B	128	ILE	2.0
1	B	236	LYS	2.0

6.2 Non-standard residues in protein, DNA, RNA chains (i)

There are no non-standard protein/DNA/RNA residues in this entry.

6.3 Carbohydrates (i)

There are no monosaccharides in this entry.

For Manuscript Review

6.4 Ligands (i)

In the following table, the Atoms column lists the number of modelled atoms in the group and the number defined in the chemical component dictionary. The B-factors column lists the minimum, median, 95th percentile and maximum values of B factors of atoms in the group. The column labelled 'Q< 0.9' lists the number of atoms with occupancy less than 0.9.

Mol	Type	Chain	Res	Atoms	RSCC	RSR	B-factors($Å^2$)	Q<0.9
2	Y7F	B	802	25/25	0.93	0.13	19,29,54,60	0
2	Y7F	A	301	25/25	0.94	0.10	16,23,37,44	0
3	SO4	B	801	5/5	0.96	0.12	40,43,46,48	0
3	SO4	A	302	5/5	0.97	0.12	37,40,40,45	0

The following is a graphical depiction of the model fit to experimental electron density of all instances of the Ligand of Interest. In addition, ligands with molecular weight > 250 and outliers as shown on the geometry validation Tables will also be included. Each fit is shown from different orientation to approximate a three-dimensional view.

Electron density around Y7F B 802:

$2mF_o$-DF_c (at 0.7 rmsd) in gray
mF_o-DF_c (at 3 rmsd) in purple (negative)
and green (positive)

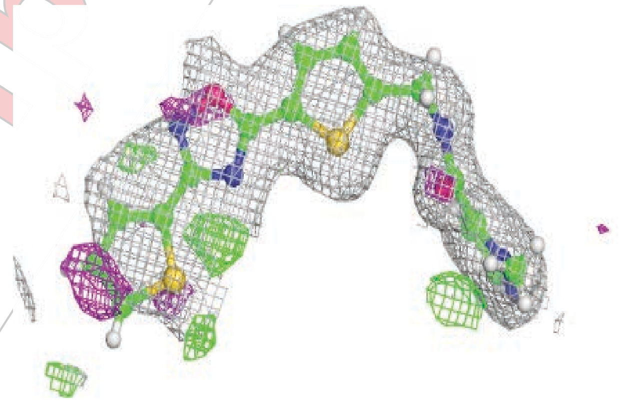

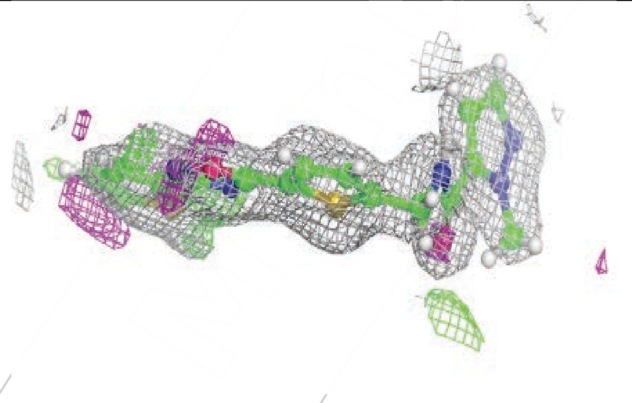

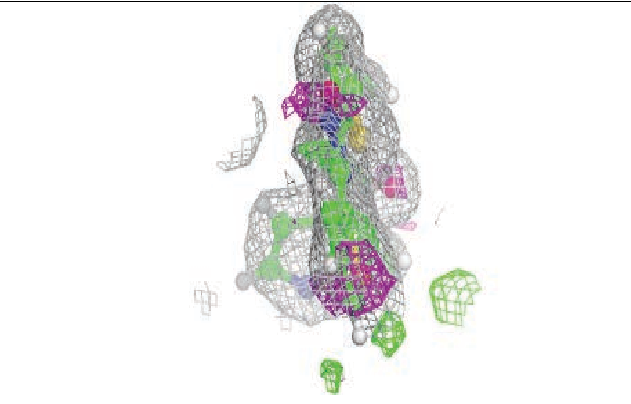

For Manuscript Review

Electron density around Y7F A 301:

$2mF_o$-DF_c (at 0.7 rmsd) in gray
mF_o-DF_c (at 3 rmsd) in purple (negative) and green (positive)

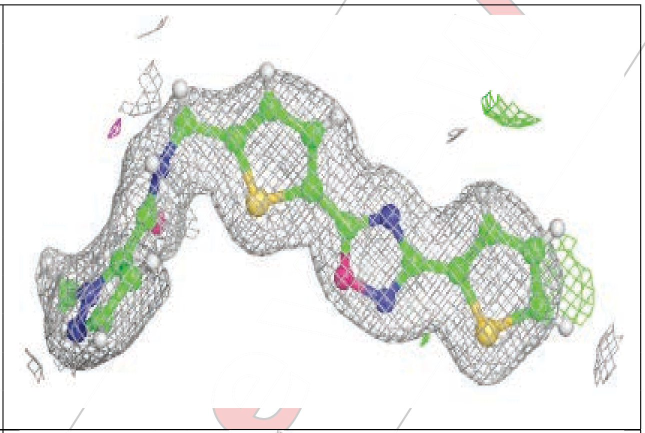

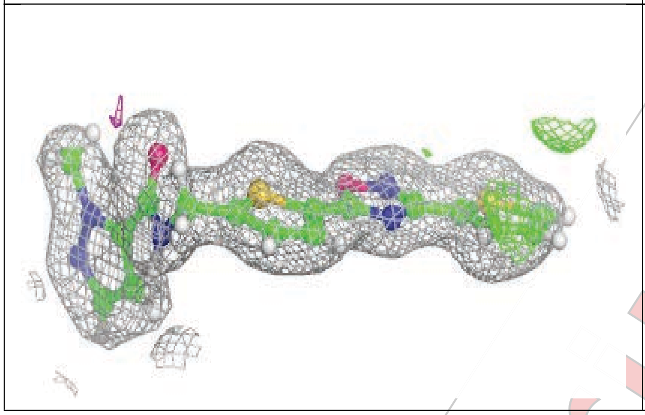

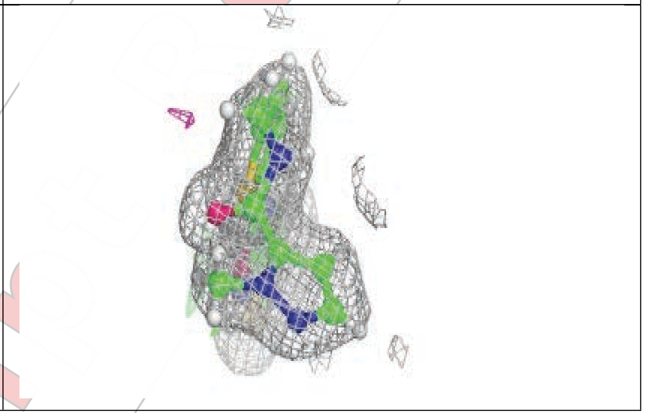

6.5 Other polymers (i)

There are no such residues in this entry.

For Manuscript Review

PDB validation report for CgBD1 bound to 24c (8R6K)

Full wwPDB X-ray Structure Validation Report (i)

Nov 23, 2023 – 07:00 pm GMT

PDB ID	:	8R6K
Title	:	Crystal structure of Candida glabrata Bdf1 bromodomain 1 bound to a phenyl-triazine ligand
Deposited on	:	2023-11-22
Resolution	:	2.19 Å(reported)

This wwPDB validation report is for manuscript review

This is a Full wwPDB X-ray Structure Validation Report.

This report is produced by the wwPDB biocuration pipeline after annotation of the structure.

We welcome your comments at *validation@mail.wwpdb.org*
A user guide is available at
https://www.wwpdb.org/validation/2017/XrayValidationReportHelp
with specific help available everywhere you see the (i) symbol.

The types of validation reports are described at
http://www.wwpdb.org/validation/2017/FAQs#types.

The following versions of software and data (see references (i)) were used in the production of this report:

MolProbity	:	4.02b-467
Mogul	:	1.8.4, CSD as541be (2020)
Xtriage (Phenix)	:	1.13
EDS	:	2.36
buster-report	:	1.1.7 (2018)
Percentile statistics	:	20191225.v01 (using entries in the PDB archive December 25th 2019)
Refmac	:	5.8.0158
CCP4	:	7.0.044 (Gargrove)
Ideal geometry (proteins)	:	Engh & Huber (2001)

For Manuscript Review

1 Overall quality at a glance (i)

The following experimental techniques were used to determine the structure:
X-RAY DIFFRACTION

The reported resolution of this entry is 2.19 Å.

Percentile scores (ranging between 0-100) for global validation metrics of the entry are shown in the following graphic. The table shows the number of entries on which the scores are based.

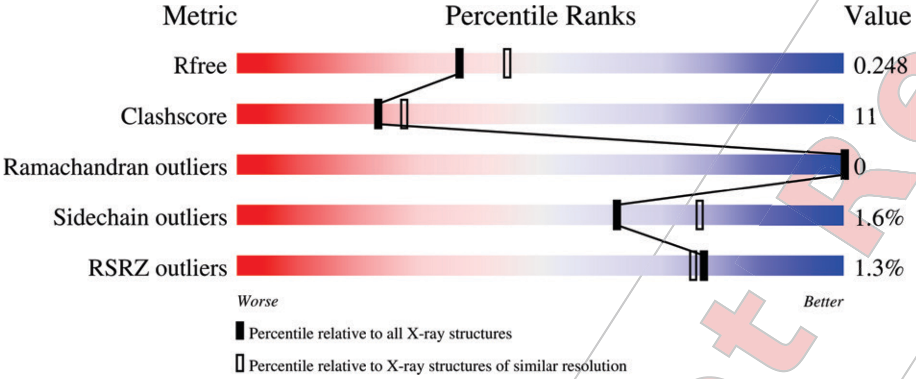

Metric	Whole archive (#Entries)	Similar resolution (#Entries, resolution range(Å))
R_{free}	130704	4898 (2.20-2.20)
Clashscore	141614	5594 (2.20-2.20)
Ramachandran outliers	138981	5503 (2.20-2.20)
Sidechain outliers	138945	5504 (2.20-2.20)
RSRZ outliers	127900	4800 (2.20-2.20)

The table below summarises the geometric issues observed across the polymeric chains and their fit to the electron density. The red, orange, yellow and green segments of the lower bar indicate the fraction of residues that contain outliers for >=3, 2, 1 and 0 types of geometric quality criteria respectively. A grey segment represents the fraction of residues that are not modelled. The numeric value for each fraction is indicated below the corresponding segment, with a dot representing fractions <=5% The upper red bar (where present) indicates the fraction of residues that have poor fit to the electron density. The numeric value is given above the bar.

Mol	Chain	Length	Quality of chain
1	A	114	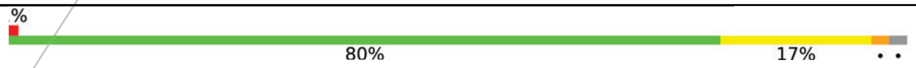
1	B	114	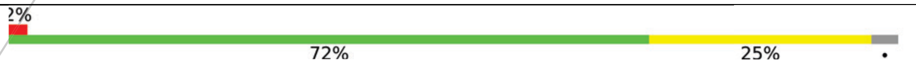
1	C	114	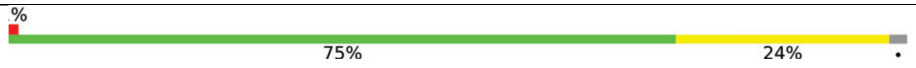

Continued on next page...

Ideal geometry (DNA, RNA) : Parkinson et al. (1996)
Validation Pipeline (wwPDB-VP) : 2.36

For Manuscript Review

Continued from previous page...

Mol	Chain	Length	Quality of chain
1	D	114	2% 74% 25% •

For Manuscript Review

2 Entry composition (i)

There are 5 unique types of molecules in this entry. The entry contains 4156 atoms, of which 54 are hydrogens and 0 are deuteriums.

In the tables below, the ZeroOcc column contains the number of atoms modelled with zero occupancy, the AltConf column contains the number of residues with at least one atom in alternate conformation and the Trace column contains the number of residues modelled with at most 2 atoms.

- Molecule 1 is a protein called Candida glabrata strain CBS138 chromosome C complete sequence.

Mol	Chain	Residues	Atoms					ZeroOcc	AltConf	Trace
1	A	112	Total 898	C 572	N 159	O 160	S 7	0	0	0
1	B	111	Total 889	C 568	N 158	O 156	S 7	0	0	0
1	C	112	Total 898	C 572	N 159	O 160	S 7	0	0	0
1	D	112	Total 898	C 572	N 159	O 160	S 7	0	0	0

There are 16 discrepancies between the modelled and reference sequences:

Chain	Residue	Modelled	Actual	Comment	Reference
A	124	GLY	-	expression tag	UNP Q6FWV7
A	125	ALA	-	expression tag	UNP Q6FWV7
A	126	MET	-	expression tag	UNP Q6FWV7
A	127	GLY	-	expression tag	UNP Q6FWV7
B	124	GLY	-	expression tag	UNP Q6FWV7
B	125	ALA	-	expression tag	UNP Q6FWV7
B	126	MET	-	expression tag	UNP Q6FWV7
B	127	GLY	-	expression tag	UNP Q6FWV7
C	124	GLY	-	expression tag	UNP Q6FWV7
C	125	ALA	-	expression tag	UNP Q6FWV7
C	126	MET	-	expression tag	UNP Q6FWV7
C	127	GLY	-	expression tag	UNP Q6FWV7
D	124	GLY	-	expression tag	UNP Q6FWV7
D	125	ALA	-	expression tag	UNP Q6FWV7
D	126	MET	-	expression tag	UNP Q6FWV7
D	127	GLY	-	expression tag	UNP Q6FWV7

- Molecule 2 is 6-methyl- {N}-[(5-methylfuran-2-yl)methyl]-3-(4-methylphenyl)-1,2,4-triazin-5-amine (three-letter code: Y78) (formula: $C_{17}H_{18}N_4O$) (labeled as "Ligand of Interest" by depositor).

For Manuscript Review

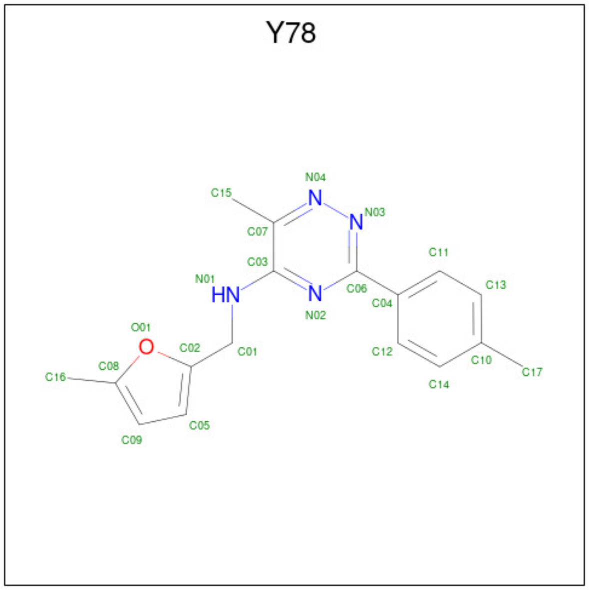

Mol	Chain	Residues	Atoms	ZeroOcc	AltConf
2	A	1	Total C H N O<br>40 17 18 4 1	0	0
2	B	1	Total C H N O<br>40 17 18 4 1	0	0
2	C	1	Total C H N O<br>40 17 18 4 1	0	0

- Molecule 3 is PHOSPHATE ION (three-letter code: PO4) (formula: O_4P).

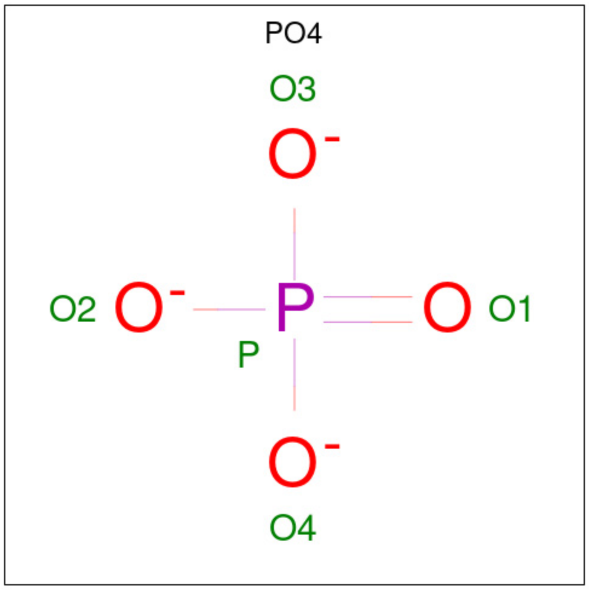

Mol	Chain	Residues	Atoms	ZeroOcc	AltConf
3	A	1	Total O P<br>5 4 1	0	0

Continued on next page...

For Manuscript Review

Continued from previous page...

Mol	Chain	Residues	Atoms	ZeroOcc	AltConf
3	A	1	Total O P<br>5 4 1	0	0
3	C	1	Total O P<br>5 4 1	0	0
3	D	1	Total O P<br>5 4 1	0	0

- Molecule 4 is POTASSIUM ION (three-letter code: K) (formula: K).

Mol	Chain	Residues	Atoms	ZeroOcc	AltConf
4	A	1	Total K<br>1 1	0	0

- Molecule 5 is water.

Mol	Chain	Residues	Atoms	ZeroOcc	AltConf
5	A	116	Total O<br>116 116	0	0
5	B	100	Total O<br>100 100	0	0
5	C	126	Total O<br>126 126	0	0
5	D	90	Total O<br>90 90	0	0

For Manuscript Review

3 Residue-property plots (i)

These plots are drawn for all protein, RNA, DNA and oligosaccharide chains in the entry. The first graphic for a chain summarises the proportions of the various outlier classes displayed in the second graphic. The second graphic shows the sequence view annotated by issues in geometry and electron density. Residues are color-coded according to the number of geometric quality criteria for which they contain at least one outlier: green = 0, yellow = 1, orange = 2 and red = 3 or more. A red dot above a residue indicates a poor fit to the electron density (RSRZ > 2). Stretches of 2 or more consecutive residues without any outlier are shown as a green connector. Residues present in the sample, but not in the model, are shown in grey.

• Molecule 1: Candida glabrata strain CBS138 chromosome C complete sequence

Chain A:

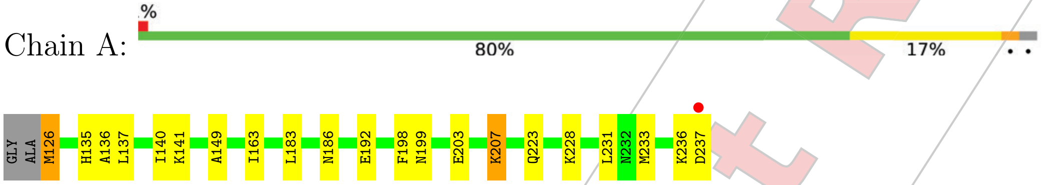

• Molecule 1: Candida glabrata strain CBS138 chromosome C complete sequence

Chain B:

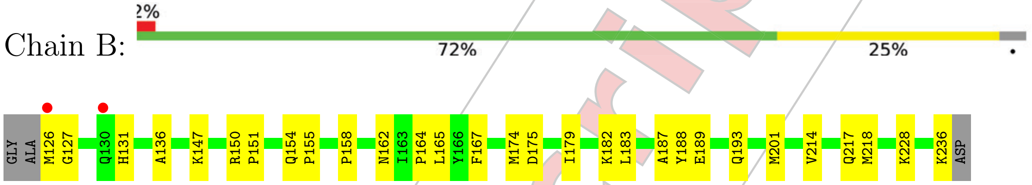

• Molecule 1: Candida glabrata strain CBS138 chromosome C complete sequence

Chain C:

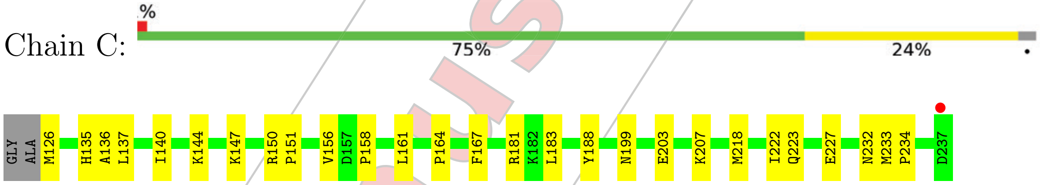

• Molecule 1: Candida glabrata strain CBS138 chromosome C complete sequence

Chain D:

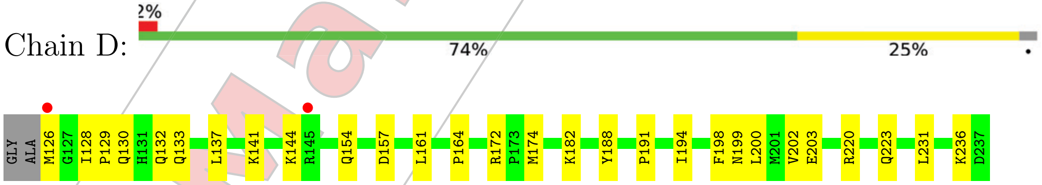

For Manuscript Review

4 Data and refinement statistics (i)

Property	Value	Source
Space group	I 4 2 2	Depositor
Cell constants<br>a, b, c, α, β, γ	128.70Å 128.70Å 148.21Å<br>90.00° 90.00° 90.00°	Depositor
Resolution (Å)	57.46 – 2.19<br>57.46 – 2.19	Depositor<br>EDS
% Data completeness<br>(in resolution range)	99.9 (57.46-2.19)<br>100.0 (57.46-2.19)	Depositor<br>EDS
R_{merge}	(Not available)	Depositor
R_{sym}	(Not available)	Depositor
$< I/\sigma(I) >$ [1]	2.86 (at 2.20Å)	Xtriage
Refinement program	PHENIX 1.20.1_4487	Depositor
R, R_{free}	0.209 , 0.247<br>0.209 , 0.248	Depositor<br>DCC
R_{free} test set	1662 reflections (5.16%)	wwPDB-VP
Wilson B-factor (Å^2)	23.1	Xtriage
Anisotropy	0.025	Xtriage
Bulk solvent k_{sol}(e/Å^3), B_{sol}(Å^2)	0.33 , 40.2	EDS
L-test for twinning[2]	$< \|L\| > = 0.46$, $< L^2 > = 0.28$	Xtriage
Estimated twinning fraction	No twinning to report.	Xtriage
F_o,F_c correlation	0.92	EDS
Total number of atoms	4156	wwPDB-VP
Average B, all atoms (Å^2)	28.0	wwPDB-VP

Xtriage's analysis on translational NCS is as follows: *The largest off-origin peak in the Patterson function is 3.98% of the height of the origin peak. No significant pseudotranslation is detected.*

[1] Intensities estimated from amplitudes.

[2] Theoretical values of $< |L| >$, $< L^2 >$ for acentric reflections are 0.5, 0.333 respectively for untwinned datasets, and 0.375, 0.2 for perfectly twinned datasets.

For Manuscript Review

5 Model quality (i)

5.1 Standard geometry (i)

Bond lengths and bond angles in the following residue types are not validated in this section: Y78, PO4, K

The Z score for a bond length (or angle) is the number of standard deviations the observed value is removed from the expected value. A bond length (or angle) with $|Z| > 5$ is considered an outlier worth inspection. RMSZ is the root-mean-square of all Z scores of the bond lengths (or angles).

Mol	Chain	Bond lengths		Bond angles	
		RMSZ	$\#\|Z\| > 5$	RMSZ	$\#\|Z\| > 5$
1	A	0.38	0/917	0.50	0/1237
1	B	0.40	0/908	0.49	0/1226
1	C	0.63	1/917 (0.1%)	0.51	0/1237
1	D	0.31	0/917	0.47	0/1237
All	All	0.45	1/3659 (0.0%)	0.49	0/4937

Chiral center outliers are detected by calculating the chiral volume of a chiral center and verifying if the center is modelled as a planar moiety or with the opposite hand.A planarity outlier is detected by checking planarity of atoms in a peptide group, atoms in a mainchain group or atoms of a sidechain that are expected to be planar.

Mol	Chain	#Chirality outliers	#Planarity outliers
1	C	0	1

All (1) bond length outliers are listed below:

Mol	Chain	Res	Type	Atoms	Z	Observed(Å)	Ideal(Å)
1	C	164	PRO	N-CD	-15.90	1.25	1.47

There are no bond angle outliers.

There are no chirality outliers.

All (1) planarity outliers are listed below:

Mol	Chain	Res	Type	Group
1	C	181	ARG	Sidechain

For Manuscript Review

5.2 Too-close contacts (i)

In the following table, the Non-H and H(model) columns list the number of non-hydrogen atoms and hydrogen atoms in the chain respectively. The H(added) column lists the number of hydrogen atoms added and optimized by MolProbity. The Clashes column lists the number of clashes within the asymmetric unit, whereas Symm-Clashes lists symmetry-related clashes.

Mol	Chain	Non-H	H(model)	H(added)	Clashes	Symm-Clashes
1	A	898	0	915	14	0
1	B	889	0	911	22	0
1	C	898	0	915	25	0
1	D	898	0	915	22	0
2	A	22	18	0	0	0
2	B	22	18	0	1	0
2	C	22	18	0	2	0
3	A	10	0	0	0	0
3	C	5	0	0	0	0
3	D	5	0	0	0	0
4	A	1	0	0	0	0
5	A	116	0	0	3	0
5	B	100	0	0	4	1
5	C	126	0	0	3	1
5	D	90	0	0	3	0
All	All	4102	54	3656	83	1

The all-atom clashscore is defined as the number of clashes found per 1000 atoms (including hydrogen atoms). The all-atom clashscore for this structure is 11.

All (83) close contacts within the same asymmetric unit are listed below, sorted by their clash magnitude.

Atom-1	Atom-2	Interatomic distance (Å)	Clash overlap (Å)
1:B:147:LYS:HE2	1:B:150:ARG:NH1	1.79	0.98
1:C:199:ASN:OD1	1:C:223:GLN:NE2	2.17	0.77
1:D:174:MET:HB2	1:D:200:LEU:HD12	1.68	0.75
1:C:126:MET:N	5:C:401:HOH:O	2.21	0.72
1:B:147:LYS:HE2	1:B:150:ARG:HH12	1.56	0.71
1:D:137:LEU:O	1:D:141:LYS:HG3	1.90	0.71
1:D:172:ARG:O	1:D:200:LEU:HD11	1.92	0.69
1:C:233:MET:SD	1:C:234:PRO:HD2	2.33	0.69
1:B:126:MET:HA	1:B:126:MET:CE	2.26	0.66
1:B:164:PRO:O	5:B:1001:HOH:O	2.12	0.66
1:A:199:ASN:OD1	1:A:223:GLN:NE2	2.28	0.66
1:C:135:HIS:CE1	1:C:232:ASN:HB3	2.30	0.65

Continued on next page...

For Manuscript Review

Continued from previous page...

Atom-1	Atom-2	Interatomic distance (Å)	Clash overlap (Å)
1:A:236:LYS:O	1:A:237:ASP:HB2	1.96	0.65
1:B:217:GLN:OE1	5:B:1002:HOH:O	2.15	0.64
1:D:129:PRO:HD2	1:D:132:GLN:OE1	1.96	0.64
1:B:182:LYS:HB3	1:B:188:TYR:CE2	2.32	0.64
1:B:147:LYS:CE	1:B:150:ARG:NH1	2.59	0.64
1:D:220:ARG:HD2	5:D:452:HOH:O	2.00	0.61
1:B:158:PRO:HG2	1:B:167:PHE:CE2	2.38	0.59
1:D:174:MET:HB2	1:D:200:LEU:CD1	2.33	0.57
1:D:198:PHE:CE1	1:D:223:GLN:HA	2.40	0.57
1:C:135:HIS:HE1	1:C:232:ASN:HB3	1.69	0.57
1:C:183:LEU:HD23	1:C:188:TYR:HD2	1.69	0.57
1:A:137:LEU:HD21	1:A:141:LYS:HZ1	1.70	0.56
1:D:128:ILE:HD11	1:D:133:GLN:HA	1.87	0.56
1:C:151:PRO:HB3	2:C:301:Y78:C05	2.36	0.55
1:C:137:LEU:HG	1:C:183:LEU:HD13	1.90	0.54
1:D:126:MET:N	5:D:406:HOH:O	2.40	0.54
1:D:199:ASN:O	1:D:203:GLU:HG2	2.08	0.54
1:C:136:ALA:CB	1:C:183:LEU:HD11	2.38	0.53
1:B:154:GLN:HB2	1:B:155:PRO:HD2	1.91	0.53
1:C:147:LYS:HB3	1:C:150:ARG:HH21	1.73	0.53
1:B:174:MET:O	1:B:201:MET:HG3	2.10	0.52
1:A:163:ILE:O	1:A:163:ILE:HG13	2.09	0.52
1:D:182:LYS:HE2	1:D:188:TYR:CZ	2.45	0.52
1:C:227:GLU:HG3	5:C:447:HOH:O	2.10	0.52
1:D:174:MET:CB	1:D:200:LEU:HD12	2.39	0.52
1:A:228:LYS:HA	1:A:231:LEU:HD12	1.92	0.51
1:A:136:ALA:O	1:A:140:ILE:HG12	2.11	0.51
1:D:161:LEU:O	1:D:164:PRO:HD2	2.11	0.51
1:C:232:ASN:HB2	5:C:455:HOH:O	2.10	0.51
1:A:135:HIS:CD2	1:A:233:MET:HA	2.46	0.50
1:B:188:TYR:HD1	1:B:193:GLN:HB3	1.75	0.50
1:A:186:ASN:HA	5:A:466:HOH:O	2.11	0.49
1:D:191:PRO:HA	1:D:194:ILE:HD12	1.95	0.49
1:C:161:LEU:HD13	2:C:301:Y78:C12	2.42	0.49
1:D:144:LYS:O	5:D:401:HOH:O	2.20	0.49
1:B:147:LYS:CE	1:B:150:ARG:CZ	2.91	0.48
1:C:140:ILE:O	1:C:144:LYS:HG3	2.14	0.47
1:A:149:ALA:HB3	5:A:407:HOH:O	2.13	0.47
1:A:126:MET:N	5:A:408:HOH:O	2.47	0.47
1:C:151:PRO:CD	1:C:218:MET:HE2	2.46	0.46
1:B:147:LYS:HE3	1:B:150:ARG:CZ	2.46	0.45

Continued on next page...

For Manuscript Review

Continued from previous page...

Atom-1	Atom-2	Interatomic distance (Å)	Clash overlap (Å)
1:B:151:PRO:HA	2:B:900:Y78:O01	2.16	0.45
1:D:231:LEU:HD23	1:D:231:LEU:HA	1.84	0.45
1:C:183:LEU:HD23	1:C:183:LEU:HA	1.77	0.45
1:A:183:LEU:HD23	1:A:183:LEU:HA	1.75	0.44
1:D:199:ASN:OD1	1:D:223:GLN:NE2	2.45	0.44
1:C:136:ALA:HB1	1:C:183:LEU:HD11	1.99	0.44
1:D:174:MET:CB	1:D:200:LEU:CD1	2.95	0.44
1:C:203:GLU:O	1:C:207:LYS:HG3	2.17	0.44
1:A:203:GLU:OE2	1:A:207:LYS:HD3	2.18	0.44
1:B:182:LYS:HG2	1:B:187:ALA:HB3	2.00	0.44
1:C:158:PRO:HG2	1:C:167:PHE:CE2	2.52	0.44
1:C:137:LEU:HG	1:C:183:LEU:CD1	2.48	0.43
1:C:147:LYS:HB3	1:C:150:ARG:NH2	2.34	0.43
1:B:127:GLY:HA2	1:B:189:GLU:OE1	2.18	0.43
1:A:233:MET:HE2	1:A:233:MET:HB2	1.81	0.43
1:C:156:VAL:O	1:C:158:PRO:HD3	2.18	0.43
1:B:165:LEU:HB2	5:B:1053:HOH:O	2.18	0.42
1:C:151:PRO:HD3	1:C:218:MET:HE2	2.01	0.42
1:D:236:LYS:HB2	1:D:236:LYS:HE3	1.77	0.42
1:A:192:GLU:OE1	1:A:192:GLU:N	2.51	0.42
1:B:131:HIS:CE1	1:B:236:LYS:HA	2.54	0.42
1:B:136:ALA:HB1	1:B:183:LEU:HD11	2.02	0.41
1:C:218:MET:O	1:C:222:ILE:HG12	2.20	0.41
1:B:214:VAL:O	1:B:218:MET:HG3	2.20	0.41
1:D:130:GLN:HE21	1:D:130:GLN:HB2	1.64	0.41
1:B:175:ASP:O	1:B:179:ILE:HG13	2.20	0.41
1:D:126:MET:HE3	1:D:126:MET:HB3	1.84	0.41
1:B:162:ASN:HB3	5:B:1061:HOH:O	2.21	0.41
1:C:136:ALA:HB3	1:C:183:LEU:HD11	2.02	0.40
1:D:202:VAL:HG21	1:D:223:GLN:HB2	2.02	0.40

All (1) symmetry-related close contacts are listed below. The label for Atom-2 includes the symmetry operator and encoded unit-cell translations to be applied.

Atom-1	Atom-2	Interatomic distance (Å)	Clash overlap (Å)
5:B:1019:HOH:O	5:C:410:HOH:O[5_555]	2.03	0.17

For Manuscript Review

5.3 Torsion angles (i)

5.3.1 Protein backbone (i)

In the following table, the Percentiles column shows the percent Ramachandran outliers of the chain as a percentile score with respect to all X-ray entries followed by that with respect to entries of similar resolution.

The Analysed column shows the number of residues for which the backbone conformation was analysed, and the total number of residues.

Mol	Chain	Analysed	Favoured	Allowed	Outliers	Percentiles	
1	A	110/114 (96%)	110 (100%)	0	0	100	100
1	B	109/114 (96%)	109 (100%)	0	0	100	100
1	C	110/114 (96%)	110 (100%)	0	0	100	100
1	D	110/114 (96%)	107 (97%)	3 (3%)	0	100	100
All	All	439/456 (96%)	436 (99%)	3 (1%)	0	100	100

There are no Ramachandran outliers to report.

5.3.2 Protein sidechains (i)

In the following table, the Percentiles column shows the percent sidechain outliers of the chain as a percentile score with respect to all X-ray entries followed by that with respect to entries of similar resolution.

The Analysed column shows the number of residues for which the sidechain conformation was analysed, and the total number of residues.

Mol	Chain	Analysed	Rotameric	Outliers	Percentiles	
1	A	97/97 (100%)	94 (97%)	3 (3%)	40	51
1	B	96/97 (99%)	95 (99%)	1 (1%)	76	86
1	C	97/97 (100%)	97 (100%)	0	100	100
1	D	97/97 (100%)	95 (98%)	2 (2%)	53	67
All	All	387/388 (100%)	381 (98%)	6 (2%)	62	76

All (6) residues with a non-rotameric sidechain are listed below:

Mol	Chain	Res	Type
1	A	126	MET
1	A	198	PHE

Continued on next page...

For Manuscript Review

Continued from previous page...

Mol	Chain	Res	Type
1	A	207	LYS
1	B	228	LYS
1	D	154	GLN
1	D	157	ASP

Sometimes sidechains can be flipped to improve hydrogen bonding and reduce clashes. All (6) such sidechains are listed below:

Mol	Chain	Res	Type
1	A	154	GLN
1	B	130	GLN
1	C	209	ASN
1	D	154	GLN
1	D	177	GLN
1	D	217	GLN

5.3.3 RNA (i)

There are no RNA molecules in this entry.

5.4 Non-standard residues in protein, DNA, RNA chains (i)

There are no non-standard protein/DNA/RNA residues in this entry.

5.5 Carbohydrates (i)

There are no monosaccharides in this entry.

5.6 Ligand geometry (i)

Of 8 ligands modelled in this entry, 1 is monoatomic - leaving 7 for Mogul analysis.

In the following table, the Counts columns list the number of bonds (or angles) for which Mogul statistics could be retrieved, the number of bonds (or angles) that are observed in the model and the number of bonds (or angles) that are defined in the Chemical Component Dictionary. The Link column lists molecule types, if any, to which the group is linked. The Z score for a bond length (or angle) is the number of standard deviations the observed value is removed from the expected value. A bond length (or angle) with $|Z| > 2$ is considered an outlier worth inspection. RMSZ is the root-mean-square of all Z scores of the bond lengths (or angles).

For Manuscript Review

Mol	Type	Chain	Res	Link	Bond lengths			Bond angles		
					Counts	RMSZ	#|Z| > 2	Counts	RMSZ	#|Z| > 2
3	PO4	D	301	-	4,4,4	0.84	0	6,6,6	0.52	0
2	Y78	B	900	-	21,24,24	3.43	8 (38%)	26,33,33	1.81	5 (19%)
3	PO4	A	303	-	4,4,4	0.86	0	6,6,6	0.44	0
3	PO4	A	302	-	4,4,4	0.89	0	6,6,6	0.44	0
2	Y78	A	301	-	21,24,24	3.37	8 (38%)	26,33,33	1.75	7 (26%)
2	Y78	C	301	-	21,24,24	3.45	8 (38%)	26,33,33	1.75	6 (23%)
3	PO4	C	302	-	4,4,4	0.88	0	6,6,6	0.46	0

In the following table, the Chirals column lists the number of chiral outliers, the number of chiral centers analysed, the number of these observed in the model and the number defined in the Chemical Component Dictionary. Similar counts are reported in the Torsion and Rings columns. '-' means no outliers of that kind were identified.

Mol	Type	Chain	Res	Link	Chirals	Torsions	Rings
2	Y78	B	900	-	-	0/7/9/9	0/3/3/3
2	Y78	C	301	-	-	0/7/9/9	0/3/3/3
2	Y78	A	301	-	-	0/7/9/9	0/3/3/3

All (24) bond length outliers are listed below:

Mol	Chain	Res	Type	Atoms	Z	Observed(Å)	Ideal(Å)
2	A	301	Y78	C13-C11	7.97	1.53	1.38
2	C	301	Y78	C13-C11	7.96	1.53	1.38
2	B	900	Y78	C13-C11	7.87	1.53	1.38
2	C	301	Y78	C03-N01	7.39	1.46	1.34
2	B	900	Y78	C03-N01	7.12	1.45	1.34
2	A	301	Y78	C03-N01	6.70	1.45	1.34
2	C	301	Y78	C12-C04	6.65	1.53	1.39
2	B	900	Y78	C12-C04	6.51	1.53	1.39
2	A	301	Y78	C12-C04	6.51	1.53	1.39
2	B	900	Y78	C14-C10	5.31	1.53	1.38
2	C	301	Y78	C14-C10	5.09	1.53	1.38
2	A	301	Y78	C14-C10	5.02	1.52	1.38
2	C	301	Y78	C16-C08	4.01	1.53	1.48
2	B	900	Y78	C16-C08	3.92	1.53	1.48
2	A	301	Y78	C16-C08	3.81	1.53	1.48
2	A	301	Y78	C14-C12	-3.77	1.31	1.38
2	B	900	Y78	C14-C12	-3.60	1.32	1.38
2	C	301	Y78	C14-C12	-3.58	1.32	1.38
2	A	301	Y78	C11-C04	-3.40	1.32	1.39
2	C	301	Y78	C11-C04	-3.37	1.32	1.39
2	B	900	Y78	C11-C04	-3.36	1.32	1.39

Continued on next page...

For Manuscript Review

Continued from previous page...

Mol	Chain	Res	Type	Atoms	Z	Observed(Å)	Ideal(Å)
2	B	900	Y78	C13-C10	-2.40	1.32	1.38
2	A	301	Y78	C13-C10	-2.33	1.32	1.38
2	C	301	Y78	C13-C10	-2.25	1.32	1.38

All (18) bond angle outliers are listed below:

Mol	Chain	Res	Type	Atoms	Z	Observed(^{o})	Ideal(^{o})
2	B	900	Y78	N02-C06-N03	-5.49	119.87	125.31
2	C	301	Y78	N02-C06-N03	-5.25	120.11	125.31
2	A	301	Y78	N02-C06-N03	-4.94	120.42	125.31
2	B	900	Y78	C07-N04-N03	-3.10	117.91	120.43
2	B	900	Y78	C01-C02-C05	-2.93	124.16	129.01
2	A	301	Y78	C04-C06-N03	2.69	121.71	117.46
2	C	301	Y78	C04-C06-N03	2.65	121.65	117.46
2	C	301	Y78	C03-N02-C06	2.64	120.85	116.10
2	C	301	Y78	C01-C02-C05	-2.57	124.74	129.01
2	A	301	Y78	C01-C02-C05	-2.53	124.81	129.01
2	A	301	Y78	C07-N04-N03	-2.50	118.40	120.43
2	A	301	Y78	C15-C07-N04	2.43	120.29	115.87
2	A	301	Y78	C03-N02-C06	2.32	120.27	116.10
2	B	900	Y78	C06-N03-N04	2.31	121.33	117.54
2	B	900	Y78	C04-C06-N02	2.18	121.00	117.33
2	A	301	Y78	C01-N01-C03	-2.13	120.33	123.14
2	C	301	Y78	C15-C07-N04	2.06	119.62	115.87
2	C	301	Y78	C01-N01-C03	-2.01	120.49	123.14

There are no chirality outliers.

There are no torsion outliers.

There are no ring outliers.

2 monomers are involved in 3 short contacts:

Mol	Chain	Res	Type	Clashes	Symm-Clashes
2	B	900	Y78	1	0
2	C	301	Y78	2	0

The following is a two-dimensional graphical depiction of Mogul quality analysis of bond lengths, bond angles, torsion angles, and ring geometry for all instances of the Ligand of Interest. In addition, ligands with molecular weight > 250 and outliers as shown on the validation Tables will also be included. For torsion angles, if less then 5% of the Mogul distribution of torsion angles is within 10 degrees of the torsion angle in question, then that torsion angle is considered an outlier. Any bond that is central to one or more torsion angles identified as an outlier by Mogul will be

For Manuscript Review

highlighted in the graph. For rings, the root-mean-square deviation (RMSD) between the ring in question and similar rings identified by Mogul is calculated over all ring torsion angles. If the average RMSD is greater than 60 degrees and the minimal RMSD between the ring in question and any Mogul-identified rings is also greater than 60 degrees, then that ring is considered an outlier. The outliers are highlighted in purple. The color gray indicates Mogul did not find sufficient equivalents in the CSD to analyse the geometry.

Ligand Y78 B 900

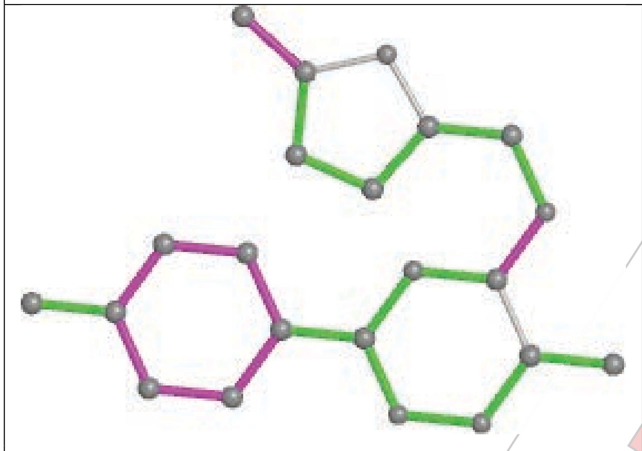
Bond lengths

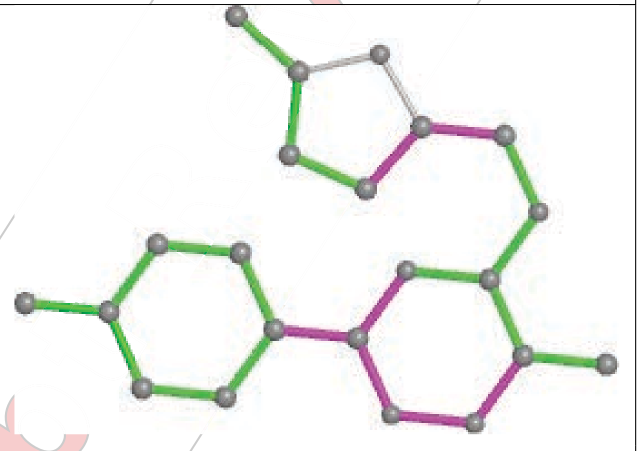
Bond angles

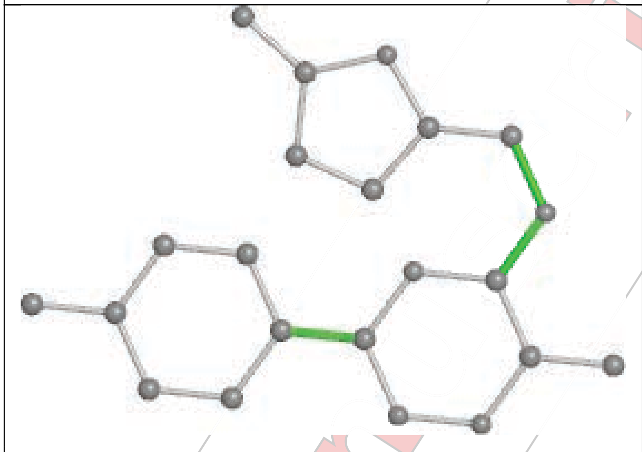
Torsions

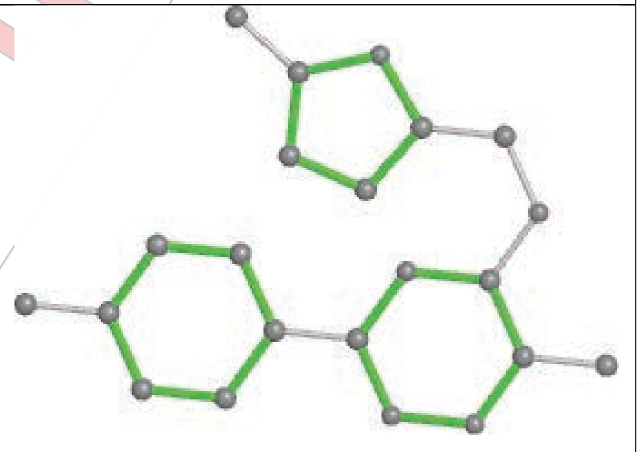
Rings

For Manuscript Review

Ligand Y78 A 301

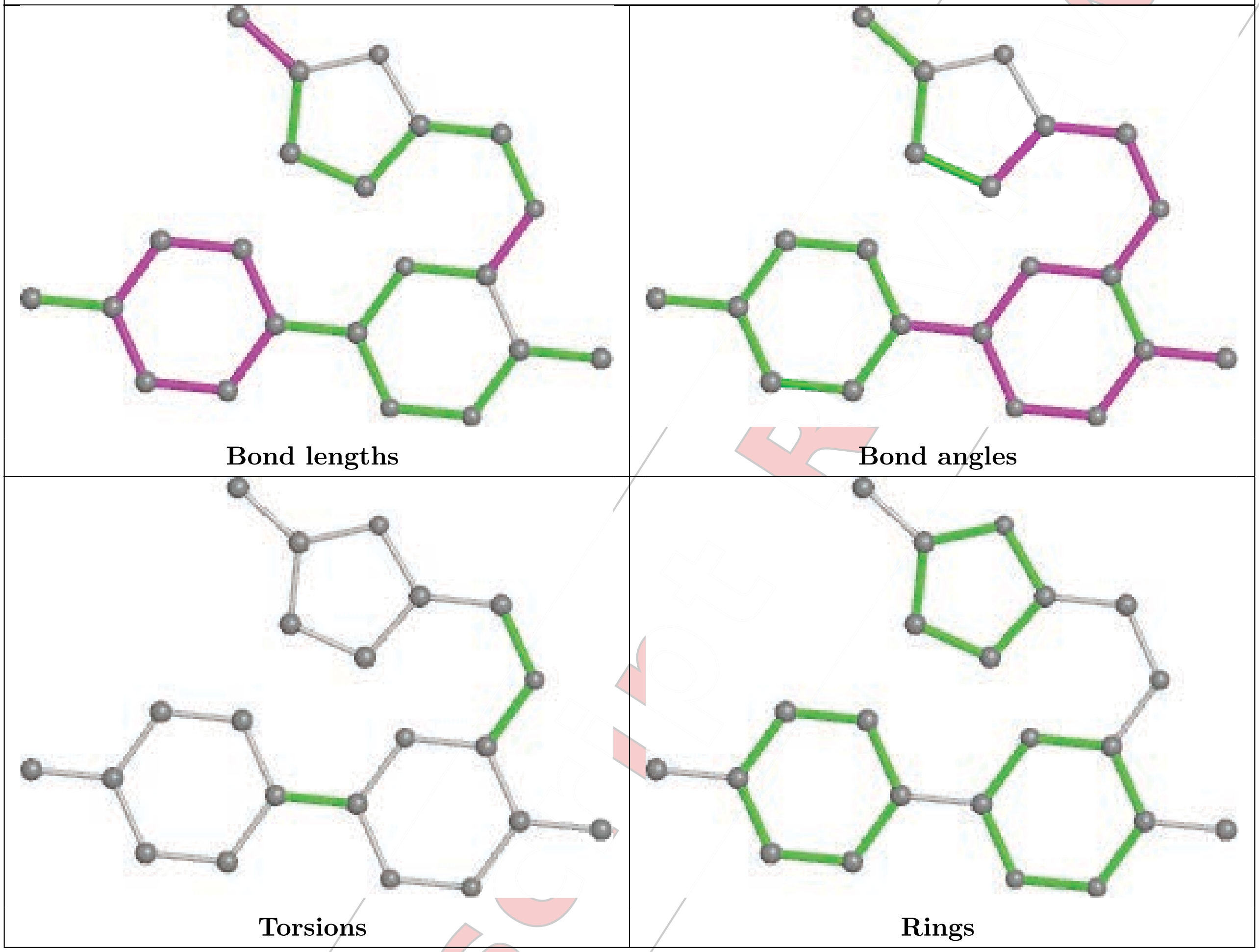

For Manuscript Review

Ligand Y78 C 301

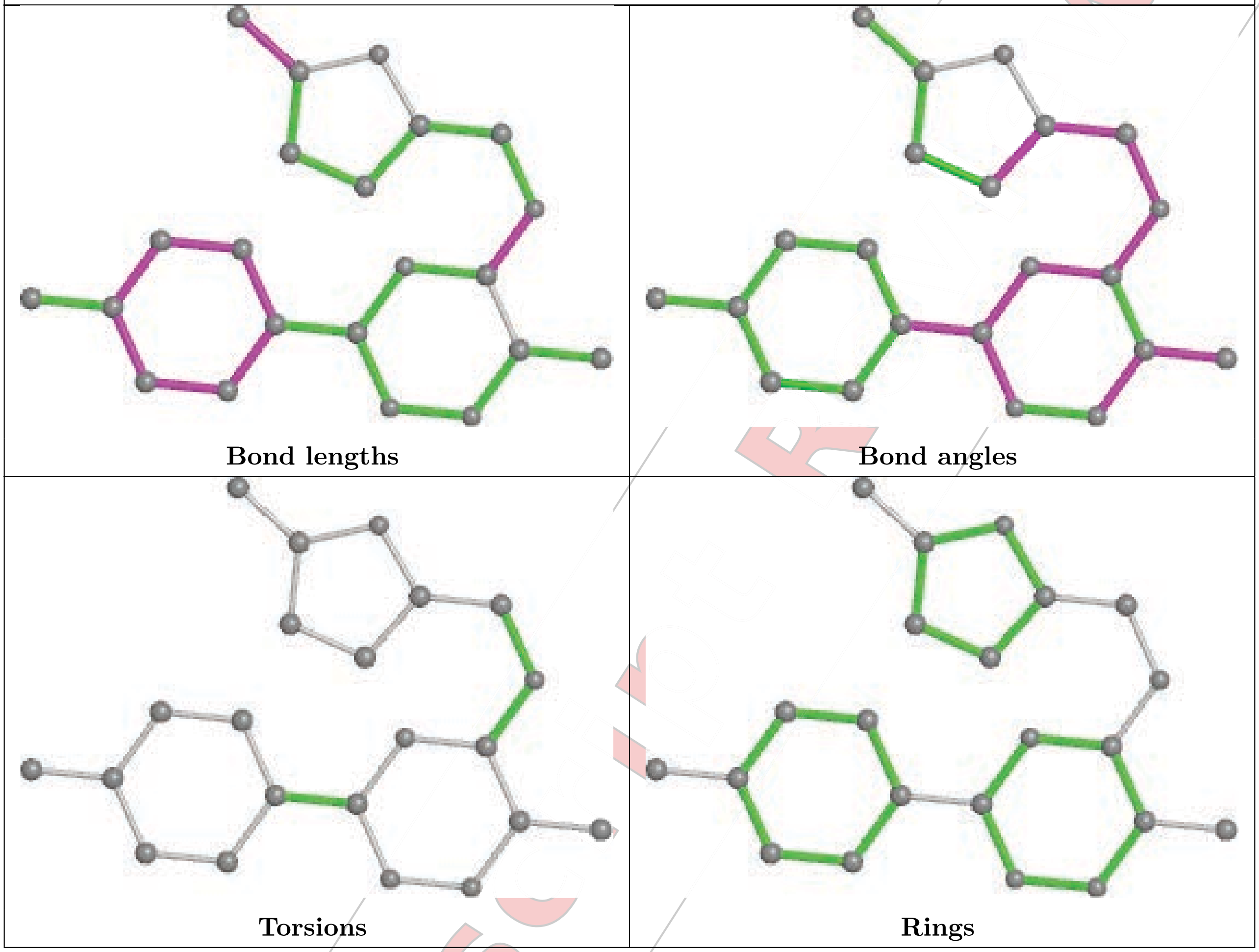

5.7 Other polymers (i)

There are no such residues in this entry.

5.8 Polymer linkage issues (i)

There are no chain breaks in this entry.

For Manuscript Review

6 Fit of model and data (i)

6.1 Protein, DNA and RNA chains (i)

In the following table, the column labelled '#RSRZ> 2' contains the number (and percentage) of RSRZ outliers, followed by percent RSRZ outliers for the chain as percentile scores relative to all X-ray entries and entries of similar resolution. The OWAB column contains the minimum, median, 95th percentile and maximum values of the occupancy-weighted average B-factor per residue. The column labelled 'Q< 0.9' lists the number of (and percentage) of residues with an average occupancy less than 0.9.

Mol	Chain	Analysed	<RSRZ>	#RSRZ>2			OWAB(Å^2)	Q<0.9
1	A	112/114 (98%)	-0.04	1 (0%)	84	83	16, 24, 44, 83	0
1	B	111/114 (97%)	-0.02	2 (1%)	68	66	19, 27, 41, 71	0
1	C	112/114 (98%)	-0.18	1 (0%)	84	83	15, 23, 34, 62	0
1	D	112/114 (98%)	-0.13	2 (1%)	68	66	16, 27, 43, 78	0
All	All	447/456 (98%)	-0.09	6 (1%)	77	75	15, 26, 43, 83	0

All (6) RSRZ outliers are listed below:

Mol	Chain	Res	Type	RSRZ
1	A	237	ASP	4.3
1	B	126	MET	3.4
1	D	126	MET	3.2
1	C	237	ASP	2.3
1	D	145	ARG	2.0
1	B	130	GLN	2.0

6.2 Non-standard residues in protein, DNA, RNA chains (i)

There are no non-standard protein/DNA/RNA residues in this entry.

6.3 Carbohydrates (i)

There are no monosaccharides in this entry.

6.4 Ligands (i)

In the following table, the Atoms column lists the number of modelled atoms in the group and the number defined in the chemical component dictionary. The B-factors column lists the minimum,

For Manuscript Review

median, 95th percentile and maximum values of B factors of atoms in the group. The column labelled 'Q< 0.9' lists the number of atoms with occupancy less than 0.9.

Mol	Type	Chain	Res	Atoms	RSCC	RSR	B-factors($Å^2$)	Q<0.9
2	Y78	C	301	22/22	0.82	0.39	67,83,105,105	0
3	PO4	C	302	5/5	0.82	0.19	71,76,86,91	0
3	PO4	A	303	5/5	0.87	0.11	69,70,74,78	0
2	Y78	A	301	22/22	0.94	0.12	13,17,22,23	0
3	PO4	A	302	5/5	0.94	0.15	46,49,51,52	0
4	K	A	304	1/1	0.94	0.11	45,45,45,45	0
2	Y78	B	900	22/22	0.95	0.14	22,33,51,58	0
3	PO4	D	301	5/5	0.97	0.22	46,50,53,56	0

The following is a graphical depiction of the model fit to experimental electron density of all instances of the Ligand of Interest. In addition, ligands with molecular weight > 250 and outliers as shown on the geometry validation Tables will also be included. Each fit is shown from different orientation to approximate a three-dimensional view.

Electron density around Y78 C 301:

$2mF_o$-DF_c (at 0.7 rmsd) in gray
mF_o-DF_c (at 3 rmsd) in purple (negative) and green (positive)

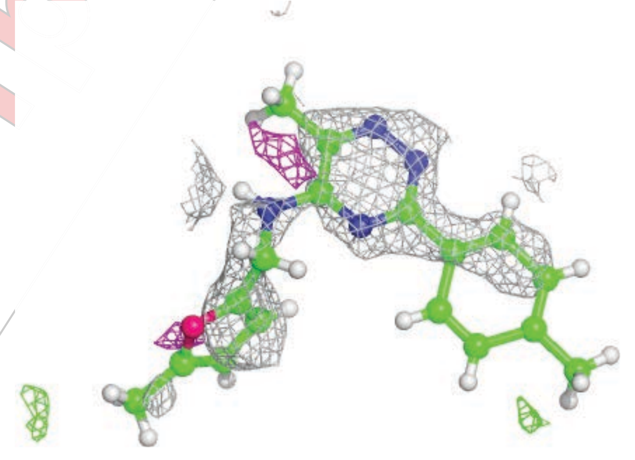

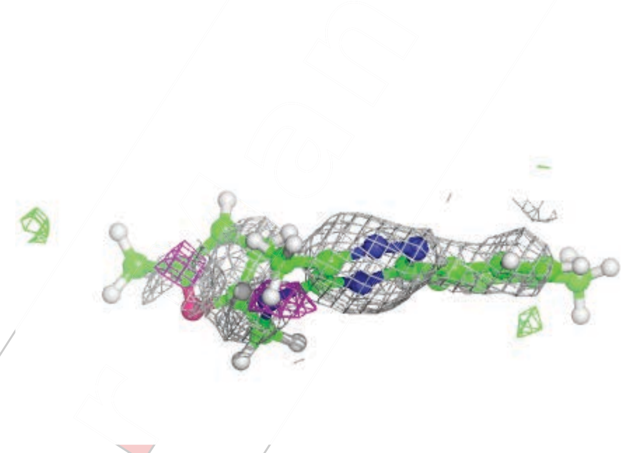

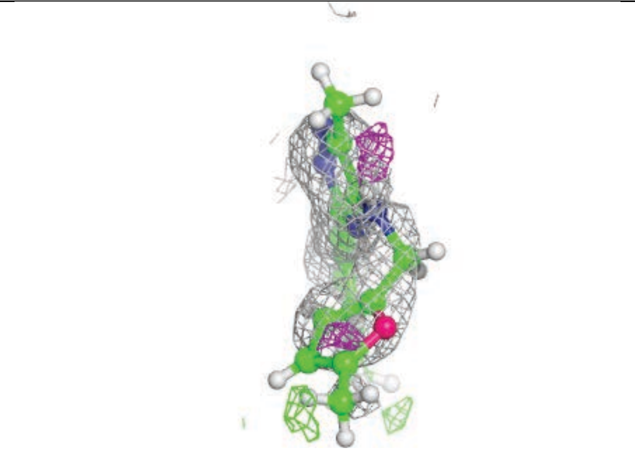

For Manuscript Review

Electron density around Y78 A 301:

2mF$_o$-DF$_c$ (at 0.7 rmsd) in gray
mF$_o$-DF$_c$ (at 3 rmsd) in purple (negative) and green (positive)

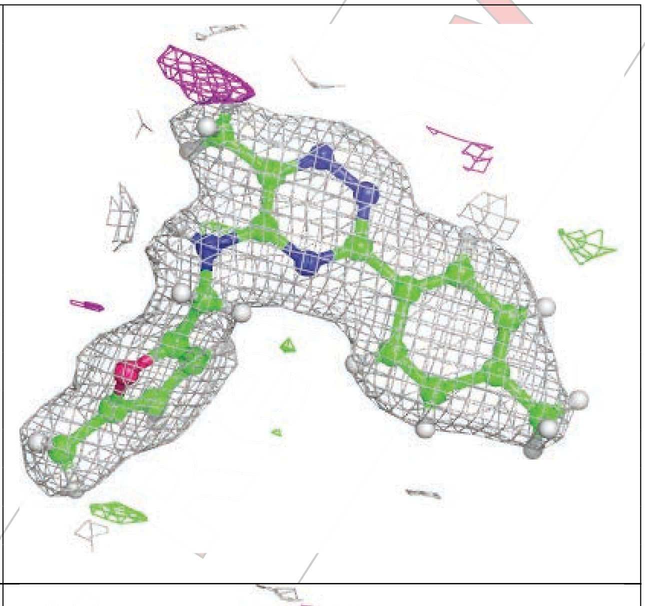

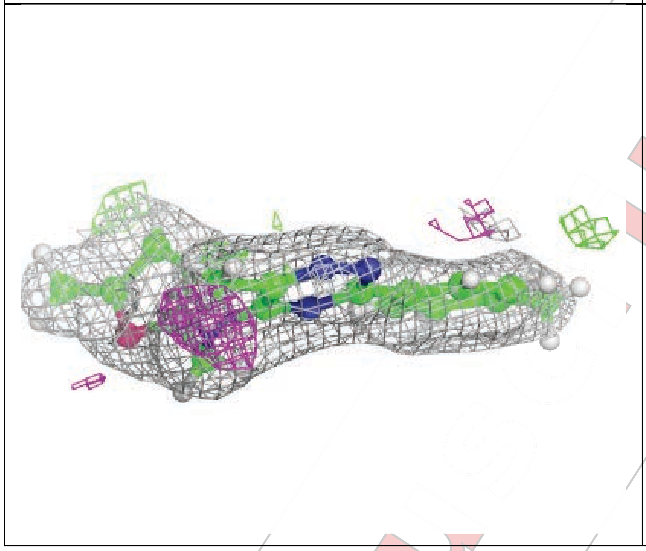

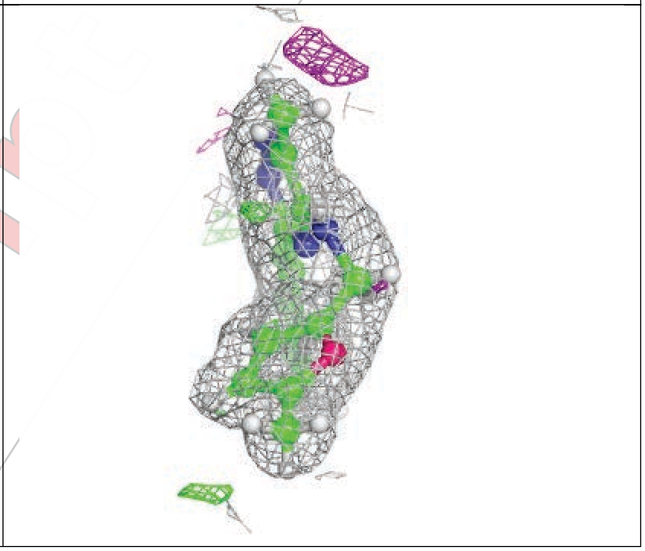

For Manuscript Review

Electron density around Y78 B 900:

$2mF_o$-DF_c (at 0.7 rmsd) in gray
mF_o-DF_c (at 3 rmsd) in purple (negative)
and green (positive)

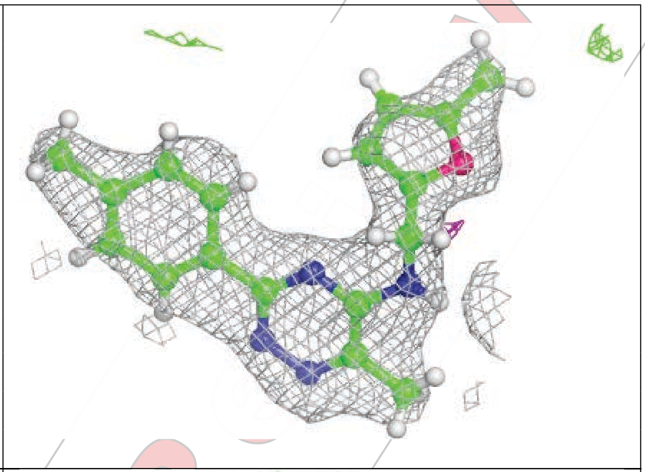

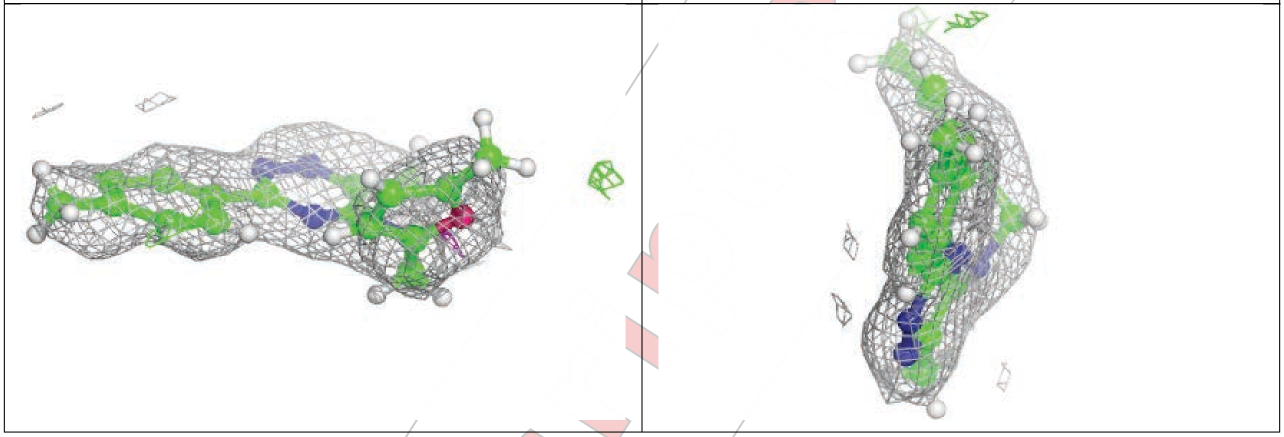

6.5 Other polymers (i)

There are no such residues in this entry.

For Manuscript Review

PDB validation report for CgBD2 in the unbound state (8R6L)

Full wwPDB X-ray Structure Validation Report (i)

Nov 23, 2023 – 02:31 pm GMT

PDB ID	:	8R6L
Title	:	Crystal structure of Candida glabrata Bdf1 bromodomain 2 in the unbound state
Deposited on	:	2023-11-22
Resolution	:	1.96 Å(reported)

This wwPDB validation report is for manuscript review

This is a Full wwPDB X-ray Structure Validation Report.

This report is produced by the wwPDB biocuration pipeline after annotation of the structure.

We welcome your comments at *validation@mail.wwpdb.org*
A user guide is available at
https://www.wwpdb.org/validation/2017/XrayValidationReportHelp
with specific help available everywhere you see the (i) symbol.

The types of validation reports are described at
http://www.wwpdb.org/validation/2017/FAQs#types.

The following versions of software and data (see references (i)) were used in the production of this report:

MolProbity	:	4.02b-467
Mogul	:	1.8.4, CSD as541be (2020)
Xtriage (Phenix)	:	1.13
EDS	:	2.36
Percentile statistics	:	20191225.v01 (using entries in the PDB archive December 25th 2019)
Refmac	:	5.8.0158
CCP4	:	7.0.044 (Gargrove)
Ideal geometry (proteins)	:	Engh & Huber (2001)
Ideal geometry (DNA, RNA)	:	Parkinson et al. (1996)

For Manuscript Review

1 Overall quality at a glance (i)

The following experimental techniques were used to determine the structure:
X-RAY DIFFRACTION

The reported resolution of this entry is 1.96 Å.

Percentile scores (ranging between 0-100) for global validation metrics of the entry are shown in the following graphic. The table shows the number of entries on which the scores are based.

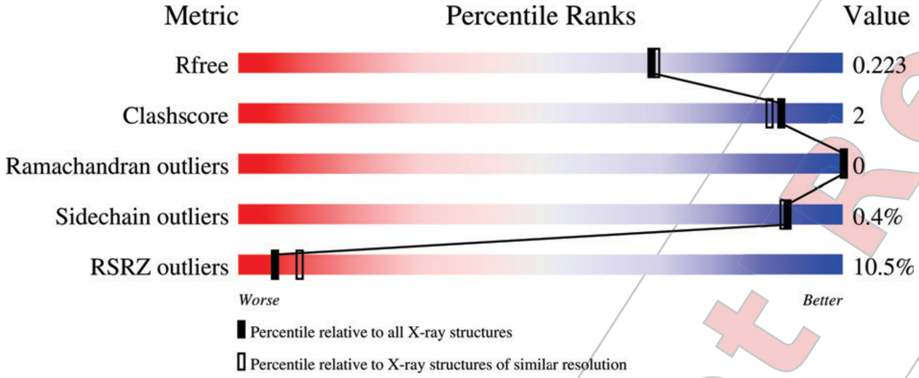

Metric	Whole archive (#Entries)	Similar resolution (#Entries, resolution range(Å))
R_{free}	130704	2580 (1.96-1.96)
Clashscore	141614	2705 (1.96-1.96)
Ramachandran outliers	138981	2678 (1.96-1.96)
Sidechain outliers	138945	2678 (1.96-1.96)
RSRZ outliers	127900	2539 (1.96-1.96)

The table below summarises the geometric issues observed across the polymeric chains and their fit to the electron density. The red, orange, yellow and green segments of the lower bar indicate the fraction of residues that contain outliers for >=3, 2, 1 and 0 types of geometric quality criteria respectively. A grey segment represents the fraction of residues that are not modelled. The numeric value for each fraction is indicated below the corresponding segment, with a dot representing fractions <=5% The upper red bar (where present) indicates the fraction of residues that have poor fit to the electron density. The numeric value is given above the bar.

Mol	Chain	Length	Quality of chain
1	A	118	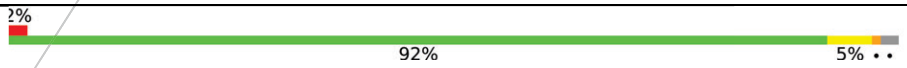
1	B	118	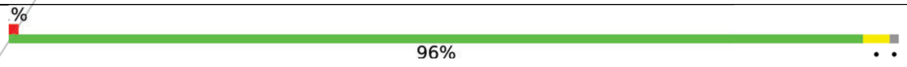
1	C	118	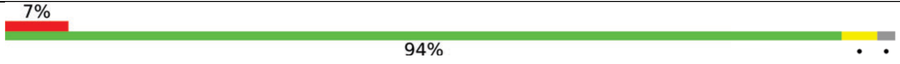
1	D	118	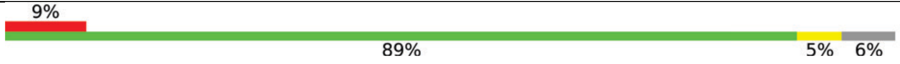

Continued on next page...

For Manuscript Review

Continued from previous page...

Mol	Chain	Length	Quality of chain
1	E	118	14% 93% • • •
1	F	118	16% 92% 6% •
1	G	118	24% 92% 6% •

For Manuscript Review

2 Entry composition (i)

There are 5 unique types of molecules in this entry. The entry contains 6973 atoms, of which 0 are hydrogens and 0 are deuteriums.

In the tables below, the ZeroOcc column contains the number of atoms modelled with zero occupancy, the AltConf column contains the number of residues with at least one atom in alternate conformation and the Trace column contains the number of residues modelled with at most 2 atoms.

- Molecule 1 is a protein called Candida glabrata strain CBS138 chromosome C complete sequence.

Mol	Chain	Residues	Atoms					ZeroOcc	AltConf	Trace
1	A	116	Total 950	C 609	N 156	O 176	S 9	0	0	0
1	B	117	Total 959	C 614	N 157	O 179	S 9	0	0	0
1	C	116	Total 950	C 609	N 156	O 176	S 9	0	0	0
1	D	111	Total 909	C 582	N 151	O 167	S 9	0	0	0
1	E	116	Total 950	C 609	N 156	O 176	S 9	0	0	0
1	F	116	Total 950	C 609	N 156	O 176	S 9	0	0	0
1	G	116	Total 950	C 609	N 156	O 176	S 9	0	0	0

There are 21 discrepancies between the modelled and reference sequences:

Chain	Residue	Modelled	Actual	Comment	Reference
A	301	ALA	-	expression tag	UNP Q6FWV7
A	302	MET	-	expression tag	UNP Q6FWV7
A	303	GLY	-	expression tag	UNP Q6FWV7
B	301	ALA	-	expression tag	UNP Q6FWV7
B	302	MET	-	expression tag	UNP Q6FWV7
B	303	GLY	-	expression tag	UNP Q6FWV7
C	301	ALA	-	expression tag	UNP Q6FWV7
C	302	MET	-	expression tag	UNP Q6FWV7
C	303	GLY	-	expression tag	UNP Q6FWV7
D	301	ALA	-	expression tag	UNP Q6FWV7
D	302	MET	-	expression tag	UNP Q6FWV7
D	303	GLY	-	expression tag	UNP Q6FWV7
E	301	ALA	-	expression tag	UNP Q6FWV7
E	302	MET	-	expression tag	UNP Q6FWV7

Continued on next page...

For Manuscript Review

Continued from previous page...

Chain	Residue	Modelled	Actual	Comment	Reference
E	303	GLY	-	expression tag	UNP Q6FWV7
F	301	ALA	-	expression tag	UNP Q6FWV7
F	302	MET	-	expression tag	UNP Q6FWV7
F	303	GLY	-	expression tag	UNP Q6FWV7
G	301	ALA	-	expression tag	UNP Q6FWV7
G	302	MET	-	expression tag	UNP Q6FWV7
G	303	GLY	-	expression tag	UNP Q6FWV7

- Molecule 2 is (4S)-2-METHYL-2,4-PENTANEDIOL (three-letter code: MPD) (formula: $C_6H_{14}O_2$).

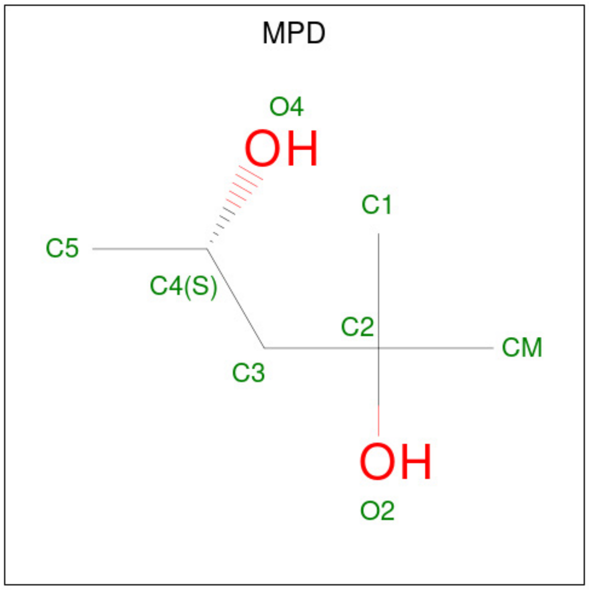

Mol	Chain	Residues	Atoms	ZeroOcc	AltConf
2	A	1	Total C O<br>8 6 2	0	0
2	A	1	Total C O<br>8 6 2	0	0
2	B	1	Total C O<br>8 6 2	0	0
2	B	1	Total C O<br>8 6 2	0	0
2	C	1	Total C O<br>8 6 2	0	0
2	D	1	Total C O<br>8 6 2	0	0
2	D	1	Total C O<br>8 6 2	0	0

Continued on next page...

For Manuscript Review

Continued from previous page...

Mol	Chain	Residues	Atoms	ZeroOcc	AltConf
2	E	1	Total C O<br>8 6 2	0	0

- Molecule 3 is GLYCEROL (three-letter code: GOL) (formula: $C_3H_8O_3$).

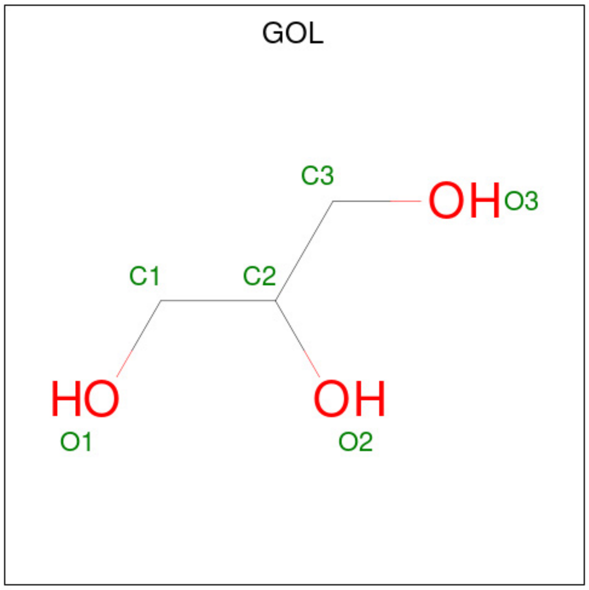

Mol	Chain	Residues	Atoms	ZeroOcc	AltConf
3	A	1	Total C O<br>6 3 3	0	0
3	A	1	Total C O<br>6 3 3	0	0
3	A	1	Total C O<br>6 3 3	0	0
3	A	1	Total C O<br>6 3 3	0	0
3	A	1	Total C O<br>6 3 3	0	0
3	B	1	Total C O<br>6 3 3	0	0
3	B	1	Total C O<br>6 3 3	0	0
3	B	1	Total C O<br>6 3 3	0	0
3	C	1	Total C O<br>6 3 3	0	0
3	C	1	Total C O<br>6 3 3	0	0

Continued on next page...

For Manuscript Review

Continued from previous page...

Mol	Chain	Residues	Atoms	ZeroOcc	AltConf
3	D	1	Total C O<br>6 3 3	0	0
3	E	1	Total C O<br>6 3 3	0	0
3	E	1	Total C O<br>6 3 3	0	0
3	E	1	Total C O<br>6 3 3	0	0

- Molecule 4 is ACETATE ION (three-letter code: ACT) (formula: $C_2H_3O_2$).

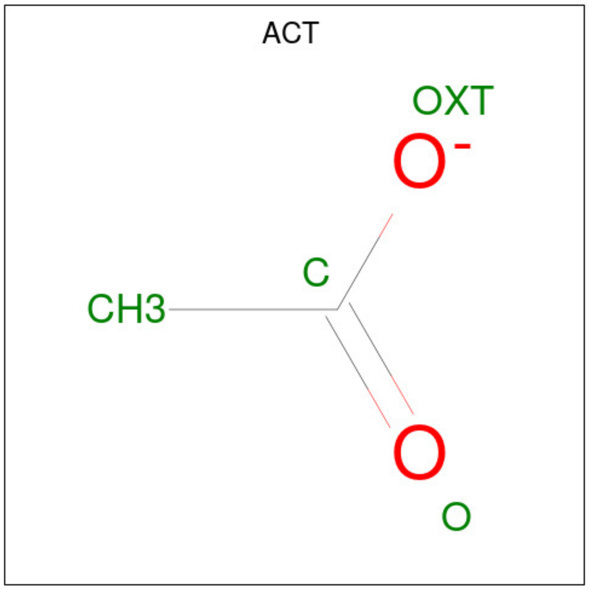

Mol	Chain	Residues	Atoms	ZeroOcc	AltConf
4	A	1	Total C O<br>4 2 2	0	0
4	A	1	Total C O<br>4 2 2	0	0
4	B	1	Total C O<br>4 2 2	0	0
4	B	1	Total C O<br>4 2 2	0	0
4	C	1	Total C O<br>4 2 2	0	0
4	E	1	Total C O<br>4 2 2	0	0
4	F	1	Total C O<br>4 2 2	0	0

For Manuscript Review

- Molecule 5 is water.

Mol	Chain	Residues	Atoms	ZeroOcc	AltConf
5	A	63	Total O<br>63 63	0	0
5	B	38	Total O<br>38 38	0	0
5	C	30	Total O<br>30 30	0	0
5	D	25	Total O<br>25 25	0	0
5	E	22	Total O<br>22 22	0	0
5	G	1	Total O<br>1 1	0	0

For Manuscript Review

3 Residue-property plots (i)

These plots are drawn for all protein, RNA, DNA and oligosaccharide chains in the entry. The first graphic for a chain summarises the proportions of the various outlier classes displayed in the second graphic. The second graphic shows the sequence view annotated by issues in geometry and electron density. Residues are color-coded according to the number of geometric quality criteria for which they contain at least one outlier: green = 0, yellow = 1, orange = 2 and red = 3 or more. A red dot above a residue indicates a poor fit to the electron density (RSRZ > 2). Stretches of 2 or more consecutive residues without any outlier are shown as a green connector. Residues present in the sample, but not in the model, are shown in grey.

- Molecule 1: Candida glabrata strain CBS138 chromosome C complete sequence

Chain A:

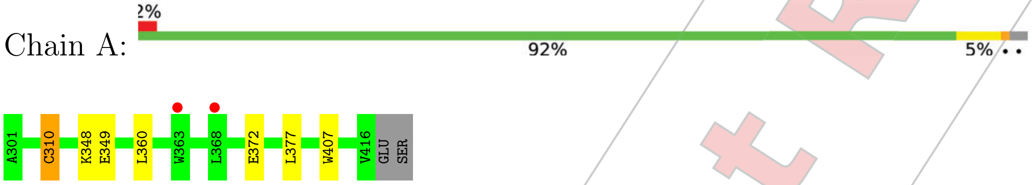

- Molecule 1: Candida glabrata strain CBS138 chromosome C complete sequence

Chain B:

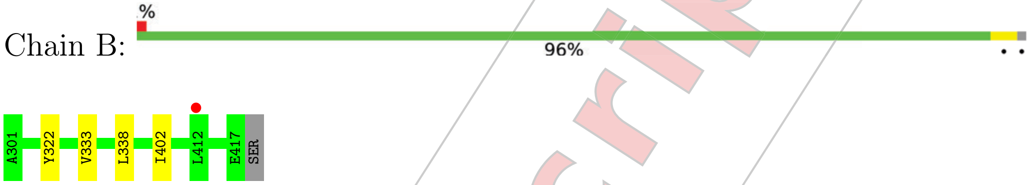

- Molecule 1: Candida glabrata strain CBS138 chromosome C complete sequence

Chain C:

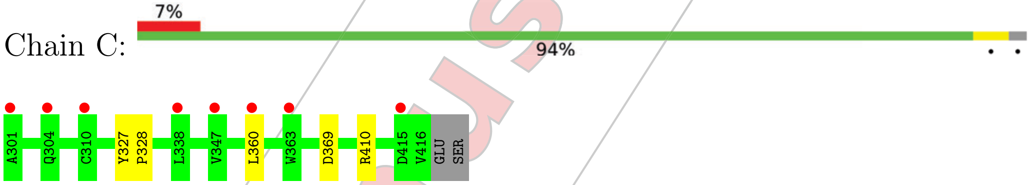

- Molecule 1: Candida glabrata strain CBS138 chromosome C complete sequence

Chain D:

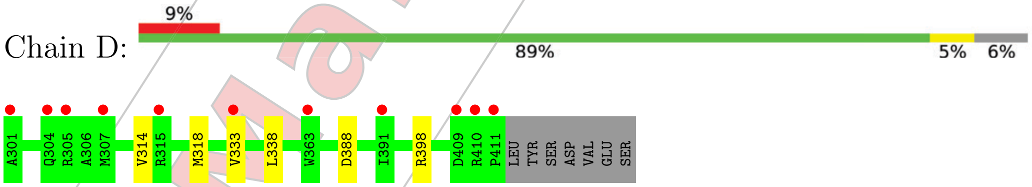

- Molecule 1: Candida glabrata strain CBS138 chromosome C complete sequence

Chain E:

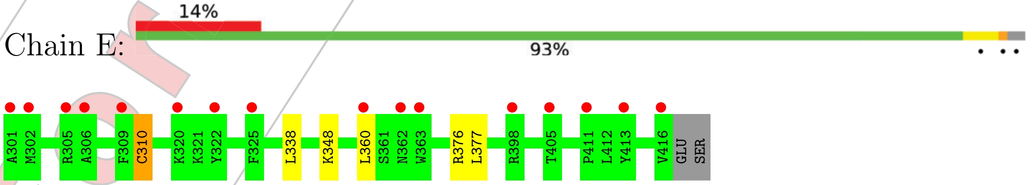

- Molecule 1: Candida glabrata strain CBS138 chromosome C complete sequence

For Manuscript Review

Chain F:

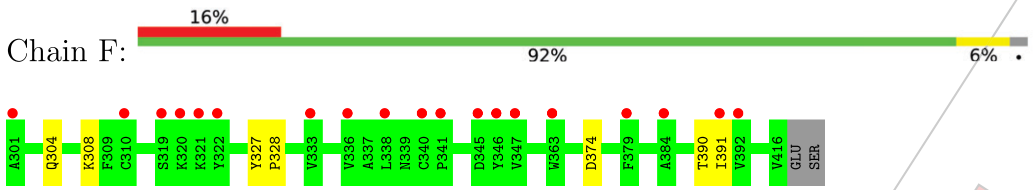

• Molecule 1: Candida glabrata strain CBS138 chromosome C complete sequence

Chain G:

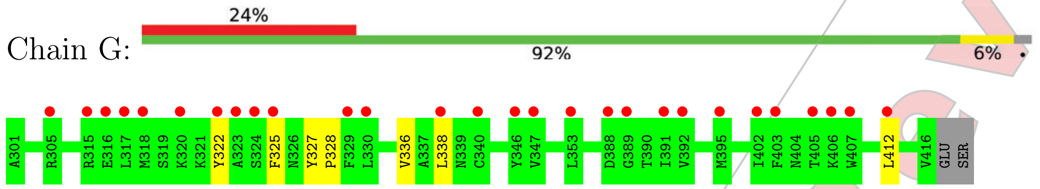

For Manuscript Review

4 Data and refinement statistics (i)

Property	Value	Source
Space group	P 1 21 1	Depositor
Cell constants<br>a, b, c, α, β, γ	80.29Å 67.69Å 96.62Å<br>90.00° 95.52° 90.00°	Depositor
Resolution (Å)	44.37 – 1.96<br>44.37 – 1.73	Depositor<br>EDS
% Data completeness<br>(in resolution range)	98.9 (44.37-1.96)<br>83.1 (44.37-1.73)	Depositor<br>EDS
R_{merge}	(Not available)	Depositor
R_{sym}	(Not available)	Depositor
$< I/\sigma(I) >$ [1]	0.68 (at 1.73Å)	Xtriage
Refinement program	PHENIX 1.20.1_4487	Depositor
R, R_{free}	0.200 , 0.227<br>0.195 , 0.223	Depositor<br>DCC
R_{free} test set	4347 reflections (4.89%)	wwPDB-VP
Wilson B-factor (Å^2)	40.5	Xtriage
Anisotropy	0.392	Xtriage
Bulk solvent k_{sol}(e/Å^3), B_{sol}(Å^2)	0.33 , 58.4	EDS
L-test for twinning[2]	$< \|L\| >$ = 0.50, $< L^2 >$ = 0.33	Xtriage
Estimated twinning fraction	No twinning to report.	Xtriage
F_o,F_c correlation	0.97	EDS
Total number of atoms	6973	wwPDB-VP
Average B, all atoms (Å^2)	76.0	wwPDB-VP

Xtriage's analysis on translational NCS is as follows: *The largest off-origin peak in the Patterson function is 5.08% of the height of the origin peak. No significant pseudotranslation is detected.*

[1] Intensities estimated from amplitudes.
[2] Theoretical values of $< |L| >$, $< L^2 >$ for acentric reflections are 0.5, 0.333 respectively for untwinned datasets, and 0.375, 0.2 for perfectly twinned datasets.

For Manuscript Review

5 Model quality (i)

5.1 Standard geometry (i)

Bond lengths and bond angles in the following residue types are not validated in this section: GOL, MPD, ACT

The Z score for a bond length (or angle) is the number of standard deviations the observed value is removed from the expected value. A bond length (or angle) with $|Z| > 5$ is considered an outlier worth inspection. RMSZ is the root-mean-square of all Z scores of the bond lengths (or angles).

Mol	Chain	Bond lengths		Bond angles	
		RMSZ	#|Z| >5	RMSZ	#|Z| >5
1	A	0.80	1/976 (0.1%)	0.69	0/1322
1	B	0.64	0/985	0.65	0/1334
1	C	0.46	0/976	0.56	1/1322 (0.1%)
1	D	0.52	0/934	0.56	0/1264
1	E	0.39	0/976	0.51	0/1322
1	F	0.30	0/976	0.44	0/1322
1	G	0.31	0/976	0.47	0/1322
All	All	0.52	1/6799 (0.0%)	0.56	1/9208 (0.0%)

All (1) bond length outliers are listed below:

Mol	Chain	Res	Type	Atoms	Z	Observed(Å)	Ideal(Å)
1	A	310	CYS	CB-SG	-5.25	1.73	1.81

All (1) bond angle outliers are listed below:

Mol	Chain	Res	Type	Atoms	Z	Observed(^{o})	Ideal(^{o})
1	C	360	LEU	CA-CB-CG	5.21	127.29	115.30

There are no chirality outliers.

There are no planarity outliers.

5.2 Too-close contacts (i)

In the following table, the Non-H and H(model) columns list the number of non-hydrogen atoms and hydrogen atoms in the chain respectively. The H(added) column lists the number of hydrogen atoms added and optimized by MolProbity. The Clashes column lists the number of clashes within the asymmetric unit, whereas Symm-Clashes lists symmetry-related clashes.

For Manuscript Review

Mol	Chain	Non-H	H(model)	H(added)	Clashes	Symm-Clashes
1	A	950	0	909	4	0
1	B	959	0	915	2	0
1	C	950	0	909	2	0
1	D	909	0	871	4	0
1	E	950	0	909	5	0
1	F	950	0	909	4	0
1	G	950	0	909	5	0
2	A	16	0	28	0	0
2	B	16	0	28	0	0
2	C	8	0	14	0	0
2	D	16	0	28	2	0
2	E	8	0	14	1	0
3	A	30	0	39	0	0
3	B	18	0	23	0	0
3	C	12	0	16	0	0
3	D	6	0	8	0	0
3	E	18	0	24	1	0
4	A	8	0	6	1	0
4	B	8	0	6	0	0
4	C	4	0	3	0	0
4	E	4	0	3	0	0
4	F	4	0	3	0	0
5	A	63	0	0	0	0
5	B	38	0	0	0	0
5	C	30	0	0	0	0
5	D	25	0	0	0	0
5	E	22	0	0	0	0
5	G	1	0	0	1	0
All	All	6973	0	6574	24	0

The all-atom clashscore is defined as the number of clashes found per 1000 atoms (including hydrogen atoms). The all-atom clashscore for this structure is 2.

All (24) close contacts within the same asymmetric unit are listed below, sorted by their clash magnitude.

Atom-1	Atom-2	Interatomic distance (Å)	Clash overlap (Å)
1:D:398:ARG:HH11	2:D:902:MPD:H31	1.43	0.81
1:D:398:ARG:NH1	2:D:902:MPD:H31	2.14	0.61
1:G:412:LEU:O	5:G:501:HOH:O	2.18	0.56
1:E:310:CYS:HB3	1:E:360:LEU:HD21	1.91	0.51
1:A:348:LYS:NZ	4:A:904:ACT:OXT	2.39	0.49
1:E:338:LEU:HD13	2:E:901:MPD:HM2	1.95	0.48

Continued on next page...

For Manuscript Review

Continued from previous page...

Atom-1	Atom-2	Interatomic distance (Å)	Clash overlap (Å)
1:E:377:LEU:HD11	3:E:903:GOL:H12	1.96	0.47
1:A:310:CYS:HB3	1:A:360:LEU:HD21	1.96	0.46
1:B:333:VAL:HG13	1:B:338:LEU:HD12	1.98	0.45
1:G:322:TYR:HB3	1:G:325:PHE:CZ	2.53	0.44
1:A:349:GLU:HG2	1:A:377:LEU:HD21	1.99	0.44
1:C:369:ASP:OD1	1:C:410:ARG:NH1	2.52	0.43
1:F:390:THR:HG22	1:G:336:VAL:O	2.19	0.43
1:C:327:TYR:CG	1:C:328:PRO:HD3	2.53	0.43
1:G:327:TYR:CG	1:G:328:PRO:HD3	2.54	0.43
1:D:333:VAL:HG13	1:D:338:LEU:HD12	2.00	0.42
1:F:304:GLN:OE1	1:F:308:LYS:HE3	2.19	0.42
1:B:322:TYR:CE1	1:B:402:ILE:HD13	2.55	0.42
1:A:372:GLU:HB2	1:A:407:TRP:CZ2	2.54	0.41
1:E:376:ARG:HD3	1:E:376:ARG:HA	1.90	0.41
1:F:327:TYR:CG	1:F:328:PRO:HD3	2.55	0.41
1:E:348:LYS:HA	1:E:348:LYS:HD3	1.93	0.40
1:D:314:VAL:O	1:D:318:MET:HG3	2.22	0.40
1:F:391:ILE:HD12	1:G:338:LEU:HD23	2.02	0.40

There are no symmetry-related clashes.

5.3 Torsion angles (i)

5.3.1 Protein backbone (i)

In the following table, the Percentiles column shows the percent Ramachandran outliers of the chain as a percentile score with respect to all X-ray entries followed by that with respect to entries of similar resolution.

The Analysed column shows the number of residues for which the backbone conformation was analysed, and the total number of residues.

Mol	Chain	Analysed	Favoured	Allowed	Outliers	Percentiles	
1	A	114/118 (97%)	113 (99%)	1 (1%)	0	100	100
1	B	115/118 (98%)	113 (98%)	2 (2%)	0	100	100
1	C	114/118 (97%)	112 (98%)	2 (2%)	0	100	100
1	D	109/118 (92%)	108 (99%)	1 (1%)	0	100	100
1	E	114/118 (97%)	112 (98%)	2 (2%)	0	100	100
1	F	114/118 (97%)	113 (99%)	1 (1%)	0	100	100

Continued on next page...

For Manuscript Review

Continued from previous page...

Mol	Chain	Analysed	Favoured	Allowed	Outliers	Percentiles	
1	G	114/118 (97%)	113 (99%)	1 (1%)	0	100	100
All	All	794/826 (96%)	784 (99%)	10 (1%)	0	100	100

There are no Ramachandran outliers to report.

5.3.2 Protein sidechains ⓘ

In the following table, the Percentiles column shows the percent sidechain outliers of the chain as a percentile score with respect to all X-ray entries followed by that with respect to entries of similar resolution.

The Analysed column shows the number of residues for which the sidechain conformation was analysed, and the total number of residues.

Mol	Chain	Analysed	Rotameric	Outliers	Percentiles	
1	A	104/106 (98%)	104 (100%)	0	100	100
1	B	105/106 (99%)	105 (100%)	0	100	100
1	C	104/106 (98%)	104 (100%)	0	100	100
1	D	99/106 (93%)	98 (99%)	1 (1%)	76	74
1	E	104/106 (98%)	103 (99%)	1 (1%)	76	74
1	F	104/106 (98%)	103 (99%)	1 (1%)	76	74
1	G	104/106 (98%)	104 (100%)	0	100	100
All	All	724/742 (98%)	721 (100%)	3 (0%)	91	90

All (3) residues with a non-rotameric sidechain are listed below:

Mol	Chain	Res	Type
1	D	388	ASP
1	E	310	CYS
1	F	374	ASP

Sometimes sidechains can be flipped to improve hydrogen bonding and reduce clashes. All (1) such sidechains are listed below:

Mol	Chain	Res	Type
1	D	380	GLN

For Manuscript Review

5.3.3 RNA (i)

There are no RNA molecules in this entry.

5.4 Non-standard residues in protein, DNA, RNA chains (i)

There are no non-standard protein/DNA/RNA residues in this entry.

5.5 Carbohydrates (i)

There are no monosaccharides in this entry.

5.6 Ligand geometry (i)

29 ligands are modelled in this entry.

In the following table, the Counts columns list the number of bonds (or angles) for which Mogul statistics could be retrieved, the number of bonds (or angles) that are observed in the model and the number of bonds (or angles) that are defined in the Chemical Component Dictionary. The Link column lists molecule types, if any, to which the group is linked. The Z score for a bond length (or angle) is the number of standard deviations the observed value is removed from the expected value. A bond length (or angle) with $|Z| > 2$ is considered an outlier worth inspection. RMSZ is the root-mean-square of all Z scores of the bond lengths (or angles).

Mol	Type	Chain	Res	Link	Bond lengths			Bond angles		
					Counts	RMSZ	$\#\|Z\| > 2$	Counts	RMSZ	$\#\|Z\| > 2$
2	MPD	A	902	-	7,7,7	0.26	0	9,10,10	0.68	0
3	GOL	B	506	-	5,5,5	0.90	0	5,5,5	1.06	0
4	ACT	B	504	-	3,3,3	1.34	0	3,3,3	1.39	0
3	GOL	E	904	-	5,5,5	0.97	0	5,5,5	0.92	0
3	GOL	E	902	-	5,5,5	1.00	0	5,5,5	0.89	0
3	GOL	A	903	-	5,5,5	1.01	0	5,5,5	0.94	0
2	MPD	D	902	-	7,7,7	0.35	0	9,10,10	0.62	0
4	ACT	A	907	-	3,3,3	1.18	0	3,3,3	1.22	0
3	GOL	A	908	-	5,5,5	1.00	0	5,5,5	0.98	0
4	ACT	B	507	-	3,3,3	1.52	1 (33%)	3,3,3	1.44	1 (33%)
3	GOL	A	905	-	5,5,5	1.14	1 (20%)	5,5,5	1.11	0
2	MPD	A	901	-	7,7,7	0.40	0	9,10,10	0.34	0
3	GOL	D	903	-	5,5,5	0.88	0	5,5,5	1.07	0
3	GOL	A	909	-	5,5,5	1.15	1 (20%)	5,5,5	0.76	0
2	MPD	E	901	-	7,7,7	0.38	0	9,10,10	0.39	0
2	MPD	D	901	-	7,7,7	0.30	0	9,10,10	0.32	0
3	GOL	A	906	-	5,5,5	1.27	1 (20%)	5,5,5	1.22	1 (20%)

For Manuscript Review

Mol	Type	Chain	Res	Link	Bond lengths			Bond angles		
					Counts	RMSZ	#|Z| > 2	Counts	RMSZ	#|Z| > 2
4	ACT	C	904	-	3,3,3	1.31	0	3,3,3	1.31	0
4	ACT	E	905	-	3,3,3	1.16	0	3,3,3	1.41	0
2	MPD	C	901	-	7,7,7	0.29	0	9,10,10	0.24	0
3	GOL	B	505	-	5,5,5	1.00	0	5,5,5	0.94	0
4	ACT	F	501	-	3,3,3	1.28	0	3,3,3	1.52	1 (33%)
3	GOL	C	902	-	5,5,5	1.10	0	5,5,5	0.84	0
3	GOL	E	903	-	5,5,5	1.16	1 (20%)	5,5,5	1.16	0
3	GOL	B	502	-	5,5,5	1.42	1 (20%)	5,5,5	0.79	0
4	ACT	A	904	-	3,3,3	2.10	1 (33%)	3,3,3	1.40	0
3	GOL	C	903	-	5,5,5	0.94	0	5,5,5	1.13	0
2	MPD	B	501	-	7,7,7	0.48	0	9,10,10	1.00	0
2	MPD	B	503	-	7,7,7	0.29	0	9,10,10	0.32	0

In the following table, the Chirals column lists the number of chiral outliers, the number of chiral centers analysed, the number of these observed in the model and the number defined in the Chemical Component Dictionary. Similar counts are reported in the Torsion and Rings columns. '-' means no outliers of that kind were identified.

Mol	Type	Chain	Res	Link	Chirals	Torsions	Rings
2	MPD	A	902	-	-	1/5/5/5	-
3	GOL	B	506	-	-	0/4/4/4	-
3	GOL	E	904	-	-	2/4/4/4	-
3	GOL	E	902	-	-	0/4/4/4	-
3	GOL	A	903	-	-	2/4/4/4	-
2	MPD	D	902	-	-	3/5/5/5	-
3	GOL	A	908	-	-	2/4/4/4	-
3	GOL	A	905	-	-	3/4/4/4	-
2	MPD	A	901	-	-	1/5/5/5	-
3	GOL	D	903	-	-	4/4/4/4	-
3	GOL	A	909	-	-	0/4/4/4	-
2	MPD	E	901	-	-	1/5/5/5	-
2	MPD	D	901	-	-	0/5/5/5	-
3	GOL	A	906	-	-	0/4/4/4	-
2	MPD	C	901	-	-	0/5/5/5	-
3	GOL	B	505	-	-	0/4/4/4	-
3	GOL	C	902	-	-	4/4/4/4	-
3	GOL	E	903	-	-	3/4/4/4	-
3	GOL	B	502	-	-	2/4/4/4	-
3	GOL	C	903	-	-	2/4/4/4	-

Continued on next page...

For Manuscript Review

Continued from previous page...

Mol	Type	Chain	Res	Link	Chirals	Torsions	Rings
2	MPD	B	501	-	-	0/5/5/5	-
2	MPD	B	503	-	-	3/5/5/5	-

All (7) bond length outliers are listed below:

Mol	Chain	Res	Type	Atoms	Z	Observed(Å)	Ideal(Å)
4	A	904	ACT	CH3-C	3.34	1.63	1.49
3	B	502	GOL	O2-C2	-2.67	1.35	1.43
3	A	906	GOL	C1-C2	2.44	1.61	1.51
3	A	909	GOL	O2-C2	-2.20	1.36	1.43
3	A	905	GOL	C3-C2	2.15	1.60	1.51
4	B	507	ACT	CH3-C	2.14	1.58	1.49
3	E	903	GOL	C3-C2	2.09	1.60	1.51

All (3) bond angle outliers are listed below:

Mol	Chain	Res	Type	Atoms	Z	Observed(°)	Ideal(°)
3	A	906	GOL	C3-C2-C1	-2.35	102.56	111.70
4	F	501	ACT	OXT-C-O	2.04	129.58	122.05
4	B	507	ACT	OXT-C-O	2.01	129.46	122.05

There are no chirality outliers.

All (33) torsion outliers are listed below:

Mol	Chain	Res	Type	Atoms
3	C	902	GOL	C1-C2-C3-O3
3	D	903	GOL	C1-C2-C3-O3
3	E	904	GOL	O1-C1-C2-C3
3	E	904	GOL	O1-C1-C2-O2
3	A	903	GOL	O1-C1-C2-C3
3	A	905	GOL	O1-C1-C2-C3
3	A	908	GOL	C1-C2-C3-O3
3	B	502	GOL	O1-C1-C2-C3
3	C	902	GOL	O1-C1-C2-C3
3	D	903	GOL	O1-C1-C2-C3
3	E	903	GOL	O1-C1-C2-C3
3	C	902	GOL	O1-C1-C2-O2
3	D	903	GOL	O2-C2-C3-O3
3	D	903	GOL	O1-C1-C2-O2
2	D	902	MPD	O2-C2-C3-C4
3	A	903	GOL	O1-C1-C2-O2

Continued on next page...

For Manuscript Review

Continued from previous page...

Mol	Chain	Res	Type	Atoms
3	A	905	GOL	O2-C2-C3-O3
3	A	908	GOL	O1-C1-C2-O2
3	C	903	GOL	O2-C2-C3-O3
3	E	903	GOL	O1-C1-C2-O2
2	B	503	MPD	C1-C2-C3-C4
3	C	902	GOL	O2-C2-C3-O3
3	B	502	GOL	C1-C2-C3-O3
3	E	903	GOL	O2-C2-C3-O3
3	C	903	GOL	C1-C2-C3-O3
2	A	902	MPD	C2-C3-C4-C5
2	B	503	MPD	C2-C3-C4-C5
2	D	902	MPD	C2-C3-C4-C5
3	A	905	GOL	O1-C1-C2-O2
2	A	901	MPD	C2-C3-C4-O4
2	B	503	MPD	C2-C3-C4-O4
2	D	902	MPD	C2-C3-C4-O4
2	E	901	MPD	C2-C3-C4-O4

There are no ring outliers.

4 monomers are involved in 5 short contacts:

Mol	Chain	Res	Type	Clashes	Symm-Clashes
2	D	902	MPD	2	0
2	E	901	MPD	1	0
3	E	903	GOL	1	0
4	A	904	ACT	1	0

5.7 Other polymers (i)

There are no such residues in this entry.

5.8 Polymer linkage issues (i)

There are no chain breaks in this entry.

For Manuscript Review

6 Fit of model and data (i)

6.1 Protein, DNA and RNA chains (i)

In the following table, the column labelled '#RSRZ> 2' contains the number (and percentage) of RSRZ outliers, followed by percent RSRZ outliers for the chain as percentile scores relative to all X-ray entries and entries of similar resolution. The OWAB column contains the minimum, median, 95th percentile and maximum values of the occupancy-weighted average B-factor per residue. The column labelled 'Q< 0.9' lists the number of (and percentage) of residues with an average occupancy less than 0.9.

Mol	Chain	Analysed	<RSRZ>	#RSRZ>2			OWAB(Å^2)	Q<0.9
1	A	116/118 (98%)	0.46	2 (1%)	70	77	32, 44, 68, 73	0
1	B	117/118 (99%)	0.31	1 (0%)	84	89	33, 53, 82, 93	0
1	C	116/118 (98%)	0.62	8 (6%)	16	25	35, 70, 105, 120	0
1	D	111/118 (94%)	0.81	11 (9%)	7	12	44, 75, 120, 133	0
1	E	116/118 (98%)	0.77	16 (13%)	2	4	45, 85, 127, 137	0
1	F	116/118 (98%)	0.91	19 (16%)	1	2	76, 97, 115, 126	0
1	G	116/118 (98%)	1.28	28 (24%)	0	0	78, 102, 120, 127	0
All	All	808/826 (97%)	0.74	85 (10%)	6	10	32, 78, 118, 137	0

All (85) RSRZ outliers are listed below:

Mol	Chain	Res	Type	RSRZ
1	F	301	ALA	7.3
1	G	325	PHE	5.7
1	F	347	VAL	4.7
1	G	338	LEU	4.7
1	G	346	TYR	4.5
1	F	340	CYS	4.4
1	G	402	ILE	4.4
1	G	392	VAL	4.4
1	E	411	PRO	4.3
1	F	338	LEU	4.2
1	G	406	LYS	4.0
1	E	309	PHE	4.0
1	G	347	VAL	3.9
1	G	388	ASP	3.7
1	E	302	MET	3.6
1	F	336	VAL	3.6
1	E	363	TRP	3.6

Continued on next page...

For Manuscript Review

Continued from previous page...

Mol	Chain	Res	Type	RSRZ
1	G	305	ARG	3.6
1	D	410	ARG	3.5
1	D	304	GLN	3.4
1	F	392	VAL	3.4
1	G	395	MET	3.4
1	F	346	TYR	3.4
1	G	318	MET	3.4
1	C	363	TRP	3.3
1	D	307	MET	3.3
1	G	322	TYR	3.3
1	E	413	TYR	3.2
1	C	301	ALA	3.1
1	G	403	PHE	3.1
1	G	407	TRP	3.1
1	C	360	LEU	3.0
1	G	324	SER	3.0
1	E	325	PHE	3.0
1	D	301	ALA	2.9
1	E	305	ARG	2.9
1	F	310	CYS	2.9
1	C	304	GLN	2.9
1	F	333	VAL	2.8
1	C	310	CYS	2.8
1	E	301	ALA	2.8
1	E	306	ALA	2.8
1	C	415	ASP	2.8
1	F	321	LYS	2.8
1	D	411	PRO	2.7
1	D	315	ARG	2.7
1	E	362	ASN	2.7
1	G	405	THR	2.7
1	F	320	LYS	2.6
1	G	412	LEU	2.6
1	G	389	GLY	2.6
1	E	405	THR	2.6
1	G	323	ALA	2.5
1	D	363	TRP	2.5
1	G	317	LEU	2.5
1	D	305	ARG	2.5
1	F	345	ASP	2.4
1	G	391	ILE	2.4
1	B	412	LEU	2.4

Continued on next page...

For Manuscript Review

Continued from previous page...

Mol	Chain	Res	Type	RSRZ
1	D	333	VAL	2.4
1	G	329	PHE	2.4
1	F	384	ALA	2.3
1	G	340	CYS	2.3
1	A	368	LEU	2.3
1	E	320	LYS	2.3
1	G	316	GLU	2.3
1	F	322	TYR	2.2
1	E	360	LEU	2.2
1	G	353	LEU	2.2
1	A	363	TRP	2.2
1	D	391	ILE	2.2
1	E	398	ARG	2.1
1	E	322	TYR	2.1
1	F	379	PHE	2.1
1	F	391	ILE	2.1
1	G	320	LYS	2.1
1	F	319	SER	2.1
1	D	409	ASP	2.1
1	G	315	ARG	2.1
1	G	330	LEU	2.1
1	C	347	VAL	2.0
1	E	416	VAL	2.0
1	C	338	LEU	2.0
1	F	341	PRO	2.0
1	F	363	TRP	2.0

6.2 Non-standard residues in protein, DNA, RNA chains (i)

There are no non-standard protein/DNA/RNA residues in this entry.

6.3 Carbohydrates (i)

There are no monosaccharides in this entry.

6.4 Ligands (i)

In the following table, the Atoms column lists the number of modelled atoms in the group and the number defined in the chemical component dictionary. The B-factors column lists the minimum, median, 95th percentile and maximum values of B factors of atoms in the group. The column labelled 'Q< 0.9' lists the number of atoms with occupancy less than 0.9.

For Manuscript Review

Mol	Type	Chain	Res	Atoms	RSCC	RSR	B-factors(Å^2)	Q<0.9
3	GOL	B	506	6/6	0.63	0.23	72,73,74,75	0
4	ACT	A	904	4/4	0.71	0.23	62,66,68,69	0
3	GOL	A	908	6/6	0.72	0.18	85,88,88,89	0
3	GOL	A	903	6/6	0.79	0.24	92,94,96,98	0
3	GOL	E	902	6/6	0.80	0.17	97,98,98,99	0
3	GOL	E	904	6/6	0.81	0.23	98,99,99,99	0
4	ACT	B	504	4/4	0.82	0.14	87,89,89,89	0
2	MPD	B	503	8/8	0.83	0.17	85,87,90,91	0
3	GOL	C	902	6/6	0.83	0.27	77,78,79,80	0
3	GOL	A	905	6/6	0.84	0.17	51,55,55,55	0
3	GOL	A	909	6/6	0.84	0.28	72,78,79,80	0
3	GOL	B	502	6/6	0.84	0.20	67,75,77,81	0
4	ACT	C	904	4/4	0.84	0.20	93,93,94,94	0
2	MPD	E	901	8/8	0.86	0.20	69,77,81,83	0
3	GOL	A	906	6/6	0.86	0.13	51,52,54,54	0
3	GOL	E	903	6/6	0.87	0.22	49,53,57,57	0
4	ACT	B	507	4/4	0.88	0.11	71,77,77,79	0
3	GOL	C	903	6/6	0.89	0.14	43,53,55,55	0
2	MPD	A	902	8/8	0.89	0.18	84,87,90,90	0
4	ACT	F	501	4/4	0.90	0.14	83,85,86,88	0
3	GOL	D	903	6/6	0.91	0.12	52,55,57,59	0
3	GOL	B	505	6/6	0.92	0.14	74,80,83,86	0
4	ACT	A	907	4/4	0.92	0.22	46,55,55,62	0
2	MPD	A	901	8/8	0.93	0.14	48,56,63,66	0
2	MPD	D	902	8/8	0.94	0.25	92,94,95,96	0
4	ACT	E	905	4/4	0.94	0.13	100,101,102,102	0
2	MPD	D	901	8/8	0.94	0.20	64,71,74,76	0
2	MPD	C	901	8/8	0.95	0.19	61,66,69,72	0
2	MPD	B	501	8/8	0.97	0.13	38,45,48,50	0

6.5 Other polymers (i)

There are no such residues in this entry.

For Manuscript Review

PDB validation report for CgBD2 bound to I-BET151 (8R6M)

Full wwPDB X-ray Structure Validation Report (i)

Nov 23, 2023 – 08:29 pm GMT

PDB ID	:	8R6M
Title	:	Crystal structure of Candida glabrata Bdf1 bromodomain 2 bound to I-BET151
Deposited on	:	2023-11-22
Resolution	:	1.95 Å(reported)

This wwPDB validation report is for manuscript review

This is a Full wwPDB X-ray Structure Validation Report.

This report is produced by the wwPDB biocuration pipeline after annotation of the structure.

We welcome your comments at *validation@mail.wwpdb.org*
A user guide is available at
https://www.wwpdb.org/validation/2017/XrayValidationReportHelp
with specific help available everywhere you see the (i) symbol.

The types of validation reports are described at
http://www.wwpdb.org/validation/2017/FAQs#types.

For Manuscript Review

The following versions of software and data (see references (i)) were used in the production of this report:

MolProbity	:	4.02b-467
Mogul	:	1.8.4, CSD as541be (2020)
Xtriage (Phenix)	:	1.13
EDS	:	2.36
buster-report	:	1.1.7 (2018)
Percentile statistics	:	20191225.v01 (using entries in the PDB archive December 25th 2019)
Refmac	:	5.8.0158
CCP4	:	7.0.044 (Gargrove)
Ideal geometry (proteins)	:	Engh & Huber (2001)

1 Overall quality at a glance (i)

The following experimental techniques were used to determine the structure:
X-RAY DIFFRACTION

The reported resolution of this entry is 1.95 Å.

Percentile scores (ranging between 0-100) for global validation metrics of the entry are shown in the following graphic. The table shows the number of entries on which the scores are based.

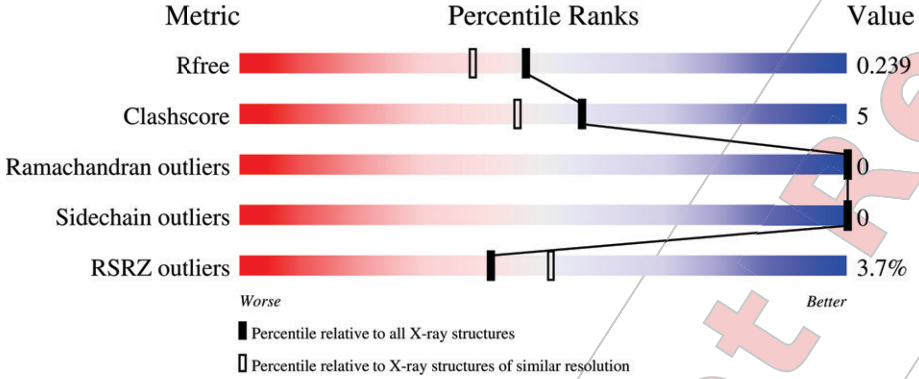

Metric	Whole archive (#Entries)	Similar resolution (#Entries, resolution range(Å))
R_{free}	130704	2580 (1.96-1.96)
Clashscore	141614	2705 (1.96-1.96)
Ramachandran outliers	138981	2678 (1.96-1.96)
Sidechain outliers	138945	2678 (1.96-1.96)
RSRZ outliers	127900	2539 (1.96-1.96)

The table below summarises the geometric issues observed across the polymeric chains and their fit to the electron density. The red, orange, yellow and green segments of the lower bar indicate the fraction of residues that contain outliers for >=3, 2, 1 and 0 types of geometric quality criteria respectively. A grey segment represents the fraction of residues that are not modelled. The numeric value for each fraction is indicated below the corresponding segment, with a dot representing fractions <=5% The upper red bar (where present) indicates the fraction of residues that have poor fit to the electron density. The numeric value is given above the bar.

Mol	Chain	Length	Quality of chain
1	A	118	% / 92% / 7% / •
1	B	118	2% / 86% / 14% / •
1	C	118	3% / 82% / 14% / •

Continued on next page...

Ideal geometry (DNA, RNA) : Parkinson et al. (1996)
Validation Pipeline (wwPDB-VP) : 2.36

For Manuscript Review

Continued from previous page...

Mol	Chain	Length	Quality of chain
1	D	118	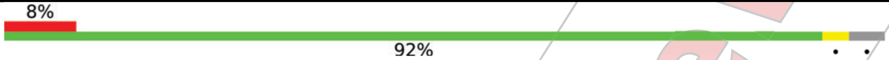

For Manuscript Review

2 Entry composition (i)

There are 5 unique types of molecules in this entry. The entry contains 4257 atoms, of which 0 are hydrogens and 0 are deuteriums.

In the tables below, the ZeroOcc column contains the number of atoms modelled with zero occupancy, the AltConf column contains the number of residues with at least one atom in alternate conformation and the Trace column contains the number of residues modelled with at most 2 atoms.

- Molecule 1 is a protein called Candida glabrata strain CBS138 chromosome C complete sequence.

Mol	Chain	Residues	Atoms					ZeroOcc	AltConf	Trace
1	A	116	Total 947	C 606	N 156	O 176	S 9	0	0	0
1	B	117	Total 955	C 612	N 157	O 177	S 9	0	0	0
1	C	113	Total 929	C 597	N 153	O 170	S 9	0	0	0
1	D	113	Total 922	C 593	N 150	O 170	S 9	0	0	0

There are 12 discrepancies between the modelled and reference sequences:

Chain	Residue	Modelled	Actual	Comment	Reference
A	301	ALA	-	expression tag	UNP Q6FWV7
A	302	MET	-	expression tag	UNP Q6FWV7
A	303	GLY	-	expression tag	UNP Q6FWV7
B	301	ALA	-	expression tag	UNP Q6FWV7
B	302	MET	-	expression tag	UNP Q6FWV7
B	303	GLY	-	expression tag	UNP Q6FWV7
C	301	ALA	-	expression tag	UNP Q6FWV7
C	302	MET	-	expression tag	UNP Q6FWV7
C	303	GLY	-	expression tag	UNP Q6FWV7
D	301	ALA	-	expression tag	UNP Q6FWV7
D	302	MET	-	expression tag	UNP Q6FWV7
D	303	GLY	-	expression tag	UNP Q6FWV7

- Molecule 2 is GLYCEROL (three-letter code: GOL) (formula: $C_3H_8O_3$).

For Manuscript Review

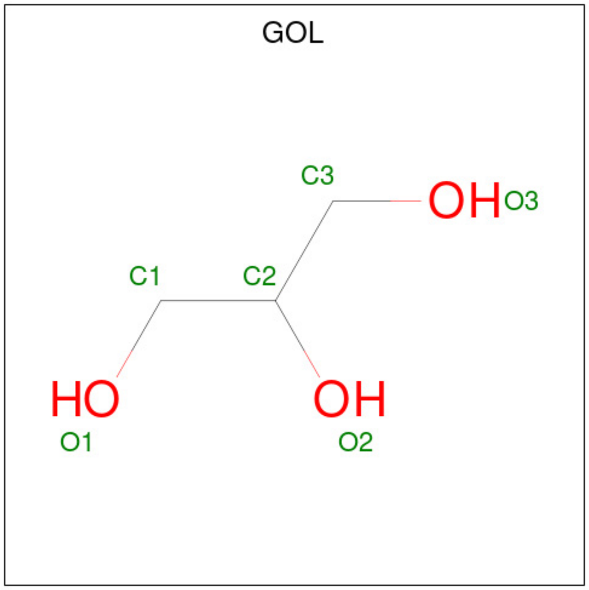

Mol	Chain	Residues	Atoms	ZeroOcc	AltConf
2	A	1	Total C O<br>6 3 3	0	0
2	A	1	Total C O<br>6 3 3	0	0
2	A	1	Total C O<br>6 3 3	0	0
2	A	1	Total C O<br>6 3 3	0	0
2	A	1	Total C O<br>6 3 3	0	0
2	A	1	Total C O<br>6 3 3	0	0
2	A	1	Total C O<br>6 3 3	0	0
2	A	1	Total C O<br>6 3 3	0	0
2	B	1	Total C O<br>6 3 3	0	0
2	B	1	Total C O<br>6 3 3	0	0
2	B	1	Total C O<br>6 3 3	0	0
2	B	1	Total C O<br>6 3 3	0	0
2	B	1	Total C O<br>6 3 3	0	0
2	B	1	Total C O<br>6 3 3	0	0

Continued on next page...

For Manuscript Review

Continued from previous page...

Mol	Chain	Residues	Atoms	ZeroOcc	AltConf
2	B	1	Total C O<br>6 3 3	0	0
2	C	1	Total C O<br>6 3 3	0	0
2	C	1	Total C O<br>6 3 3	0	0
2	C	1	Total C O<br>6 3 3	0	0
2	C	1	Total C O<br>6 3 3	0	0
2	C	1	Total C O<br>6 3 3	0	0
2	D	1	Total C O<br>6 3 3	0	0
2	D	1	Total C O<br>6 3 3	0	0
2	D	1	Total C O<br>6 3 3	0	0
2	D	1	Total C O<br>6 3 3	0	0

- Molecule 3 is SULFATE ION (three-letter code: SO4) (formula: O_4S).

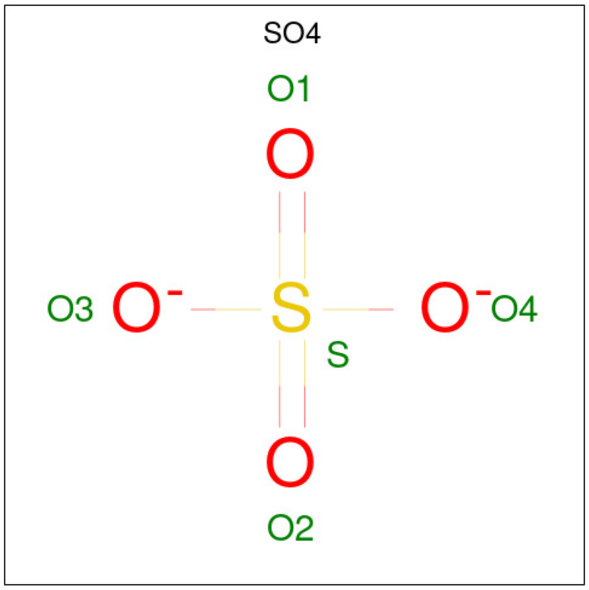

Mol	Chain	Residues	Atoms	ZeroOcc	AltConf
3	A	1	Total O S<br>5 4 1	0	0

Continued on next page...

For Manuscript Review

Continued from previous page...

Mol	Chain	Residues	Atoms	ZeroOcc	AltConf
3	A	1	Total O S<br>5 4 1	0	0

- Molecule 4 is 7-(3,5-DIMETHYL-1,2-OXAZOL-4-YL)-8-METHOXY-1-[(1R)-1-(PYRIDIN-2-YL)ETHYL]-1H,2H,3H-IMIDAZO[4,5-C]QUINOLIN-2-ONE (three-letter code: 1GH) (formula: $C_{23}H_{21}N_5O_3$) (labeled as "Ligand of Interest" by depositor).

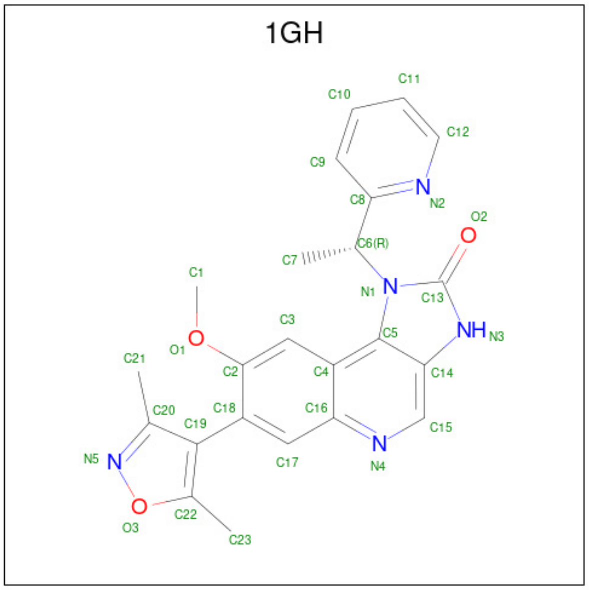

Mol	Chain	Residues	Atoms	ZeroOcc	AltConf
4	A	1	Total C N O<br>31 23 5 3	0	0
4	B	1	Total C N O<br>31 23 5 3	0	0
4	C	1	Total C N O<br>31 23 5 3	0	0
4	D	1	Total C N O<br>31 23 5 3	0	0

- Molecule 5 is water.

Mol	Chain	Residues	Atoms	ZeroOcc	AltConf
5	A	78	Total O<br>78 78	0	0
5	B	64	Total O<br>64 64	0	0
5	C	49	Total O<br>49 49	0	0

Continued on next page...

For Manuscript Review

Continued from previous page...

Mol	Chain	Residues	Atoms	ZeroOcc	AltConf
5	D	35	Total O<br>35 35	0	0

For Manuscript Review

3 Residue-property plots (i)

These plots are drawn for all protein, RNA, DNA and oligosaccharide chains in the entry. The first graphic for a chain summarises the proportions of the various outlier classes displayed in the second graphic. The second graphic shows the sequence view annotated by issues in geometry and electron density. Residues are color-coded according to the number of geometric quality criteria for which they contain at least one outlier: green = 0, yellow = 1, orange = 2 and red = 3 or more. A red dot above a residue indicates a poor fit to the electron density (RSRZ > 2). Stretches of 2 or more consecutive residues without any outlier are shown as a green connector. Residues present in the sample, but not in the model, are shown in grey.

- Molecule 1: Candida glabrata strain CBS138 chromosome C complete sequence

Chain A:
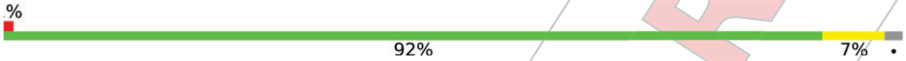

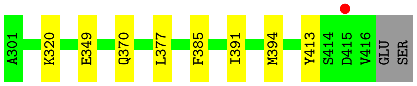

- Molecule 1: Candida glabrata strain CBS138 chromosome C complete sequence

Chain B:
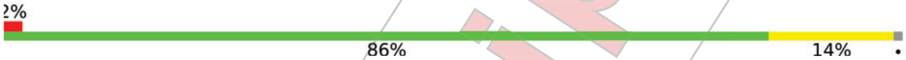

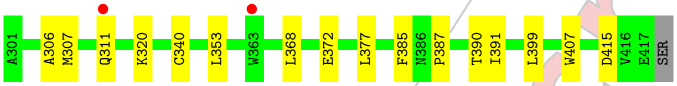

- Molecule 1: Candida glabrata strain CBS138 chromosome C complete sequence

Chain C:
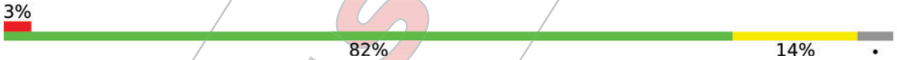

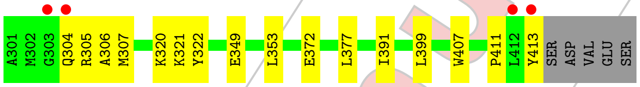

- Molecule 1: Candida glabrata strain CBS138 chromosome C complete sequence

Chain D:
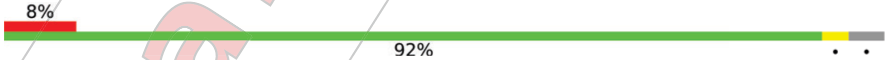

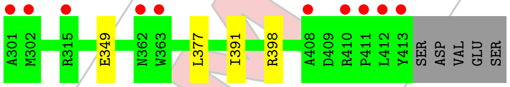

For Manuscript Review

4 Data and refinement statistics (i)

Property	Value	Source
Space group	C 2 2 21	Depositor
Cell constants<br>a, b, c, α, β, γ	70.59Å 88.43Å 257.79Å<br>90.00° 90.00° 90.00°	Depositor
Resolution (Å)	43.58 – 1.95<br>55.17 – 1.95	Depositor<br>EDS
% Data completeness<br>(in resolution range)	47.4 (43.58-1.95)<br>47.4 (55.17-1.95)	Depositor<br>EDS
R_{merge}	(Not available)	Depositor
R_{sym}	(Not available)	Depositor
$< I/\sigma(I) >$ [1]	1.64 (at 1.95Å)	Xtriage
Refinement program	PHENIX 1.20.1_4487	Depositor
R, R_{free}	0.195 , 0.240<br>0.195 , 0.239	Depositor<br>DCC
R_{free} test set	1407 reflections (4.99%)	wwPDB-VP
Wilson B-factor (Å^2)	24.3	Xtriage
Anisotropy	0.156	Xtriage
Bulk solvent k_{sol}(e/Å^3), B_{sol}(Å^2)	0.35 , 34.4	EDS
L-test for twinning[2]	$< \|L\| > = 0.50$, $< L^2 > = 0.33$	Xtriage
Estimated twinning fraction	No twinning to report.	Xtriage
F_o,F_c correlation	0.93	EDS
Total number of atoms	4257	wwPDB-VP
Average B, all atoms (Å^2)	36.0	wwPDB-VP

Xtriage's analysis on translational NCS is as follows: *The largest off-origin peak in the Patterson function is 6.79% of the height of the origin peak. No significant pseudotranslation is detected.*

[1] Intensities estimated from amplitudes.

[2] Theoretical values of $< |L| >$, $< L^2 >$ for acentric reflections are 0.5, 0.333 respectively for untwinned datasets, and 0.375, 0.2 for perfectly twinned datasets.

For Manuscript Review

5 Model quality (i)

5.1 Standard geometry (i)

Bond lengths and bond angles in the following residue types are not validated in this section: SO4, 1GH, GOL

The Z score for a bond length (or angle) is the number of standard deviations the observed value is removed from the expected value. A bond length (or angle) with $|Z| > 5$ is considered an outlier worth inspection. RMSZ is the root-mean-square of all Z scores of the bond lengths (or angles).

Mol	Chain	Bond lengths		Bond angles	
		RMSZ	#|Z| >5	RMSZ	#|Z| >5
1	A	0.25	0/973	0.45	0/1317
1	B	0.25	0/981	0.45	0/1329
1	C	0.25	0/955	0.45	0/1293
1	D	0.25	0/948	0.42	0/1284
All	All	0.25	0/3857	0.44	0/5223

There are no bond length outliers.

There are no bond angle outliers.

There are no chirality outliers.

There are no planarity outliers.

5.2 Too-close contacts (i)

In the following table, the Non-H and H(model) columns list the number of non-hydrogen atoms and hydrogen atoms in the chain respectively. The H(added) column lists the number of hydrogen atoms added and optimized by MolProbity. The Clashes column lists the number of clashes within the asymmetric unit, whereas Symm-Clashes lists symmetry-related clashes.

Mol	Chain	Non-H	H(model)	H(added)	Clashes	Symm-Clashes
1	A	947	0	901	7	0
1	B	955	0	911	10	0
1	C	929	0	891	10	0
1	D	922	0	876	5	0
2	A	48	0	64	3	0
2	B	42	0	56	1	0
2	C	30	0	40	1	0
2	D	24	0	32	1	0
3	A	10	0	0	0	0

Continued on next page...

For Manuscript Review

Continued from previous page...

Mol	Chain	Non-H	H(model)	H(added)	Clashes	Symm-Clashes
4	A	31	0	21	3	0
4	B	31	0	21	3	0
4	C	31	0	21	2	0
4	D	31	0	21	2	0
5	A	78	0	0	2	0
5	B	64	0	0	1	0
5	C	49	0	0	1	0
5	D	35	0	0	0	0
All	All	4257	0	3855	39	0

The all-atom clashscore is defined as the number of clashes found per 1000 atoms (including hydrogen atoms). The all-atom clashscore for this structure is 5.

All (39) close contacts within the same asymmetric unit are listed below, sorted by their clash magnitude.

Atom-1	Atom-2	Interatomic distance (Å)	Clash overlap (Å)
1:C:320:LYS:H	2:C:503:GOL:H2	1.58	0.69
1:D:398:ARG:HH22	2:D:503:GOL:H32	1.65	0.62
1:B:415:ASP:OD1	1:B:415:ASP:N	2.37	0.58
4:B:508:1GH:HA	4:B:508:1GH:H73C	1.86	0.58
1:C:349:GLU:HB3	1:C:377:LEU:HD21	1.84	0.57
1:A:391:ILE:HD13	1:D:391:ILE:HD13	1.86	0.56
1:A:349:GLU:HB3	1:A:377:LEU:HD21	1.91	0.53
1:D:349:GLU:HB3	1:D:377:LEU:HD21	1.91	0.52
4:D:505:1GH:HA	4:D:505:1GH:H73C	1.92	0.51
1:A:370:GLN:NE2	5:A:601:HOH:O	2.44	0.50
1:B:372:GLU:HB2	1:B:407:TRP:CZ2	2.47	0.50
5:A:612:HOH:O	4:B:508:1GH:H9	2.12	0.49
1:C:305:ARG:NH1	1:C:411:PRO:HB3	2.27	0.49
4:C:506:1GH:HA	4:C:506:1GH:H73C	1.95	0.48
4:A:511:1GH:H73C	4:A:511:1GH:HA	1.96	0.48
1:B:391:ILE:HD11	1:C:391:ILE:HD11	1.97	0.47
4:A:511:1GH:H9	5:B:629:HOH:O	2.14	0.46
1:B:353:LEU:HD11	1:B:399:LEU:HD21	1.98	0.46
1:B:307:MET:O	1:B:311:GLN:HG3	2.16	0.46
1:A:320:LYS:HE3	2:A:508:GOL:H11	1.98	0.45
1:A:394:MET:SD	1:D:391:ILE:HD11	2.57	0.45
1:B:306:ALA:HB1	1:B:368:LEU:HG	1.98	0.45
1:A:413:TYR:H	2:A:503:GOL:H31	1.81	0.45
1:C:353:LEU:HD11	1:C:399:LEU:HD21	2.00	0.44
1:C:321:LYS:HG2	1:C:322:TYR:CE1	2.53	0.44

Continued on next page...

For Manuscript Review

Continued from previous page...

Atom-1	Atom-2	Interatomic distance (Å)	Clash overlap (Å)
1:C:306:ALA:HB2	1:C:413:TYR:OH	2.18	0.43
1:B:377:LEU:HD12	1:B:377:LEU:HA	1.90	0.42
1:C:321:LYS:HB2	1:C:321:LYS:HE3	1.84	0.41
4:C:506:1GH:H71C	4:C:506:1GH:H9	1.88	0.41
1:B:340:CYS:HB2	1:B:385:PHE:CZ	2.56	0.41
5:C:636:HOH:O	4:D:505:1GH:H9	2.21	0.41
4:B:508:1GH:H9	4:B:508:1GH:H71C	1.87	0.41
1:C:304:GLN:HA	1:C:307:MET:HB2	2.03	0.41
1:B:387:PRO:HD2	1:B:390:THR:HG21	2.02	0.41
1:C:372:GLU:HB2	1:C:407:TRP:CZ2	2.56	0.40
1:D:391:ILE:H	1:D:391:ILE:HG12	1.55	0.40
1:A:385:PHE:O	2:A:505:GOL:H32	2.22	0.40
4:A:511:1GH:H9	4:A:511:1GH:H71C	1.81	0.40
1:B:320:LYS:H	2:B:502:GOL:H2	1.87	0.40

There are no symmetry-related clashes.

5.3 Torsion angles ⓘ

5.3.1 Protein backbone ⓘ

In the following table, the Percentiles column shows the percent Ramachandran outliers of the chain as a percentile score with respect to all X-ray entries followed by that with respect to entries of similar resolution.

The Analysed column shows the number of residues for which the backbone conformation was analysed, and the total number of residues.

Mol	Chain	Analysed	Favoured	Allowed	Outliers	Percentiles	
1	A	114/118 (97%)	112 (98%)	2 (2%)	0	100	100
1	B	115/118 (98%)	114 (99%)	1 (1%)	0	100	100
1	C	111/118 (94%)	110 (99%)	1 (1%)	0	100	100
1	D	111/118 (94%)	109 (98%)	2 (2%)	0	100	100
All	All	451/472 (96%)	445 (99%)	6 (1%)	0	100	100

There are no Ramachandran outliers to report.

For Manuscript Review

5.3.2 Protein sidechains (i)

In the following table, the Percentiles column shows the percent sidechain outliers of the chain as a percentile score with respect to all X-ray entries followed by that with respect to entries of similar resolution.

The Analysed column shows the number of residues for which the sidechain conformation was analysed, and the total number of residues.

Mol	Chain	Analysed	Rotameric	Outliers	Percentiles	
1	A	103/106 (97%)	103 (100%)	0	100	100
1	B	104/106 (98%)	104 (100%)	0	100	100
1	C	101/106 (95%)	101 (100%)	0	100	100
1	D	100/106 (94%)	100 (100%)	0	100	100
All	All	408/424 (96%)	408 (100%)	0	100	100

There are no protein residues with a non-rotameric sidechain to report.

Sometimes sidechains can be flipped to improve hydrogen bonding and reduce clashes. There are no such sidechains identified.

5.3.3 RNA (i)

There are no RNA molecules in this entry.

5.4 Non-standard residues in protein, DNA, RNA chains (i)

There are no non-standard protein/DNA/RNA residues in this entry.

5.5 Carbohydrates (i)

There are no monosaccharides in this entry.

5.6 Ligand geometry (i)

30 ligands are modelled in this entry.

In the following table, the Counts columns list the number of bonds (or angles) for which Mogul statistics could be retrieved, the number of bonds (or angles) that are observed in the model and the number of bonds (or angles) that are defined in the Chemical Component Dictionary. The Link column lists molecule types, if any, to which the group is linked. The Z score for a bond length (or angle) is the number of standard deviations the observed value is removed from the

For Manuscript Review

expected value. A bond length (or angle) with $|Z| > 2$ is considered an outlier worth inspection. RMSZ is the root-mean-square of all Z scores of the bond lengths (or angles).

Mol	Type	Chain	Res	Link	Bond lengths			Bond angles		
					Counts	RMSZ	#|Z| > 2	Counts	RMSZ	#|Z| > 2
2	GOL	D	504	-	5,5,5	0.92	0	5,5,5	1.01	0
2	GOL	C	501	-	5,5,5	0.92	0	5,5,5	0.98	0
4	1GH	A	511	-	30,35,35	0.97	2 (6%)	33,52,52	1.91	9 (27%)
4	1GH	D	505	-	30,35,35	1.00	2 (6%)	33,52,52	1.84	8 (24%)
3	SO4	A	509	-	4,4,4	0.13	0	6,6,6	0.08	0
2	GOL	C	502	-	5,5,5	0.90	0	5,5,5	1.06	0
4	1GH	B	508	-	30,35,35	0.98	2 (6%)	33,52,52	1.87	9 (27%)
2	GOL	A	502	-	5,5,5	0.98	0	5,5,5	0.86	0
2	GOL	D	503	-	5,5,5	0.92	0	5,5,5	0.97	0
2	GOL	A	506	-	5,5,5	0.91	0	5,5,5	0.97	0
2	GOL	B	505	-	5,5,5	0.98	0	5,5,5	1.00	0
2	GOL	B	503	-	5,5,5	0.92	0	5,5,5	0.98	0
2	GOL	C	505	-	5,5,5	0.86	0	5,5,5	1.02	0
2	GOL	A	507	-	5,5,5	0.91	0	5,5,5	1.03	0
2	GOL	C	504	-	5,5,5	0.90	0	5,5,5	1.06	0
2	GOL	A	505	-	5,5,5	0.90	0	5,5,5	1.01	0
3	SO4	A	510	-	4,4,4	0.14	0	6,6,6	0.04	0
2	GOL	A	503	-	5,5,5	1.00	0	5,5,5	0.91	0
2	GOL	A	501	-	5,5,5	0.93	0	5,5,5	0.96	0
2	GOL	B	506	-	5,5,5	0.90	0	5,5,5	1.02	0
4	1GH	C	506	-	30,35,35	0.99	2 (6%)	33,52,52	1.83	9 (27%)
2	GOL	B	502	-	5,5,5	0.95	0	5,5,5	1.00	0
2	GOL	B	507	-	5,5,5	0.92	0	5,5,5	1.01	0
2	GOL	A	504	-	5,5,5	0.95	0	5,5,5	0.95	0
2	GOL	A	508	-	5,5,5	0.90	0	5,5,5	1.03	0
2	GOL	D	501	-	5,5,5	0.93	0	5,5,5	0.98	0
2	GOL	C	503	-	5,5,5	0.89	0	5,5,5	1.02	0
2	GOL	D	502	-	5,5,5	0.90	0	5,5,5	1.02	0
2	GOL	B	501	-	5,5,5	0.94	0	5,5,5	0.98	0
2	GOL	B	504	-	5,5,5	0.93	0	5,5,5	0.96	0

In the following table, the Chirals column lists the number of chiral outliers, the number of chiral centers analysed, the number of these observed in the model and the number defined in the Chemical Component Dictionary. Similar counts are reported in the Torsion and Rings columns. '-' means no outliers of that kind were identified.

Mol	Type	Chain	Res	Link	Chirals	Torsions	Rings
2	GOL	D	504	-	-	0/4/4/4	-
2	GOL	C	501	-	-	4/4/4/4	-

Continued on next page...

For Manuscript Review

Continued from previous page...

Mol	Type	Chain	Res	Link	Chirals	Torsions	Rings
4	1GH	A	511	-	-	0/14/14/14	0/5/5/5
4	1GH	D	505	-	-	0/14/14/14	0/5/5/5
2	GOL	C	502	-	-	2/4/4/4	-
4	1GH	B	508	-	-	0/14/14/14	0/5/5/5
2	GOL	A	502	-	-	2/4/4/4	-
2	GOL	D	503	-	-	0/4/4/4	-
2	GOL	A	506	-	-	4/4/4/4	-
2	GOL	B	505	-	-	2/4/4/4	-
2	GOL	B	503	-	-	4/4/4/4	-
2	GOL	C	505	-	-	0/4/4/4	-
2	GOL	A	507	-	-	0/4/4/4	-
2	GOL	C	504	-	-	0/4/4/4	-
2	GOL	A	505	-	-	0/4/4/4	-
2	GOL	A	503	-	-	2/4/4/4	-
2	GOL	A	501	-	-	4/4/4/4	-
2	GOL	B	506	-	-	2/4/4/4	-
4	1GH	C	506	-	-	0/14/14/14	0/5/5/5
2	GOL	B	502	-	-	4/4/4/4	-
2	GOL	B	507	-	-	0/4/4/4	-
2	GOL	A	504	-	-	2/4/4/4	-
2	GOL	A	508	-	-	0/4/4/4	-
2	GOL	D	501	-	-	2/4/4/4	-
2	GOL	C	503	-	-	2/4/4/4	-
2	GOL	D	502	-	-	1/4/4/4	-
2	GOL	B	501	-	-	4/4/4/4	-
2	GOL	B	504	-	-	4/4/4/4	-

All (8) bond length outliers are listed below:

Mol	Chain	Res	Type	Atoms	Z	Observed(Å)	Ideal(Å)
4	D	505	1GH	C13-N3	-2.51	1.34	1.37
4	B	508	1GH	C23-C22	2.43	1.51	1.48
4	D	505	1GH	C23-C22	2.42	1.51	1.48
4	A	511	1GH	C23-C22	2.37	1.51	1.48
4	C	506	1GH	C23-C22	2.33	1.51	1.48
4	C	506	1GH	C13-N3	-2.31	1.34	1.37
4	B	508	1GH	C13-N3	-2.24	1.34	1.37
4	A	511	1GH	C13-N3	-2.22	1.34	1.37

For Manuscript Review

All (35) bond angle outliers are listed below:

Mol	Chain	Res	Type	Atoms	Z	Observed(^{o})	Ideal(^{o})
4	B	508	1GH	C14-C5-C4	-4.54	116.22	122.21
4	A	511	1GH	C14-C5-C4	-4.52	116.24	122.21
4	A	511	1GH	O1-C2-C3	-4.48	119.44	125.24
4	C	506	1GH	C14-C5-C4	-4.43	116.36	122.21
4	D	505	1GH	C14-C5-C4	-4.35	116.47	122.21
4	B	508	1GH	O1-C2-C3	-4.28	119.70	125.24
4	C	506	1GH	O1-C2-C3	-4.12	119.92	125.24
4	D	505	1GH	O1-C2-C3	-4.11	119.93	125.24
4	B	508	1GH	C12-N2-C8	3.97	121.97	117.32
4	D	505	1GH	C15-N4-C16	3.92	121.62	116.91
4	A	511	1GH	C15-N4-C16	3.78	121.46	116.91
4	C	506	1GH	C15-N4-C16	3.70	121.37	116.91
4	B	508	1GH	C15-N4-C16	3.65	121.31	116.91
4	D	505	1GH	C12-N2-C8	3.65	121.60	117.32
4	C	506	1GH	C12-N2-C8	3.63	121.58	117.32
4	A	511	1GH	C12-N2-C8	3.63	121.57	117.32
4	A	511	1GH	O2-C13-N3	-3.17	124.67	127.50
4	D	505	1GH	C14-N3-C13	-3.01	108.46	110.28
4	D	505	1GH	O2-C13-N3	-2.92	124.89	127.50
4	C	506	1GH	C14-N3-C13	-2.83	108.57	110.28
4	A	511	1GH	C14-N3-C13	-2.83	108.57	110.28
4	C	506	1GH	O2-C13-N3	-2.78	125.01	127.50
4	B	508	1GH	O2-C13-N3	-2.62	125.15	127.50
4	A	511	1GH	N3-C13-N1	2.61	110.06	106.66
4	D	505	1GH	N3-C13-N1	2.57	110.00	106.66
4	C	506	1GH	N3-C13-N1	2.52	109.94	106.66
4	B	508	1GH	N3-C13-N1	2.47	109.88	106.66
4	B	508	1GH	C14-N3-C13	-2.43	108.81	110.28
4	B	508	1GH	C21-C20-N5	2.37	126.48	119.19
4	A	511	1GH	O1-C2-C18	2.28	119.66	116.26
4	C	506	1GH	C21-C20-N5	2.26	126.14	119.19
4	D	505	1GH	C21-C20-N5	2.23	126.03	119.19
4	A	511	1GH	C21-C20-N5	2.19	125.91	119.19
4	B	508	1GH	O1-C2-C18	2.09	119.37	116.26
4	C	506	1GH	O1-C2-C18	2.02	119.28	116.26

There are no chirality outliers.

All (45) torsion outliers are listed below:

Mol	Chain	Res	Type	Atoms
2	A	501	GOL	O1-C1-C2-C3

Continued on next page...

For Manuscript Review

Continued from previous page...

Mol	Chain	Res	Type	Atoms
2	A	502	GOL	C1-C2-C3-O3
2	A	506	GOL	O1-C1-C2-O2
2	A	506	GOL	O1-C1-C2-C3
2	A	506	GOL	C1-C2-C3-O3
2	A	506	GOL	O2-C2-C3-O3
2	B	501	GOL	C1-C2-C3-O3
2	B	503	GOL	C1-C2-C3-O3
2	B	504	GOL	O1-C1-C2-O2
2	B	504	GOL	C1-C2-C3-O3
2	B	506	GOL	C1-C2-C3-O3
2	C	501	GOL	O1-C1-C2-C3
2	C	501	GOL	C1-C2-C3-O3
2	C	502	GOL	O1-C1-C2-O2
2	C	502	GOL	O1-C1-C2-C3
2	D	501	GOL	C1-C2-C3-O3
2	A	501	GOL	O1-C1-C2-O2
2	A	504	GOL	O2-C2-C3-O3
2	C	501	GOL	O2-C2-C3-O3
2	A	501	GOL	C1-C2-C3-O3
2	A	503	GOL	O1-C1-C2-C3
2	A	504	GOL	C1-C2-C3-O3
2	B	501	GOL	O1-C1-C2-C3
2	B	502	GOL	O1-C1-C2-C3
2	B	502	GOL	C1-C2-C3-O3
2	B	503	GOL	O1-C1-C2-C3
2	B	504	GOL	O1-C1-C2-C3
2	C	503	GOL	C1-C2-C3-O3
2	A	502	GOL	O2-C2-C3-O3
2	B	501	GOL	O1-C1-C2-O2
2	B	503	GOL	O1-C1-C2-O2
2	B	503	GOL	O2-C2-C3-O3
2	B	504	GOL	O2-C2-C3-O3
2	B	506	GOL	O2-C2-C3-O3
2	D	501	GOL	O2-C2-C3-O3
2	C	503	GOL	O2-C2-C3-O3
2	A	503	GOL	O1-C1-C2-O2
2	B	501	GOL	O2-C2-C3-O3
2	A	501	GOL	O2-C2-C3-O3
2	B	502	GOL	O1-C1-C2-O2
2	B	505	GOL	O1-C1-C2-O2
2	C	501	GOL	O1-C1-C2-O2
2	B	502	GOL	O2-C2-C3-O3

Continued on next page...

For Manuscript Review

Continued from previous page...

Mol	Chain	Res	Type	Atoms
2	B	505	GOL	O1-C1-C2-C3
2	D	502	GOL	O1-C1-C2-C3

There are no ring outliers.

10 monomers are involved in 16 short contacts:

Mol	Chain	Res	Type	Clashes	Symm-Clashes
4	A	511	1GH	3	0
4	D	505	1GH	2	0
4	B	508	1GH	3	0
2	D	503	GOL	1	0
2	A	505	GOL	1	0
2	A	503	GOL	1	0
4	C	506	1GH	2	0
2	B	502	GOL	1	0
2	A	508	GOL	1	0
2	C	503	GOL	1	0

The following is a two-dimensional graphical depiction of Mogul quality analysis of bond lengths, bond angles, torsion angles, and ring geometry for all instances of the Ligand of Interest. In addition, ligands with molecular weight > 250 and outliers as shown on the validation Tables will also be included. For torsion angles, if less then 5% of the Mogul distribution of torsion angles is within 10 degrees of the torsion angle in question, then that torsion angle is considered an outlier. Any bond that is central to one or more torsion angles identified as an outlier by Mogul will be highlighted in the graph. For rings, the root-mean-square deviation (RMSD) between the ring in question and similar rings identified by Mogul is calculated over all ring torsion angles. If the average RMSD is greater than 60 degrees and the minimal RMSD between the ring in question and any Mogul-identified rings is also greater than 60 degrees, then that ring is considered an outlier. The outliers are highlighted in purple. The color gray indicates Mogul did not find sufficient equivalents in the CSD to analyse the geometry.

For Manuscript Review

Ligand 1GH A 511

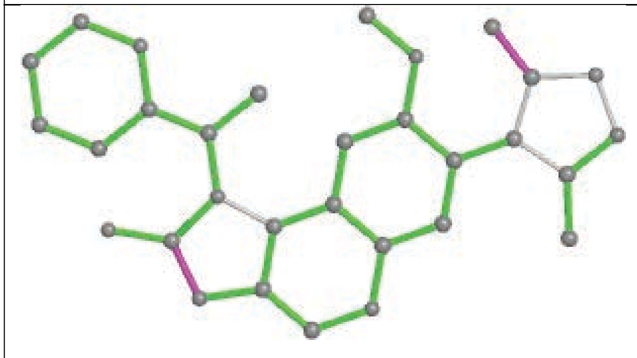

Bond lengths

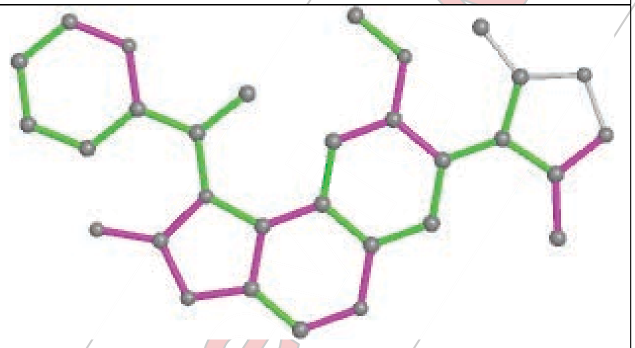

Bond angles

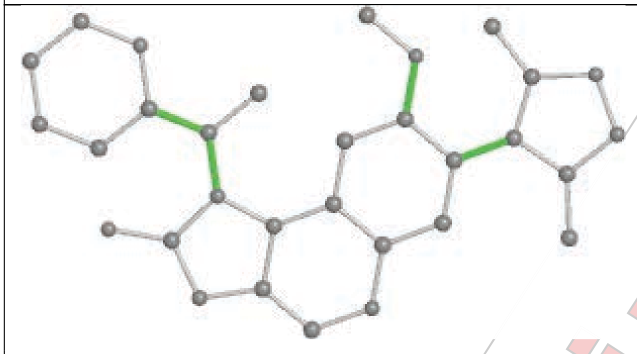

Torsions

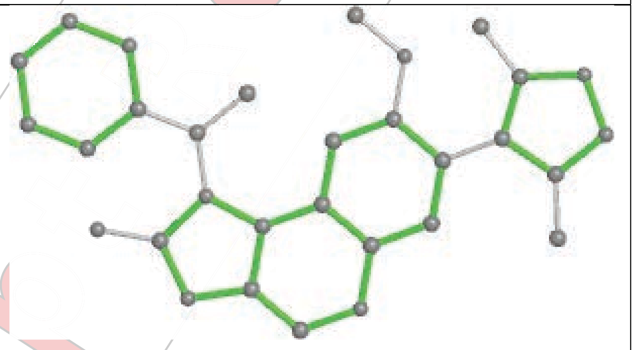

Rings

Ligand 1GH D 505

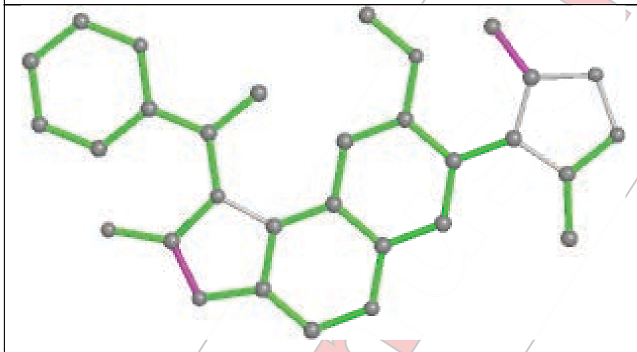

Bond lengths

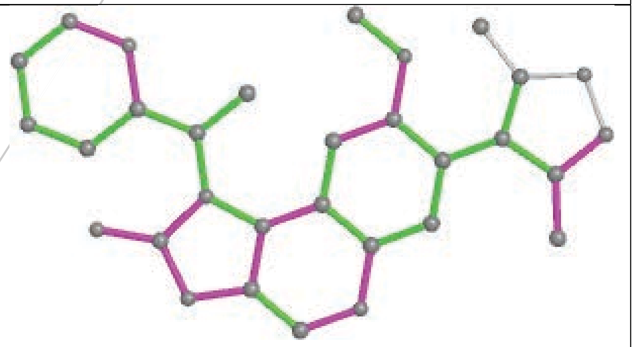

Bond angles

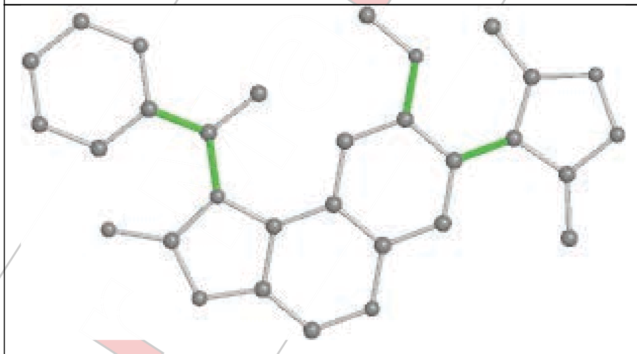

Torsions

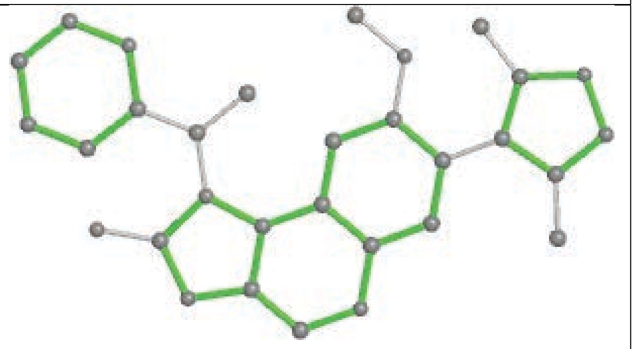

Rings

For Manuscript Review

Ligand 1GH B 508

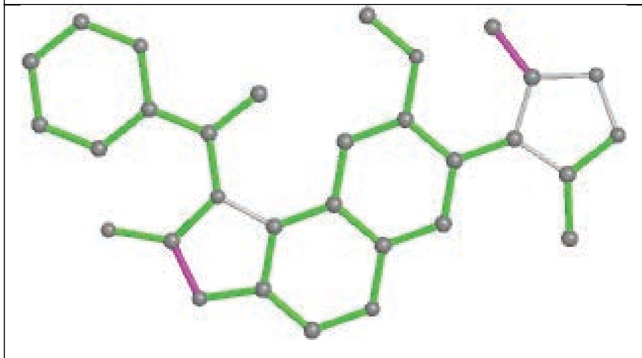
Bond lengths

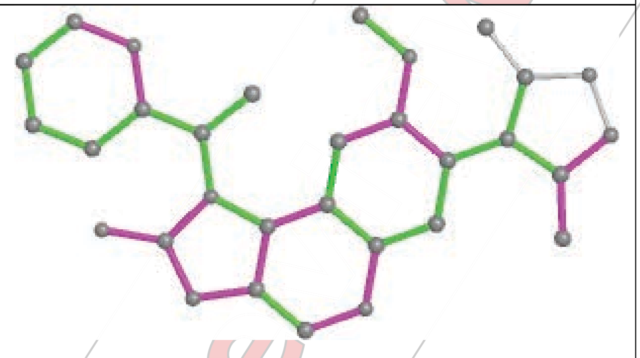
Bond angles

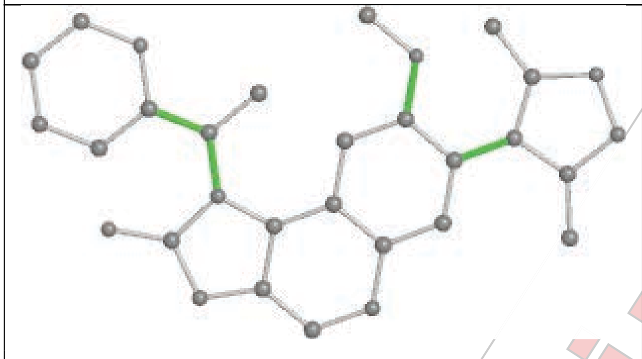
Torsions

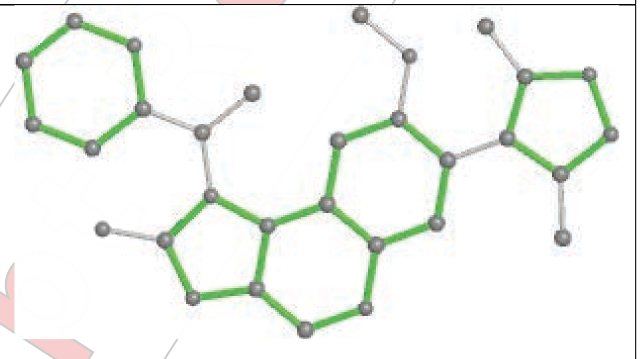
Rings

Ligand 1GH C 506

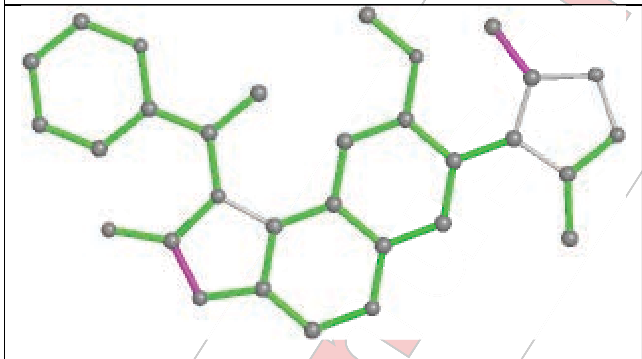
Bond lengths

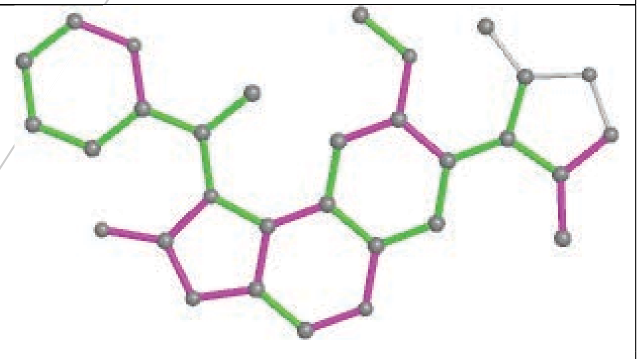
Bond angles

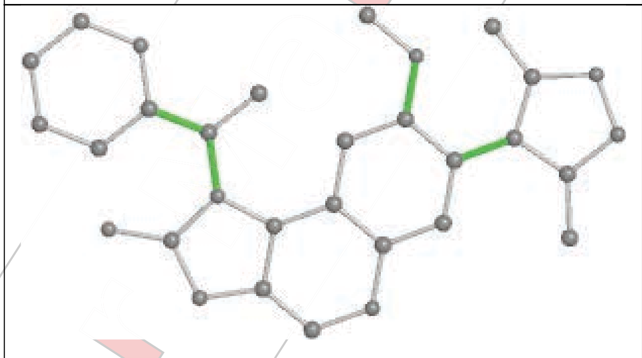
Torsions

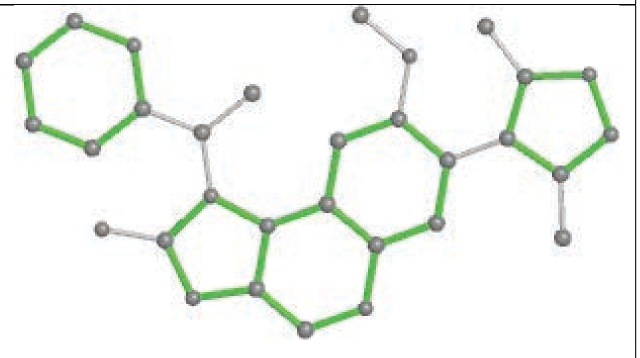
Rings

5.7 Other polymers (i)

There are no such residues in this entry.

5.8 Polymer linkage issues (i)

There are no chain breaks in this entry.

For Manuscript Review

6 Fit of model and data (i)

6.1 Protein, DNA and RNA chains (i)

In the following table, the column labelled '#RSRZ> 2' contains the number (and percentage) of RSRZ outliers, followed by percent RSRZ outliers for the chain as percentile scores relative to all X-ray entries and entries of similar resolution. The OWAB column contains the minimum, median, 95th percentile and maximum values of the occupancy-weighted average B-factor per residue. The column labelled 'Q< 0.9' lists the number of (and percentage) of residues with an average occupancy less than 0.9.

Mol	Chain	Analysed	<RSRZ>	#RSRZ>2			OWAB(Å^2)	Q<0.9
1	A	116/118 (98%)	0.34	1 (0%)	84	89	15, 26, 40, 64	0
1	B	117/118 (99%)	0.35	2 (1%)	70	77	21, 31, 57, 68	0
1	C	113/118 (95%)	0.40	4 (3%)	44	53	17, 33, 57, 70	0
1	D	113/118 (95%)	0.72	10 (8%)	10	16	26, 44, 79, 109	0
All	All	459/472 (97%)	0.45	17 (3%)	41	51	15, 32, 65, 109	0

All (17) RSRZ outliers are listed below:

Mol	Chain	Res	Type	RSRZ
1	D	413	TYR	6.3
1	D	412	LEU	5.9
1	D	363	TRP	4.3
1	D	408	ALA	3.7
1	C	413	TYR	3.4
1	B	363	TRP	3.1
1	D	411	PRO	3.0
1	D	302	MET	2.9
1	D	315	ARG	2.6
1	B	311	GLN	2.6
1	C	304	GLN	2.4
1	D	410	ARG	2.3
1	C	412	LEU	2.3
1	A	415	ASP	2.2
1	D	301	ALA	2.2
1	D	362	ASN	2.1
1	C	303	GLY	2.1

6.2 Non-standard residues in protein, DNA, RNA chains (i)

There are no non-standard protein/DNA/RNA residues in this entry.

For Manuscript Review

6.3 Carbohydrates (i)

There are no monosaccharides in this entry.

6.4 Ligands (i)

In the following table, the Atoms column lists the number of modelled atoms in the group and the number defined in the chemical component dictionary. The B-factors column lists the minimum, median, 95th percentile and maximum values of B factors of atoms in the group. The column labelled 'Q< 0.9' lists the number of atoms with occupancy less than 0.9.

Mol	Type	Chain	Res	Atoms	RSCC	RSR	B-factors(Å^2)	Q<0.9
2	GOL	B	506	6/6	0.79	0.29	55,59,64,68	0
2	GOL	A	507	6/6	0.81	0.31	44,52,57,77	0
2	GOL	C	505	6/6	0.82	0.28	53,56,58,70	0
2	GOL	C	503	6/6	0.86	0.27	51,59,63,66	0
2	GOL	C	504	6/6	0.86	0.30	48,53,58,61	0
2	GOL	B	507	6/6	0.86	0.26	44,52,52,61	0
2	GOL	A	505	6/6	0.88	0.12	41,48,51,54	0
2	GOL	D	504	6/6	0.88	0.18	44,53,58,62	0
3	SO4	A	510	5/5	0.88	0.36	91,104,108,109	0
2	GOL	C	501	6/6	0.89	0.19	37,47,58,59	0
2	GOL	A	506	6/6	0.89	0.23	42,49,53,57	0
2	GOL	D	503	6/6	0.90	0.19	40,52,56,57	0
2	GOL	B	504	6/6	0.91	0.18	37,42,51,84	0
2	GOL	C	502	6/6	0.91	0.12	37,45,49,52	0
2	GOL	A	503	6/6	0.91	0.19	16,33,39,62	0
2	GOL	A	501	6/6	0.91	0.17	43,46,53,65	0
2	GOL	B	503	6/6	0.92	0.14	34,39,43,43	0
2	GOL	A	508	6/6	0.93	0.20	42,52,54,55	0
2	GOL	B	505	6/6	0.93	0.20	35,46,54,55	0
2	GOL	B	502	6/6	0.93	0.11	35,40,45,66	0
2	GOL	A	504	6/6	0.93	0.14	33,40,51,52	0
2	GOL	D	501	6/6	0.94	0.17	35,39,51,52	0
2	GOL	B	501	6/6	0.94	0.13	29,35,45,45	0
4	1GH	B	508	31/31	0.95	0.13	17,23,34,38	0
4	1GH	A	511	31/31	0.96	0.13	12,21,28,33	0
2	GOL	D	502	6/6	0.96	0.10	41,44,46,50	0
3	SO4	A	509	5/5	0.97	0.08	48,51,60,65	0
2	GOL	A	502	6/6	0.97	0.12	21,25,30,33	0
4	1GH	C	506	31/31	0.97	0.13	15,22,29,36	0
4	1GH	D	505	31/31	0.98	0.13	18,25,33,36	0

The following is a graphical depiction of the model fit to experimental electron density of all

For Manuscript Review

instances of the Ligand of Interest. In addition, ligands with molecular weight > 250 and outliers as shown on the geometry validation Tables will also be included. Each fit is shown from different orientation to approximate a three-dimensional view.

Electron density around 1GH B 508:

$2mF_o$-DF_c (at 0.7 rmsd) in gray
mF_o-DF_c (at 3 rmsd) in purple (negative) and green (positive)

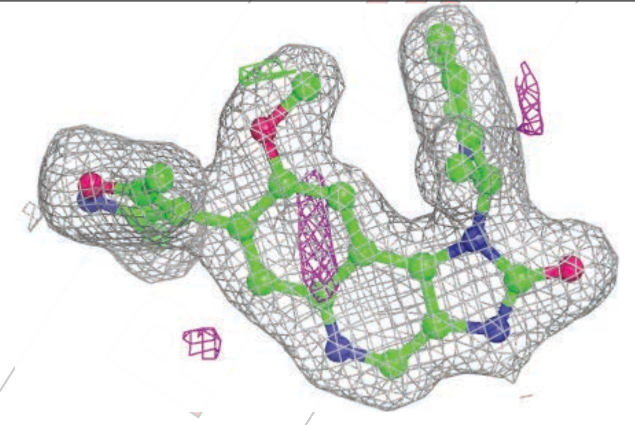

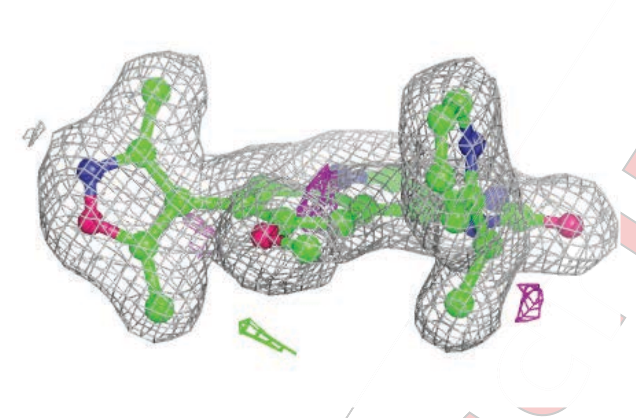

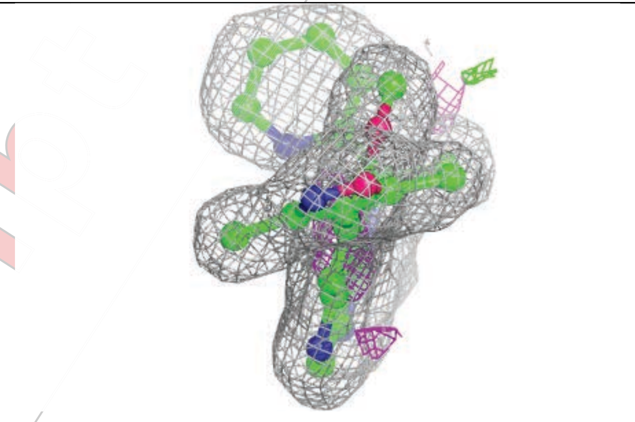

For Manuscript Review

Electron density around 1GH A 511:

$2mF_o$-DF_c (at 0.7 rmsd) in gray
mF_o-DF_c (at 3 rmsd) in purple (negative)
and green (positive)

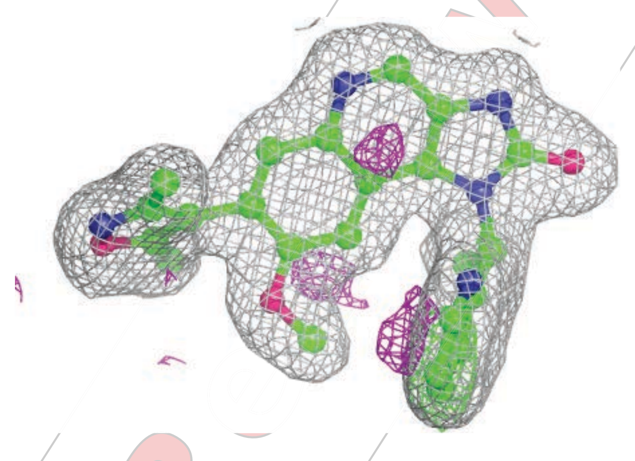

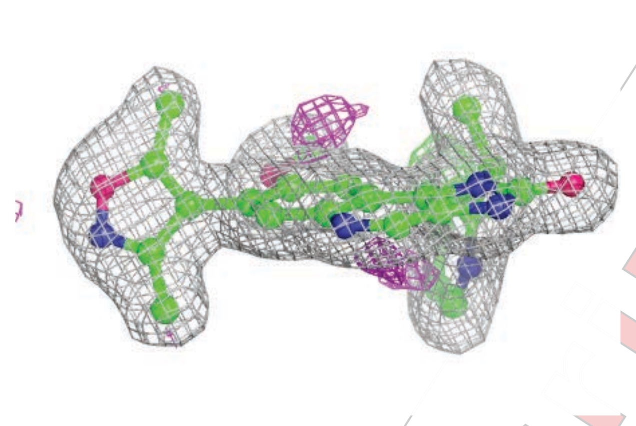

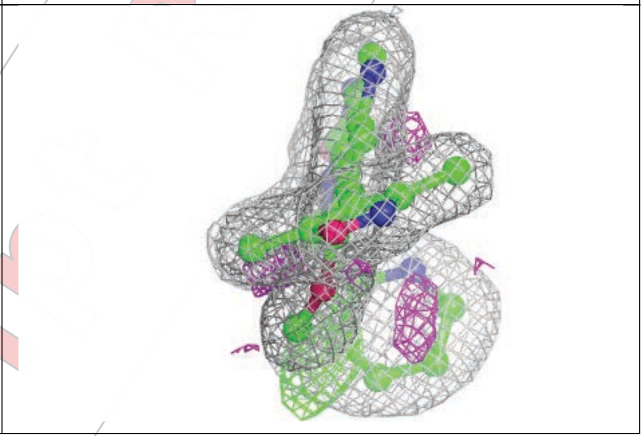

For Manuscript Review

Electron density around 1GH C 506:

$2mF_o$-DF_c (at 0.7 rmsd) in gray
mF_o-DF_c (at 3 rmsd) in purple (negative)
and green (positive)

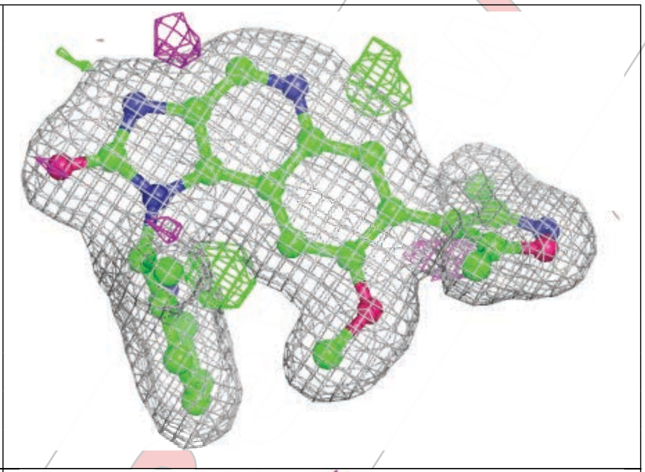

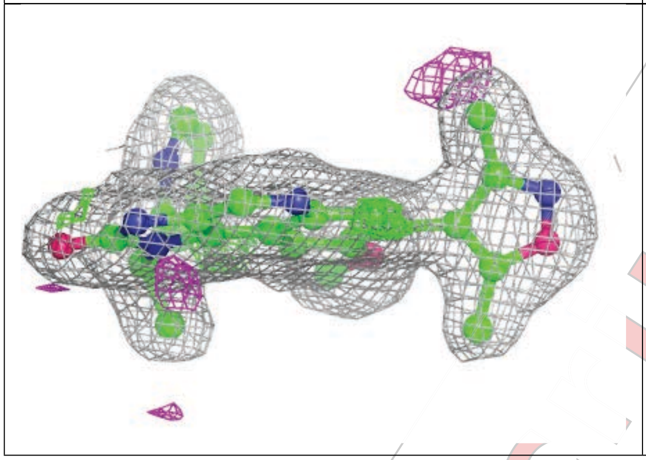

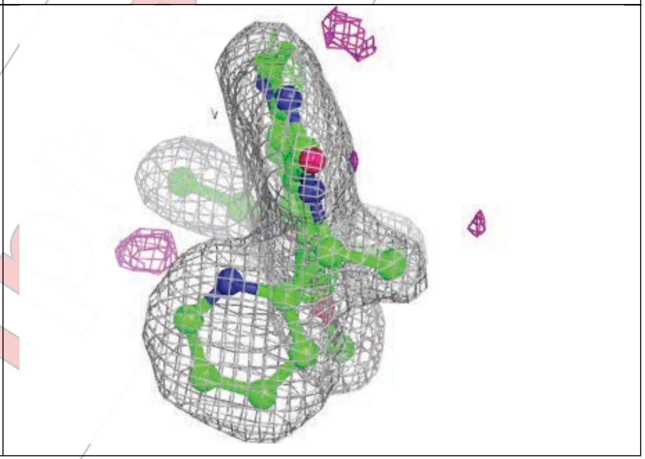

For Manuscript Review

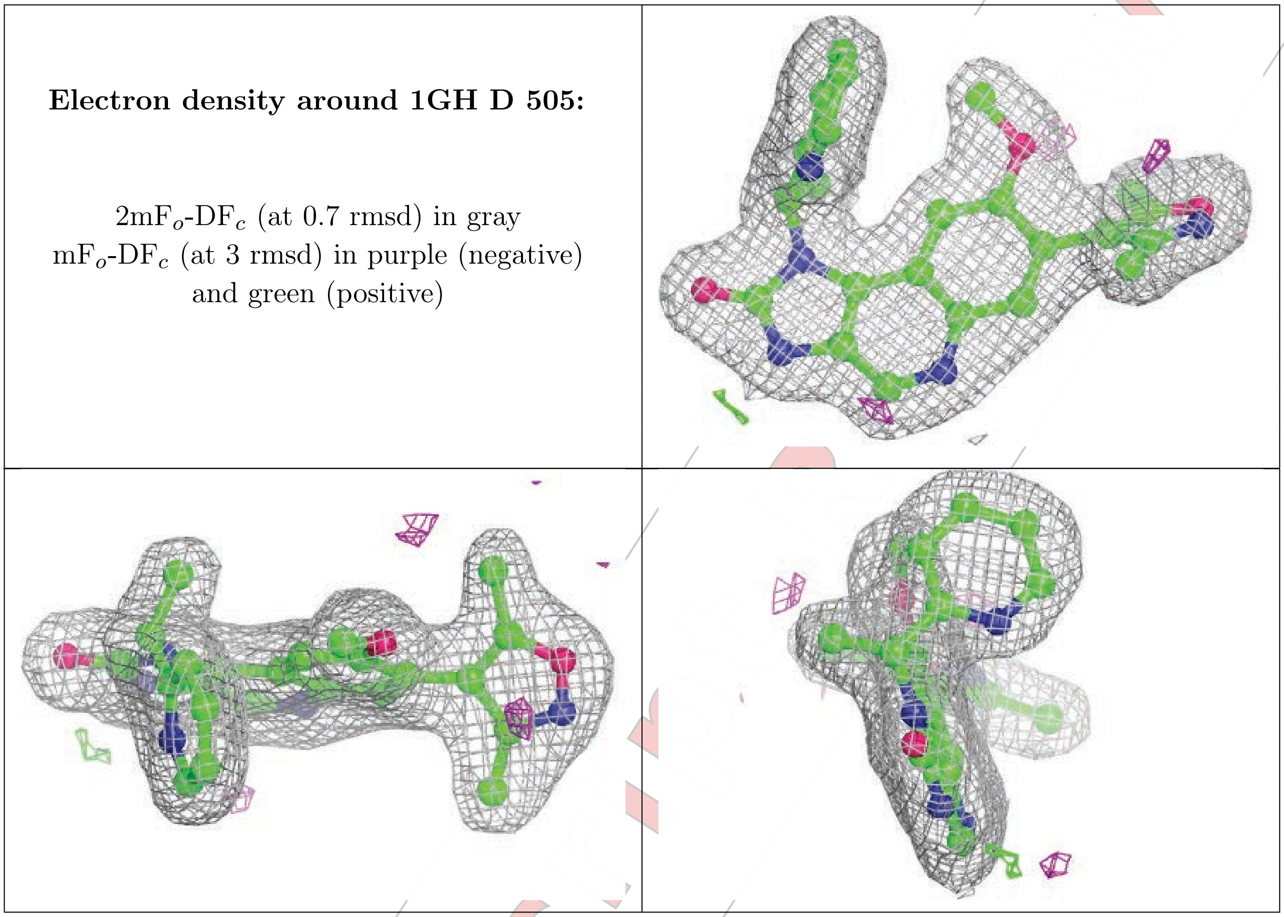

6.5 Other polymers (i)

There are no such residues in this entry.

For Manuscript Review

PDB validation report for CgBD2 bound to 29 (8R6N)

For Manuscript Review

WORLDWIDE PDB PROTEIN DATA BANK

Full wwPDB X-ray Structure Validation Report (i)

Nov 23, 2023 – 02:31 pm GMT

PDB ID	:	8R6N
Title	:	Crystal structure of Candida glabrata Bdf1 bromodomain 2 bound to a pyridoindole ligand
Deposited on	:	2023-11-22
Resolution	:	2.11 Å(reported)

This wwPDB validation report is for manuscript review

This is a Full wwPDB X-ray Structure Validation Report.

This report is produced by the wwPDB biocuration pipeline after annotation of the structure.

We welcome your comments at *validation@mail.wwpdb.org*
A user guide is available at
https://www.wwpdb.org/validation/2017/XrayValidationReportHelp
with specific help available everywhere you see the (i) symbol.

The types of validation reports are described at
http://www.wwpdb.org/validation/2017/FAQs#types.

The following versions of software and data (see references (i)) were used in the production of this report:

MolProbity	:	4.02b-467
Mogul	:	1.8.4, CSD as541be (2020)
Xtriage (Phenix)	:	1.13
EDS	:	2.36
buster-report	:	1.1.7 (2018)
Percentile statistics	:	20191225.v01 (using entries in the PDB archive December 25th 2019)
Refmac	:	5.8.0158
CCP4	:	7.0.044 (Gargrove)
Ideal geometry (proteins)	:	Engh & Huber (2001)

1 Overall quality at a glance (i)

The following experimental techniques were used to determine the structure:
X-RAY DIFFRACTION

The reported resolution of this entry is 2.11 Å.

Percentile scores (ranging between 0-100) for global validation metrics of the entry are shown in the following graphic. The table shows the number of entries on which the scores are based.

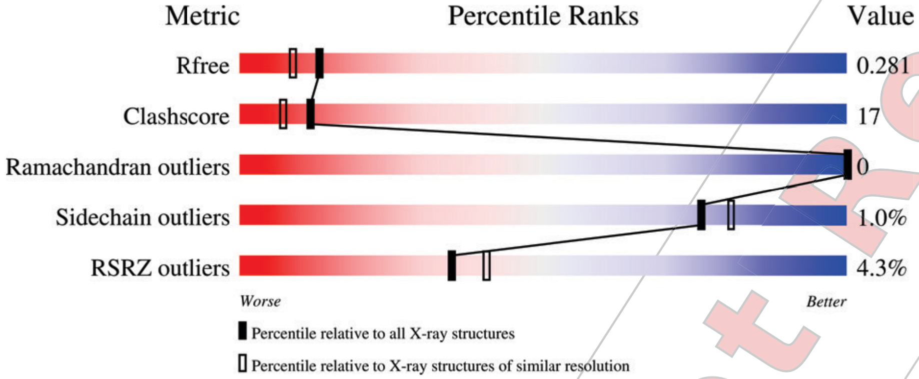

Metric	Whole archive (#Entries)	Similar resolution (#Entries, resolution range(Å))
R_{free}	130704	6241 (2.14-2.10)
Clashscore	141614	6778 (2.14-2.10)
Ramachandran outliers	138981	6705 (2.14-2.10)
Sidechain outliers	138945	6706 (2.14-2.10)
RSRZ outliers	127900	6112 (2.14-2.10)

The table below summarises the geometric issues observed across the polymeric chains and their fit to the electron density. The red, orange, yellow and green segments of the lower bar indicate the fraction of residues that contain outliers for >=3, 2, 1 and 0 types of geometric quality criteria respectively. A grey segment represents the fraction of residues that are not modelled. The numeric value for each fraction is indicated below the corresponding segment, with a dot representing fractions <=5% The upper red bar (where present) indicates the fraction of residues that have poor fit to the electron density. The numeric value is given above the bar.

Mol	Chain	Length	Quality of chain
1	A	118	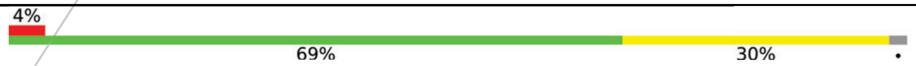

Ideal geometry (DNA, RNA) : Parkinson et al. (1996)
Validation Pipeline (wwPDB-VP) : 2.36

For Manuscript Review

2 Entry composition (i)

There are 3 unique types of molecules in this entry. The entry contains 1030 atoms, of which 26 are hydrogens and 0 are deuteriums.

In the tables below, the ZeroOcc column contains the number of atoms modelled with zero occupancy, the AltConf column contains the number of residues with at least one atom in alternate conformation and the Trace column contains the number of residues modelled with at most 2 atoms.

- Molecule 1 is a protein called Candida glabrata strain CBS138 chromosome C complete sequence.

Mol	Chain	Residues	Atoms					ZeroOcc	AltConf	Trace
			Total	C	N	O	S			
1	A	116	950	609	156	176	9	0	0	0

There are 3 discrepancies between the modelled and reference sequences:

Chain	Residue	Modelled	Actual	Comment	Reference
A	301	ALA	-	expression tag	UNP Q6FWV7
A	302	MET	-	expression tag	UNP Q6FWV7
A	303	GLY	-	expression tag	UNP Q6FWV7

- Molecule 2 is 2-ethanoyl- {N}-(4-morpholin-4-ylphenyl)-1,3,4,5-tetrahydropyrido[4,3-b]indole-8-carboxamide (three-letter code: Y7B) (formula: $C_{24}H_{26}N_4O_3$) (labeled as "Ligand of Interest" by depositor).

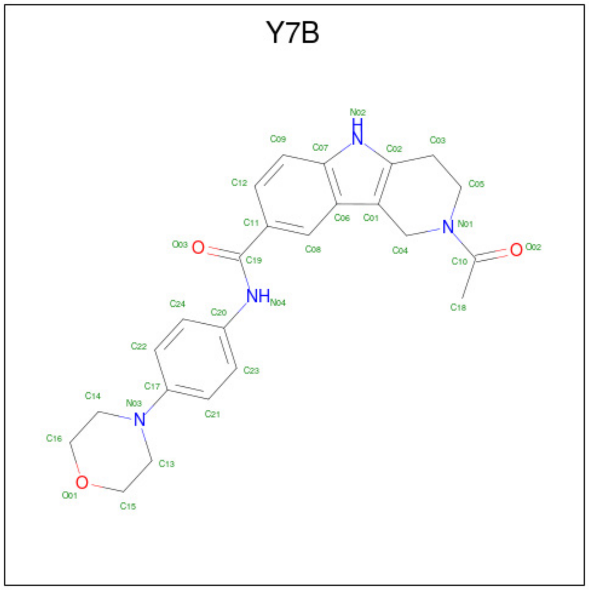

For Manuscript Review

Mol	Chain	Residues	Atoms					ZeroOcc	AltConf
			Total	C	H	N	O		
2	A	1	57	24	26	4	3	0	0

- Molecule 3 is water.

Mol	Chain	Residues	Atoms		ZeroOcc	AltConf
			Total	O		
3	A	23	23	23	0	0

For Manuscript Review

3 Residue-property plots (i)

These plots are drawn for all protein, RNA, DNA and oligosaccharide chains in the entry. The first graphic for a chain summarises the proportions of the various outlier classes displayed in the second graphic. The second graphic shows the sequence view annotated by issues in geometry and electron density. Residues are color-coded according to the number of geometric quality criteria for which they contain at least one outlier: green = 0, yellow = 1, orange = 2 and red = 3 or more. A red dot above a residue indicates a poor fit to the electron density (RSRZ > 2). Stretches of 2 or more consecutive residues without any outlier are shown as a green connector. Residues present in the sample, but not in the model, are shown in grey.

- Molecule 1: Candida glabrata strain CBS138 chromosome C complete sequence

Chain A:

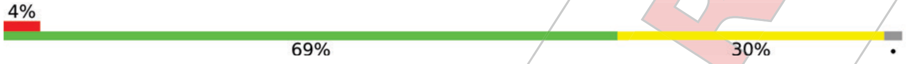

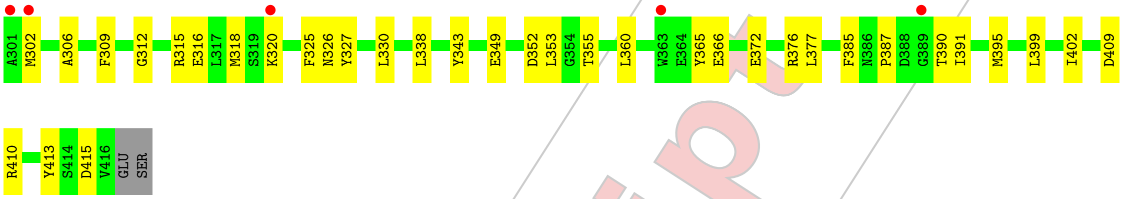

For Manuscript Review

4 Data and refinement statistics (i)

Property	Value	Source
Space group	I 41 2 2	Depositor
Cell constants<br>a, b, c, α, β, γ	67.58Å 67.58Å 152.83Å<br>90.00° 90.00° 90.00°	Depositor
Resolution (Å)	61.81 – 2.11<br>61.81 – 2.11	Depositor<br>EDS
% Data completeness<br>(in resolution range)	54.7 (61.81-2.11)<br>54.7 (61.81-2.11)	Depositor<br>EDS
R_{merge}	(Not available)	Depositor
R_{sym}	(Not available)	Depositor
$< I/\sigma(I) >$ [1]	1.26 (at 2.12Å)	Xtriage
Refinement program	PHENIX 1.20.1_4487	Depositor
R, R_{free}	0.257 , 0.287<br>0.250 , 0.281	Depositor<br>DCC
R_{free} test set	295 reflections (5.10%)	wwPDB-VP
Wilson B-factor (Å^2)	25.3	Xtriage
Anisotropy	0.187	Xtriage
Bulk solvent k_{sol}(e/Å^3), B_{sol}(Å^2)	0.33 , 52.4	EDS
L-test for twinning[2]	$< \|L\| > = 0.51$, $< L^2 > = 0.34$	Xtriage
Estimated twinning fraction	No twinning to report.	Xtriage
F_o,F_c correlation	0.88	EDS
Total number of atoms	1030	wwPDB-VP
Average B, all atoms (Å^2)	42.0	wwPDB-VP

Xtriage's analysis on translational NCS is as follows: *The largest off-origin peak in the Patterson function is 12.39% of the height of the origin peak. No significant pseudotranslation is detected.*

[1] Intensities estimated from amplitudes.

[2] Theoretical values of $< |L| >$, $< L^2 >$ for acentric reflections are 0.5, 0.333 respectively for untwinned datasets, and 0.375, 0.2 for perfectly twinned datasets.

For Manuscript Review

5 Model quality (i)

5.1 Standard geometry (i)

Bond lengths and bond angles in the following residue types are not validated in this section: Y7B

The Z score for a bond length (or angle) is the number of standard deviations the observed value is removed from the expected value. A bond length (or angle) with $|Z| > 5$ is considered an outlier worth inspection. RMSZ is the root-mean-square of all Z scores of the bond lengths (or angles).

Mol	Chain	Bond lengths		Bond angles	
		RMSZ	$\#|Z| > 5$	RMSZ	$\#|Z| > 5$
1	A	0.28	0/976	0.49	0/1322

There are no bond length outliers.

There are no bond angle outliers.

There are no chirality outliers.

There are no planarity outliers.

5.2 Too-close contacts (i)

In the following table, the Non-H and H(model) columns list the number of non-hydrogen atoms and hydrogen atoms in the chain respectively. The H(added) column lists the number of hydrogen atoms added and optimized by MolProbity. The Clashes column lists the number of clashes within the asymmetric unit, whereas Symm-Clashes lists symmetry-related clashes.

Mol	Chain	Non-H	H(model)	H(added)	Clashes	Symm-Clashes
1	A	950	0	909	33	0
2	A	31	26	0	1	0
3	A	23	0	0	0	0
All	All	1004	26	909	33	0

The all-atom clashscore is defined as the number of clashes found per 1000 atoms (including hydrogen atoms). The all-atom clashscore for this structure is 17.

All (33) close contacts within the same asymmetric unit are listed below, sorted by their clash magnitude.

Atom-1	Atom-2	Interatomic distance (Å)	Clash overlap (Å)
1:A:327:TYR:HA	1:A:330:LEU:CD1	2.12	0.79

Continued on next page...

For Manuscript Review

Continued from previous page...

Atom-1	Atom-2	Interatomic distance (Å)	Clash overlap (Å)
1:A:327:TYR:HA	1:A:330:LEU:HD13	1.65	0.79
1:A:320:LYS:H	1:A:320:LYS:HD2	1.53	0.73
1:A:413:TYR:CE1	1:A:415:ASP:HB2	2.27	0.69
1:A:352:ASP:OD1	1:A:355:THR:OG1	2.13	0.65
1:A:391:ILE:HG22	1:A:395:MET:HE2	1.77	0.64
1:A:413:TYR:HE1	1:A:415:ASP:CB	2.13	0.62
1:A:413:TYR:HE1	1:A:415:ASP:HB2	1.63	0.62
1:A:360:LEU:HD23	1:A:365:TYR:HD2	1.66	0.61
1:A:391:ILE:HG22	1:A:395:MET:CE	2.29	0.61
1:A:353:LEU:HD11	1:A:399:LEU:HD21	1.86	0.58
1:A:312:GLY:O	1:A:316:GLU:HG3	2.07	0.55
1:A:320:LYS:HD2	1:A:320:LYS:N	2.21	0.55
1:A:343:TYR:HA	1:A:385:PHE:CE1	2.43	0.54
1:A:349:GLU:HG2	1:A:377:LEU:HD21	1.89	0.54
1:A:325:PHE:HB2	1:A:395:MET:HG2	1.89	0.54
1:A:302:MET:HE2	1:A:306:ALA:CB	2.39	0.53
1:A:372:GLU:O	1:A:376:ARG:HG3	2.10	0.52
1:A:327:TYR:HA	1:A:330:LEU:HD11	1.90	0.51
1:A:302:MET:HG3	1:A:366:GLU:O	2.13	0.49
1:A:387:PRO:HD2	1:A:390:THR:HG21	1.95	0.48
1:A:325:PHE:HZ	1:A:402:ILE:HD11	1.79	0.47
1:A:326:ASN:O	1:A:330:LEU:HD12	2.17	0.45
1:A:391:ILE:HD12	1:A:391:ILE:N	2.31	0.45
1:A:302:MET:HG3	1:A:366:GLU:C	2.36	0.45
1:A:325:PHE:CB	1:A:395:MET:HG2	2.47	0.44
1:A:366:GLU:O	1:A:366:GLU:HG2	2.17	0.44
1:A:338:LEU:HD13	2:A:501:Y7B:C01	2.48	0.43
1:A:330:LEU:HD12	1:A:330:LEU:N	2.33	0.43
1:A:309:PHE:CZ	1:A:410:ARG:HA	2.53	0.42
1:A:315:ARG:HA	1:A:318:MET:HE2	2.00	0.42
1:A:302:MET:CE	1:A:306:ALA:HB1	2.50	0.41
1:A:302:MET:HE2	1:A:306:ALA:HB1	2.03	0.40

There are no symmetry-related clashes.

5.3 Torsion angles (i)

5.3.1 Protein backbone (i)

In the following table, the Percentiles column shows the percent Ramachandran outliers of the chain as a percentile score with respect to all X-ray entries followed by that with respect to entries

For Manuscript Review

of similar resolution.

The Analysed column shows the number of residues for which the backbone conformation was analysed, and the total number of residues.

Mol	Chain	Analysed	Favoured	Allowed	Outliers	Percentiles	
1	A	114/118 (97%)	112 (98%)	2 (2%)	0	100	100

There are no Ramachandran outliers to report.

5.3.2 Protein sidechains (i)

In the following table, the Percentiles column shows the percent sidechain outliers of the chain as a percentile score with respect to all X-ray entries followed by that with respect to entries of similar resolution.

The Analysed column shows the number of residues for which the sidechain conformation was analysed, and the total number of residues.

Mol	Chain	Analysed	Rotameric	Outliers	Percentiles	
1	A	104/106 (98%)	103 (99%)	1 (1%)	76	81

All (1) residues with a non-rotameric sidechain are listed below:

Mol	Chain	Res	Type
1	A	409	ASP

Sometimes sidechains can be flipped to improve hydrogen bonding and reduce clashes. There are no such sidechains identified.

5.3.3 RNA (i)

There are no RNA molecules in this entry.

5.4 Non-standard residues in protein, DNA, RNA chains (i)

There are no non-standard protein/DNA/RNA residues in this entry.

5.5 Carbohydrates (i)

There are no monosaccharides in this entry.

For Manuscript Review

5.6 Ligand geometry (i)

1 ligand is modelled in this entry.

In the following table, the Counts columns list the number of bonds (or angles) for which Mogul statistics could be retrieved, the number of bonds (or angles) that are observed in the model and the number of bonds (or angles) that are defined in the Chemical Component Dictionary. The Link column lists molecule types, if any, to which the group is linked. The Z score for a bond length (or angle) is the number of standard deviations the observed value is removed from the expected value. A bond length (or angle) with $|Z| > 2$ is considered an outlier worth inspection. RMSZ is the root-mean-square of all Z scores of the bond lengths (or angles).

Mol	Type	Chain	Res	Link	Bond lengths			Bond angles		
					Counts	RMSZ	#$\|Z\| > 2$	Counts	RMSZ	#$\|Z\| > 2$
2	Y7B	A	501	-	33,35,35	1.45	6 (18%)	43,50,50	1.39	4 (9%)

In the following table, the Chirals column lists the number of chiral outliers, the number of chiral centers analysed, the number of these observed in the model and the number defined in the Chemical Component Dictionary. Similar counts are reported in the Torsion and Rings columns. '-' means no outliers of that kind were identified.

Mol	Type	Chain	Res	Link	Chirals	Torsions	Rings
2	Y7B	A	501	-	-	2/16/33/33	0/5/5/5

All (6) bond length outliers are listed below:

Mol	Chain	Res	Type	Atoms	Z	Observed(Å)	Ideal(Å)
2	A	501	Y7B	C19-N04	3.80	1.45	1.35
2	A	501	Y7B	C10-N01	3.67	1.46	1.35
2	A	501	Y7B	C09-C07	-2.68	1.37	1.41
2	A	501	Y7B	C17-N03	2.47	1.45	1.38
2	A	501	Y7B	O03-C19	-2.27	1.18	1.23
2	A	501	Y7B	C20-N04	2.22	1.46	1.41

All (4) bond angle outliers are listed below:

Mol	Chain	Res	Type	Atoms	Z	Observed(^{o})	Ideal(^{o})
2	A	501	Y7B	C03-C05-N01	4.62	115.48	110.04
2	A	501	Y7B	C14-N03-C13	3.82	119.94	111.52
2	A	501	Y7B	C18-C10-N01	2.98	121.73	118.26
2	A	501	Y7B	C11-C08-C06	-2.05	119.07	121.06

There are no chirality outliers.

All (2) torsion outliers are listed below:

For Manuscript Review

Mol	Chain	Res	Type	Atoms
2	A	501	Y7B	C21-C17-N03-C14
2	A	501	Y7B	C22-C17-N03-C14

There are no ring outliers.

1 monomer is involved in 1 short contact:

Mol	Chain	Res	Type	Clashes	Symm-Clashes
2	A	501	Y7B	1	0

The following is a two-dimensional graphical depiction of Mogul quality analysis of bond lengths, bond angles, torsion angles, and ring geometry for all instances of the Ligand of Interest. In addition, ligands with molecular weight > 250 and outliers as shown on the validation Tables will also be included. For torsion angles, if less then 5% of the Mogul distribution of torsion angles is within 10 degrees of the torsion angle in question, then that torsion angle is considered an outlier. Any bond that is central to one or more torsion angles identified as an outlier by Mogul will be highlighted in the graph. For rings, the root-mean-square deviation (RMSD) between the ring in question and similar rings identified by Mogul is calculated over all ring torsion angles. If the average RMSD is greater than 60 degrees and the minimal RMSD between the ring in question and any Mogul-identified rings is also greater than 60 degrees, then that ring is considered an outlier. The outliers are highlighted in purple. The color gray indicates Mogul did not find sufficient equivalents in the CSD to analyse the geometry.

Ligand Y7B A 501

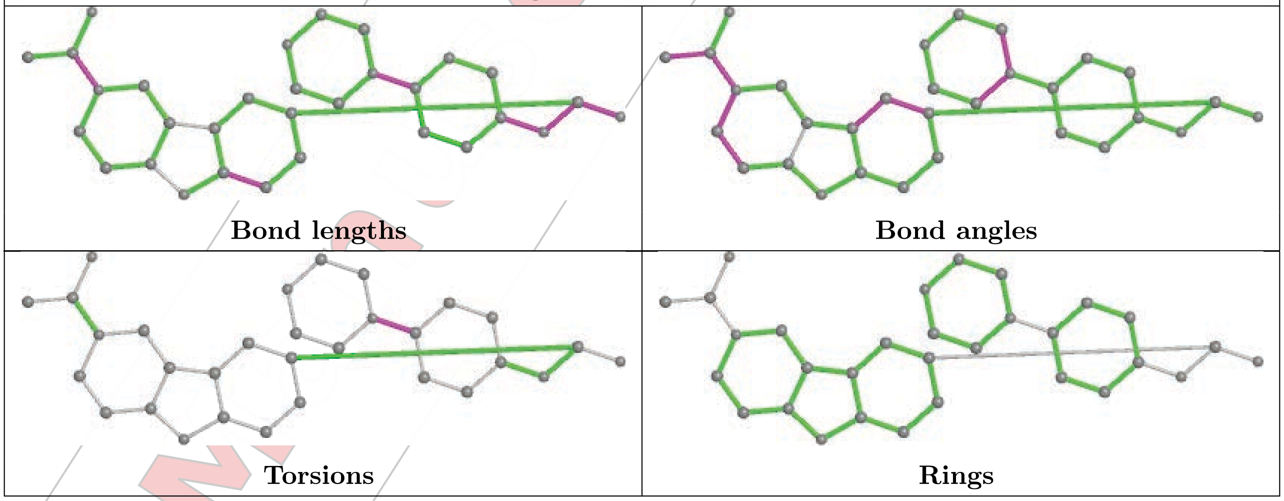

5.7 Other polymers (i)

There are no such residues in this entry.

For Manuscript Review

5.8 Polymer linkage issues (i)

There are no chain breaks in this entry.

For Manuscript Review

6 Fit of model and data (i)

6.1 Protein, DNA and RNA chains (i)

In the following table, the column labelled '#RSRZ> 2' contains the number (and percentage) of RSRZ outliers, followed by percent RSRZ outliers for the chain as percentile scores relative to all X-ray entries and entries of similar resolution. The OWAB column contains the minimum, median, 95th percentile and maximum values of the occupancy-weighted average B-factor per residue. The column labelled 'Q< 0.9' lists the number of (and percentage) of residues with an average occupancy less than 0.9.

Mol	Chain	Analysed	<RSRZ>	#RSRZ>2			OWAB(Å^2)	Q<0.9
1	A	116/118 (98%)	0.43	5 (4%)	35	41	16, 45, 69, 76	0

All (5) RSRZ outliers are listed below:

Mol	Chain	Res	Type	RSRZ
1	A	363	TRP	4.0
1	A	302	MET	3.5
1	A	301	ALA	3.1
1	A	389	GLY	2.2
1	A	320	LYS	2.1

6.2 Non-standard residues in protein, DNA, RNA chains (i)

There are no non-standard protein/DNA/RNA residues in this entry.

6.3 Carbohydrates (i)

There are no monosaccharides in this entry.

6.4 Ligands (i)

In the following table, the Atoms column lists the number of modelled atoms in the group and the number defined in the chemical component dictionary. The B-factors column lists the minimum, median, 95th percentile and maximum values of B factors of atoms in the group. The column labelled 'Q< 0.9' lists the number of atoms with occupancy less than 0.9.

Mol	Type	Chain	Res	Atoms	RSCC	RSR	B-factors(Å^2)	Q<0.9
2	Y7B	A	501	31/31	0.93	0.19	14,30,114,114	0

The following is a graphical depiction of the model fit to experimental electron density of all

For Manuscript Review

instances of the Ligand of Interest. In addition, ligands with molecular weight > 250 and outliers as shown on the geometry validation Tables will also be included. Each fit is shown from different orientation to approximate a three-dimensional view.

Electron density around Y7B A 501:

2mF$_o$-DF$_c$ (at 0.7 rmsd) in gray
mF$_o$-DF$_c$ (at 3 rmsd) in purple (negative) and green (positive)

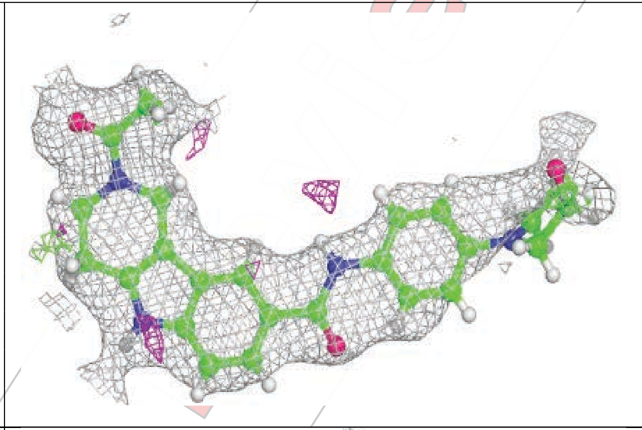

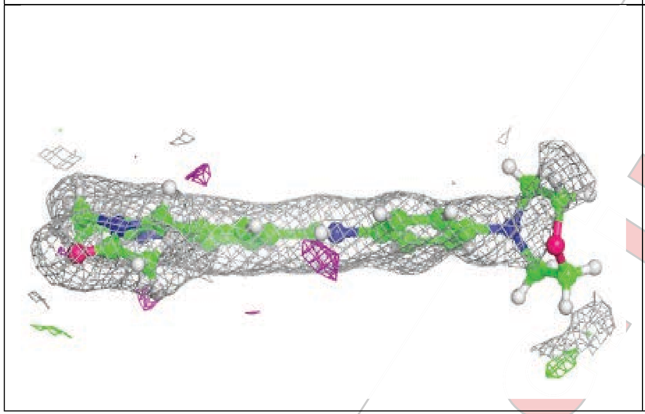

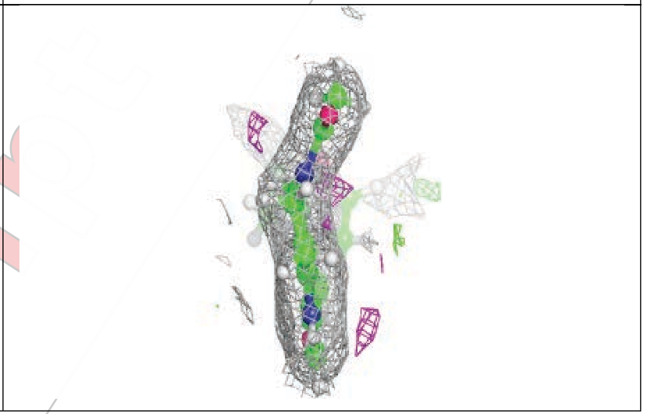

6.5 Other polymers (i)

There are no such residues in this entry.

For Manuscript Review
